# Unveiling the Use of 1,1-Bis(triflyl)ethylene as CF_3_SO_2_CH=CH_2_ Source with the Assistance of (*n*-Bu)_4_NF: Synthesis of 3-[(Trifluoromethyl)sulfonyl]cyclobut-1-enes

**DOI:** 10.1021/acs.orglett.4c01514

**Published:** 2024-05-20

**Authors:** A. Sonia Petcu, Carlos Lázaro-Milla, José M. Alonso, Pedro Almendros

**Affiliations:** † Instituto de Química Orgánica General, IQOG, CSIC, Juan de la Cierva 3, 28006 Madrid, Spain; ‡ Grupo de Lactamas y Heterociclos Bioactivos, Departamento de Química Orgánica, Unidad Asociada al CSIC, Facultad de Química, Universidad Complutense de Madrid, 28040 Madrid, Spain

## Abstract

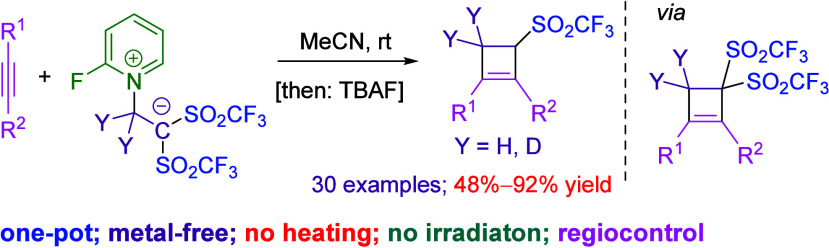

Allylic sulfone-embedded cyclobutenes have been prepared in one pot from alkynes. The carbocycle and the alkenyl sulfone moieties were installed through consecutive bis(triflyl)cyclobutenylation of a triple bond and tetra-*n*-butylammonium fluoride (TBAF)-assisted hydrodesulfonylation of an allylic bis(sulfone). It is noteworthy that 1,1-bis(triflyl)ethylene acts as a CF_3_SO_2_CH=CH_2_ source for the first time.

The biological activities of allylic sulfones, combined with its use as synthetic precursors for the preparation of advanced materials and complex bioactive products, encourage chemists to explore novel protocols for the synthesis of molecules containing the allylic sulfone moiety (Scheme 1a,b).^1^ The cyclobutene core is of industrial and biological relevance.^2^ The introduction of both moieties into the same strained nucleus should significantly increase the pharmacological and chemical interest. The mild base tetra-*n*-butylammonium fluoride (TBAF) is a source of fluoride that is employed as a desilylation reagent and a phase transfer catalyst. The radical- or metal-promoted reductive desulfonylation reaction (Scheme 1c) and the TBAF-driven silyl ether deprotection are well known.^3,4^ While the deboronation with TBAF has been recently achieved,^5^ the TBAF-assisted desulfonylation of sulfones, to the best of our knowledge, has not been reported. It should be noted that organic molecules having a single triflyl group, namely triflones, have attracted interest for applications as therapeutics, ligands, or advanced materials.^6^ We recently reported the desulfinative palladium-catalyzed Tsuji–Trost reaction of *gem*-bis(triflyl)cyclobutenes to access vinyl sulfone-attached cyclobutenes (Scheme 1d, right),^7a^ while Yanai et al. described a desulfinative Friedel–Crafts reaction (Scheme 1d, left).^7b^ We wish to report herein that the combined use of betaine **1** with TBAF allows the one-pot preparation of a class of hitherto unknown allylic sulfones, namely, 3-[(trifluoromethyl)sulfonyl]cyclobut-1-enes (Scheme 1e). In this way, the use of betaine **1** as synthetic equivalent of CF_3_SO_2_CH=CH_2_ has been unraveled.^8^

**Scheme 1:**
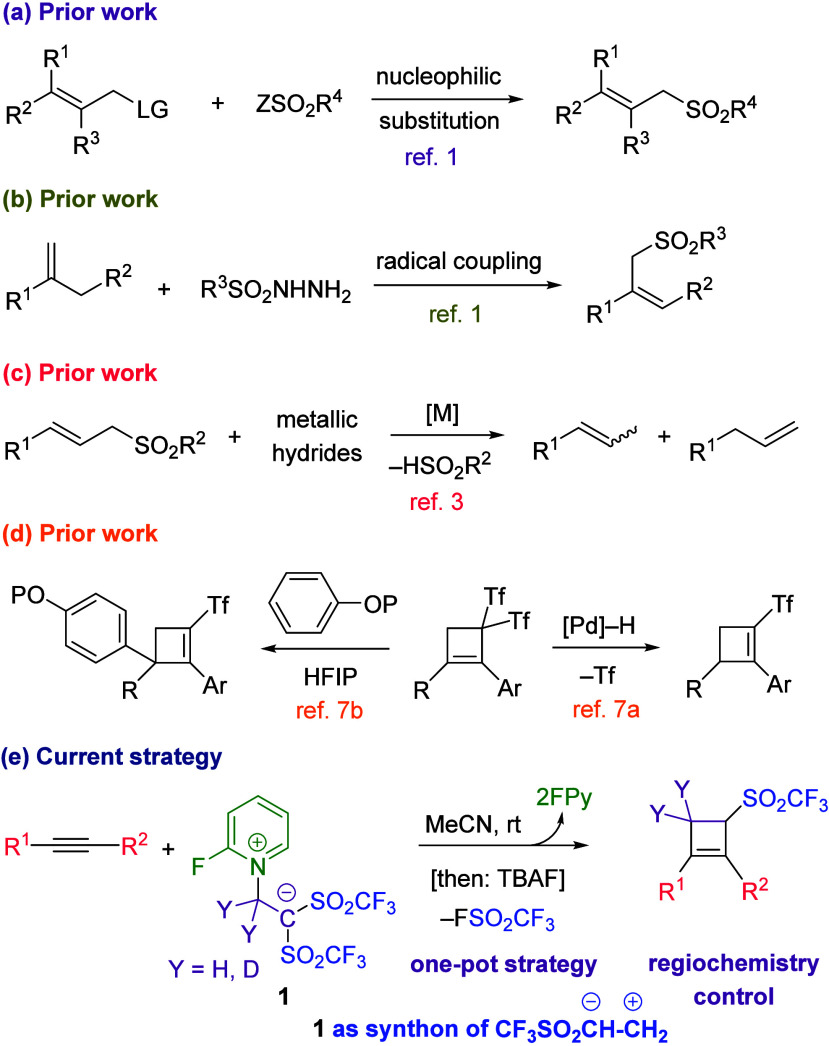
Prior Art and Synopsis of the Present Strategy

Yanai et al. pioneered the use of betaine **1**, a shelf-stable and easy-to-handle source of (CF_3_SO_2_)_2_C=CH_2_, in bis[(trifluoromethyl)sulfonyl]ethylation reactions.^9^ Starting from the appropriate alkyne and reagent **1**, the synthesis of 1,2-disubstituted 3-[(trifluoromethyl)sulfonyl]cyclobut-1-enes using a one-pot protocol would be very appealing regarding effectiveness because costly intermediate separations and purification steps could be circumvented. This challenging approach should surpass the difficulty of how to guide and to modulate the reactivity of the reagents involved in this transformation. Fortunately, the reaction of alkyne **2a** with betaine **1** in acetonitrile at room temperature^10^ followed by addition of TBAF (1 M in THF) smoothly yielded the desired CF_3_SO_2_-functionalized cyclobut-1-ene **3a** (Table 1). Our solvent screening method identified acetonitrile as the most suitable reaction medium. Change in the nature of the additive has a tremendous impact on the reaction because replacement of TBAF with different compounds, such as tetrabutylammonium iodide (TBAI), CsF, Tf_2_NH, 1,4-diazabicyclo[2.2.2]octane (DABCO), and InBr_3_, was fruitless or did not proceed to completion (Table 1), which points out the decisive role of both the fluoride anion and the mild basic character of TBAF for promoting the required transformation.

**Table 1:**
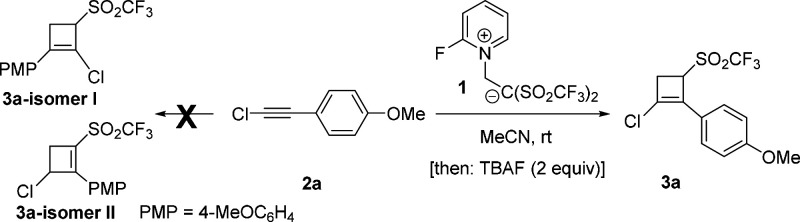
Reaction Development

entry	deviation from standard conditions	yield^a^
1	none	79
2	TBAI as additive	0
3	CsF as additive	15
4	Tf_2_NH as additive	0
5	DABCO as additive	0
6	InBr_3_ as additive	0
7	60 °C	70
8	THF as solvent	20

a Yield of pure, isolated product with correct analytical and spectral data.

The inspection of the substrate scope was performed through the evaluation of various substituted alkynes **2** (Scheme 2).

**Scheme 2:**
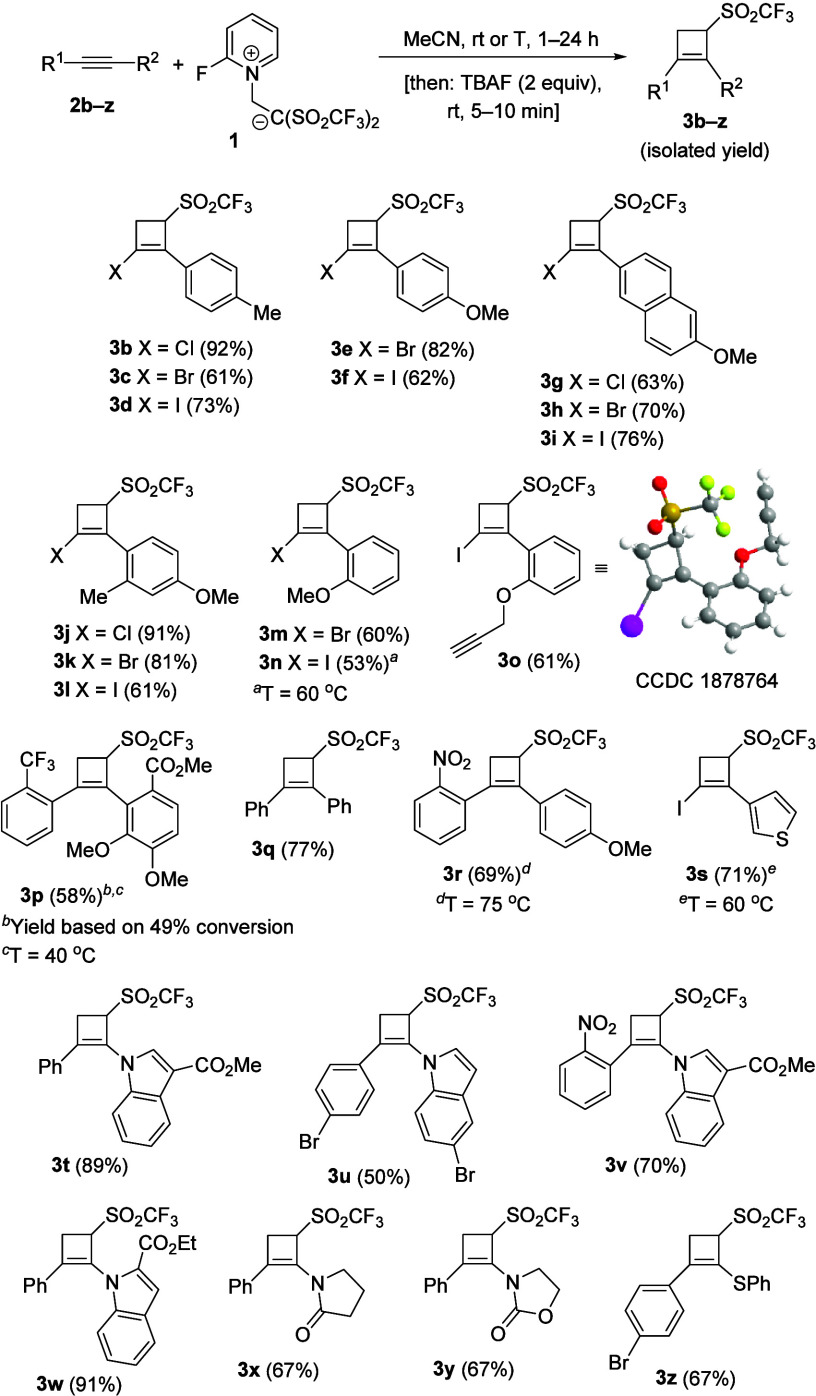
Controlled Synthesis of 3-(Triflyl)cyclobut-1-enes **3b**–**z**

To assess both steric and electronic influence, a range of substituents bearing different steric and electronic demands was placed at the alkyne moiety. Variation of the steric hindrance had little impact on the product formation as bulky aryl groups, such as naphthyl (**3g**–**i**) and *ortho*-substituted phenyls (**3j**–**p**), were well accommodated. The sequence also defeats some electronic limitations by even providing final cyclobutenes **3** bearing electron-deficient substituents such as **3p**, **3r**, and **3v**. However, it should be noted that although electron-poor moieties may participate in the reaction sequence, the regiochemical control is imparted by the more electron-rich moiety. It is noteworthy that, in addition to the exquisite regiocontrol, total chemoselectivity was attained during the formation of cyclobutene **3o**, which kept unreacted the alkyne group in the propargyl ether moiety. As we previously reported,^10a^ terminal alkynes bearing an alkyl substituent are not able to react with betaine **1**, probably because the resulting alkenyl carbocation intermediates are not stabilized enough. Alkynes **2** having heterocyclic nuclei that are either aromatic, such as thiophene and indole, or nonaromatic, such pyrrolidone and oxazolidone, also furnished strained carbocycles **3s**–**y** in an efficient way. Equally, heteroatomic functionalities, such as PhS and halogens (Cl, Br, and I), were also productive for the formation of highly functionalized cyclobutenes **3a**–**o** and **3s**. Recrystallization of compound **3o** provided single crystals, which allowed us to confirm the structure by X-ray diffraction analysis.

Taking into account that the applications of deuterium labeling in different areas of chemistry is growing rapidly, the access to deuterated molecules bearing structural diversity is in high demand. In this context, we wondered whether deuterated betaine^11^**1-**d_2_ and TBAF could be jointly employed for the preparation of the deuterated 3-(triflyl)cyclobut-1-ene framework. Indeed, the aforementioned combination allowed the synthesis of deuterated adducts **3**-*d*_2_ (Scheme 3). It is important to mention that full transfer of both deuterium atoms from betaine **1**-*d*_2_ to cyclobutenes **3**-*d*_2_ occurred as loss of deuterium from **1**-*d*_2_ could indicate other mechanistic pathways occurring in the reaction.

**Scheme 3:**
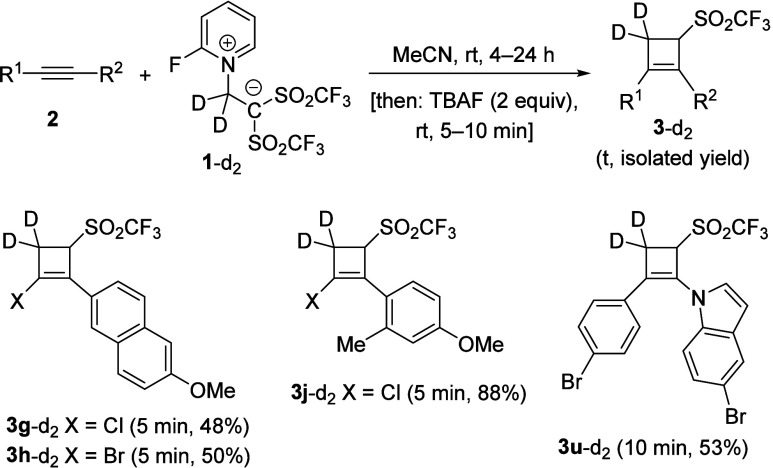
Controlled Preparation of Labeled 3-(Triflyl)cyclobut-1-enes **3**-*d*_2_

The addition of 3 Å molecular sieves to the reaction mixture did not influence the regioselectivity but gave rise to the formation of **3a** in moderately diminished yield, which points out some role of adventitious water for the success of this transformation. The water available in HPLC grade solvents was suitable for a satisfactory advance of the reaction. A plausible reaction pathway is proposed as depicted in Scheme 4. In solution, betaine **1** releases neutral 1,1-bis[(trifluoromethyl)sulfonyl]ethene. Subsequent regioselective [2 + 2] cyclization between the electron-deficient alkene and alkynes **2** furnishes bis[(trifluoromethyl)sulfonyl]cyclobutenes **INT-I**. Next, nucleophilic attack of the fluoride toward the sulfur atom of one of the sulfone groups gives rise to species **INT-II**,^12^ which evolves to intermediate **INT-III** by loss of trifluoromethanesulfonyl fluoride.^13^ Probably, the driving force for the formation of α-triflylated allylic carbanion **INT-III** is both the stability imparted by the delocalization of the negative charge of the allylic carbanion through the cyclobutene ring and the strong inductive effect of the electron-withdrawing triflyl group. Final protonation^14,15^ without concomitant allylic rearrangement forms allyl sulfone-embedded cyclobutenes **3**. To support the mechanistic proposal of the one-pot process, the isolated *gem*-bis(triflyl)cyclobutene **INT-Ia** (R^1^ = Cl; R^2^ = 4-MeOC_6_H_4_)^10b^ was solved in acetonitrile and treated with TBAF (2 equiv) to provide desulfonylated cyclobutene **3a** (92% yield) after 5 min.

**Scheme 4:**
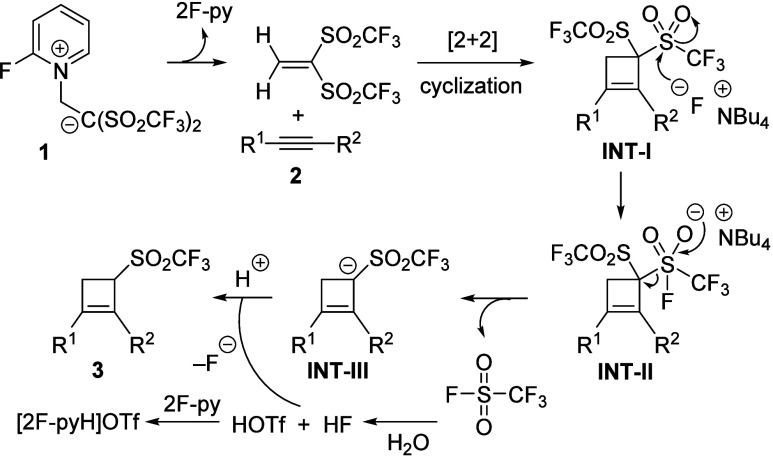
Rationalization for the Direct Formation of 1,2-Disubstituted 3-[(Trifluoromethyl)sulfonyl]cyclobut-1-enes **3**

To additionally illustrate the practicality of the above procedure, an experiment was carried out starting from one gram of alkyne **2a** under standard conditions, which gave rise to 3-(triflyl)cyclobut-1-ene **3a** in 75% yield (Scheme 5a). Besides, several postsynthetic modifications were performed in selected adducts **3**. Conditions were found for the chemoselective C–I bond cleavage following both catalytic hydrogenation and visible light/tris(trimethylsilyl)silane-mediated reduction to provide 2-unsubstituted cyclobut-1-enes **4d** and **4i** (Scheme 5b). 1,2-Diaryl-3-(triflyl)cyclobut-1-enes **5i**, **5k**, and **5n** were obtained when iodo-cyclobutenes **3i**,**n** and bromo-cyclobutene **3k** were coupled with (4-methoxyphenyl)boronic acid under Suzuki conditions at 40–60 °C (Scheme 5c). It is noteworthy that the acyclic (*E*)-3-(aryl)-2-(*p*-tolyl)but-2-enals **6da** and **6db** were formed after exposure of 1-iodocyclobutene **3d** to Suzuki coupling under forcing reaction conditions (Scheme 5d). A speculative rationalization for the formation of tetrasubstituted enals **6** is delineated in Scheme S12 (see the Supporting Information).

**Scheme 5:**
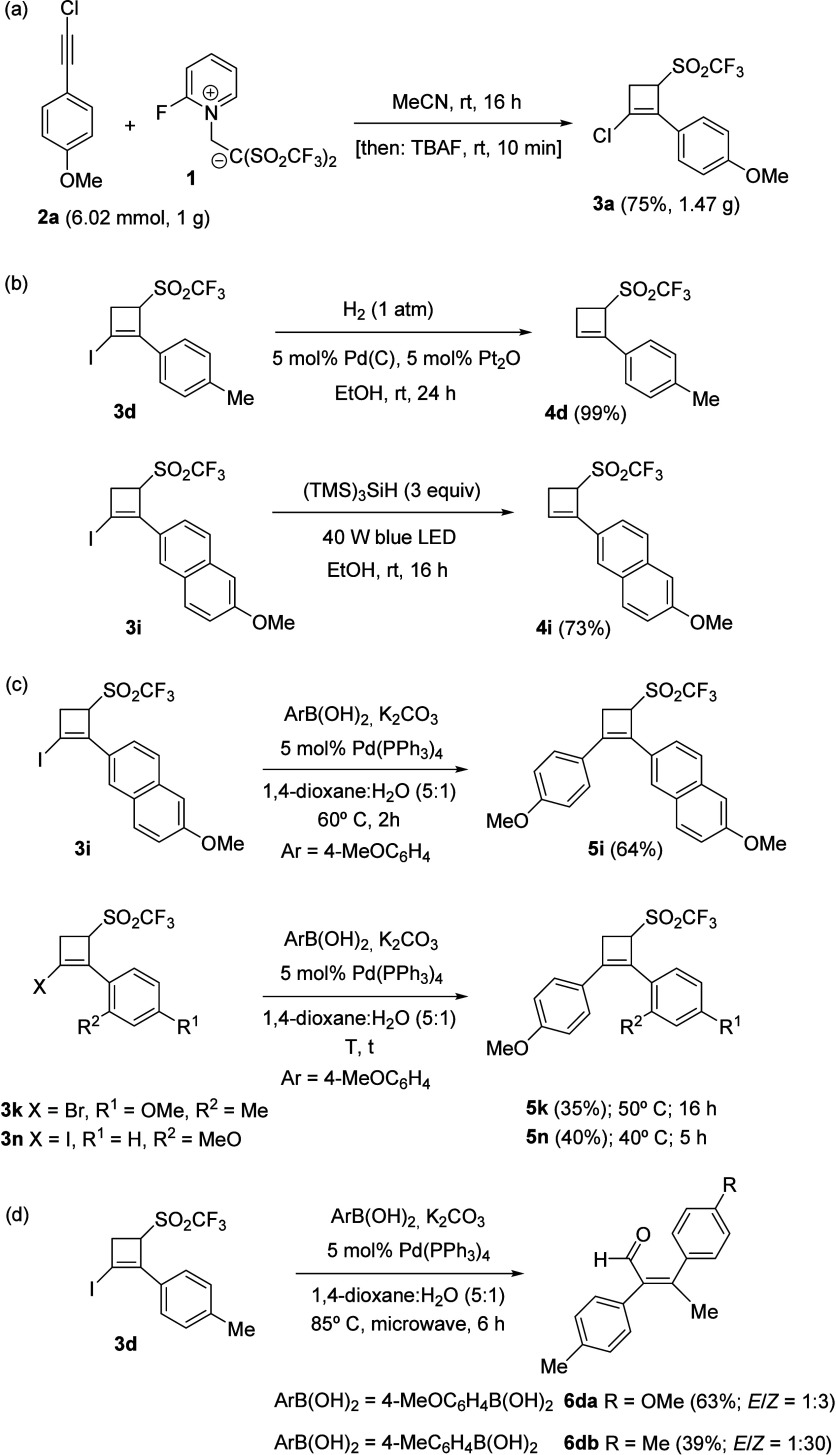
Gram-Scale Experiment and Postsynthetic Modifications

Interestingly, the presence of an alkyl group at the alkyne moiety resulted in a different reaction outcome because desulfonylation occurred but with concomitant exocyclic alkene formation. Indeed, cyclobutene **7a** was formed from alkyne **2a-alkyl** after TBAF treatment (Scheme 6).

**Scheme 6:**
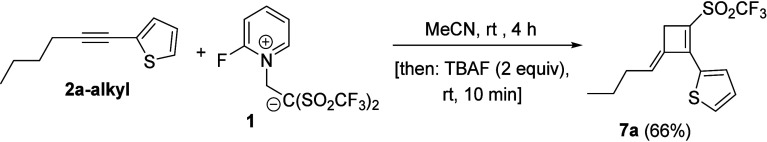
Controlled Synthesis of 2-(Triflyl)cyclobut-1-ene **7a**

To summarize, we have developed the one-pot bis(triflyl)cyclobutenylation of triple bonds and the TBAF-assisted hydrodesulfonylation of the resulting allylic bis(sulfones) for the straightforward synthesis of polysubstituted 3-[(trifluoromethyl)sulfonyl]cyclobut-1-enes. These allyl sulfone-embedded cyclobutenes^8^ were obtained in good yields and total regioselectivity using readily available precursors.

## Data Availability

The data underlying this study are available in the published article and its Supporting Information.

## Supporting Information Available

Experimental procedures, characterization data of new compounds, copies of NMR spectra, and crystallographic data (PDF)
