# Supplementary material for: Unveiling the Use of 1,1-Bis(triflyl)ethylene as CF3SO2CH=CH2 Source with the Assistance of (n-Bu)4NF: Synthesis of 3-[(Trifluoromethyl)sulfonyl]cyclobut-1-enes
Source: Org Lett. 2024 May 20;26(21):4560–5. doi: 10.1021/acs.orglett.4c01514 (PMC11148847; doi:10.1021/acs.orglett.4c01514)

Supplementary Information for the paper

Unveiling the Use of 1,1-Bis(triflyl)ethylene as $CF_3SO_2CH=CH_2$ Source with the Assistance of (*n*-Bu)$_4$NF. Synthesis of 3-((Trifluoromethyl)sulfonyl)cyclobut-1-enes

A. Sonia Petcu,[†,‡] Carlos Lázaro-Milla,[‡] José M. Alonso,*[,‡] and Pedro Almendros*[,†]

[†]Instituto de Química Orgánica General, IQOG, CSIC, Juan de la Cierva 3, 28006-Madrid, Spain

[‡]Grupo de Lactamas y Heterociclos Bioactivos, Departamento de Química Orgánica, Unidad Asociada al CSIC, Facultad de Química, Universidad Complutense de Madrid, 28040-Madrid, Spain

E-mail: josalo08@ucm.es; palmendros@iqog.csic.es

Table of Contents

General Methods S2

Experimental Section S2–S30

Mechanistic Scheme S12 S30

Crystallographic Data S31–S33

^{1}H NMR, ^{2}H NMR, ^{13}C NMR, and ^{19}F NMR Spectra S34–S122

General Methods: ^1H NMR and ^{13}C NMR spectra were recorded on a Bruker Avance AVIII-700 with cryoprobe, Bruker AMX-500, Bruker Avance-300, or Varian VRX-300S. NMR spectra were recorded in $CDCl_3$ solutions, except otherwise stated. Chemical shifts are given in ppm relative to TMS (^1H, 0.0 ppm), or $CDCl_3$ (^1H, 7.27 ppm; ^{13}C, 76.9 ppm), or acetone-d_6 (^1H, 2.05 ppm; ^{13}C, 206.3 ppm), or C_6D_6 (^1H, 7.16 ppm; ^{13}C, 128.0 ppm), or CD_3CN (^1H, 1.94 ppm; ^{13}C, 118.2 ppm), or DMSO-d_6 (^1H, 2.50 ppm; ^{13}C, 39.5 ppm). Chemical shifts in ^{19}F are given in ppm relative to (trifluoromethyl)benzene ($C_6H_5CF_3$) in $CDCl_3$ (^{19}F, –63.7 ppm). Chemical shifts in ^2H are given in ppm relative to $CDCl_3$ in $CDCl_3$ (^2H, 7.26 ppm). Structural assignments were made with additional information from gCOSY, gHMQC, gHSQC, edited-gHSQC, and gHMBC experiments. Low and high resolution mass spectra were taken on an AGILENT 6520 Accurate-Mass QTOF LC/MS spectrometer using the electronic impact (EI) or electrospray modes (ES) unless otherwise stated. IR spectra were recorded on a FT/IR4X JASCO spectrometer. All commercially available compounds were used without further purification. Microwave irradiation was carried out in a Monowave 300 from Anton Paar GmbH. The reaction temperatures during microwave heating were measured with an internal infrared sensor. Syntheses in the microwave reactor were conducted in sealed reaction vessels. For light-promoted reactions, the reaction vessel (borosilicate glass) was placed about 2 cm from a Kessil PR160L lamp (427 nm) and stirred (a fan was used to dissipate the heat generated by the lamp). Column chromatography was carried out using silica gel 60, 0.04-0.06 mm, for flash chromatography (230-400 mesh ASTM) provided by Scharlau. For reactions that require heating, a heating-on block was used. All commercially available compounds were used without further purification.

Experimental procedures

Pyridinium salt **1** was synthesized according to a literature procedure: Yanai, H.; Takahashi, Y.; Fukaya, H.; Dobashi, Y.; Matsumoto, T. *Chem. Commun*. **2013**, *49*, 10091.

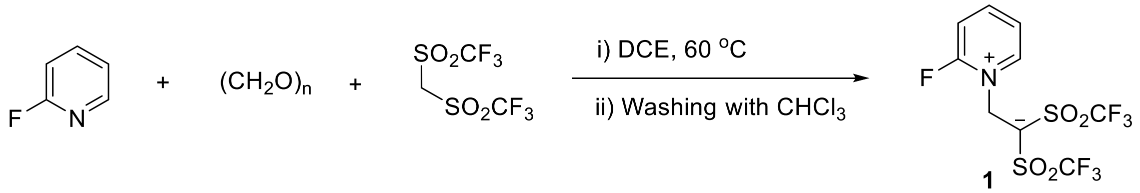

Scheme S1. Synthesis of Yanai's reagent **1**.

To a solution of Tf_2CH_2 (281 mg, 1.00 mmol) in 1,2-dichloroethane (6.0 mL), paraformaldehyde (90% purity, 73.0 mg, 2.19 mmol) and 2-fluoropyridine (172 µL, 2.00 mmol) were added at room

temperature. After being stirred for 8 h at 60 °C, the reaction mixture was concentrated under reduced pressure. The resulting residue was washed with $CHCl_3$ (1.0 mL x 3) to give zwitterion **1** in 91% yield (356 mg, 0.915 mmol).

Alkynes **2** were prepared following the corresponding general method, except alkyne **2q**, which was purchased from Merck Chemicals and used without further purification.

A) General procedure for the synthesis of chloroalkynes (2a,[1] 2b,[1] 2g,[2] 2j[3]):

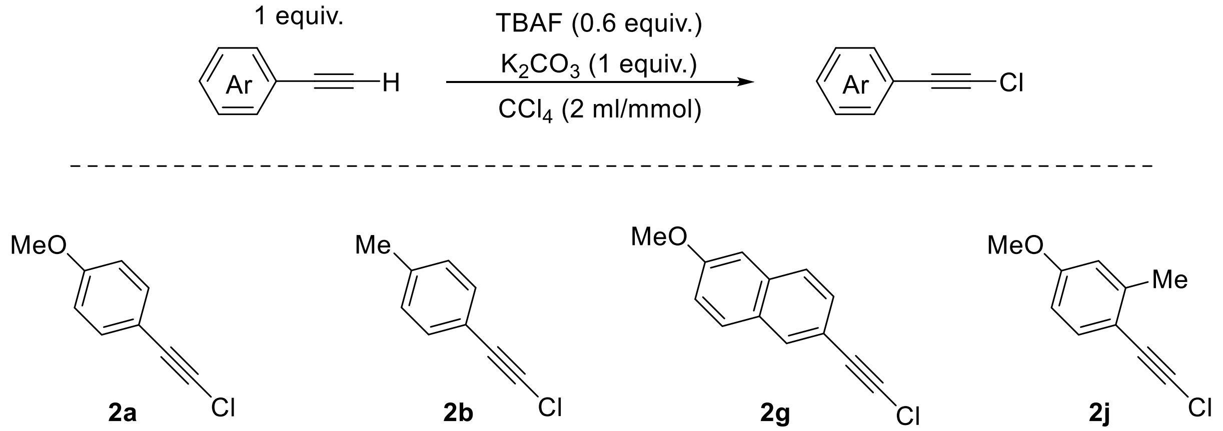

Scheme S2. Synthesis of chloroalkynes **2a**, **2b**, **2g**, **2j**.

A solution of the corresponding terminal alkyne (1 equiv.) was stirred in a round-bottom flask with potassium carbonate (1 equiv.) in CCl_4 (2 mL/mmol) under argon atmosphere. TBAF (0.6 equiv, 1M solution in THF) was added dropwise and the mixture was stirred at 35 °C until reaction completion (TLC). The reaction was quenched with MeOH and HCl (1N). Then, the aqueous layer was extracted with AcOEt. The combined organic layers were dried over $MgSO_4$ and the solvent was removed under reduced pressure. The crude product was purified by flash chromatography using hexanes/AcOEt mixtures. The NMR data of products **2** agreed with the ones previously described in the literature.

B) General procedure for the synthesis of bromoalkynes (2c,[4] 2e,[5] 2h[6], 2k,[3] 2m[7]) and iodoalkynes (2d,[3]2o,[8] 2s[9]):

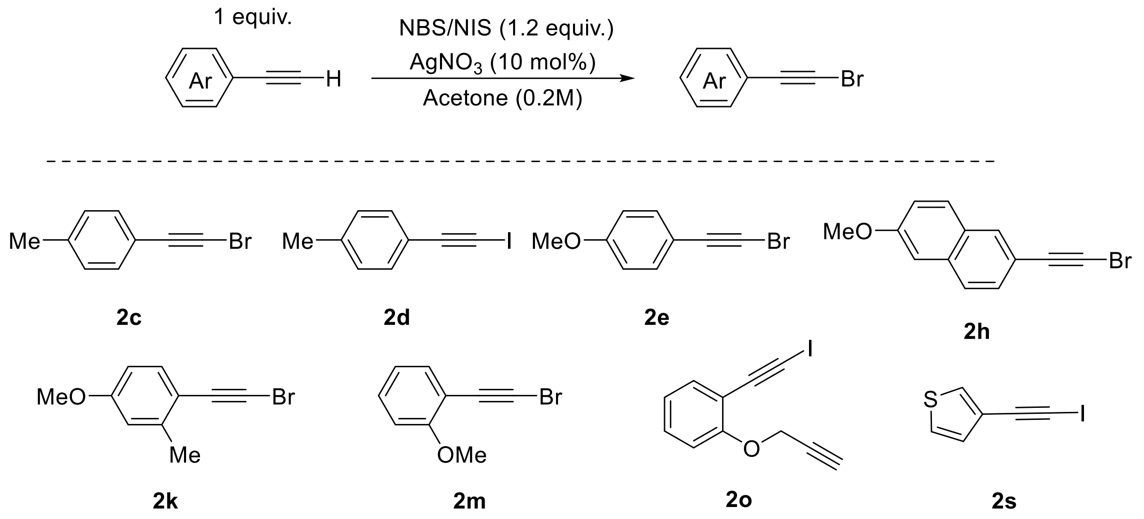

Scheme S3. Synthesis of bromoalkynes **2c**, **2e**, **2h**, **2k, 2m** and iodoalkynes **2d**, **2o, 2s**.

NBS or NIS (1.2 equiv.) and silver nitrate (0.1 equiv.) were added to a solution of the corresponding terminal alkyne in acetone. The reaction mixture was stirred at room temperature until reaction completion (TLC). The reaction was quenched with water and the aqueous layer was extracted with DCM. The combined organic layers were dried over $MgSO_4$ and the solvent was removed under reduced pressure. The crude product was purified by flash chromatography using hexanes/AcOEt mixtures. The NMR data of products **2** agreed with the ones previously described in the literature.

C) General procedure for the synthesis of iodoalkynes (2f,[3] 2i,[10] 2l,[3] 2n[11]):

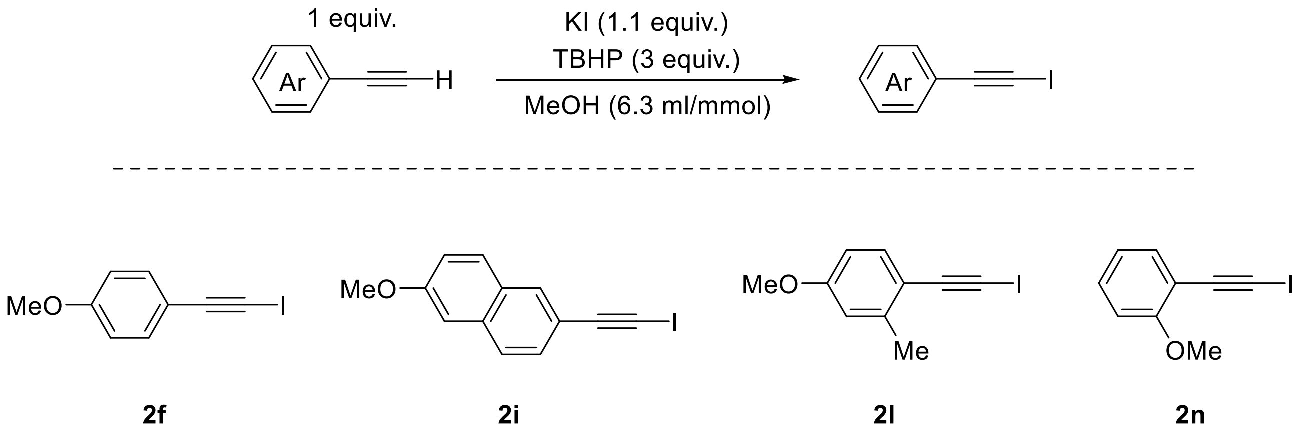

Scheme S4. Synthesis of iodoalkynes **2f**, **2i**, **2l**, **2n**.

A solution of the corresponding terminal alkyne (1 equiv.), and potassium iodide (1.1 equiv.) in methanol (6.3 mL/mmol) was stirred at room temperature. Then, a solution of TBHP (3. equiv., 70 wt% in water) was added and the reaction was stirred at room temperature until the reaction was completed (TLC). The mixture was quenched with saturated aqueous $Na_2S_2O_3$ solution, washed with brine and extracted with AcOEt. The combined organic layers were dried over $MgSO_4$ and the solvent was removed under reduced pressure. The crude product was purified by flash chromatography using hexanes/AcOEt mixtures. The NMR data of products **2** agreed with the ones previously described in the literature.

D) General procedure for the synthesis of alkynes 2p,[12] 2r[13].

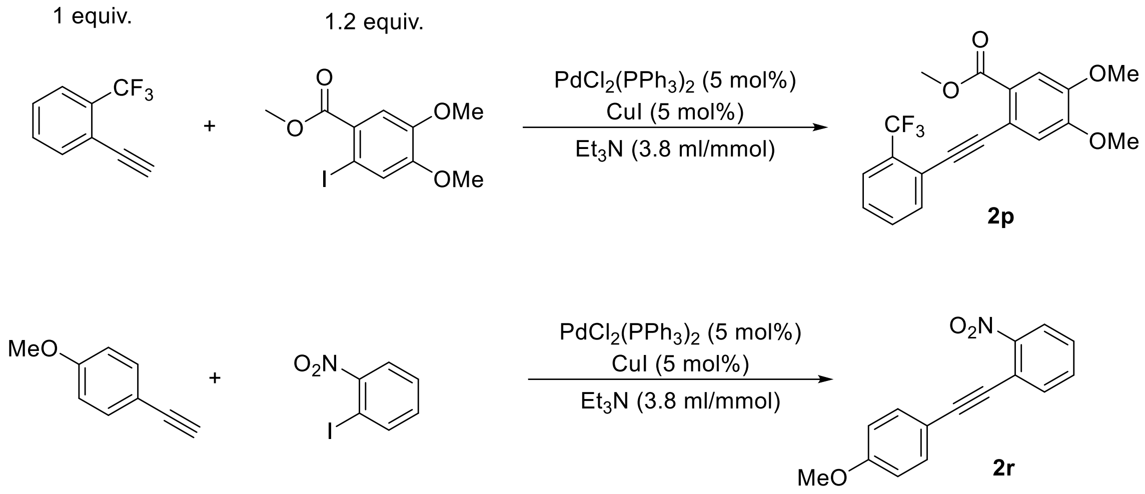

Scheme S5. Synthesis of **2p** and **2r**.

In an oven-dried round bottom flask was placed methyl 2-iodo-4,5-dimethoxybenzoate (1.2 equiv.), CuI (5 mol%) and $PdCl_2(PPh_3)_2$ (5 mol%). Then, the reaction was purged under argon atmosphere and TEA (3.8 mL/mmol) was added. The mixture was stirred at room temperature for 5 minutes. After that time, the 2-ethynyl-α,α,α-trifluorotoluene (1 equiv.) was added in one portion and the reaction was stirred at the same temperature until disappearance of the starting material (TLC). The crude reaction mixture was filtered through a celite pad, and the solvent was evaporated under reduced pressure. The mixture was purified on column chromatography using hexanes/AcOEt mixtures. The NMR data obtained for products **2** agreed with the ones previously described in the literature.

E) General procedure for the synthesis of alkynes 2t,[14] 2u,[14] 2v,[14] 2w,[15].

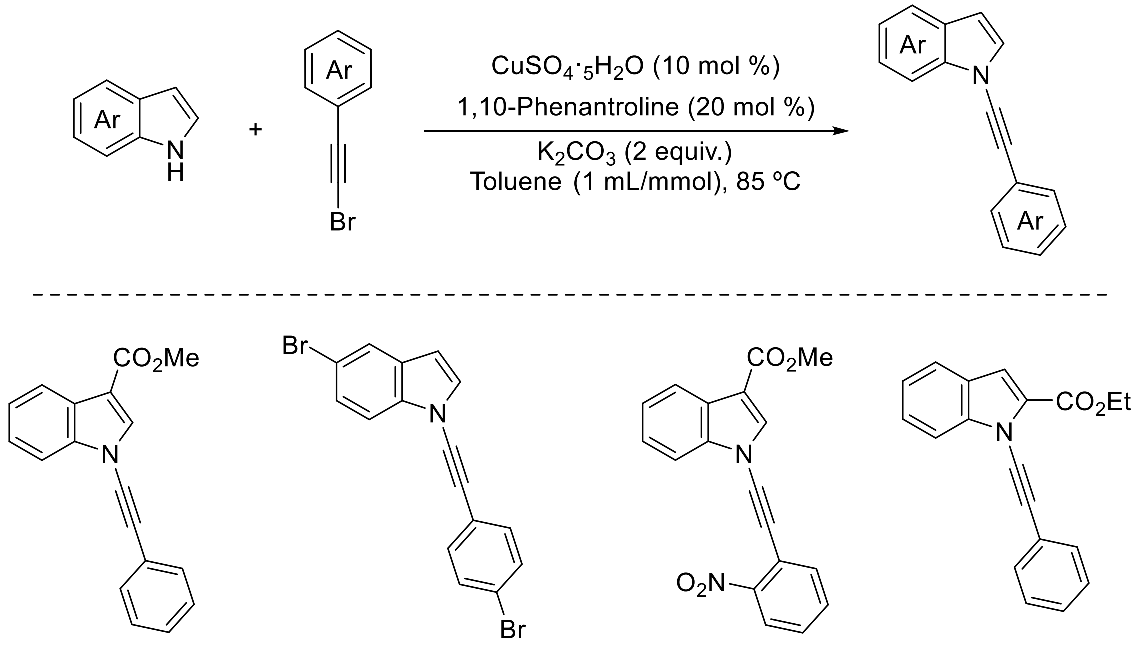

2t **2u** **2v** **2w**

Scheme S6. Synthesis of **2t**, **2u**, **2v**, **2w**.

A solution of the corresponding bromoalkyne (1.1 equiv.), the appropriate indole derivative (1 equiv.), K_2CO_3 (2 equiv.), $CuSO_4 \cdot 5H_2O$ (10 mol %), and 1,10-phenanthroline (20 mol %) was dissolved in anhydrous toluene (1 mL/mmol). The reaction mixture was purged under argon atmosphere and heated to 85 °C until reaction completion (TLC). Then, the reaction mixture was cooled to room temperature and diluted with AcOEt. The resulting mixture was filtered through a celite pad and the filtrate was concentrated *in vacuo*. The crude residue was purified using silica gel column flash chromatography. The NMR data of products **2** agreed with the ones previously described in the literature.

F) General procedure for the synthesis of ynamides (2x,[16] 2y[17]):

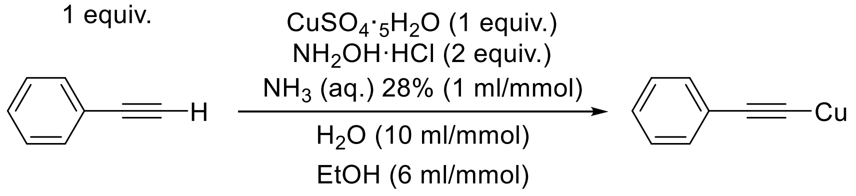

Scheme S7. Synthesis of the phenylacetylene cuprate.

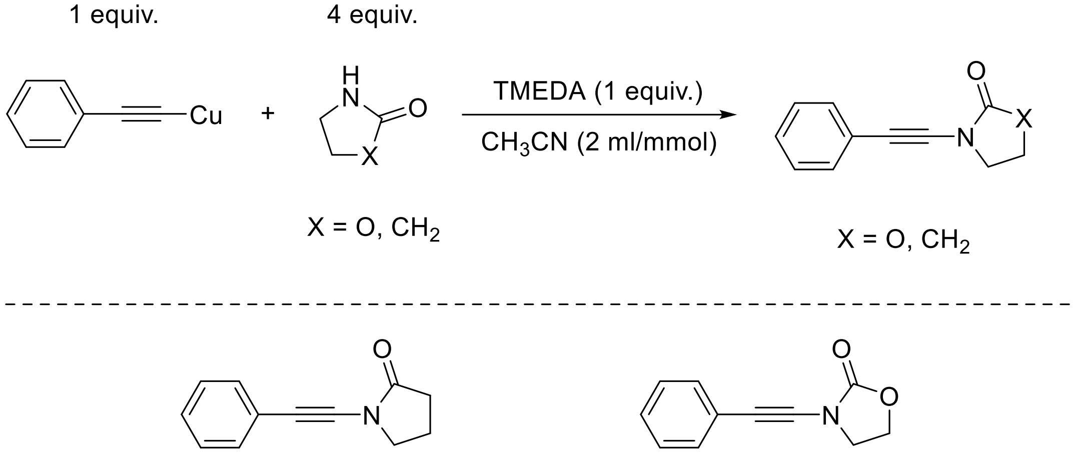

2x **2y**

Scheme S8. Synthesis of the ynamides **2x**, **2y**.

A solution of phenylacetylene (1 equiv.) in ethanol (6 mL/mmol) was added to an ice-cooled mixture of copper sulfate pentahydrate (1 equiv.), 28% aqueous ammonia (1 mL/mmol), water (10 mL/mmol) and hydroxylamine hydrochloride (2 equiv.). The mixture was stirred for 10 min and the precipitate was filtered, washed with water (2 mL/mmol), ethanol (2 mL/mmol) and diethyl ether, and dried *in vacuo* overnight.

In a round-bottom flask was stirred a solution of phenylacetylene cuprate (1 equiv.) and the corresponding amide (2-pyrrolidinone or 2-oxazolidone) (4 equiv.) in acetonitrile (2 mL/mmol). An O_2 balloon was placed through the septum. Then, N,N,N',N'-tetramethylethylenediamine (1 equiv.) was added to the reaction and the mixture was vigorously stirred until completion of the reaction (dark green colour solution). The solvent was evaporated under reduced pressure and the mixture was purified on column chromatography using mixtures of hexanes/AcOEt. The NMR data obtained of products **2** agreed with the ones previously described in the literature.

G) General procedure for the synthesis of alkyne 2z[14].

The NMR data obtained for this product were in agreement with the ones previously described in the literature.

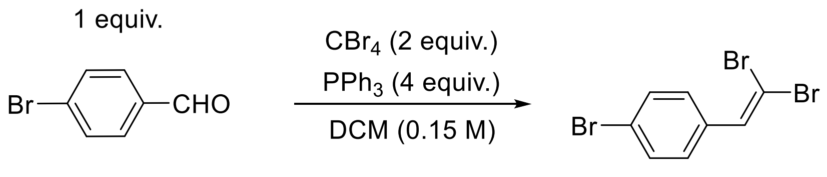

Scheme S9. Synthesis of 1-bromo-4-(2,2-dibromovinyl)benzene.

In a round-bottom flask a solution of triphenylphosphine (4 equiv.), carbon tetrabromide (2 equiv.) in DCM (0.15 M) was stirred under argon atmosphere at 0 °C for 30 min. Bromobenzaldehyde was added in portions over a period of 5 minutes and the mixture was stirred at 0 ºC for 1h. After the completion of the reaction (TLC), the reaction mixture was quenched with water, and the aqueous layer was extracted with DCM. The combined organic layers were dried over $MgSO_4$ and the solvent was removed under reduced pressure. The crude product was purified by flash chromatography using n-pentane/DCM as eluent.[18]

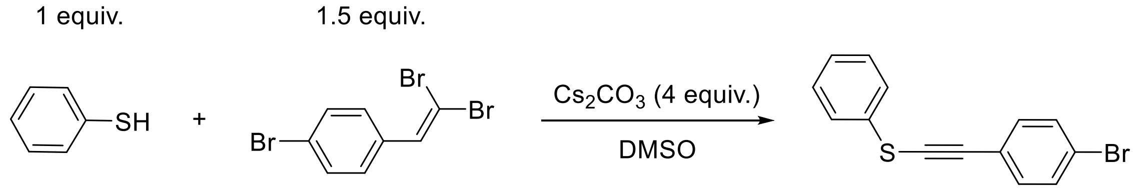

2z

Scheme S10. Synthesis of the thia-alkyne **2z**.

The obtained 1-bromo-4-(2,2-dibromovinyl)benzene was charged in a 15 mL pressure tube with Cs_2CO_3 (4 equiv.), thiophenol (1 equiv.) and DMSO (6 mL/mmol), The tube was capped and heated at 100 °C for 12 h. After the completion of the reaction (TLC), the mixture was cooled down to room temperature, diluted with Et_2O and washed with water. The combined organic layers were dried over $MgSO_4$ and the solvent was removed under reduced pressure. The crude product was purified by flash chromatography using hexanes/AcOEt.

References:

1) Chen, J.; Wu, X.; Zhang, S.; Yan, X.; Wu, X.; Cao, Q.; Xu, H.; Li, X. Commercially available palladium salts as practical and green single-component catalysts in the coordination polymerization of 1-chloro-2-phenylacetylenes in air. *Polym. Chem.* **2022**, *13*, 3870–3875.

2) Chowdhury, R. M.; Wilden, J. D. An improved transition-metal-free synthesis of aryl alkynyl sulfides via substitution of a halide at an *sp*-centre. *Org. Biomol. Chem.* **2015**, *13*, 5859–5861.

3) Mader, S.; Molinari, L.; Rudolph, M.; Rominger, F.; Hashmi, A. S. K. Dual Gold-Catalyzed Head-to-Tail Coupling of Iodoalkynes. *Chem. Eur. J.* **2015**, *21*, 3910–3913.

4) Hazarika, D.; Phukan, P. TsNBr2 promoted decarboxylative bromination of α,β-unsaturated carboxylic acids. *Tetrahedron Lett*. **2018**, *59*, 4593–4596.

5) Rajbongshi, K. K.; Hazarika, D.; Phukan, P. TsNBr 2 mediated oxidative functionalization of alkynes. *Tetrahedron*. **2016**, *72*, 4151–4158.

6) Suzuki, S., Asako, T.; Itami, K.; Yamaguchi, J. Modular synthesis of heptaarylindole. *Org. Biomol. Chem*. **2018**, *16*, 3771–3776.

7) Smela, M. P.; Hoye, T. R. A Traceless Tether Strategy for Achieving Formal Intermolecular Hexadehydro-Diels–Alder Reactions. *Org. Lett*. **2018**, *20*, 5502–5505.

8) Teske, J. A.; Deiters, A. *Org. Lett.* **2008***, 10,* 2195–2198.

9) do Carmo Pinheiro, R.; Back, D. F.; Gilson, Z. Iron(III) Chloride/Dialkyl Diselenides-Promoted Cascade Cyclization of ortho-Diynyl Benzyl Chalcogenides. *Adv. Synth. Catal*. **2019**, *361*, 1866–1873.

10) Starkov, P.; Rota, F.; D'Oyley, J. M.; Sheppard, T. D. Catalytic Electrophilic Halogenation of Silyl-Protected and Terminal Alkynes: Trapping Gold(I) Acetylidesvs. a Brønsted Acid-Promoted Reaction. *Adv. Synth. Catal*. **2012**, *354*, 3217–3224.

11) Gómez-Herrera, A.; Nahra, F.; Brill, M.; Nolan, S. P.; Cazin, C. S. J. Sequential Functionalization of Alkynes and Alkenes Catalyzed by Gold(I) and Palladium (II) N-Heterocyclic Carbene Complexes. *ChemCatChem*. **2016**, *8*, 3381–3388.

12) Petcu, A. S.; Lázaro-Milla, C.; Rodríguez, F. J.; Iriepa, I.; Bautista-Aguilera, Ó. M.; Aragoncillo, C.; Alonso, J. M.; Almendros, P. Straightforward Synthesis of Bis [(trifluoromethyl)sulfonyl] ethylated Isocoumarins from 2-Ethynylbenzoates. *J. Org. Chem*. **2023**, *88*, 7373–7380.

13) Guang, X.; Li, W.-J.; Shuai, M.-S.; Zhang, M.; Fu, X.-Z.; Yang, Y.-Y. Zhou, M.; He, B.; Zhao, Y.-L. Rh(III)-Catalyzed C7-Alkylation of Isatogens with Malonic Acid Diazoesters. *J. Org. Chem*. **2024**, *89*, 2984–2995.

14) Alcaide, B.; Almendros, P.; Lázaro-Milla, C. Regioselective Synthesis of Heteroatom-Functionalized Cyclobutene-triflones and Cyclobutenones. *Adv. Synth. Catal*. **2017**, *359*, 2630–2639.

15) Sato, A. H.; Ohashi, K.; Ito, K.; Iwasawa, T. Regio- and stereoselective synthesis of 1-(1-halovinyl)-1H-indoles from 1-ethynyl-1H-indoles with in situ generated HX. *Tetrahedron Lett*. **2013**, *54*, 2878–2881.

16) Gallego, D.; Brück, A.; Irran, E.; Meier, F.; Kaupp, M.; Driess, M.; Hartwig, J. F. From Bis(silylene) and Bis(germylene) Pincer-Type Nickel (II) Complexes to Isolable Intermediates of the Nickel-Catalyzed Sonogashira Cross-Coupling Reaction. *J. Am. Chem. Soc*. **2013**, *135*, 15617–15626.

17) Jouvin, K.; Heimburger, J.; Evano, G. Click-alkynylation of N- and P-nucleophiles by oxidative cross-coupling with alkynylcopper reagents: a general synthesis of ynamides and alkynylphosphonates. *Chem. Sci.* **2012**, *3*, 756–760.
18) Hack, D.; Chauhan, P.; Deckers, K.; Hermann, G. N.; Mertens, L.; Raabe, G.; Enders, D. Combining Silver Catalysis and Organocatalysis: A Sequential Michael Addition/Hydroalkoxylation One-Pot Approach to Annulated Coumarins. *Org. Lett.* **2014**, *16*, 5188–5191.

General procedure for the synthesis of cyclobutenes 3 and 7a

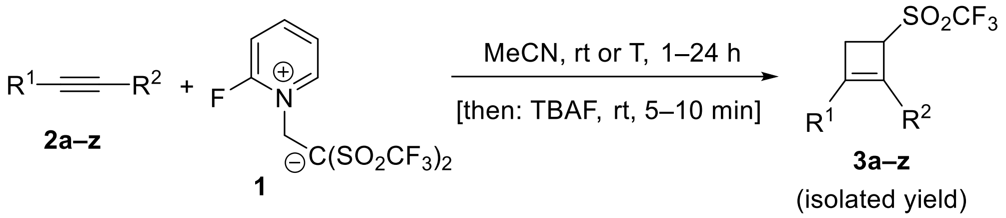

Scheme S11. General synthesis of cyclobutenes **3**.

The corresponding alkyne **2** (1 equiv.), betaine **1** (1.05 equiv.) and acetonitrile (8 ml/mmol) were added to an oven-dried round bottom flask. The reaction was stirred at room temperature until disappearance of the starting alkyne (TLC), before TBAF (2 equiv. ; 1M solution in THF) was slowly added. Then, the reaction was stirred at rt for 5-10 minutes. Next, the crude mixture was quenched with 5 mL of water and extracted with AcOEt (3 x 5mL). The organic phases were combined and dried with $MgSO_4$. After filtration and evaporation of the solvent under reduced pressure, the mixture was purified on column chromatography using mixtures of hexanes and AcOEt.

Compound 3a

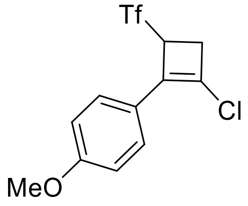

Following the general procedure and starting from 8 mg of alkyne **2a,** 13 mg (79%) of compound **3a** were obtained as a colorless solid, after purification on column chromatography using hexanes/AcOEt (20:1) as eluent; m.p. 87-89 °C; ^{1}H NMR ($CDCl_3$, 300 MHz, 25 °C) δ 7.72 (2H, d, *J* = 8.9 Hz, $H_{Ar\text{-}PMP}$), 6.95 (2H, d, *J* = 9.0 H, $H_{Ar\text{-}PMP}$), 5.06–4.55 (1H, m, C*H*Tf), 3.86 (3H, s, OMe), 3.22

(2H, m, CH_2); $^{13}C\ \{^1H\}$ NMR ($CDCl_3$, 75 MHz, 25 °C) δ 160.5 (1C, C_{Ar}), 132.9 (1C, Ar-*C*=C-Cl), 128.4 (2CH, CH_{Ar}), 124.7 (1C, Ar-C=*C*-Cl), 122.4 (1C, C_{Ar}), 119.9 (1C, q, *J* = 327 Hz, CF_3), 114.1 (2C, CH_{Ar}), 56.0 (*CH*Tf), 55.4 (OMe), 36.1 (CH_2); ^{19}F NMR ($CDCl_3$, 282 MHz, 25 °C) δ –75.97 (3F, s, CF_3); HRMS (ESI-TOF) m/z: $[M-H]^-$ Calcd. for $C_{12}H_9ClF_3O_3S$ 324.9919; Found 324.9914.

Compound 3b

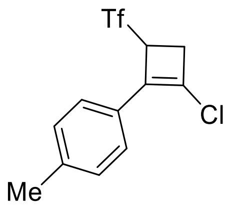

Following the general procedure and starting from 14 mg of alkyne **2b**, 19.5 mg (92%) of compound **3b** were obtained as a colorless solid, after purification on column chromatography using hexanes/AcOEt (100:1) as eluent; m.p. 90-92 °C; ^1H NMR ($CDCl_3$, 300 MHz, 25 °C) δ 7.64 (2H, d, *J* = 8.2 Hz, H_{Ar}), 7.23 (2H, d, *J* = 8.2 Hz, H_{Ar}), 4.85 (1H, dd, *J* = 4.1, 2.5 Hz, C*H*Tf), 3.22 (1H, dd, *J* = 14.2, 2.5 Hz, CH_2), 3.18 (1H, dd, *J* = 14.2, 4.1 Hz, CH_2), 2.38 (3H, s, Me); $^{13}C\ \{^1H\}$ NMR ($CDCl_3$, 75 MHz, 25 °C) δ 140.0 (C_{Ar}) 133.4 (Ar-*C*=C-Cl), 129.3 (2C, CH_{Ar}), 126.8 (2C, CH_{Ar}), 126.4 (C_{Ar}), 119.8 (q, J = 328.92 Hz, SO_2CF_3), 56.0 (*CH*Tf), 36.2 (CH_2), 21.5 (Me); ^{19}F NMR ($CDCl_3$, 282 MHz, 25 °C) δ –76.0 (3F, s, CF_3); HRMS (ESI-TOF) m/z: $[M-H]^-$ Calcd. for $C_{12}H_9ClF_3O_2S$ 308.9969; Found 308.9973.

Compound 3c

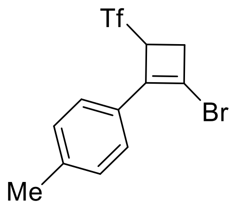

Following the general procedure and starting from 19 mg of alkyne **3c**, 21 mg (61%) of compound **3c** were obtained as a colorless solid, after purification on column chromatography using hexanes/AcOEt (50:1) as eluent; m.p. 91-93 °C; ^1H NMR ($CDCl_3$, 300 MHz, 25 °C) δ 7.69 (2H, d, *J* = 8.2 Hz, H_{Ar}), 7.23 (2H, d, *J* = 8.2 Hz, H_{Ar}), 4.94 (1H, dd, *J* = 4.2, 2.1 Hz, CHTf), 3.31 (1H, d, *J* = 14.0 Hz, CH_2), 3.24 (1H, dd, *J* = 14.0, 4.2 Hz, CH_2), 2.38 (3H s, Me); $^{13}C\ \{^1H\}$ NMR ($CDCl_3$, 75 MHz, 25 °C) δ 140.3 (C_{Ar}), 137.1 (Ar-*C*=C-Br), 129.4 (2C, CH_{Ar}), 127.2 (C_{Ar}), 126.6 (2C, CH_{Ar}), 119.9 (q, J = 329.0 Hz, SO_2CF_3), 115.2 (Ar-C=*C*-Br), 58.3 (*CH*Tf), 37.0 (CH_2), 21.7 (Me); ^{19}F NMR ($CDCl_3$, 282 MHz, 25 °C) δ –76.0 (3F, s, CF_3); HRMS (ESI-TOF) m/z: $[M-H]^-$ Calcd. for $C_{12}H_9BrF_3O_2S$ 352.9464; Found 352.9464.

Compound 3d

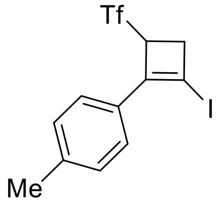

Following the general procedure and starting from 106 mg of alkyne **2d**, 108 mg (73%) of compound **3d** were obtained as a yellow solid, after purification on column chromatography using hexanes/AcOEt (100:1) as eluent; m.p. 92-94 °C; ^{1}H NMR ($CDCl_3$, 300 MHz, 25 °C) δ 7.72 (2H, d, *J* = 8.2 Hz, H_{Ar}), 7.24 (2H, d, *J* = 8.2 Hz, H_{Ar}), 5.09 (1H, dd, *J* = 4.4, 2.0 Hz, C*H*Tf), 3.31 (1H, d, *J* = 14.2 Hz, CH_2), 3.22 (1H, dd, *J* = 14.2, 4.4 Hz, CH_2), 2.37 (3H, s, Me); ^{13}C {^{1}H} NMR ($CDCl_3$, 75 MHz, 25 °C) δ 143.4 (C_{Ar}), 140.5 (Ar-*C*=C-I), 129.3 (2C, CH_{Ar}), 127.8 (C_{Ar}), 125.9 (2C, CH_{Ar}), 119.8 (q, J = 329.13 Hz, SO_2CF_3), 87.9 (Ar-C=*C*-I), 61.4 (*CH*Tf), 38.0 (CH_2), 21.7 (Me); ^{19}F NMR ($CDCl_3$, 282 MHz, 25 °C) δ –75.9 (3F, s, CF_3); IR (cm^{-1}) ν 2950 (CH_3), 1390 (*SO_2*CF_3), 1200 (SO_2*CF_3*), 1100 (*SO_2*CF_3), 800 (*p*-Ar), 700 (CH_2), 650; HRMS (ESI-TOF) m/z: [M+Na]$^+$ Calcd. for $C_{12}H_{10}F_3IO_2SNa$ 424.9290; Found 424.9298.

Compound 3e

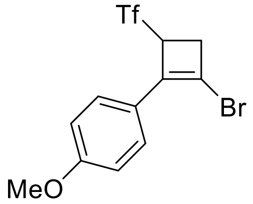

Following the general procedure and starting from 16 mg of alkyne **2e**, 23 mg (82 %) of compound **3e** were obtained as a colorless solid, after purification on column chromatography using hexanes/AcOEt (20:1) as eluent; m.p. 83-85 °C; ^{1}H NMR ($CDCl_3$, 300 MHz, 25 °C) δ 7.77 (2H, d, *J* = 8.9 Hz, $H_{Ar\text{-}PMP}$), 6.96 (2H, d, *J* = 8.9 Hz, $H_{Ar\text{-}PMP}$), 5.16–4.62 (1H, m, C*H*Tf), 3.86 (3H, s, OMe), 3.40–2.94 (2H, m, CH_2); ^{13}C {^{1}H} NMR ($CDCl_3$, 75 MHz, 25 °C) δ 160.8 (1C, C_{Ar}), 136.3 (1C, Ar-*C*=C-Br), 128.1 (2C, CH_{Ar}), 122.6 (1C, C_{Ar}), 119. 8 (1C, q, J = 320 Hz, CF_3), 113.9 (2C, CH_{Ar}), 113.2 (1C, Ar-C=*C*-Br), 58.2 (*CH*Tf), 55.4 (OMe), 36.7 (CH_2); ^{19}F NMR ($CDCl_3$, 282 MHz, 25 °C) δ –76.43 (3F, s, CF_3); HRMS (ESI-TOF) m/z: [M-H]$^-$ Calcd for $C_{12}H_9BrF_3O_3S$ 368.9413; Found 368.9422.

Compound 3f

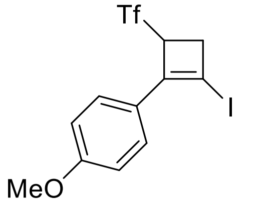

Following the general procedure and starting from 13 mg of alkyne **2f**, 13 mg (62 %) of compound **3f** were obtained as a yellow solid, after purification on column chromatography using hexanes/AcOEt (11:1) as eluent; m.p. 81-83 °C; ^{1}H NMR ($CDCl_3$, 300 MHz, 25 °C) δ 7.70 (2H, d, *J* = 8.9 Hz, H_{Ar-PMP}), 6.87 (2H, d, *J* = 8.9 Hz, H_{Ar-PMP}), 4.99 (1H, dd, *J* = 4.5, 2.0 Hz, C*H*Tf), 3.76 (3H, s, OMe), 3.40–2.64 (2H, m, CH_2); ^{13}C {^{1}H} NMR ($CDCl_3$, 75 MHz, 25 °C) δ 160.6 (1C, C_{Ar}), 142.4 (1C, Ar-*C*=C-I), 127.5 (2C, CH_{Ar}), 123.3 (1C, C_{Ar}), 119.8 (1C, q, J = 321.4 Hz, CF_3), 113.8 (2C, CH_{Ar}), 85.5 (1C, Ar-C=*C*-I), 61.1 (*CH*Tf), 35.2 (OMe), 37.6 (CH_2); ^{19}F NMR ($CDCl_3$, 282 MHz, 25 °C) δ –75.92 (3F, s, CF_3); HRMS (ESI-TOF) m/z: $[M+NH_4]^+$ Calcd for $C_{12}H_{14}F_3IO_3SN$ 435.9686; Found, 435.9699.

Compound 3g

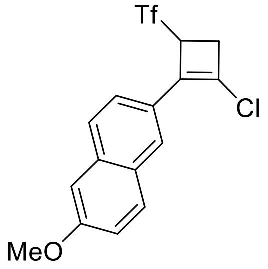

Following the general procedure and starting from 15.6 mg of alkyne **2g**, 11.4 mg (63%) of compound **3g** were obtained as a colorless solid, after purification on column chromatography using hexanes/AcOEt (30:1) as eluent; m.p. 112-114 °C; ^{1}H NMR ($CDCl_3$, 300 MHz, 25 °C) δ 8.11 (1H, s, H_{Ar}), 7.86 (1H, dd, *J* = 8.8, 1.8 Hz, H_{Ar}), 7.77 (2H, t, *J* = 9.7 Hz, H_{Ar}), 7.18 (1H, dd, *J* = 8.8, 2.5 Hz, H_{Ar}), 7.13 (1H, d, *J* = 2.5 Hz, H_{Ar}), 4.94 (1H, dd, *J* = 4.2, 2.2 Hz, C*H*Tf), 3.94 (3H, s, OMe), 3.30 (1H, d, *J* = 13.3 Hz, CH_2), 3.26 (1H, d, *J* = 4.2 Hz, CH_2); ^{13}C {^{1}H} NMR ($CDCl_3$, 175 MHz, 25 °C) δ 159.0 (C_{Ar}), 135.1 (C_{Ar}), 133.7 (Ar-*C*=C-Cl), 130.4 (CH_{Ar}), 128.4 (C_{Ar}), 127.3 (CH_{Ar}), 127.0 (CH_{Ar}), 126.6 (Ar-C=*C*-Cl), 125.0 (C_{Ar}), 124.2 (CH_{Ar}), 120.0 (q, J = 329.0 Hz, SO_2CF_3), 119.8 (CH_{Ar}), 105.9 (CH_{Ar}), 56.2 (*CH*Tf), 55.5 (OMe), 36.4 (CH_2); ^{19}F NMR ($CDCl_3$, 282 MHz, 25 °C) δ –75.9 (3F, s, CF_3); FTIR (cm^{-1}) ν = 2950 (CH_3), 1390 (*SO_2*CF_3), 1300 (CH_3), SO_2*CF_3* (1200), 1100 (*SO_2*CF_3), 1000 (C-Cl), 900 (Ar), 850 (o-Ar), 700 (CH_2), 600; HRMS (ESI-TOF) m/z: $[M-H]^-$ Calcd. for $C_{16}H_{11}ClF_3O_3S$ 375.0075; Found 375.0077.

Compound 3g-d$_2$

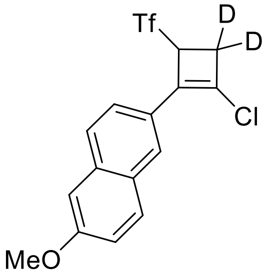

Following the general procedure and starting from 14 mg of alkyne **2g**, 10.6 mg (48%) of compound **3g-d$_2$** were obtained as a yellow solid, after purification on column chromatography using hexanes/AcOEt (200:1) as eluent; m.p. 111-113 °C; ^{1}H NMR (CDCl$_3$, 300 MHz, 25 °C) δ 8.11 (1H, d, J = 1.8 Hz, H$_{Ar}$), 7.86 (1H, dd, J = 8.7, 1.8 Hz, H$_{Ar}$), 7.77 (2H, t, J = 9.6 Hz, H$_{Ar}$), 7.18 (1H, dd, J = 8.7, 2.5 Hz, H$_{Ar}$), 7.13 (1H, d, J = 2.5 Hz, H$_{Ar}$), 4.93 (1H, s, C*H*Tf), 3.94 (3H, s, OMe); ^{13}C {^{1}H} NMR (CDCl$_3$, 175 MHz, 25 °C) δ 159.0 (C$_{Ar}$, C-OMe), 135.0 (C$_{Ar}$), 133.8 (Ar-*C*=C-Cl), 130.4 (CH$_{Ar}$), 128.4 (C$_{Ar}$), 127.3 (CH$_{Ar}$), 127.0 (CH$_{Ar}$), 126.5 (Ar-C=*C*-Cl), 125.0 (C$_{Ar}$), 124.2 (CH$_{Ar}$), 120.0 (q, J = 328.43 Hz, SO$_2$CF$_3$), 119.8 (CH$_{Ar}$), 105.9 (CH$_{Ar}$), 56.0 (*CH*Tf), 55.5 (OMe); ^{19}F NMR (CDCl$_3$, 282 MHz, 25 °C) δ –75.91 (3F, s, CF$_3$); ^{2}H NMR (CDCl$_3$, 107 MHz, 25 °C) δ 3.25 (2D, s, CD$_2$); IR (cm^{-1}) ν = 2950 (CH$_3$), 1390 (*SO$_2$*CF$_3$), 1300 (CH$_3$), SO$_2$*CF$_3$* (1200), 1100 (*SO$_2$*CF$_3$), 1000 (C-Cl), 900 (Ar), 700 (CH$_2$), 600; HRMS (ESI-TOF) m/z: [M-H]$^-$ Calcd. for $C_{16}H_9D_2ClF_3O_3S$ 377.0201; Found 377.0208.

Compound 3h

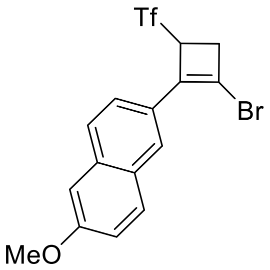

Following the general procedure and starting from 13 mg of alkyne **2h**, 13.2 mg (70%) of compound **3h** were obtained as a yellow solid, after purification on column chromatography using hexanes/AcOEt (30:1) as eluent; m.p. 107-109 °C; ^{1}H NMR (CDCl$_3$, 300 MHz, 25 °C) δ 8.16 (1H, s, H$_{Ar}$), 7.91 (1H, dd, *J* = 8.6, 2.0 Hz, H$_{Ar}$), 7.78 (2H, dd, *J* = 11.2, 8.9 Hz,), 7.18 (1H, dd, *J* = 8.9, 2.5 Hz, H$_{Ar}$), 7.13 (1H, d, *J* = 2.5 Hz, H$_{Ar}$), 5.04 (1H, dd, *J* = 4.0, 2.0 Hz, C*H*Tf), 3.94 (3H, s, OMe), 3.36 (1H, d, *J* = 14.2 Hz, CH$_2$), 3.29 (1H, dd, *J* = 14.2, 4.0 Hz, CH$_2$); ^{13}C {^{1}H} NMR (CDCl$_3$, 175 MHz, 25 °C) δ 159.0 (C$_{Ar}$), 137.3 (Ar-*C*=C-Br), 135.2 (C$_{Ar}$), 130.4 (CH$_{Ar}$), 128.4 (C$_{Ar}$), 127.2 (CH$_{Ar}$), 126.8 (CH$_{Ar}$), 125.3 (C$_{Ar}$), 123.7 (CH$_{Ar}$), 119.9 (q, J = 329.0 Hz, SO$_2$CF$_3$), 119.8 (CH$_{Ar}$), 115.3 (Ar-C=*C*-Br),

106.0 (CH_{Ar}), 58.5 (*CH*Tf), 55.5 (OMe), 37.1 (CH_2); ^{19}F NMR ($CDCl_3$, 282 MHz, 25 °C) δ –75.9 (3F, s, CF_3); HRMS (ESI-TOF) m/z: $[M-H]^-$ Calcd. for $C_{16}H_{11}BrF_3O_3S$ 418.9570; Found 418.9571.

Compound 3h-d₂

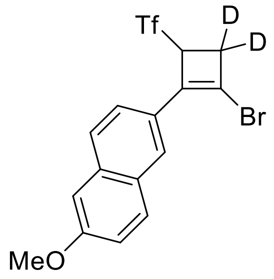

Following the general procedure and starting from 15.5 mg of alkyne **2h**, 9.4 mg (50%) of compound **3h-d$_2$** were obtained as a colorless solid, after purification on column chromatography using hexanes/AcOEt (200:1) as eluent; m.p. 98-100 °C; ^1H NMR ($CDCl_3$, 300 MHz, 25 °C) δ 8.16 (1H, d, J = 1.8 Hz, H_{Ar}), 7.91 (1H, dd, J = 8.6, 1.8 Hz, H_{Ar}), 7.80 (1H d, J = 8.9 Hz, H_{Ar}), 7.76 (1H, d, J = 8.6 Hz, H_{Ar}), 7.18 (1H, dd, J = 8.9, 2.6 Hz, H_{Ar}), 7.13 (1H, d, J = 2.6 Hz, H_{Ar}), 5.03 (1H, s, C*H*Tf), 3.95 (3H, s, OMe); ^{13}C {^1H} NMR ($CDCl_3$, 75 MHz, 25 °C) δ 159.0 (C_{Ar}, C-OMe), 135.2 (C_{Ar}), 132.7 (Ar-*C*=C-Br), 130.4 (CH_{Ar}), 128.4 (C_{Ar}), 127.2 (CH_{Ar}), 126.8 (CH_{Ar}), 125.3 (C_{Ar}), 123.7 (CH_{Ar}), 119.8 (CH_{Ar}), 118.6 (Ar-C=*C*-Br), 106.0 (CH_{Ar}), 58.3 (*CH*Tf), 55.5 (OMe); ^{19}F NMR ($CDCl_3$, 282 MHz, 25 °C) δ –75.9 (3F, s, CF_3); HRMS (ESI-TOF) m/z: $[M-H]^-$ Calcd. for $C_{16}H_9D_2BrF_3O_3S$ 420.9695; Found 420.9694.

Compound 3i

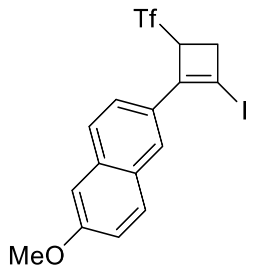

Following the general procedure and starting from 16 mg of alkyne **2i**, 20 mg (76 %) of compound **3i** were obtained as a yellow solid, after purification on column chromatography using hexanes/AcOEt (6:1) as eluent; m.p. 93-95 °C; ^1H NMR ($CDCl_3$, 300 MHz, 25 °C) δ 8.20 (1H, br s, H_{Ar}), 7.96 (1H, dd, J = 8.6, 1.9 Hz, H_{Ar}), 7.88–7.58 (2H, m, H_{Ar}), 7.20 (1H, dd, J = 8.9, 2.5 Hz, H_{Ar}), 7.14 (1H, m, H_{Ar}), 5.24–5.10 (1H, m, *CH*Tf), 3.96 (3H, s, OMe), 3.55–2.64 (2H, m, CH_2); ^{13}C {^1H} NMR ($CDCl_3$, 75 MHz, 25 °C) δ 158.8 (1C, C_{Ar}), 143.0 (1C, Ar-*C*=C-I), 135.2 (1C, C_{Ar}), 130.0 (1C, CH_{Ar}), 128,1 (1C, C_{Ar}), 126.8 (1C, CH_{Ar}), 126.2 (1C, CH_{Ar}), 125.8 (1C, C_{Ar}), 122.7 (1C, CH_{Ar}), 120.9 (1C, q, J

= 270 MHz, CF_3), 119.6 (1C, CH_{Ar}), 105.8 (1C, CH_{Ar}), 87.8 (1C, Ar-C=*C*-I), 61.1 (*CH*Tf), 55.3 (OMe), 37.7 (CH_2); HRMS (ESI-TOF) m/z: $[M+NH_4]^+$ Calcd. for $C_{16}H_{16}F_3IO_3SN$ 485.9840; Found 485.9842.

Compound 3j

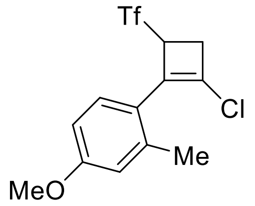

Following the general procedure and starting from 10.7 mg of alkyne **2j**, 13.1 mg (91%) of compound **3j** were obtained as a colorless solid, after purification on column chromatography using hexanes/AcOEt (100:1) as eluent; m.p. 73-75 °C; ^{1}H NMR ($CDCl_3$, 300 MHz, 25 °C) δ 7.29 (1H, d, *J* = 6.7 Hz, H_{Ar}), 6.77 (1H, dd, *J* = 5.6, 1.7 Hz, H_{Ar}), 6.76 (1H, s, H_{Ar}), 4.87 (1H, dd, *J* = 3.8, 2.2 Hz,), 3.82 (3H, s, OMe), 3.23 (1H, d, *J* = 3.8 Hz,), 3.19 (1H, d, *J* = 14.0 Hz,), 2.42 (3H, s, Me); ^{13}C {^{1}H} NMR ($CDCl_3$, 75 MHz, 25 °C) δ 160.6 (C_{Ar}), 138.7 (C_{Ar}), 135.1 (Ar-*C*=C-Cl), 130.2 (CH_{Ar}), 129.5 (Ar-C=*C*-Cl), 121.5 (C_{Ar}), 119.7 (q, *J* = 328.6 Hz, SO_2CF_3), 116.3 (CH_{Ar}), 111.5 (CH_{Ar}), 57.6 (*CH*Tf), 55.4 (OMe), 35.5 (CH_2), 20.8 (Me); ^{19}F NMR ($CDCl_3$, 282 MHz, 25 °C) δ –76.6 (3F, s, CF_3); HRMS (ESI-TOF) m/z: $[M-H]^-$ Calcd. for $C_{13}H_{11}ClF_3O_3S$ 339.0075; Found: 339.0079.

Compound 3j-d$_2$

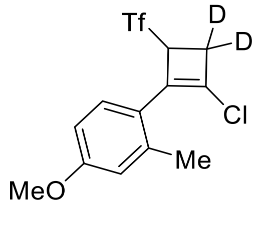

Following the general procedure and starting from 15.4 mg of alkyne **2j**, 11.7 mg (88%) of compound **3j-d$_2$** were obtained as a colorless solid, after purification on column chromatography using hexanes/AcOEt (200:1) as eluent; m.p. 65-67 °C; ^{1}H NMR ($CDCl_3$, 300 MHz, 25 °C) δ 7.30–7.27 (1H, m, H_{Ar}), 6.79–6.76 (2H, m, H_{Ar}), 4.86 (1H, s, C*H*Tf) 3.82 (3H, s, OMe), 2.42 (3H, s, Me); ^{13}C {^{1}H} NMR ($CDCl_3$, 75 MHz, 25 °C) δ 160.6 (C_{Ar}, C-OMe), 138.7 (C_{Ar}), 135.2 (Ar-*C*=C-Cl), 130.2 (CH_{Ar}), 129.3 (Ar-C=*C*-Cl), 121.5 (C_{Ar}), 119.7 (q, J = 328.43 Hz, SO_2CF_3), 116.3 (CH_{Ar}), 111.5 (CH_{Ar}), 57.5 (*CH*Tf), 55.4 (OMe), 20.8 (Me); ^{19}F NMR ($CDCl_3$, 282 MHz, 25 °C) δ –76.6 (3F, s, CF_3); HRMS (ESI-TOF) m/z: $[M-H]^-$ Calcd. for $C_{13}H_9D_2ClF_3O_3S$ 341.0201; Found 341.0199.

Compound 3k

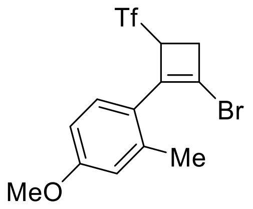

Following the general procedure and starting from 14.5 mg of alkyne **2k**, 18 mg (81%) of compound **3k** were obtained as a colorless solid, after purification on column chromatography using hexanes/AcOEt (100:1) as eluent; m.p. 78-80 °C; ^{1}H NMR ($CDCl_3$, 300 MHz, 25 °C) δ 7.31 (1H, dt, *J* = 8.4, 1.1 Hz, H_{Ar}), 6.79 (1H, d, *J* = 2.7 Hz, H_{Ar}), 6.76 (1H, s, H_{Ar}), 4.95 (1H, dd, *J* = 4.1, 2.0 Hz, C*H*Tf), 3.82 (3H, s, OMe), 3.32 (1H, dd, *J* = 14.1, 2.0 Hz, CH_2), 3.25 (1H, dd, *J* = 14.1, 4.1 Hz, CH_2), 2.43 (3H, s, Me); ^{13}C {^{1}H} NMR ($CDCl_3$, 75 MHz, 25 °C) δ 160.6 (C_{Ar}), 139.3 (Ar-*C*=C-Br), 138.5 (C_{Ar}), 130.1 (CH_{Ar}), 122.1 (C_{Ar}), 119.6 (q, J = 328.5 Hz, SO_2CF_3), 119.1 (Ar-C=*C*-Br), 116.3 (CH_{Ar}), 111.5 (CH_{Ar}), 60.1 (*CH*Tf), 55.4 (OMe), 36.3 (CH_2), 20.9 (Me); ^{19}F NMR ($CDCl_3$, 282 MHz, 25 °C) δ –76.6 (3F, s, CF_3); HRMS (ESI-TOF) m/z: $[M-H]^-$ Calcd. for $C_{13}H_{11}BrF_3O_3S$ 382.9570; Found 382.9568.

Compound 3l

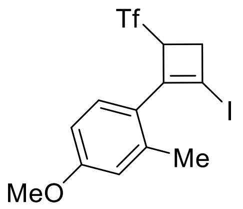

Following the general procedure and starting from 13 mg of alkyne **2l**, 12.6 mg (61%) of compound **3l** were obtained as a yellow solid, after purification on column chromatography using hexanes/AcOEt (70:1) as eluent; m.p. 83-85 °C; ^{1}H NMR ($CDCl_3$, 300 MHz, 25 °C) δ 7.31 (1H, d, *J* = 8.2 Hz, H_{Ar}), 6.79 (2H, d, *J* = 2.7 Hz, H_{Ar}), 6.76 (1H, s, H_{Ar}) 5.07 (1H, dd, *J* = 4.1, 2.1 Hz, C*H*Tf), 3.82 (3H, s, OMe), 3.32 (1H, dd, *J* = 14.1, 2.1 Hz, CH_2), 3.25 (1H, dd, *J* = 14.1, 4.1 Hz, CH_2), 2.43 (s, 3H, Me); ^{13}C {^{1}H} NMR ($CDCl_3$, 75 MHz, 25 °C) δ 160.6 (C_{Ar}, C-OMe), 146.7 (Ar-*C*=C-I), 138.1 (C_{Ar}), 129.8 (CH_{Ar}), 123.3 (C_{Ar}), 119.6 (q, J = 328.5 Hz, SO_2CF_3), 116.3 (CH_{Ar}), 111.5 (CH_{Ar}), 93.8 (Ar-C=*C*-I), 63.3 (*CH*Tf), 55.4 (OMe), 37.4 (CH_2), 21.0 (Me); ^{19}F NMR ($CDCl_3$, 282 MHz, 25 °C) δ –76.6 (3F, s, CF_3); HRMS (ESI-TOF) m/z: $[M+H]^+$ Calcd. for $C_{13}H_{13}F_3IO_3S$ 432.9577; Found 432.9580.

Compound 3m

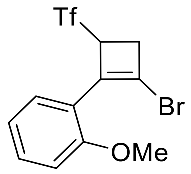

Following the general procedure and starting from 21 mg of alkyne **2m**, 22.4 mg (60%) of compound **3m** were obtained as a colorless solid, after purification on column chromatography using hexanes/AcOEt (50:1) as eluent; m.p. 110-112 °C; ^{1}H NMR ($CDCl_3$, 300 MHz, 25 °C) δ 7.65 (1H, dd, *J* = 7.5, 1.7 Hz, H_{Ar}), 7.37 (1H, ddd, *J* = 8.6, 7.5, 1.7 Hz, H_{Ar}), 7.01 (1H, td, *J* = 7.5, 1.1 Hz, H_{Ar}), 6.91 (1H, dd, *J* = 8.6, 1.1 Hz, H_{Ar}), 5.23 (1H, dd, *J* = 4.2, 2.2 Hz, C*H*Tf), 3.86 (3H, s, OMe), 3.37 (1H, dd, *J* = 14.2, 2.2 Hz, CH_2), 3.30 (1H, dd, *J* = 14.2, 4.2 Hz, CH_2); ^{13}C {^{1}H} NMR ($CDCl_3$, 75 MHz, 25 °C) δ 157.3 (C_{Ar}), 136.7 (Ar-*C*=C-Br), 131.3 (CH_{Ar}), 128.9 (CH_{Ar}), 120.8 (CH_{Ar}), 119.7 (q, J = 328.1 Hz, SO_2CF_3), 118.8 (C_{Ar}), 118.2 (Ar-C=*C*-Br), 111.1 (CH_{Ar}), 60.2 (*CH*Tf), 55.4 (OMe), 37.6 (CH_2); ^{19}F NMR ($CDCl_3$, 282 MHz, 25 °C) δ –77.2 (3F, s, CF_3); HRMS (ESI-TOF) m/z: $[M-H]^-$ Calcd. for $C_{12}H_9BrF_3O_3S$, 368.9413; Found, 368.9409.

Compound 3n

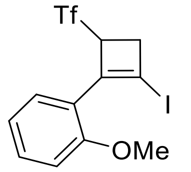

Following the general procedure and starting from 16.3 mg of alkyne **2n**, 14 mg (53%) of compound **3n** were obtained as a colorless solid, after purification on column chromatography using hexanes/AcOEt (50:1) as eluent; m.p. 103-105 °C; ^{1}H NMR ($CDCl_3$, 300 MHz, 25 °C) δ 7.64 (1H, dd, *J* = 7.6, 1.7 Hz, H_{Ar}), 7.39 (1H, ddd, *J* = 8.4, 7.6, 1.7 Hz, H_{Ar}), 7.02 (1H, td, *J* = 7.6, 1.0 Hz, H_{Ar}), 6.91 (1H, dd, *J* = 8.4, 1.0 Hz, H_{Ar}), 5.37 (1H, dd, *J* = 4.3, 2.2 Hz, C*H*Tf), 3.86 (3H, s, OMe), 3.37 (1H, dd, *J* = 14.2, 2.2 Hz, CH_2), 3.29 (1H, dd, *J* = 14.2, 4.3 Hz, CH_2); ^{13}C {^{1}H} NMR ($CDCl_3$, 75 MHz, 25 °C) δ 157.2 (C_{Ar}), 143.7 (Ar-*C*=C-I), 131.4 (CH_{Ar}), 128.1 (CH_{Ar}), 120.7 (CH_{Ar}), 119.6 (q, J = 328.4 Hz, SO_2CF_3), 119.6 (C_{Ar}), 111.1 (CH_{Ar}), 91.9 (Ar-C=*C*-I), 63.0 (*CH*Tf), 55.4 (OMe), 38.6 (CH_2); ^{19}F NMR ($CDCl_3$, 282 MHz, 25 °C) δ –77.2 (3F, s, CF_3); HRMS (ESI-TOF) m/z: $[M-H]^-$ Calcd. For $C_{12}H_9F_3IO_3S$, 416.9275; Found, 416.9272.

Compound 3o

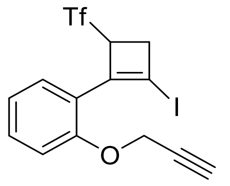

Following the general procedure and starting from 31 mg (0.087 mmol) of alkyne **2o**, 24 mg (61%) of compound **3o** were obtained as a colorless solid, after purification on column chromatography using hexanes/AcOEt (98:2 → 95:5) as eluent; m.p. 103-105 °C; ^{1}H NMR (CDCl$_3$, 700 MHz, 25 °C) δ 7.67 (d, 1H, *J* = 7.6 Hz, CH^{Ar}), 7.39 (t, 1H, *J* = 7.9 Hz, CH^{Ar}), 7.07 (t, 1H, *J* = 7.5 Hz, CH^{Ar}), 7.01 (d, 1H, *J* = 8.4 Hz, CH^{Ar}), 5.42 (d, 1H, *J* = 3.7 Hz, CH), 4.72 (s, 2H, OCH$_2$), 3.35 (d, 1H, *J* = 14.1 Hz, C*H*H), 3.30 (dd, 1H, *J* = 14.2, 4.3 Hz, CH*H*), 2.58 (s, 1H, C≡CH); ^{13}C {^{1}H} NMR (CDCl$_3$, 175 MHz, 25 °C) δ 155.2 ($C^{Ar\text{-}q}$), 142.9 (*C*=C), 131.1 (CH^{Ar}), 128.1 (CH^{Ar}), 122.3 (CH^{Ar}), 120.1 ($C^{Ar\text{-}q}$), 119.5 (q, J_{CF} = 328.4 Hz, CF$_3$), 112.1 (CH^{Ar}), 92.3 (C=*C*Tf), 77.8 (*C*≡CH), 76.0 (C≡*C*H), 62.8 (CH), 56.3 (OCH$_2$), 38.6 (CH$_2$); ^{19}F NMR (CDCl$_3$, 282 MHz, 25 °C) δ –76.9 (s, 3F, CF$_3$); IR (cm^{-1}) ν 1389, 1205 (O=S=O), 1206 (C–F); HRMS (ESI-TOF) m/z: [M + NH$_4$]$^+$ Calcd. for $C_{14}H_{14}F_3INO_3S$ 459.9686; Found 459.9685.

Compound 3p

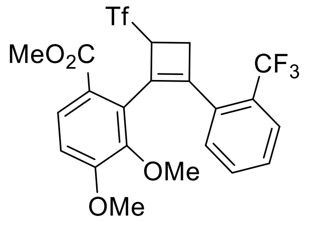

Following the general procedure and starting from 5 mg of alkyne **2p**, 6 mg (58%) of compound **3p** were obtained as a thick yellow oil, after purification on column chromatography using hexanes/AcOEt (3:1) as eluent; ^{1}H NMR (CDCl$_3$, 300 MHz, 25 °C) δ 7.71 (1H, dd, *J* = 6.9, 2.3 Hz, H$_{Ar}$), 7.52 (1H, s, H$_{Ar}$), 7.40 (2H, qd, *J* = 7.8, 3.5 Hz, H$_{Ar}$), 7.27 (1H, dd, *J* = 5.8, 3.5 Hz, H$_{Ar}$), 6.68 (1H, s, H$_{Ar}$), 5.31 (1H, d, *J* = 4.5 Hz, H$_{Ar}$), 3.93 (3H, s, OMe), 3.92 (3H, s, OMe), 3.68 (3H, s, COOMe), 3.54 (1H, d, *J* = 14.2 Hz, CH$_2$), 3.32 (1H, dd, *J* = 14.2, 4.5 Hz, CH$_2$); ^{13}C {^{1}H} NMR (CDCl$_3$, 75 MHz, 25 °C) δ 166.5 (*C*OOMe), 152.3 (C_{Ar}-OMe), 149.0 (C_{Ar}-OMe), 144.6 (C_{Ar}-COOMe), 137.1 (C$_{Ar}$), 132.0 (C$_{Ar}$), 131.9 (CH$_{Ar}$), 131.1 (CH$_{Ar}$), 129.0 (CH$_{Ar}$), 128.1 (d, ^2J = 31.37 Hz, C$_{Ar}$), 127.5 (C$_{Ar}$), 126.4 (q, ^3J = 5.5 Hz, CH$_{Ar}$), 124.0 (q, ^1J = 273.5 Hz, *C*F$_3$), 121.3 (C$_{Ar}$), 119.7 (q, ^1J = 328.5 Hz, SO$_2$*C*F$_3$), 113.6 (CH$_{Ar}$), 113.1 (CH$_{Ar}$), 58.3 (*C*HTf), 56.2 (OMe), 56.1 (OMe), 52.4 (COOMe), 33.2 (CH$_2$); ^{19}F NMR (CDCl$_3$, 282 MHz, 25 °C) δ –77.2 (3F, s, SO$_2$CF$_3$), –59.1 (3F, s CF$_3$-Ar); IR (cm^{-1}) ν 2950 (CH$_3$), 1700 (CO), 1500, 1390 (*SO$_2$*CF$_3$), 1300 (CH$_3$), 1250 (-CO-O-), 1200 (SO$_2$*CF$_3$*), 1150 (CF$_3$) 1100 (*SO$_2$*CF$_3$),

1050 (Ar), 800 (*o*-Ar), 700 (CH_2); HRMS (ESI-TOF) m/z: $[M+H]^+$ Calcd. for $C_{22}H_{19}F_6O_6S$ 525.0801; Found 525.0800.

Compound 3q

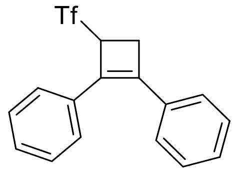

Following the general procedure and starting from 29 mg of alkyne **2q**, 45 mg (77%) of compound **3q** were obtained as a colorless solid, after purification on column chromatography using hexanes/AcOEt (10:1) as eluent; m.p. 75-77 °C; ^{1}H NMR ($CDCl_3$, 300 MHz, 25 °C) δ 7.57–7.49 (2H, m, H_{Ar}), 7.43 (2H, m, H_{Ar}), 7.34–7.24 (6H, m, H_{Ar}), 4.86 (1H, dd, *J* = 4.7, 1.9 Hz, *CH*Tf), 3.26 (1H, ddd, *J* = 14.0, 1.9, 1.0 Hz, CH_2), 3.08 (1H, dd, *J* = 14.0, 4.7 Hz, CH_2); ^{13}C {^{1}H} NMR ($CDCl_3$, 75 MHz, 25 °C) δ 144.1 (1C, C_{Ar}), 132.9 (1C, C_{Ar}), 132.3 (1C, C_{Ar}), 130.3 (1C, C_{Ar}), 129.8 (1C, CH_{Ar}), 129.1 (1C, CH_{Ar}), 128.7 (2C, CH_{Ar}), 128.7 (2C, CH_{Ar}), 127.4 (2C, CH_{Ar}), 126.6 (2C, CH_{Ar}), 119.8 (1C, q, *J* = 326 Hz, CF_3), 56.4 (*CH*Tf), 29.1 (CH_2); ^{19}F NMR ($CDCl_3$, 282 MHz, 25 °C) δ –76.43 (3F, s, CF_3); HRMS (ESI-TOF) m/z: $[M+NH_4]^+$ Calcd. for $C_{17}H_{17}F_3O_2SN$ 356.0927; Found 356.0927.

Compound 3r

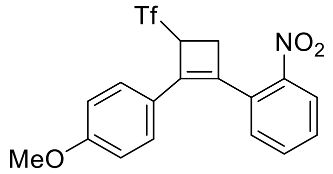

Following the general procedure and starting from 13 mg of alkyne **2r**, 15 mg (69%) of compound **3r** were obtained as a yellow solid, after purification on column chromatography using hexanes/AcOEt (8:1) as eluent; m.p. 104-106 °C; ^{1}H NMR ($CDCl_3$, 300 MHz, 25 °C) δ 8.14–7.92 (1H, m, H_{Ar}), 7.67–7.40 (3H, m, H_{Ar}), 7.25 (2H, d, *J* = 8.9 Hz, $H_{Ar\text{-}PMP}$), 6.72 (2H, d, *J* = 8.9 Hz, $H_{Ar\text{-}PMP}$), 4.96–4.71 (1H, m, *CH*Tf), 3.71 (3H, s, OMe), 3.24 (1H, dd, *J* = 13.9, 4.4 Hz, CH_2), 3.18–2.99 (1H, m, CH_2); ^{13}C {^{1}H} NMR ($CDCl_3$, 75 MHz, 25 °C) δ 160.4 (1C, C_{Ar}), 147.2 (1C, C_{Ar}), 138.6 (1C, C_{Ar}), 133.9 (1C, CH_{Ar}), 133.4 (1C, C_{Ar}), 131.3 (1C, CH_{Ar}), 129.9 (1C, CH_{Ar}), 129.7 (1C, C_{Ar}), 128.4 (2C, CH_{Ar}), 125.1 (1C, CH_{Ar}), 123.7 (1C, C_{Ar}), 120.0 (1C, q, J = 327 Hz, CF_3), 114.1 (2C, CH_{Ar}), 57.1 (*CH*Tf), 55.3 (OMe), 32.5 (CH_2); ^{19}F NMR ($CDCl_3$, 282 MHz, 25 °C) δ –76.43 (3F, s, CF_3); HRMS (ESI-TOF) m/z: $[M+Na]^+$ Calcd for $C_{18}H_{14}F_3NO_5SNa$ 436.0437; Found 436.0434.

Compound 3s

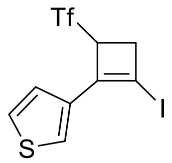

Following the general procedure and starting from 18.3 mg of alkyne **2s**, 22.7 mg (71%) of compound **3s** were obtained as a thick brown oil, after purification on column chromatography using hexanes/AcOEt (50:1) as eluent; ^{1}H NMR ($CDCl_3$, 300 MHz, 25 °C) δ 7.90 (1H, dd, *J* = 2.9, 1.3 Hz, H_{Ar}), 7.71 (1H, dd, *J* = 5.1, 1.3 Hz, H_{Ar}), 7.38 (1H, dd, *J* = 5.1, 2.9 Hz, H_{Ar}), 4.99 (1H, dd, *J* = 4.2, 1.9 Hz, C*H*Tf), 3.33 (1H, ddt, *J* = 14.2, 1.9, 0.9 Hz, CH_2), 3.25 (1H, dd, *J* = 14.2, 4.2 Hz, CH_2); ^{13}C {^{1}H} NMR ($CDCl_3$, 75 MHz, 25 °C) δ 139.5 (Ar-*C*=C-I), 132.3 (C_{Ar}), 126.3 (CH_{Ar}), 126.1 (CH_{Ar}), 124.6 (CH_{Ar}), 119.8 (q, ^{1}J = 328.95 Hz, SO_2CF_3), 87.2 (Ar-C=*C*-I), 61.9 (*CH*Tf), 38.5 (CH_2); ^{19}F NMR ($CDCl_3$, 282 MHz, 25 °C) δ –75.9 (3F, s, CF_3); HRMS (ESI-TOF) m/z: $[M+Na]^+$ Calcd. for $C_9H_6F_3IO_2S_2Na$ 416.8698; Found 416.8692.

Compound 3t

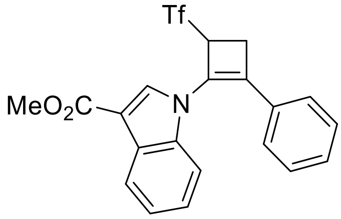

Following the general procedure and starting from 19.5 mg of alkyne **2t**, 31.4mg (89%) of compound **3t** were obtained as a yellow solid, after purification on column chromatography using hexanes/AcOEt (5:1) as eluent; m.p. 125-127 °C; ^{1}H NMR ($CDCl_3$, 300 MHz, 25 °C) δ 8.28 (1H, dt, *J* = 7.9, 1.2 Hz, H_{Ar}), 8.00 (1H, s, H_{Ar}), 7.47–7.27 (5H, m, H_{Ar}), 7.32–7.19 (1H, m, H_{Ar}), 7.19 (1H, d, *J* = 1.2 Hz), 7.16 (1H, d, *J* = 1.7 Hz, H_{Ar}), 5.11 (1H, dd, *J* = 4.6, 1.7 Hz, C*H*Tf), 3.96 (3H, s, COOMe), 3.40 (1H, dd, *J* = 13.4, 1.2 Hz, CH_2), 3.28 (1H, dd, *J* = 13.4, 4.6 Hz, CH_2); ^{13}C {^{1}H} NMR ($CDCl_3$, 75 MHz, 25 °C) δ 164.9 (*C*OOMe), 144.1 (C_{Ar}), 135.7 (C_{Ar}), 132.3 (CH_{Ar}), 131.0 (CH_{Ar}), 129.9 (C_{Ar}), 129.0 (2C, CH_{Ar}), 127.6 (2C, CH_{Ar}), 126.5 (C_{Ar}), 124.3 (CH_{Ar}), 123.4 (CH_{Ar}), 122.2 (CH_{Ar}), 119.6 (q, ^{1}J = 328.3 Hz, SO_2CF_3), 119.5 (C_{Ar}), 111.8 (CH_{Ar}), 111.3 (C_{Ar}), 58.9 (*CH*Tf), 51.5 (COOMe), 26.6 (CH_2); ^{19}F NMR ($CDCl_3$, 282 MHz, 25 °C) δ –77.0 (3F, s, CF_3); HRMS (ESI-TOF) m/z: $[M-H]^-$ Calcd. for $C_{21}H_{15}F_3NO_4S$ 434.0679; Found 434.0669.

Compound 3u

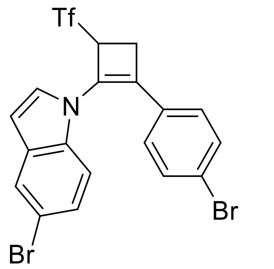

Following the general procedure and starting from 28.2 mg of alkyne **2u**, 20.2 mg (50%) of compound **3u** were obtained as a thick yellow oil, after purification on column chromatography using hexanes/AcOEt (5:1) as eluent; ^{1}H NMR ($CDCl_3$, 300 MHz, 25 °C) δ 7.81 (1H, d, *J* = 1.7 Hz, H_{Ar}), 7.46 (2H, d, *J* = 8.6 Hz, $H_{Ar\text{-}PMP}$), 7.31 (1H, dd, *J* = 8.7, 1.7 Hz, H_{Ar}), 7.28 (1H, d, *J* = 3.2 Hz, H_{Ar}), 7.00 (2H, d, *J* = 8.6 Hz, $H_{Ar\text{-}PMP}$), 6.96 (1H, dd, *J* = 8.7, 0.8 Hz, H_{Ar}), 6.68 (1H, dd, *J* = 3.2, 0.8 Hz, H_{Ar}), 5.04 (1H, dd, *J* = 4.6, 1.8 Hz, C*H*Tf), 3.32 (1H, ddd, *J* = 13.2, 1.8, 0.8 Hz, CH_2), 3.20 (1H, dd, *J* = 13.2, 4.6 Hz, CH_2); ^{13}C {^{1}H} NMR ($CDCl_3$, 75 MHz, 25 °C) δ 139.5 (C_{Ar}), 133.6 (C_{Ar}), 132.2 (CH_{Ar}), 130.7 (C_{Ar}), 129.3 (C_{Ar}), 128.9 (CH_{Ar}), 127.1 (CH_{Ar}), 126.3 (CH_{Ar}), 125.0 (C_{Ar}), 124.2 (CH_{Ar}), 121.1 (C_{Ar}), 119.7 (q, ^1J = 328.33 Hz, SO_2CF_3), 115.0 (C_{Ar}), 113.2 (CH_{Ar}), 105.6 (CH_{Ar}), 59.0 (*CH*Tf), 26.5 (CH_2); ^{19}F NMR ($CDCl_3$, 282 MHz, 25 °C) δ –76.8 (3F, s, CF_3); HRMS (ESI-TOF) m/z: $[M-H]^-$ Calcd. for $C_{19}H_{11}Br_2F_3NO_2S$, 531.8835; Found, 531.8836.

Compound 3u-d$_2$

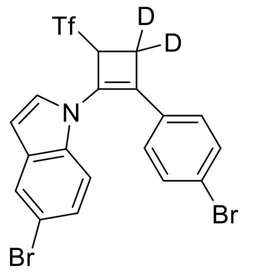

Following the general procedure and starting from 12.4 mg of alkyne **2u**, 9.4 mg (53%) of compound **3u-d$_2$** were obtained as a thick brown oil, after purification on column chromatography using hexanes/AcOEt (30:1) as eluent; ^{1}H NMR ($CDCl_3$, 300 MHz, 25 °C) δ 7.81 (1H, d, *J* = 2.0 Hz, H_{Ar}), 7.46 (2H, d, *J* = 8.5 Hz, $H_{Ar\text{-}PMP}$), 7.29 (2H, dd, *J* = 8.9, 2.0 Hz, H_{Ar}), 7.00 (2H, d, *J* = 8.5 Hz, $H_{Ar\text{-}PMP}$), 6.96 (1H, d, *J* = 8.9 Hz, H_{Ar}), 6.68 (1H, dd, *J* = 3.4, 0.9 Hz, H_{Ar}), 5.03 (1H, s, C*H*Tf); ^{13}C {^{1}H} NMR ($CDCl_3$, 75 MHz, 25 °C) δ 139.3 (C_{Ar}), 133.6 (C_{Ar}), 132.3 (CH_{Ar}), 130.7 (C_{Ar}), 129.3 (C_{Ar}), 129.0 (CH_{Ar}), 127.1 (CH_{Ar}), 126.3 (CH_{Ar}), 125.0 (C_{Ar}), 124.2 (CH_{Ar}), 121.2 (C_{Ar}), 119.7 (q, ^1J = 327.5 Hz, SO_2CF_3), 115.0 (C_{Ar}), 113.2 (CH_{Ar}), 105.7 (CH_{Ar}), 58.8 (*CH*Tf); ^{19}F NMR ($CDCl_3$, 282 MHz, 25 °C) δ –76.8 (3F, s, CF_3); ^{2}H NMR ($CDCl_3$, 107 MHz, 25 °C) δ 3.25 (2D, d, *J* = 12.0 Hz, CD_2); HRMS (ESI-TOF) m/z: $[M-H]^-$ Calcd. for $C_{19}H_9D_2Br_2F_3NO_2S$ 533.8960; Found 533.8965.

Compound 3v

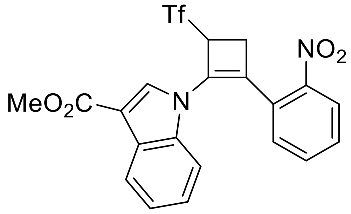

Following the general procedure and starting from 13 mg of alkyne **2v**, 17 mg (70 %) of compound **3v** were obtained as a yellow solid, after purification on column chromatography using hexanes/AcOEt (3:1) as eluent; m.p. 133-135 °C; ^{1}H NMR ($CDCl_3$, 300 MHz, 25 °C) δ 8.20 (1H, dd, *J* = 8.0, 0.6 Hz, H_{Ar}), 8.16 (1H, ddd, *J* = 8.2, 2.3, 1.1 Hz, H_{Ar}), 8.01 (1H, t, *J* = 1.9 Hz, H_{Ar}), 7.51–7.35 (1H, m, H_{Ar}), 7.33–7.15 (4H, m, H_{Ar}), 7.07–6.96 (1H, m, H_{Ar}), 5.07 (1H, dd, *J* = 4.6, 1.7 Hz, *CH*Tf), 3.88 (3H, s, COOMe), 3.45–3.31 (1H, m, CH_2), 3.26 (1H, dd, *J* = 13.4, 4.6 Hz, CH_2); ^{13}C {^{1}H} NMR ($CDCl_3$, 75 MHz, 25 °C) δ 164.5 (1C, C=O), 148.4 (1C, C_{Ar}), 139.6 (1C, C_{Ar}), 135.2 (1C, C_{Ar}), 133.2 (1C, CH_{Ar}), 131.5 (1C, CH_{Ar}), 131.4 (1C, C_{Ar}), 130.1 (1C, CH_{Ar}), 126.5 (1C, C_{Ar}), 125.0 (1C, CH_{Ar}), 124.5 (1C, CH_{Ar}), 123.7 (1C, CH_{Ar}), 122.7 (1C, C_{Ar}), 122.5 (1C, CH_{Ar}), 122.1 (1C, CH_{Ar}), 119.6 (1C, q = 327 Hz, CF_3), 112.1 (1C, C_{Ar}), 111.6 (1C, CH_{Ar}), 58.6 (*CH*Tf), 51.5 (Me), 26.6 (CH_2); ^{19}F NMR ($CDCl_3$, 282 MHz, 25 °C) δ –76.43 (3F, s, CF_3); IR (cm^{-1}) ν 2950 (CH_3), 2350, 1700 (CO), 1550 (NO_2), 1450 (NO_2), 1390 (*SO_2*CF_3), 1200 (SO_2*CF_3*), 1100 (*SO_2*CF_3), 750 (*o*-Ar), 700 (CH_2), 650; HRMS (ESI-TOF) m/z: $[M+Na]^+$ Calcd. for $C_{21}H_{15}F_3N_2O_6SNa$ 503.0495; Found 503.0498.

Compound 3w

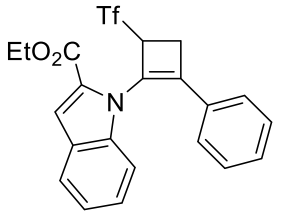

Following the general procedure and starting from 15 mg of alkyne **2w**, 20 mg (91 %) of compound **3w** were obtained as a yellow solid, after purification on column chromatography using hexanes/AcOEt (20:1) as eluent; m.p. 131-133 °C; ^{1}H NMR ($CDCl_3$, 300 MHz, 25 °C) δ 7.64 (1H, dt, *J* = 7.8, 1.1 Hz, H_{Ar}), 7.41 (1H, s, H_{Ar}), 7.29–7.06 (6H, m, H_{Ar}), 7.00–6.87 (2H, m, H_{Ar}), 5.36 (1H, dd, *J* = 4.7, 1.9 Hz, *CH*Tf), 4.29 (2H, m, COO*CH_2*CH_3), 3.29 (1H, dd, *J* = 13.2, 1.9 Hz, *CH_2*CHTf), 3.19 (1H, dd, *J* = 13.2, 4.7 Hz, *CH_2*CHTf), 1.33 (3H, t, *J* = 7.1 Hz, $COOCH_2$*CH_3*); ^{13}C {^{1}H} NMR ($CDCl_3$, 75 MHz, 25 °C) δ 161.5 (1C, C=O), 144.0 (1C, C_{Ar}), 137.8 (1C, C_{Ar}), 130.5 (1C, C_{Ar}), 130.2 (1C, CH_{Ar}), 128.6 (2C, CH_{Ar}), 127.4 (1C, C_{Ar}), 127.2 (2C, CH_{Ar}), 126.6 (1C, CH_{Ar}), 126.6 (1C, C_{Ar}), 122.7 (1C, CH_{Ar}), 122.4 (1C, CH_{Ar}), 121.2 (1C, C_{Ar}), 119.4 (1C, q, J = 327 Hz, CF_3), 113.4 (1C, CH_{Ar}), 112.8 (1C,

CH_{Ar}), 61.1 (CH_2, COOEt), 59.8 (*CH*Tf), 25.6 (CH_2), 14.3 (Me); ^{19}F NMR ($CDCl_3$, 282 MHz, 25 °C) δ –77.86 (3F, s, CF_3); HRMS (ESI-TOF) m/z: $[M+Na]^+$ Calcd for $C_{22}H_{18}F_3NO_4SNa$ 472.0801; Found 472.0807.

Compound 3x

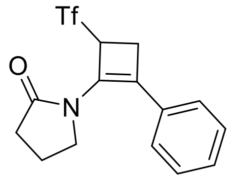

Following the general procedure and starting from 6 mg of alkyne **2x**, 7 mg (67%) of compound **3x** were obtained as a colorless solid, after purification on column chromatography using hexanes/AcOEt (5:1) as eluent; m.p. 96-98 °C; ^{1}H NMR ($CDCl_3$, 700 MHz, 25 °C) δ 7.42 (2H, t, *J* = 7.3 Hz, H_{Ar}), 7.38 (1H, t, *J* = 7.3 Hz, H_{Ar}), 7.31 (2H, d, *J* = 7.1 Hz, H_{Ar}), 5.22 (1H, dd, *J* = 4.5, 1.4 Hz, C*H*Tf), 3.89 (1H, ddd, *J* = 9.6, 7.3, 5.8 Hz, CH_2), 3.52 (1H, dt, *J* = 9.6, 7.5 Hz, CH_2), 3.18 (1H, d, *J* = 12.9 Hz, CH_2CHTf), 3.00 (1H, dd, *J* = 12.9, 4.5 Hz, CH_2CHTf), 2.50 (2H, t, *J* = 8.0 Hz, CH_2), 2.25–2.18 (2H, m, CH_2); ^{13}C {^{1}H} NMR ($CDCl_3$, 175 MHz, 25 °C) δ 175.9 (NC=O), 138.6 (C_{Ar}), 131.4 (C_{Ar}), 129.7 (CH_{Ar}), 128.9 (CH_{Ar}), 127.4 (CH_{Ar}), 121.9 (C_{Ar}), 119.9 (q, ^1J = 328.56 Hz, SO_2CF_3), 57.15 (*CH*Tf), 47.4 (CH_2), 30.6 (CH_2), 27.7 (*CH$_2$*CHTf), 19.6 (CH_2); ^{19}F NMR ($CDCl_3$, 282 MHz, 25 °C) δ –77.2 (3F, s, CF_3); HRMS (ESI-TOF) m/z: $[M+Na]^+$ Calcd. for $C_{15}H_{14}F_3NO_3SNa$ 368.0539; Found 368.0539.

Compound 3y

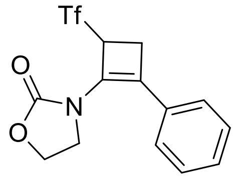

Following the general procedure and starting from 10.7 mg of alkyne **2y**, 13.4 mg (67%) of compound **3y** were obtained as a colorless solid, after purification on column chromatography using hexanes/AcOEt (6:1) as eluent; m.p. 108-110 °C; ^{1}H NMR ($CDCl_3$, 300 MHz, 25 °C) δ 7.43 (3H, td, *J* = 5.4, 2.7 Hz, H_{Ar}), 7.39–7.32 (2H, m, H_{Ar}), 5.15 (1H, dd, *J* = 4.5, 1.7 Hz, C*H*Tf), 4.54 (2H, dd, *J* = 8.6, 7.2 Hz, CH_2), 4.09 (1H, dt, *J* = 8.6, 7.2 Hz, CH_2), 3.78 (1H, q, *J* = 8.6 Hz, CH_2), 3.17 (1H, ddd, *J* = 13.1, 1.7, 0.7 Hz), 3.01 (1H, dd, *J* = 13.1, 4.5 Hz, CH_2); ^{13}C {^{1}H} NMR ($CDCl_3$, 75 MHz, 25 °C) δ 156.0 (CO), 140.1 (C_{Ar}), 130.95 (C_{Ar}), 130.1 (CH_{Ar}), 129.0 (CH_{Ar}), 127.4 (CH_{Ar}), 120.1 (C_{Ar}), 119.8 (q, ^1J = 328.0 Hz, SO_2CF_3), 63.6 (CH_2), 57.3 (*CH*Tf), 44.8 (CH_2), 27.5 (*CH$_2$*CHTf); ^{19}F NMR ($CDCl_3$, 282 MHz, 25 °C) δ –77.1 (3F, s, CF_3); IR (cm^{-1}) ν 2950, 1750 (NCO), 1400 ($CH_{2\text{-cyclic}}$), 1390 (*SO$_2$*CF_3), 1200

(SO_2*CF_3*), 1100 (*SO_2*CF_3), 1000 (Ar), 800 (Ar), 700 (CH_2), 650; HRMS (ESI-TOF) m/z: $[M+Na]^+$ Calcd. for $C_{14}H_{12}F_3NO_4SNa$ 370.0331; Found 370.0327.

Compound 3z

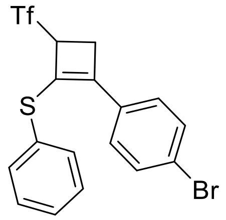

Following the general procedure and starting from 32.7 mg of alkyne **2z**, 34.3 mg (67%) of compound **3z** were obtained as a thick yellow oil, after purification on column chromatography using hexanes/AcOEt (7:1) as eluent; ^{1}H NMR ($CDCl_3$, 300 MHz, 25 °C) δ 7.53 (4H, d, *J* = 8.7 Hz, $H_{Ar\text{-}PMP}$), 7.45 (2H, dd, *J* = 7.6, 1.7 Hz, H_{Ar}), 7.34 (3H, dd, *J* = 7.6, 0.9 Hz, H_{Ar}), 4.56 (1H, dd, *J* = 4.1, 2.1 Hz, C*H*Tf), 3.25 (1H, dd, *J* = 13.7, 2.1 Hz, CH_2), 3.21 (1H, dd, *J* = 13.7, 4.1 Hz, CH_2); ^{13}C {^{1}H} NMR ($CDCl_3$, 75 MHz, 25 °C) δ 151.9 (C_{Ar}), 132.1 (CH_{Ar}), 131.5 (CH_{Ar}), 131.2 (C_{Ar}), 130.7 (C_{Ar}), 129.6 (CH_{Ar}), 128.5 (CH_{Ar}), 128.3 (CH_{Ar}), 124.7 (C_{Ar}), 121.5 (C_{Ar}), 119.7 (q, ^1J = 328.85 Hz, SO_2CF_3), 57.9 (*CH*Tf), 30.0 (CH_2); ^{19}F NMR ($CDCl_3$, 282 MHz, 25 °C) δ –76.4 (3F, s, CF_3); HRMS (ESI-TOF) m/z: $[M-H]^-$ Calcd. for $C_{17}H_{11}BrF_3O_2S_2$ 446.9341; Found 446.9339.

Compound 7a

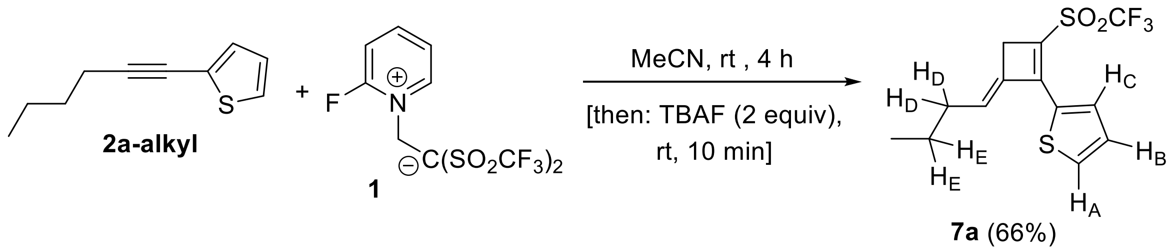

Following the general procedure and starting from 25 mg of alkyne **2a-alkyl**, 32.3 mg (66%) of compound **7a** were obtained as a thick brown oil, after purification on column chromatography using hexanes/AcOEt (200:1) as eluent; ^{1}H NMR ($CDCl_3$, 300 MHz, 25 °C) δ 8.01 (1H, dd, *J* = 3.9, 1.0 Hz, H_C), 7.64 (1H, dd, *J* = 5.0, 1.0 Hz, H_A), 7.18 (1H, dd, *J* = 5.0, 3.9 Hz, H_B), 6.32 (1H, tt, *J* = 7.7, 1.3 Hz, CH), 3.43 (2H, s, C*H_2*CTf), 2.20 (2H, q, *J* = 7.4 Hz, $CH_{2\text{-}D}$), 1.54 (2H, h, *J* = 7.4 Hz, $CH_{2\text{-}E}$), 0.98 (3H, t, *J* = 7.4 Hz, CH_3); ^{13}C {^{1}H} NMR ($CDCl_3$, 75 MHz, 25 °C) δ 153.8 (*C*=CTf), 134.3 (CH_C), 133.0 (*C*=CH), 132.5 (CH_A), 130.4 (C_{Ar}-S), 129.4 (*CH*-$CH_{2\text{-}D}$), 128.6 (CH_B), 120.4 (q, ^1J = 326.4 Hz, SO_2*CF_3*), 117.4 (*C*-Tf), 36.1 (*CH_2*CTf), 30.9 ($CH_{2\text{-}D}$), 22.5 ($CH_{2\text{-}E}$), 14.0 (CH_3); ^{19}F NMR ($CDCl_3$, 282 MHz, 25 °C) δ –78.3 (3F, s, CF_3); HRMS (ESI-TOF) m/z: $[M+H]^+$ Calcd. for $C_{13}H_{14}F_3O_2S_2$ 323.0382; Found 323.0376.

Compound 4d

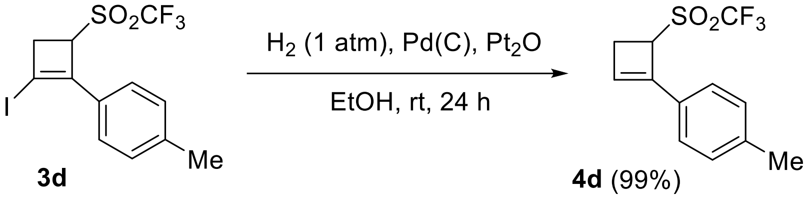

15.6 mg of cyclobutene **3d**, Pt_2O (5 mol%) and Pd/C 10% (5 mol%) were placed in a round bottom flask in ethanol (100 mL/mmol). H_2(g) was bubbled up into the reaction flask for 20 minutes at room temperature. The reaction was stirred overnight under a balloon of H_2 (g). The crude reaction mixture was filtered through a celite pad and evaporated under reduced pressure. The mixture was purified on column chromatography using hexanes/AcOEt (200:1) as eluent to give 8 mg (99%) of compound **4d** as an orange solid; m.p. 81-83 °C; ^{1}H NMR (acetone-d_6, 700 MHz, 25 °C) δ 7.50 (2H, d, *J* = 8.0 Hz, H_{Ar}), 7.21 (2H, d, *J* = 8.0 Hz, H_{Ar}), 6.77 (1H, d, *J* = 1.5 Hz, C=C*H*), 5.46 (1H, d, *J* = 4.3 Hz, C*H*Tf), 3.09 (1H, ddd, *J* = 14.8, 4.3, 1.5 Hz, CH_2), 2.89 (1H, d, *J* = 14.8 Hz, CH_2), 2.33 (3H, s, Me); ^{1}H NMR (acetone-d_6, 175 MHz, 25 °C) δ 139.9 (C_{Ar}), 139.8 (C_{Ar}), 133.8 (C=*CH*), 129.9 (2C, CH_{Ar}), 126.4 (2C, CH_{Ar}), 120.9 (q, ^{1}J = 328.65 Hz, SO_2CF_3), 59.2 (*CH*Tf), 30.5 (CH_2), 21.3 (Me); ^{19}F NMR (acetone-d_6, 282 MHz, 25 °C) δ –77.6 (3F, s, CF_3); IR (cm^{-1}) ν 2950 (CH_3), 2890 (CH), 1390 (*SO_2*CF_3), 1375 (CH_3), 1200 (SO_2*CF_3*), 1250, 1100 (*SO_2*CF_3), 800 (*o*-Ar), 700 (CH_2), 650, 500; HRMS (ESI-TOF) m/z: $[M+Na]^+$ Calcd. for $C_{12}H_{11}F_3O_2SNa$ 299.0324; Found 299.0331.

Compound 4i

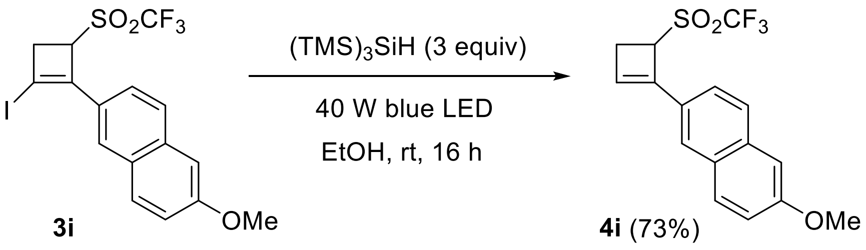

A solution of iodocyclobutene **3i** (20 mg, 0.043 mmol), $(TMS)_3SiH$ (42 mL, 0.129 mmol) in ethanol (0.5 mL) was stirred at room temperature under blue led irradiation for 16h. After that time, the solvent was evaporated and the residue was purified on column chromatography using hexanes/AcOEt (8:1) as eluent to give product **4i** (11 mg, 73%) as an orange solid; m.p. 84-86 °C; ^{1}H NMR ($CDCl_3$, 300 MHz, 25 °C) δ 7.87 (1H, br s, H_{Ar}), 7.72–7.63 (1H, m, H_{Ar}), 7.62 (1H, s, H_{Ar}), 7.50 (1H, dd, *J* = 8.5, 1.8 Hz, H_{Ar}), 7.08 (1H, dd, *J* = 8.9, 2.6 Hz, H_{Ar}), 7.04 (1H, m, H_{Ar}), 6.59 (1H, s, C=C*H*), 4.94 (1H, t, *J* = 3.1 Hz, *CH*Tf), 3.85 (3H, s, OMe), 3.09–2.83 (2H, m, CH_2); ^{13}C {^{1}H} NMR

($CDCl_3$, 75 MHz, 25 °C) δ 158.5 (1C, C_{Ar}), 139.3 (1C, C_{Ar}), 134.8 (1C, C_{Ar}), 132.2 (C=CH), 130.2 (1C, CH_{Ar}), 128.4 (1C, C_{Ar}), 127.1 (1C, C_{Ar}), 127.1 (1C, CH_{Ar}), 126.5 (1C, C_{Ar}), 125.3 (1C, CH_{Ar}), 123.0 (1C, CH_{Ar}), 119.9 (1C, q, J = 272 MHz, CF_3), 119.5 (1C, CH_{Ar}), 105.9 (1C, CH_{Ar}), 58,5 (*CH*Tf), 54,9 (OMe), 29.9 (CH_2); ^{19}F NMR ($CDCl_3$, 282 MHz, 25 °C) δ –76.13 (3F, s, CF_3); HRMS (ESI-TOF) m/z: $[M+Na]^+$ Calcd for $C_{16}H_{13}F_3O_3SNa$ 365.0430; Found 365.0438.

General procedure for the Suzuki coupling reaction.

The appropriate cyclobutene **3** (1 equiv.), the corresponding boronic acid (1.5 equiv.), $Pd(PPh_3)_4$ (5 mol%), K_2CO_3 (3 equiv.) and 1,4-dioxane (27 ml/mmol)/H_2O (11.5 ml/mmol) were added to an oven-dried round bottom flask. The reaction was stirred at 40–60 °C until disappearance of the starting material (TLC). The crude reaction mixture was then extracted with AcOEt (3 x 15mL). The organic phases were combined and dried under $MgSO_4$. After evaporation of the solvent under reduced pressure, the mixture was purified on column chromatography using mixtures of hexanes and AcOEt.

Compound 5i

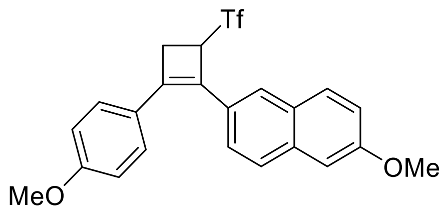

Starting from iodocyclobutene **3i** (15 mg, 0.032 mmol) and (4-methoxyphenyl)boronic acid (7 mg, 0.048 mmol), product **5i** (9 mg, 64%) was obtained as a yellow oil after purification on column chromatography using hexanes/AcOEt (6:1); ^{1}H NMR ($CDCl_3$, 300 MHz, 25 °C) δ 7.92 (1H, d, *J* = 1.7 Hz, H_{Ar}), 7.66 (2H, dd, *J* = 8.7, 3.0 Hz, H_{Ar}), 7.56 (1H, dd, *J* = 8.5, 1.8 Hz, H_{Ar}), 7.42 (2H, d, *J* = 8.8 Hz, $H_{Ar\text{-}PMP}$), 7.14 – 6.97 (2H, m, H_{Ar}), 6.80 (2H, d, *J* = 8.8 Hz, $H_{Ar\text{-}PMP}$), 4.92 (1H, dd, *J* = 4.7, 1.8 Hz, *CH*Tf), 3.86 (3H, s, OMe), 3.76 (3H, s, OMe), 3.25 (1H, d, *J* = 13.9 Hz, CH_2), 3.07 (1H, dd, *J* = 13.9, 4.6 Hz, CH_2); ^{13}C {^{1}H} NMR ($CDCl_3$, 75 MHz, 25 °C) δ 160.7 (1C, C_{Ar}), 158.5 (1C, C_{Ar}), 143.3 (1C, C_{Ar}), 134.6 (1C, C_{Ar}), 129.9 (1C, CH_{Ar}), 128,5 (1C, $_{Ar}$), 128.1 (2C, CH_{Ar}), 128.0 (1C, C_{Ar}), 127.8 (1C, C_{Ar}), 127.1 (1C, CH_{Ar}), 126.7 (1C, CH_{Ar}), 125.8 (1C, C_{Ar}), 125.2 (1C, CH_{Ar}), 119.4 (1C, CH_{Ar}), 114.0 (2C, CH_{Ar}), 105.8 (1C, CH_{Ar}), 56.7 (*CH*Tf), 55.4 (OMe), 55.4 (OMe), 29.5 (CH_2); ^{19}F NMR ($CDCl_3$, 282 MHz, 25 °C) δ –76.42 (3F, s, CF_3); HRMS (ESI-TOF) m/z: $[M+Na]^+$ Calcd for $C_{23}H_{19}F_3O_4SNa$ 471.0848; Found 471.0854.

Compound 5k

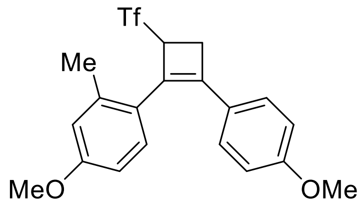

Starting from 13.6 mg of cyclobutene **3k**, 6 mg (35%) of compound **5k** were obtained as a thick yellow oil after purification on column chromatography using hexanes/AcOEt (20:1) as eluent; ^{1}H NMR ($CDCl_3$, 700 MHz, 25 °C) δ 7.28 (1H, d, *J* = 8.5 Hz, H_{Ar}), 7.20 (2H, d, *J* = 8.8 Hz, $H_{Ar\text{-}PMP}$), 6.83 (2H, d, *J* = 8.8 Hz, $H_{Ar\text{-}PMP}$), 6.79 (1H, s, H_{Ar}), 6.78 (1H, dd, *J* = 8.5 Hz, 2.6 Hz, H_{Ar}), 4.87 (1H, dd, *J* = 4.6, 1.8 Hz, C*H*Tf), 3.84 (3H, s, OMe), 3.81 (3H, s, OMe), 3.31 (1H, dd, *J* = 13.6, 1.8 Hz, CH_2), 3.14 (1H, dd, *J* = 13.6, 4.6 Hz, CH_2), 2.29 (3H, s, Me); ^{13}C {^{1}H} NMR ($CDCl_3$, 175 MHz, 25 °C) δ 160.7 (C_{Ar}-OMe), 159.9 (C_{Ar}-OMe), 145.2 (C_{Ar}), 138.5 (C_{Ar}), 130.1 (CH_{Ar}), 128.1 (CH_{Ar}), 127.6 (C_{Ar}), 125.9 (C_{Ar}), 125.4 (C_{Ar}), 119.9 (q, ^{1}J = 328.48 Hz, SO_2*C*F_3), 116.0 (CH_{Ar}), 114.1 (CH_{Ar}), 111.6 (CH_{Ar}), 58.2 (*CH*Tf), 55.5 (OMe), 55.3 (OMe), 28.5 (CH_2), 20.4 (Me); ^{19}F NMR ($CDCl_3$, 282 MHz, 25 °C) δ –76.9 (3F, s, CF_3); IR (cm^{-1}) ν 2950 (CH_3), 1600, 1520, 1490, 1390 (*SO_2*CF_3), 1250, 1200 (SO_2*CF_3*), 1150, 1100 (*SO_2*CF_3), 1000 (Ar), 850 (*p*-Ar), 700 (CH_2), 600, 400; HRMS (ESI-TOF) m/z: [M+Na]$^+$ Calcd for $C_{20}H_{19}F_3O_4SNa$ 435.0848; Found 435.0854.

Compound 5n

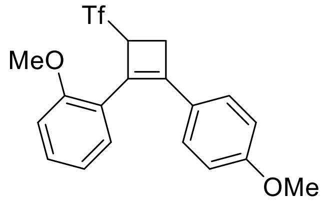

Starting from 19.5 mg of cyclobutene **3n**, 7.5 mg (40%) of compound **5n** were obtained as a thick yellow oil after purification on column chromatography using hexanes/AcOEt (40:1) as eluent; ^{1}H NMR ($CDCl_3$, 300 MHz, 25 °C) δ 7.52 (1H, dd, *J* = 7.7, 1.8 Hz, H_{Ar}), 7.39 (2H, d, *J* = 8.9 Hz, $H_{Ar\text{-}PMP}$), 7.33 (1H, dd, *J* = 7.7, 1.3 Hz), 7.03–6.90 (2H, m, H_{Ar}), 6.84 (2H, d, *J* = 8.9 Hz, $H_{Ar\text{-}PMP}$), 5.12 (1H, dd, *J* = 4.8, 1.9 Hz, C*H*Tf), 3.86 (3H, s, OMe), 3.82 (3H, s, OMe), 3.37 (1H, dd, *J* = 13.6, 1.9 Hz, CH_2), 3.15 (1H, dd, *J* = 13.6, 4.8 Hz, CH_2) ; ^{13}C {^{1}H} NMR ($CDCl_3$, 125 MHz, 25 °C) δ 160.7 (C_{Ar}-OMe), 157.6 (C_{Ar}-OMe), 144.5 (C_{Ar}), 130.5 (CH_{Ar}), 129.8 (CH_{Ar}), 128.2 ($CH_{Ar\text{-}PMP}$), 126.2 (C_{Ar}), 125.8 (C_{Ar}), 121.8 (C_{Ar}), 120.8 (CH_{Ar}), 114.0 ($CH_{Ar\text{-}PMP}$), 111.1 (CH_{Ar}), 57.6 (*CH*Tf), 55.5 (OMe), 55.4 (OMe), 29.5 (CH_2); ^{19}F NMR ($CDCl_3$, 470 MHz, 25 °C) δ –77.8 (3F, s, CF_3); IR (cm^{-1}) ν 2950 (CH_3), 2850 (CH_3), 2350, 2300, 1600, 1500, 1490, 1470, 1390 (*SO_2*CF_3), 1250, 1200 (SO_2*CF_3*), 1150, 1100 (*SO_2*CF_3), 1000, 850 (*p*-Ar), 750 (*o*-Ar), 700 (CH_2), 400; HRMS (ESI-TOF) m/z: [M-H]$^-$ Calcd. for $C_{19}H_{16}F_3O_4S$ 397.0727; Found 397.0725.

General procedure for the ring-opening reaction.

Cyclobutene **3d** (1 equiv.), the corresponding boronic acid (1.5 equiv.), $Pd(PPh_3)_4$ (5 mol%), K_2CO_3 (3 equiv.) and 1,4-dioxane (27 ml/mmol)/H_2O (11.5 ml/mmol) were added to an oven-dried microwave vessel. Next, the reaction mixture was heated under microwave irradiation at 85 °C for 6h. Then, the crude mixture was extracted with AcOEt (3 x 15mL). The organic phases were combined and dried with $MgSO_4$. After evaporation of the solvent under reduced pressure, the mixture was purified on column chromatography using mixtures of hexanes and AcOEt.

Compound 6da

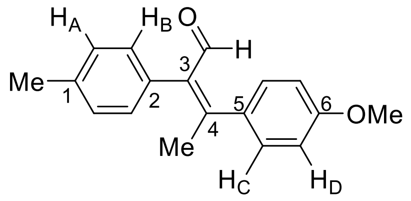

Starting from 14.5 mg of cyclobutene **3d**, 4.5 mg (63%) of compound **6da** were obtained as a yellow solid after purification on column chromatography using hexanes/AcOEt (70:1 → 10:1) as eluent. NMR showed a non-separable mixture (1:3) of E/Z isomers; m.p. 112-114 °C; ^{1}H NMR ($CDCl_3$, 700 MHz, 25 °C) δ 9.65 (1H, s, CHO), 7.32 (2H, d, *J* = 8.1 Hz, H_C), 7.24 (2H, d, *J* = 7.6 Hz, H_A), 7.08 (2H, d, *J* = 7.6 Hz, H_B), 6.97 (2H, d, *J* = 8.1 Hz, H_D), 3.87 (3H, s, OMe), 2.39 (3H, s, *Me*-Ar), 2.20 (3H, s, *Me*-C=CCHO); ^{13}C {^{1}H} NMR ($CDCl_3$, 175 MHz, 25 °C) δ 193.3 (CHO), 160.4 (C_6), 159.0 (C_4), 140.2 (C_3), 137.4 (C_1), 132.8 (C_2), 131.8 (C_5), 130.7 (CH_c), 130.0 (CH_B), 129.2 (CH_A), 113.9 (CH_D), 55.6 (OMe), 24.7 (*Me*-C=CCHO), 21.5 (*Me*-Ar); HRMS (ESI-TOF) m/z: $[M+Na]^+$ Calcd. for $C_{18}H_{18}O_2Na$ 289.1199; Found 289.1192.

Compound 6db

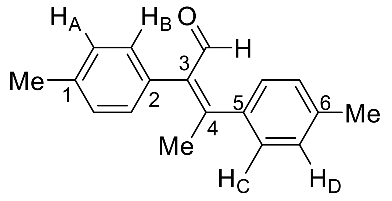

Starting from 21.7 mg of cyclobutene **3d**, 7.1 mg (39%) of compound **6db** were obtained as a yellow solid after purification on column chromatography using hexanes/AcOEt (200:1) as eluent. NMR showed a non-separable mixture (1:30) of E/Z isomers; m.p. 150-152 °C; ^{1}H NMR ($CDCl_3$, 500 MHz, 25 °C) δ 9.67 (1H, s, CHO), 7.29–7.27 (2H, m, H_{Ar}), 7.26 (4H, d, *J* = 8.0 Hz), 7.10 (2H, d, *J* = 8.1 Hz, H_{Ar}), 2.44 (3H, s, *Me*-Ar), 2.41 (3H, s, *Me*-Ar), 2.21 (3H, s, *Me*-C=CCHO); ^{13}C {^{1}H} NMR ($CDCl_3$, 125 MHz, 25 °C) δ 193.2 (CHO), 159.4 (C_4), 140.3 (C_3), 139.1 (C_5), 137.4 (C_1), 136.8

(C_6), 132.6 (C_2), 130.0 (CH_B), 129.21 (CH_C), 129.2 (CH_A), 129.1 (CH_D), 24.8 (*Me*-C=CCHO), 21.4 (*Me*-Ar), 21.4 (*Me*-Ar); IR (cm^{-1}) ν 2900 (CH_3), 2850 (CH_3), 1700 (CHO), 1600 (C=C), 1500, 1400, 1000, 800 (Ar); HRMS (ESI-TOF) m/z: $[M+H]^+$ Calcd. for $C_{18}H_{19}O$ 251.1430; Found 251.1434.

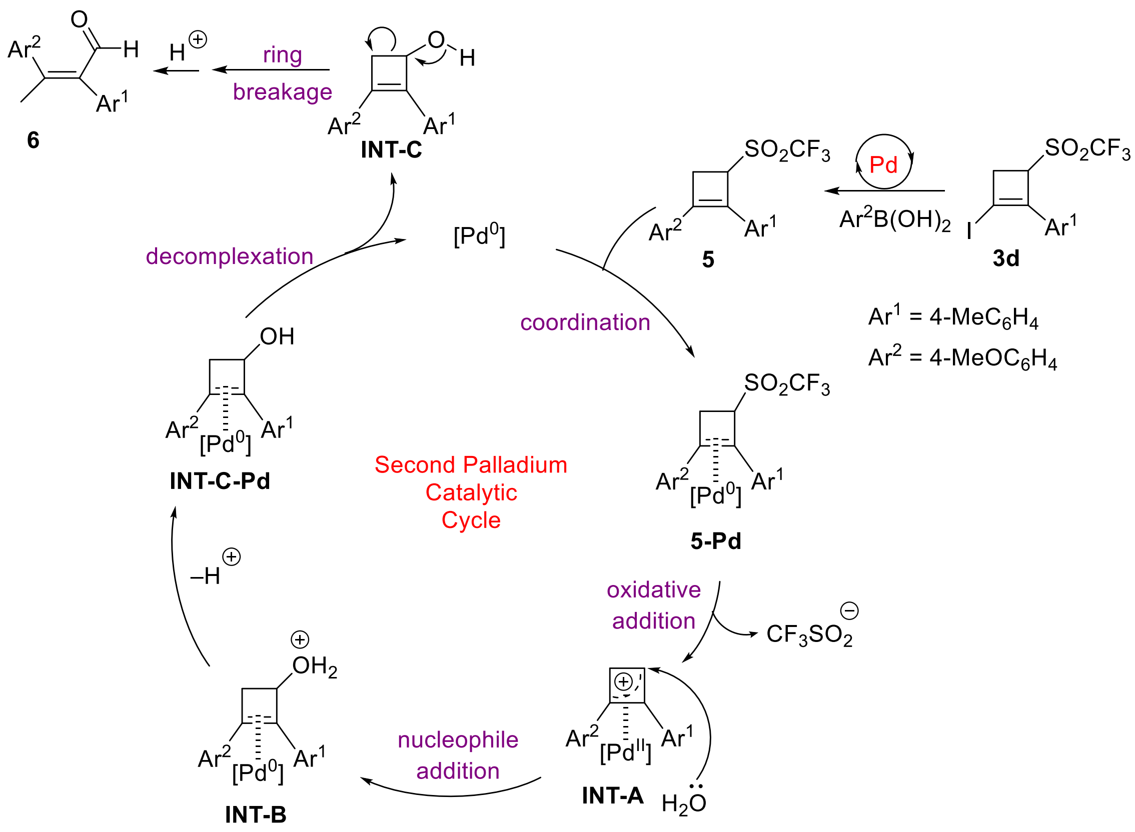

Scheme S12. Plausible Pathway for the Formation of Enals 6

A rationalization for the formation of tetrasubstituted enals **6** is delineated in Scheme S12. Early formation of Suzuki adducts **5** should occur by reaction between 1-iodocyclobutene **3d** and boronic acids. After completion of the first palladium catalytic cycle, intermediates **5** should enter the second catalytic cycle (allylic substitution-type reaction). Initial coordination to form complexes **5-Pd** should be followed by the genesis of π-allyl palladium intermediates **INT-A** through oxidative addition with the concurrent elimination of the CF_3SO_2 group. Further nucleophilic water attack to one site of the cyclic Pd(II) species should generate cationic intermediates **INT-B**. Next, deprotonation to form neutral intermediates **INT-C-Pd** followed by palladium decomplexation and regeneration of the catalytic species, should provide **INT-C**. Final products **6** should be forged by ring breakage and protonation.

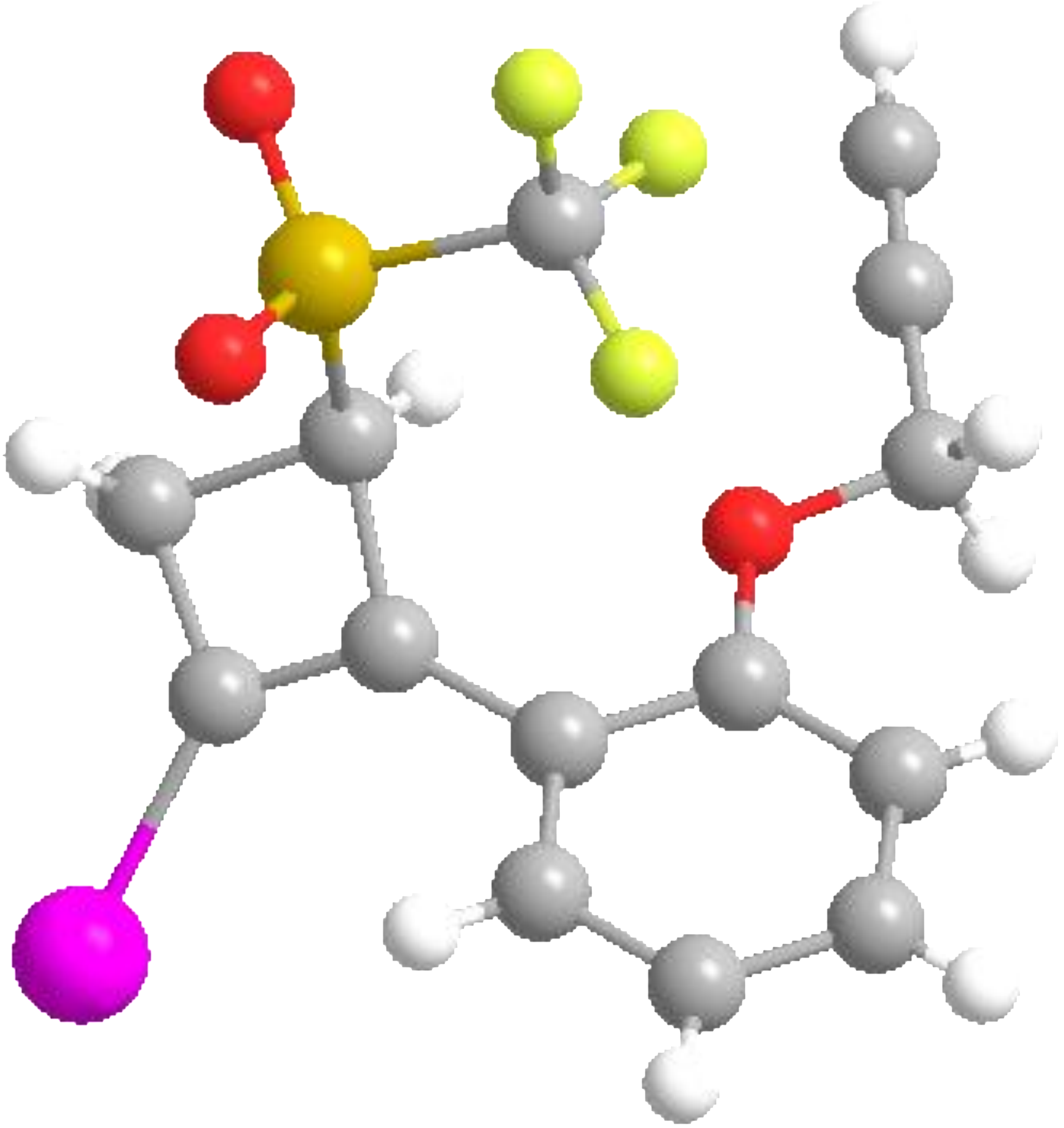

Figure S1. ORTEP diagram of **3o** at 50% probability ellipsoids.

Experimental. Single colourless block-shaped crystals of **3o** were obtained from slow evaporation of ethyl acetate-hexane (1:10) mixture at room temperature. A suitable crystal 0.25 ×0.21×0.04 mm^3 was selected and mounted on a Bruker Smart CCD difractometer using graphite-monochromated Mo-Kα radiation (λ = 0.71073 Å). The crystal was kept at a steady *T* = 296.15 K during data collection.

X-Ray data collection and structure refinement

Data collection for compound **3o** was carried out at room temperature on a Bruker Smart CCD difractometer using graphite-monochromated Mo-Kα radiation (λ = 0.71073 Å) operating at 50 kV and 40 mA. Data were collected over a sphere of the reciprocal space by combination of five exposure sets. Each exposure was of 20 s covered 0.3 in ω. The cell parameters were determined and refinement by least-squares fit of all reflections. The 50 frames were recollected at the end of the data collections to monitor crystal decay, and no appreciable decay was observed. The structure was solved by direct methods and refined by full-matrix least-square prodecures on F^2 (SHELXL-97)[1]. All non-hydrogen atoms were refined anisotropically. All hydrogen atoms were included in their calculated positions and refined riding on the respective carbon atoms. Mercury CSD 3.9[2] was used for molecular graphics.

A summary of the fundamental crystal and refinement data is given in Table S1.

Identification code	cl-1343b
Empirical formula	C14H10F3IO3S
Formula weight	442.18
Temperature/K	296.15
Crystal system	triclinic
Space group	P-1
a/Å	5.7213(6)
b/Å	8.9004(10)
c/Å	15.9378(17)
α/°	97.272(2)
β/°	91.201(2)
γ/°	101.228(2)
Volume/Å^3	788.78(15)
Z	2

ρcalcg/cm^3	1.862
μ/mm^{-1}	2.201
F(000)	428.0
Crystal size/mm^3	0.25 × 0.21 × 0.04
Radiation	MoKα (λ = 0.71073)
2Θ range for data collection/°	2.578 to 57.614
Index ranges	-7 ≤ h ≤ 7, -11 ≤ k ≤ 11, -20 ≤ l ≤ 20
Reflections collected	7889
Independent reflections	3717 [Rint = 0.0280, Rsigma = 0.0447]
Data/restraints/parameters	3717/0/199
Goodness-of-fit on F^2	1.067
Final R indexes [I>=2σ (I)]	R1 = 0.0451, wR2 = 0.1074
Final R indexes [all data]	R1 = 0.0741, wR2 = 0.1303
Largest diff. peak/hole / e Å^{-3}	1.01/-0.83

1. Siemens, SMART, SAINT and SHELXTL, Siemens Analytical X-ray Instruments INC., Madison, Wisconsin, USA, **2002**.
2. I.J. Bruno, J.A. Chisholm, P. R. Edgington, P. McCabe, E. Pidcock, L. Rodriguez-Monge, R. Taylor, J. Van de Streek and P. A. Wood, J. APPL. Crystallogr., **2008**, 41, 466-470.

^{1}H NMR compound **3a** ($CDCl_3$, 300 MHz, 25 °C)

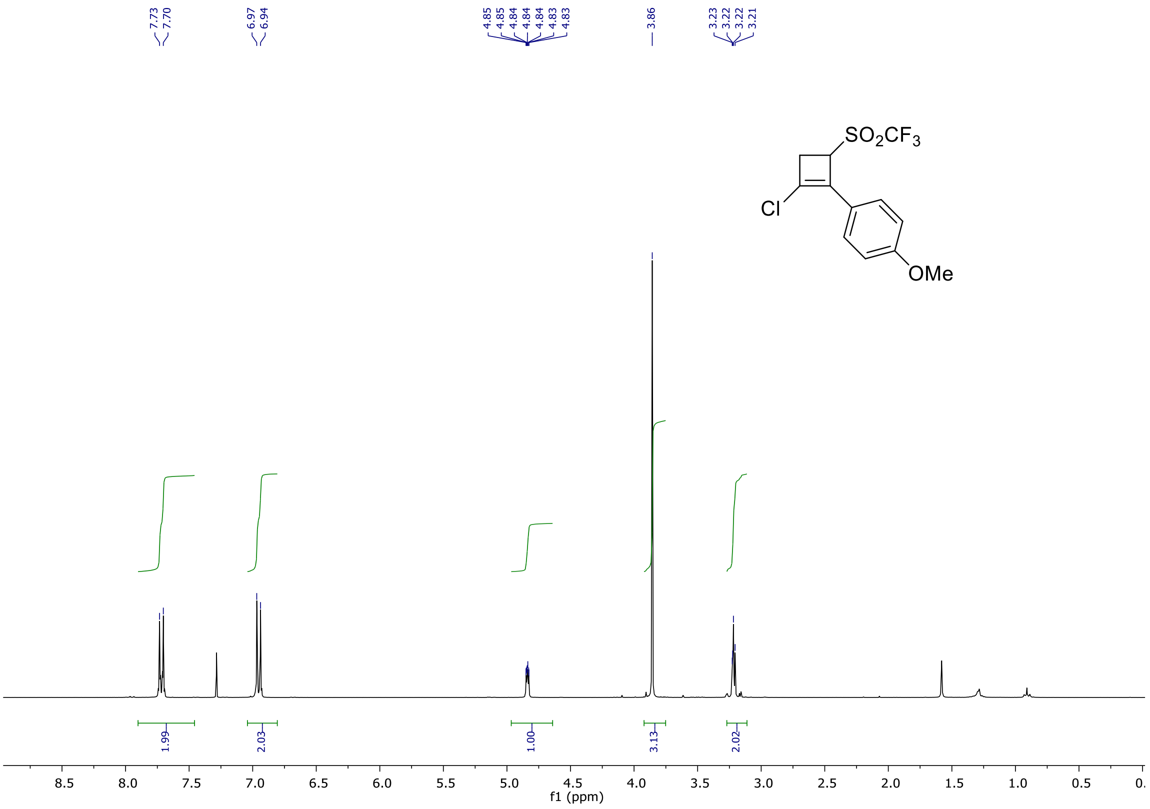

^{13}C NMR compound **3a** ($CDCl_3$, 75 MHz, 25 °C)

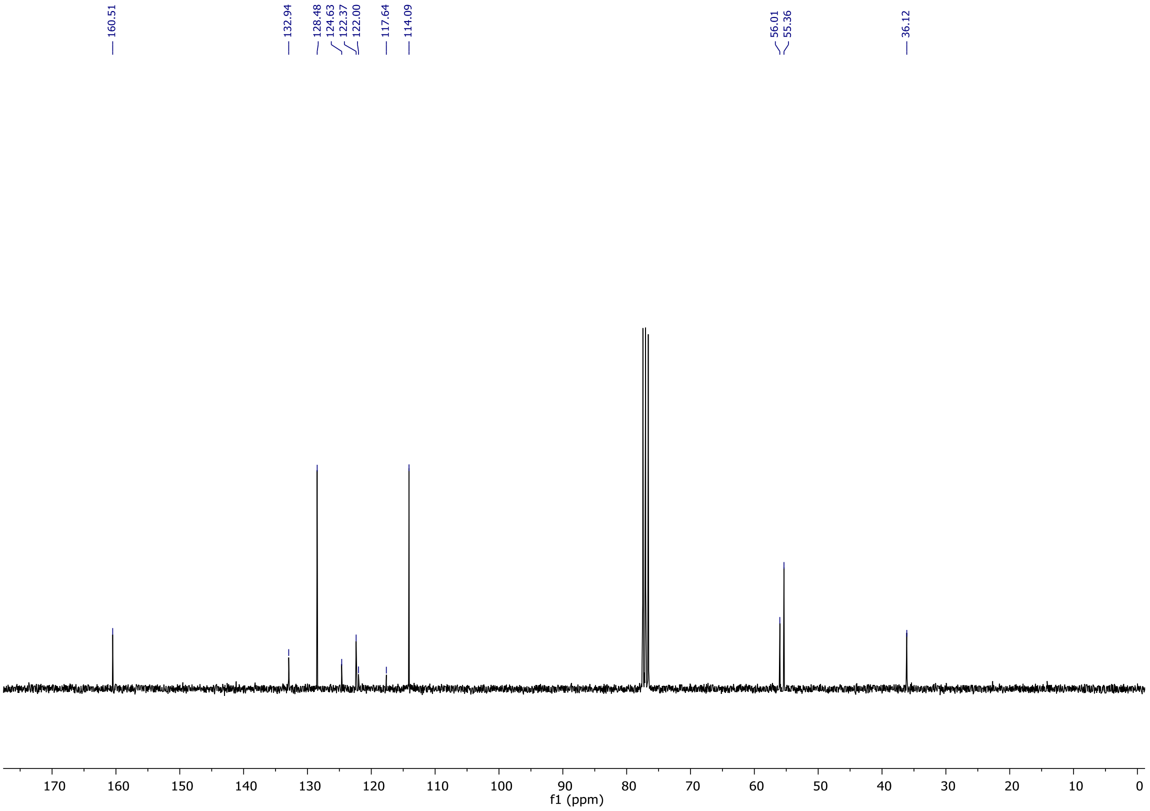

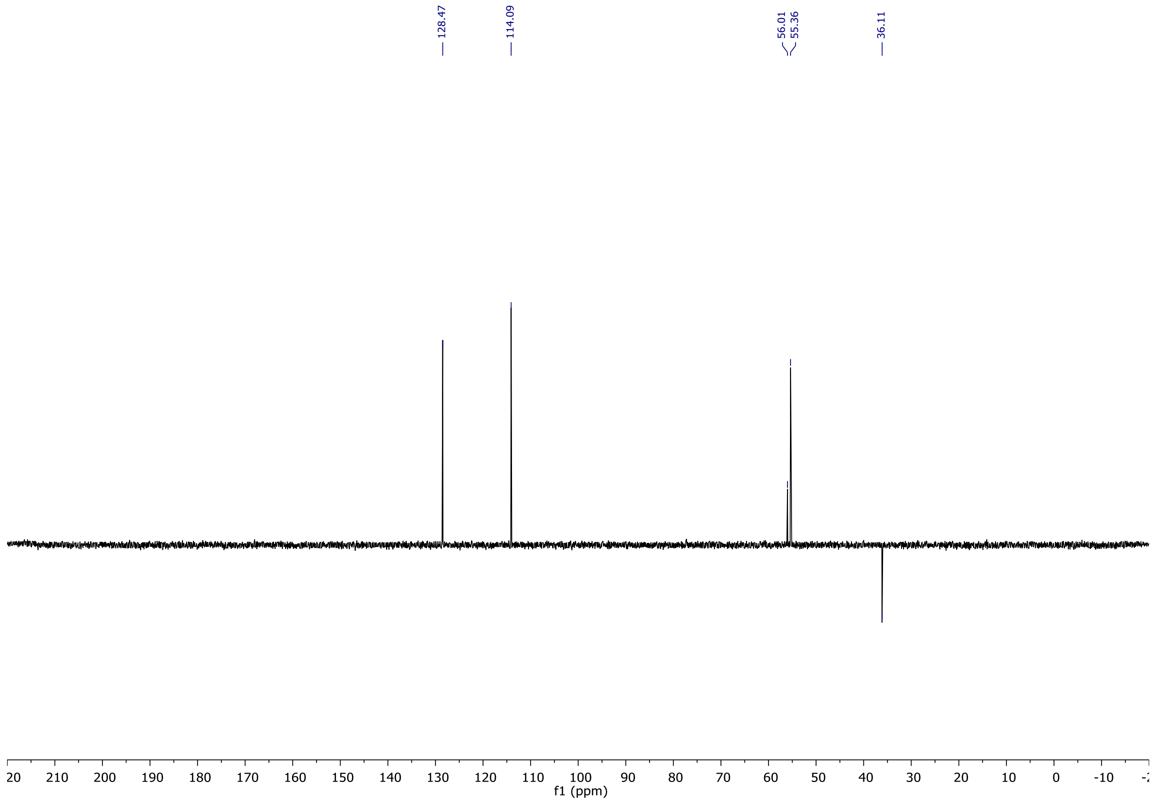

128.47
114.09
56.01
55.36
36.11
f1 (ppm)

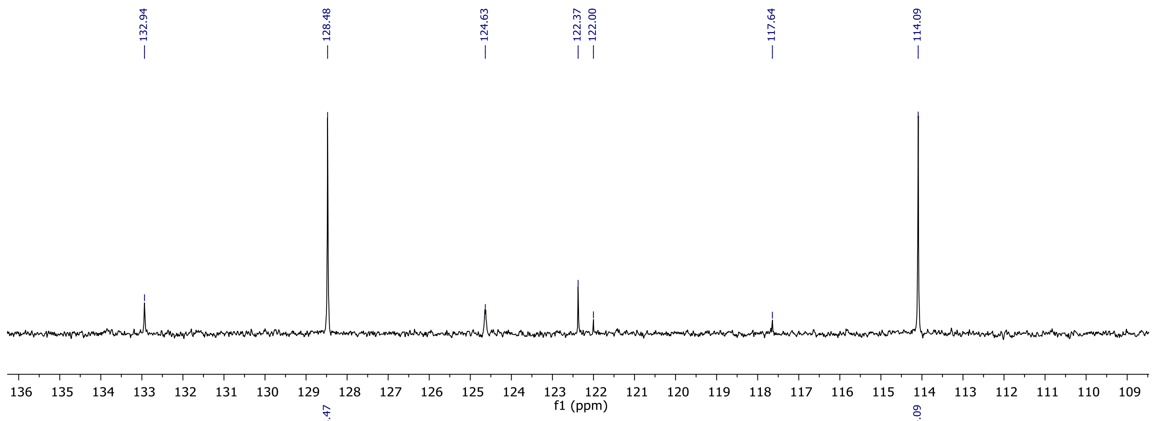

132.94
128.48
124.63
122.37
122.00
117.64
114.09
f1 (ppm)

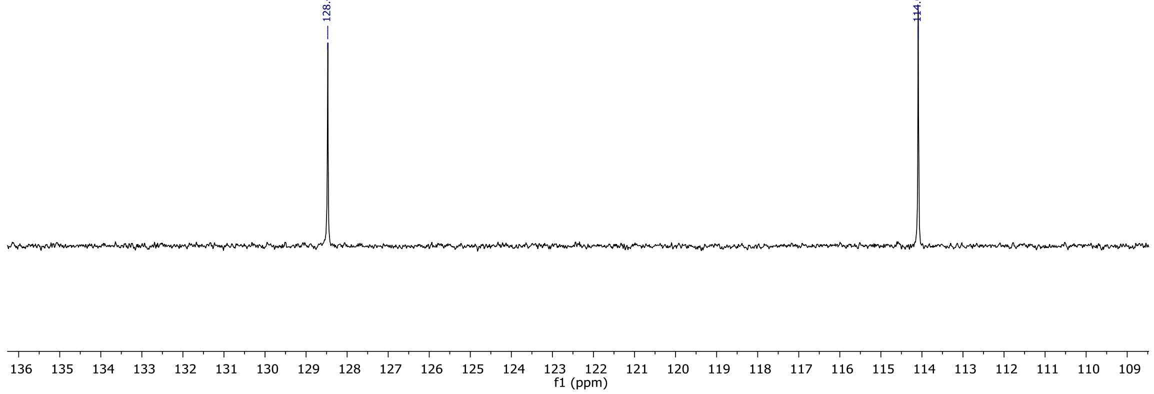

128.47
114.09
f1 (ppm)

^{19}F NMR compound **3a** ($CDCl_3$, 282 MHz, 25 °C)

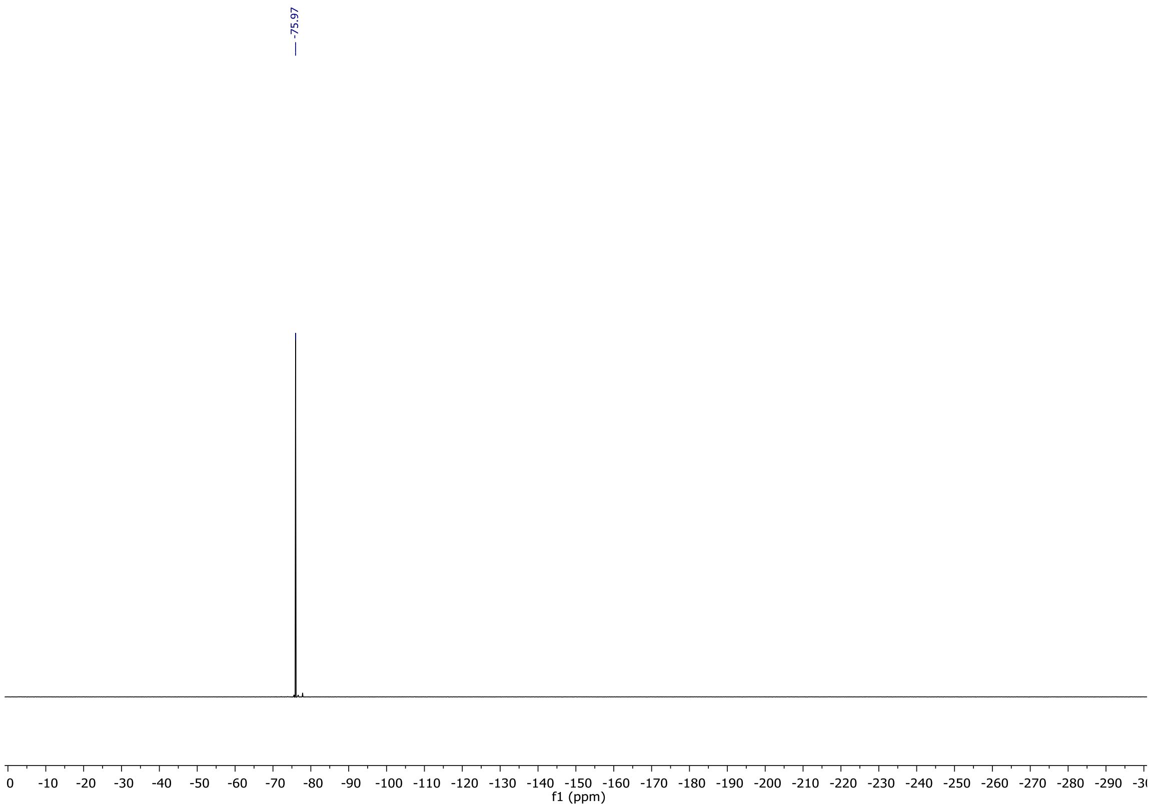

^{1}H NMR compound **3b** ($CDCl_3$, 300 MHz, 25 °C)

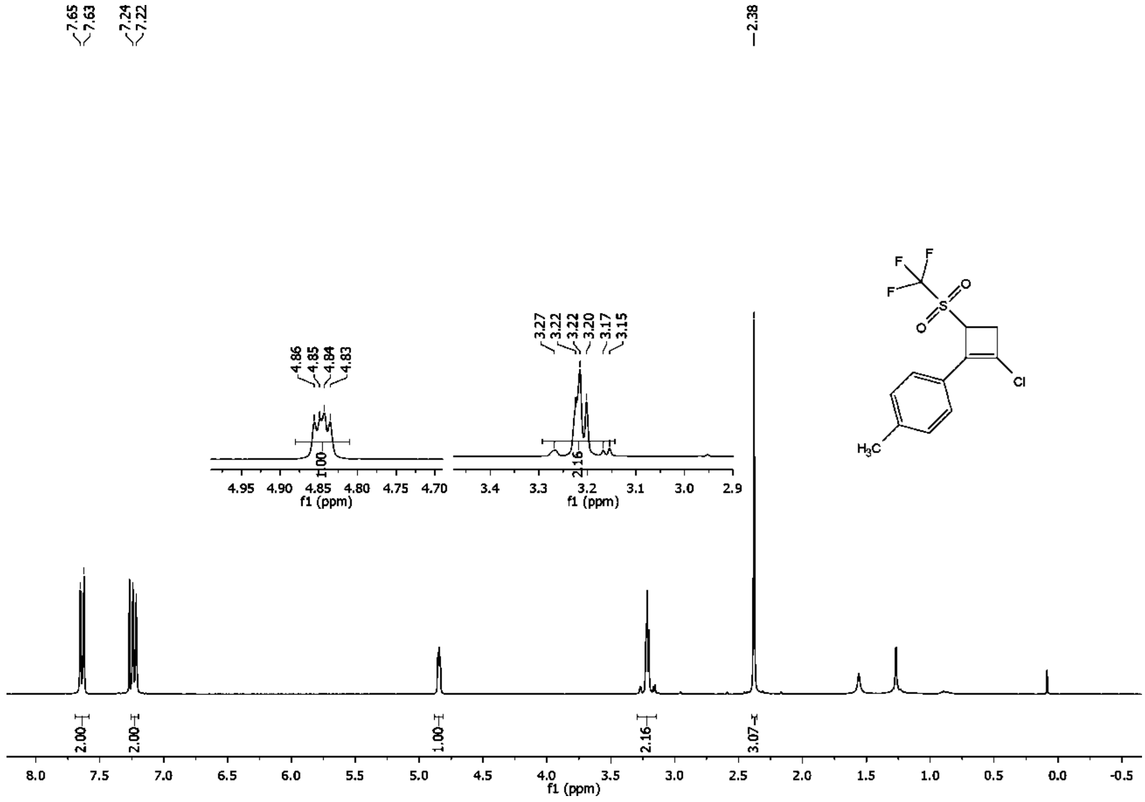

^{13}C NMR compound **3b** ($CDCl_3$, 75 MHz, 25 °C)

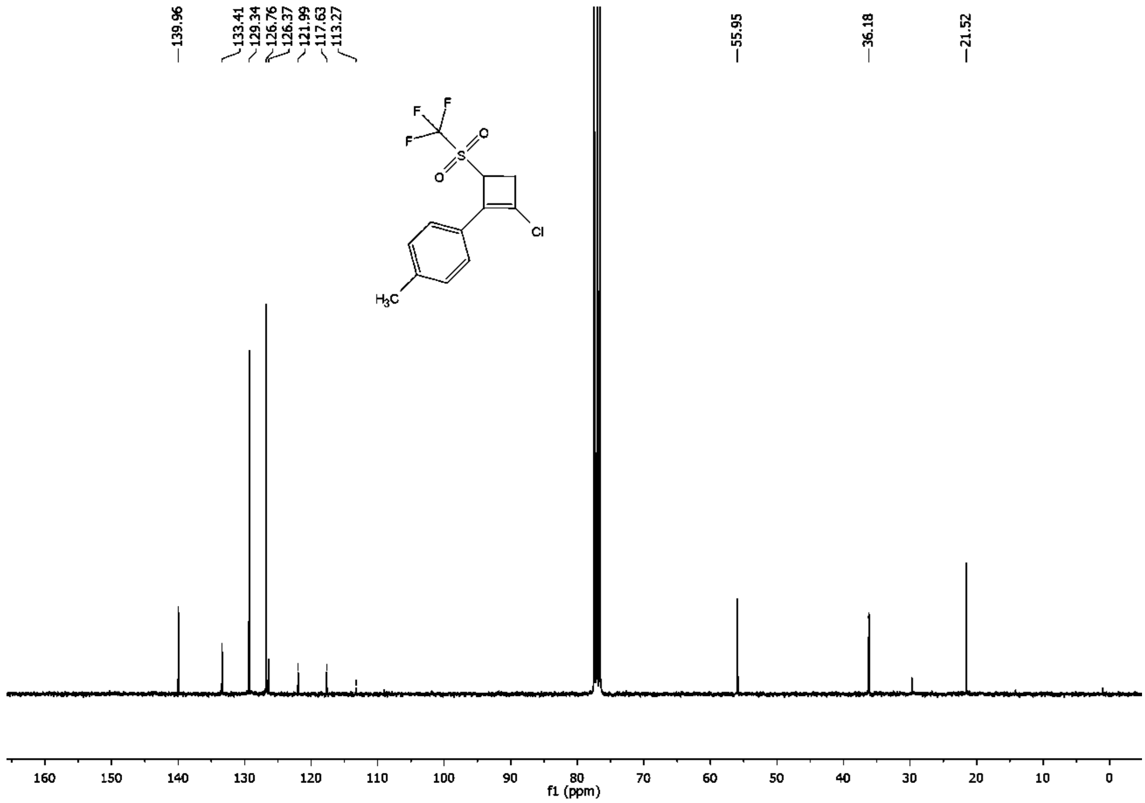

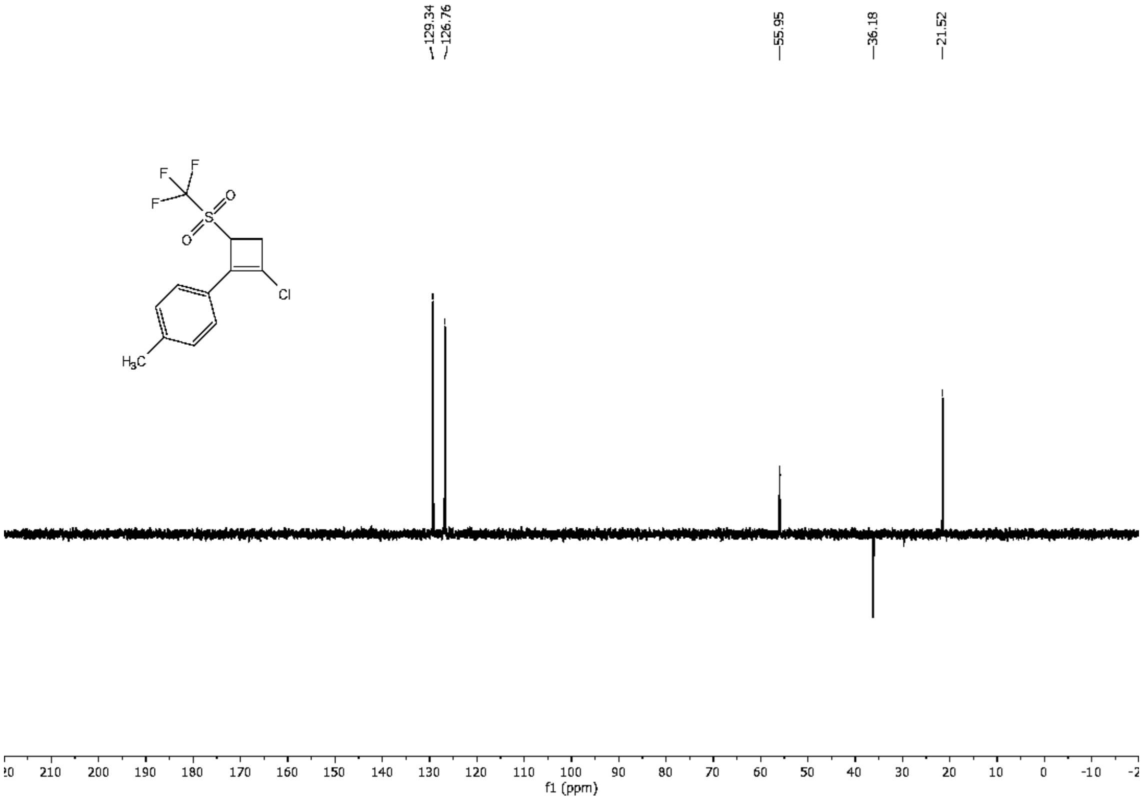

^{19}F NMR compound **3b** ($CDCl_3$, 282 MHz, 25 ^{o}C)

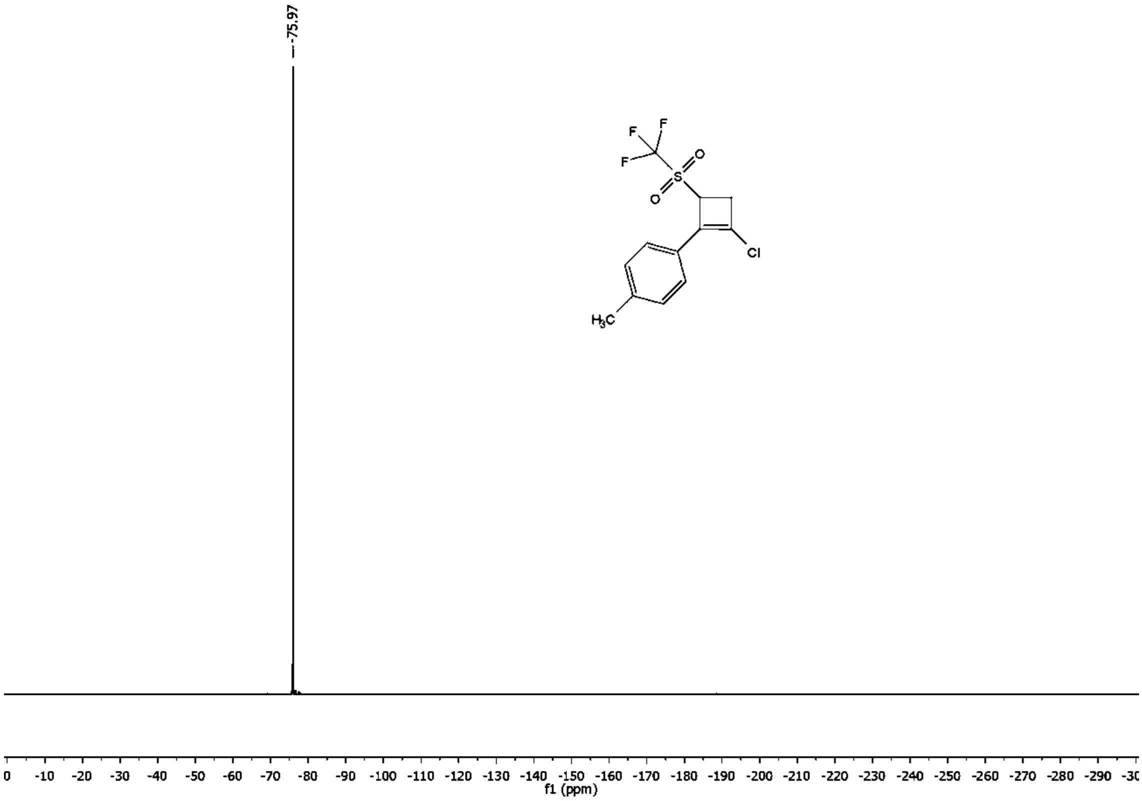

^{1}H NMR compound **3c** ($CDCl_3$, 300 MHz, 25 °C)

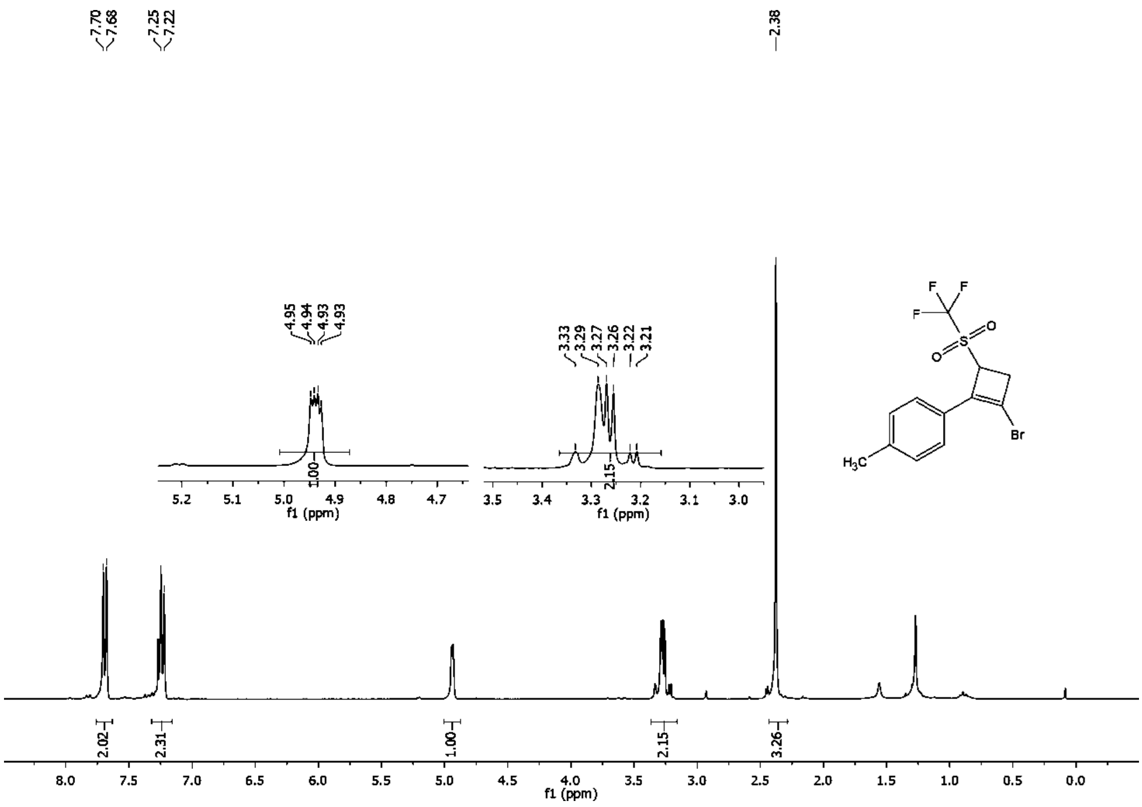

^{13}C NMR compound **3c** ($CDCl_3$, 75 MHz, 25 °C)

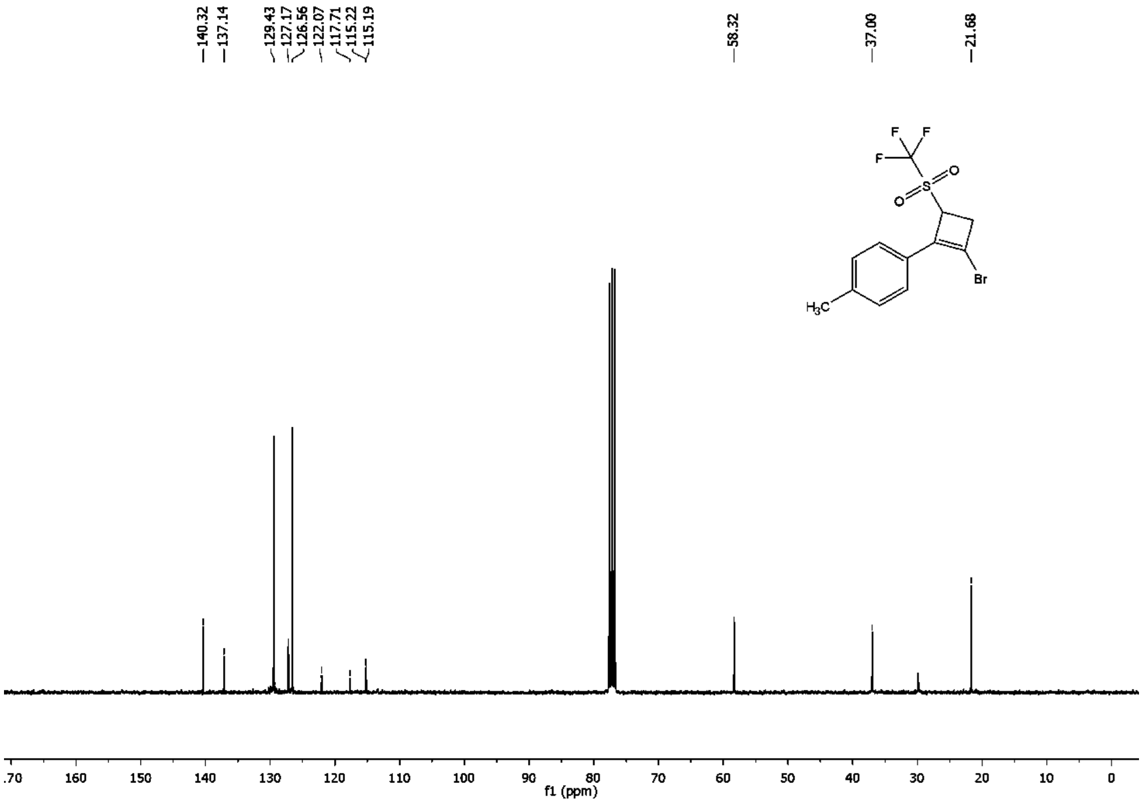

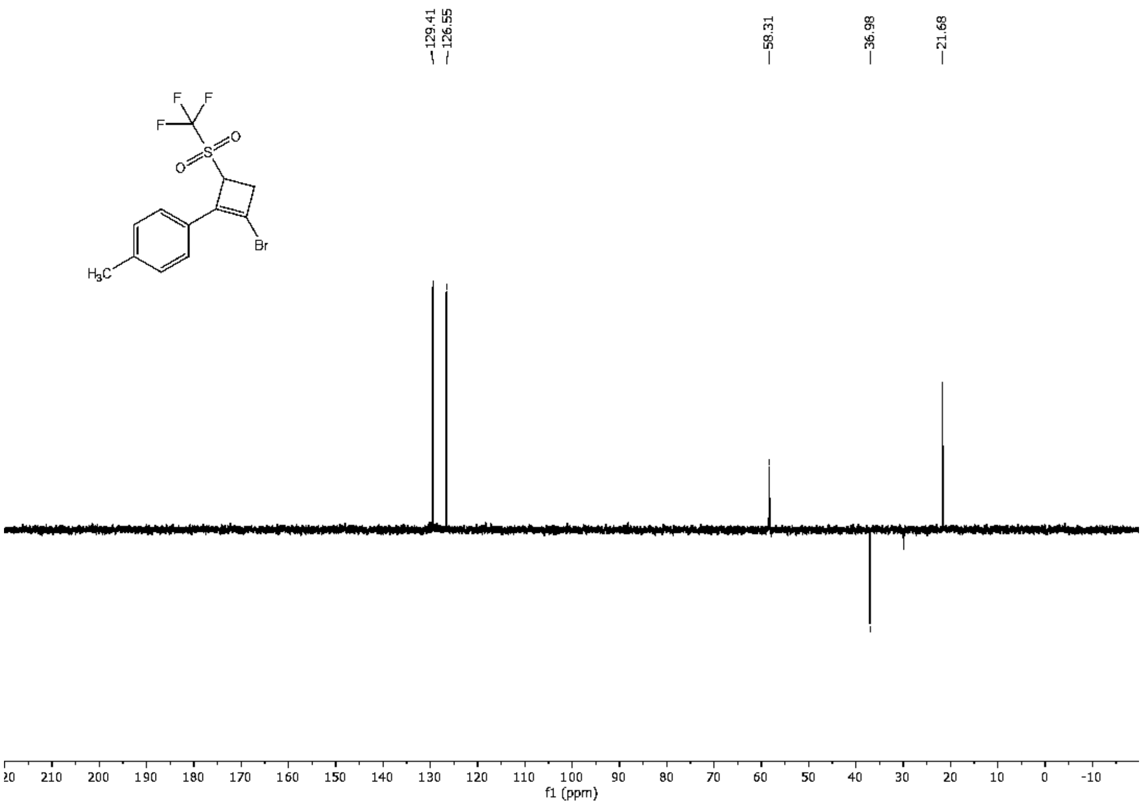

^{19}F NMR compound **3c** ($CDCl_3$, 282 MHz, 25 °C)

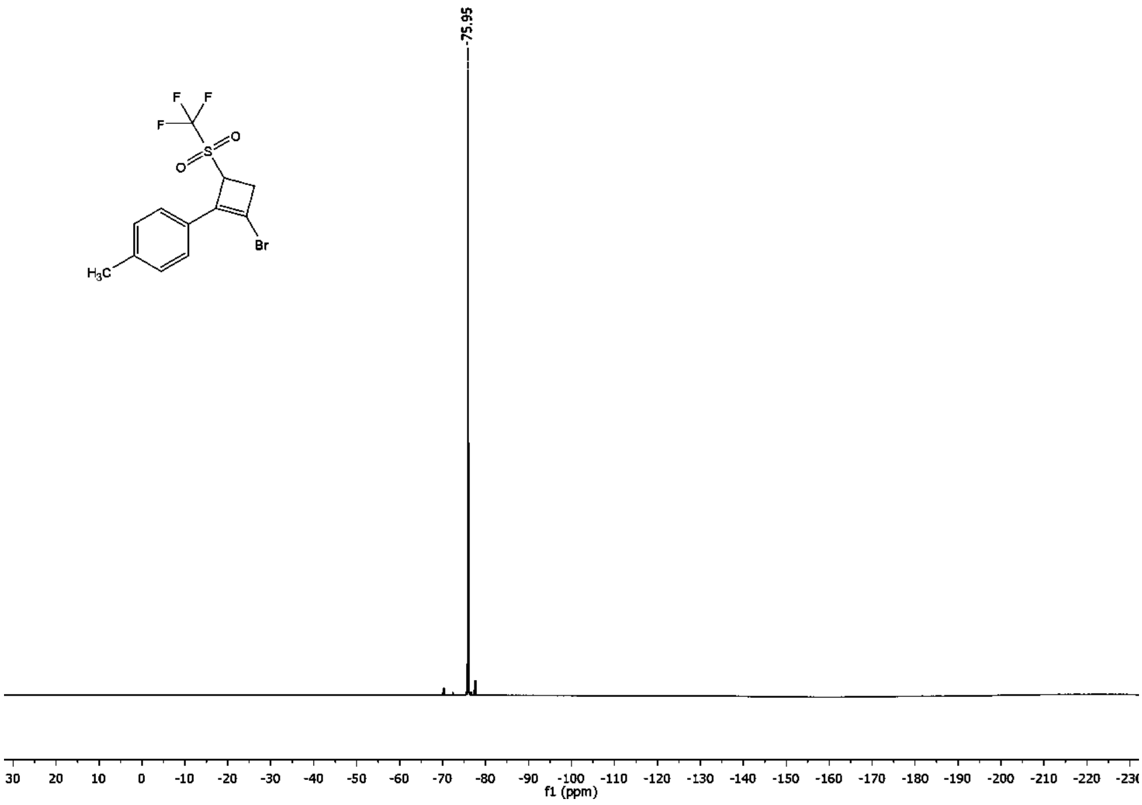

^{1}H NMR compound **3d** ($CDCl_3$, 300 MHz, 25 °C)

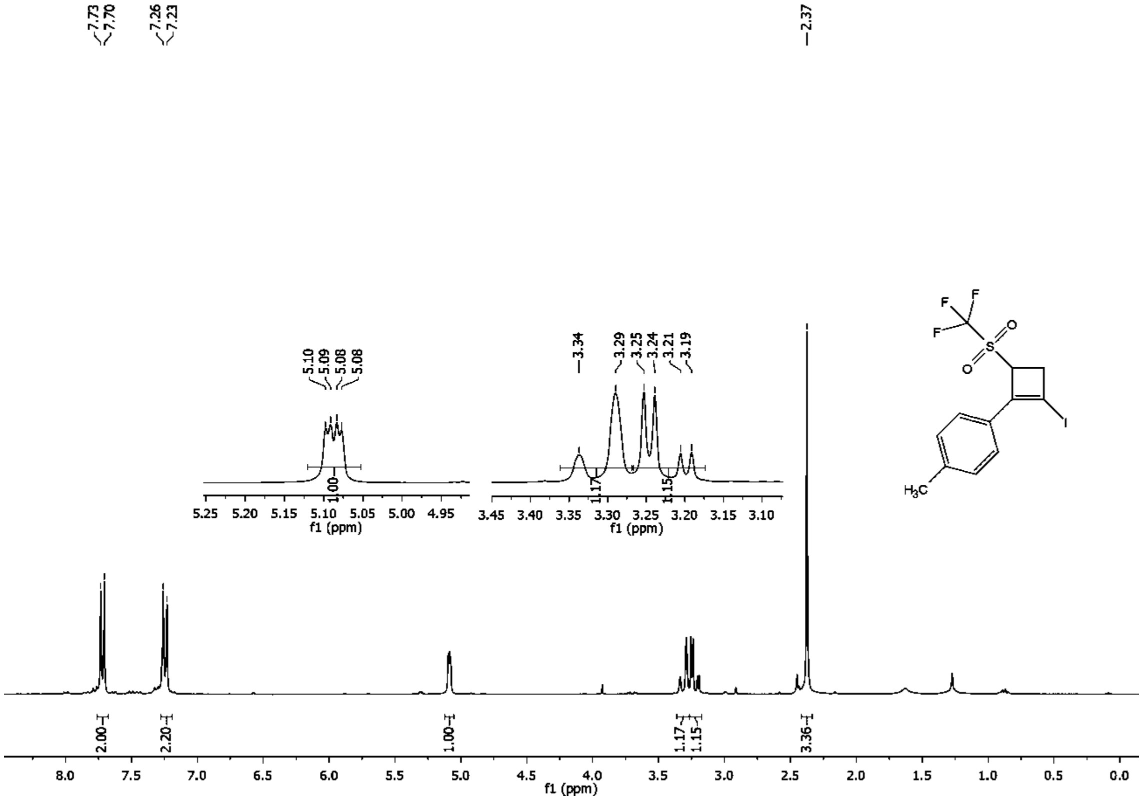

^{13}C NMR compound **3d** ($CDCl_3$, 75 MHz, 25 °C)

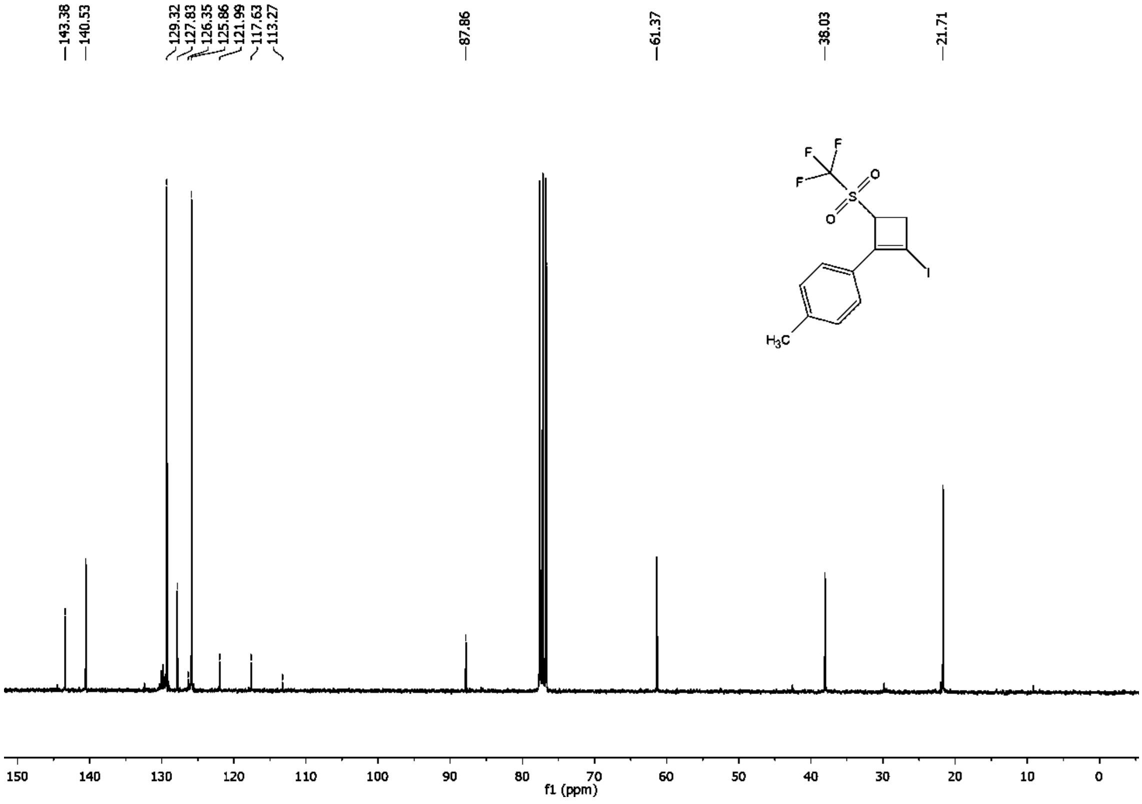

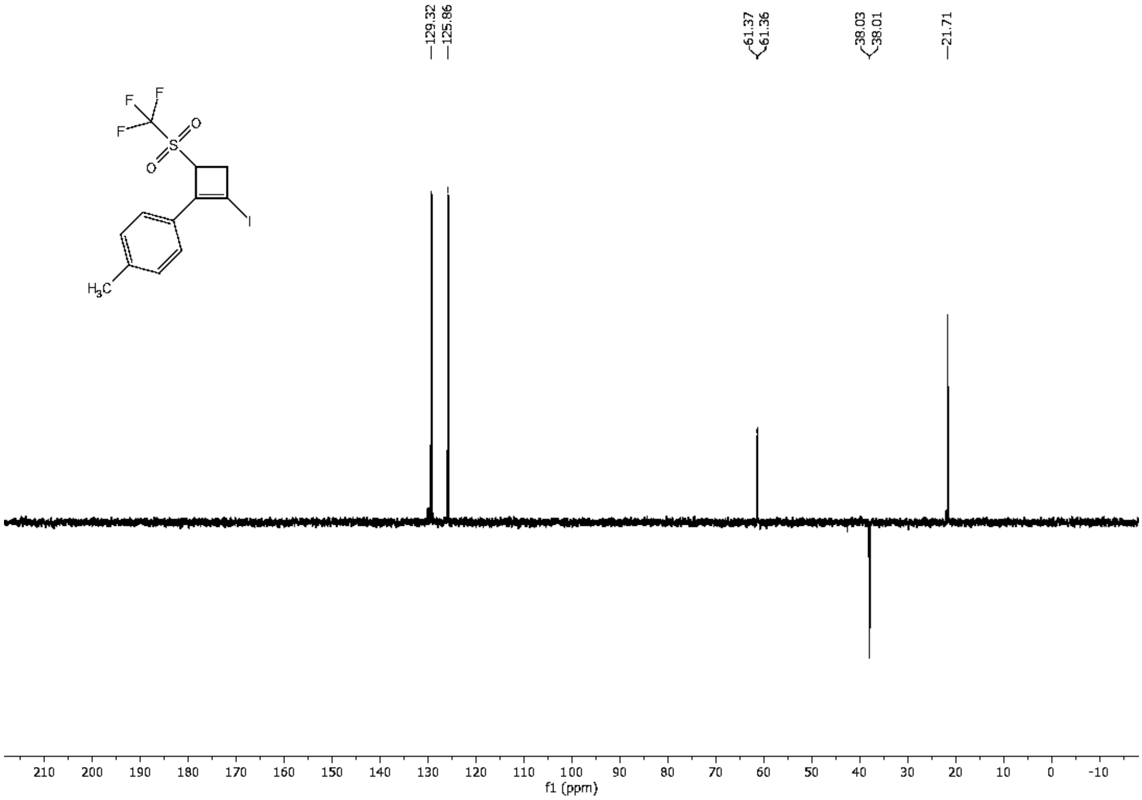

^{19}F NMR compound **3d** ($CDCl_3$, 282 MHz, 25 °C)

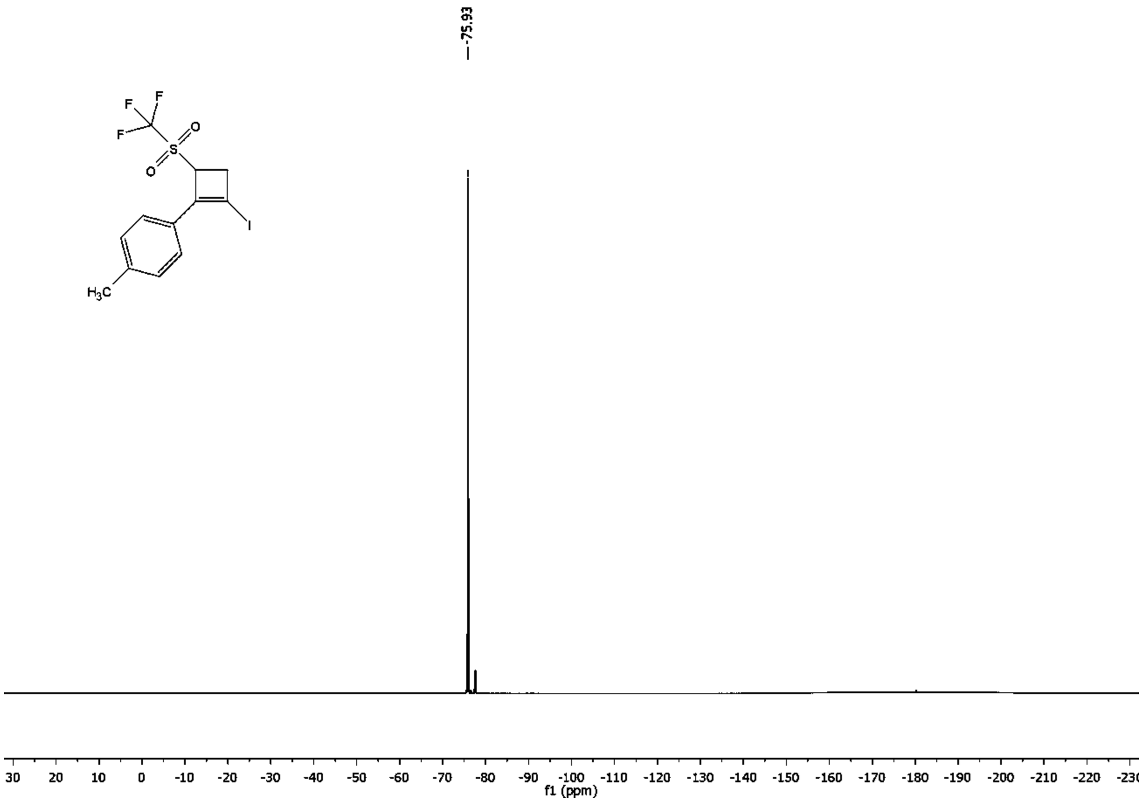

^{1}H NMR compound **3e** ($CDCl_3$, 300 MHz, 25 °C)

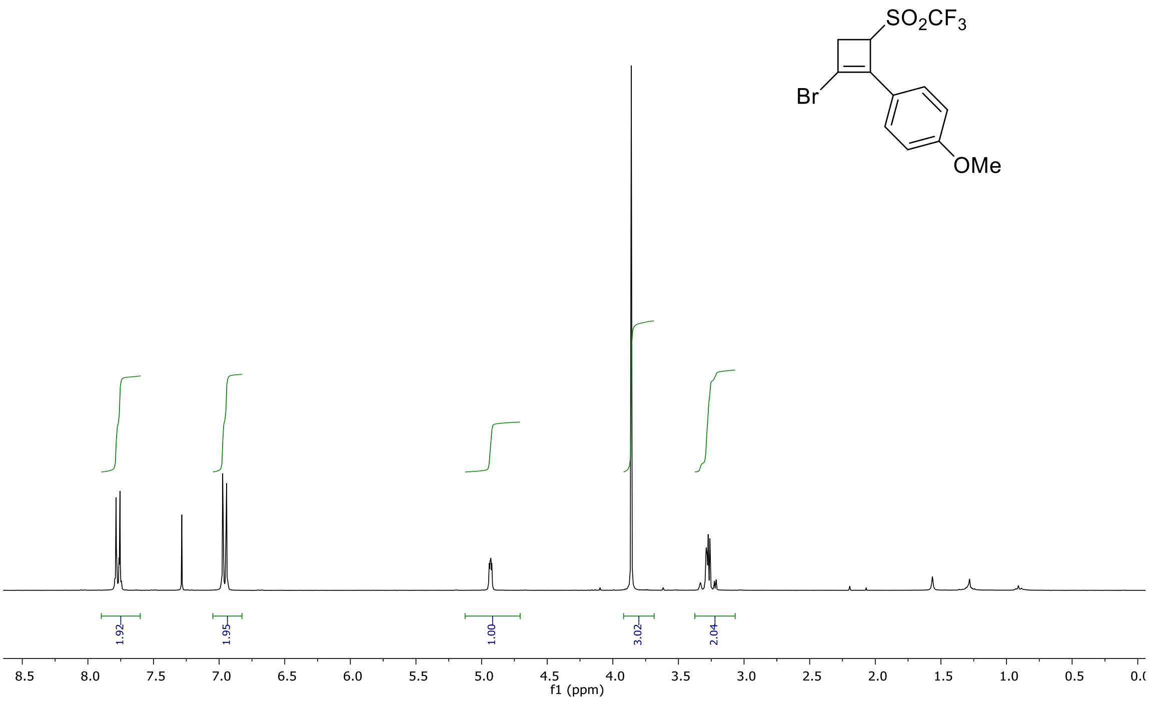

^{13}C NMR compound **3e** ($CDCl_3$, 75 MHz, 25 °C)

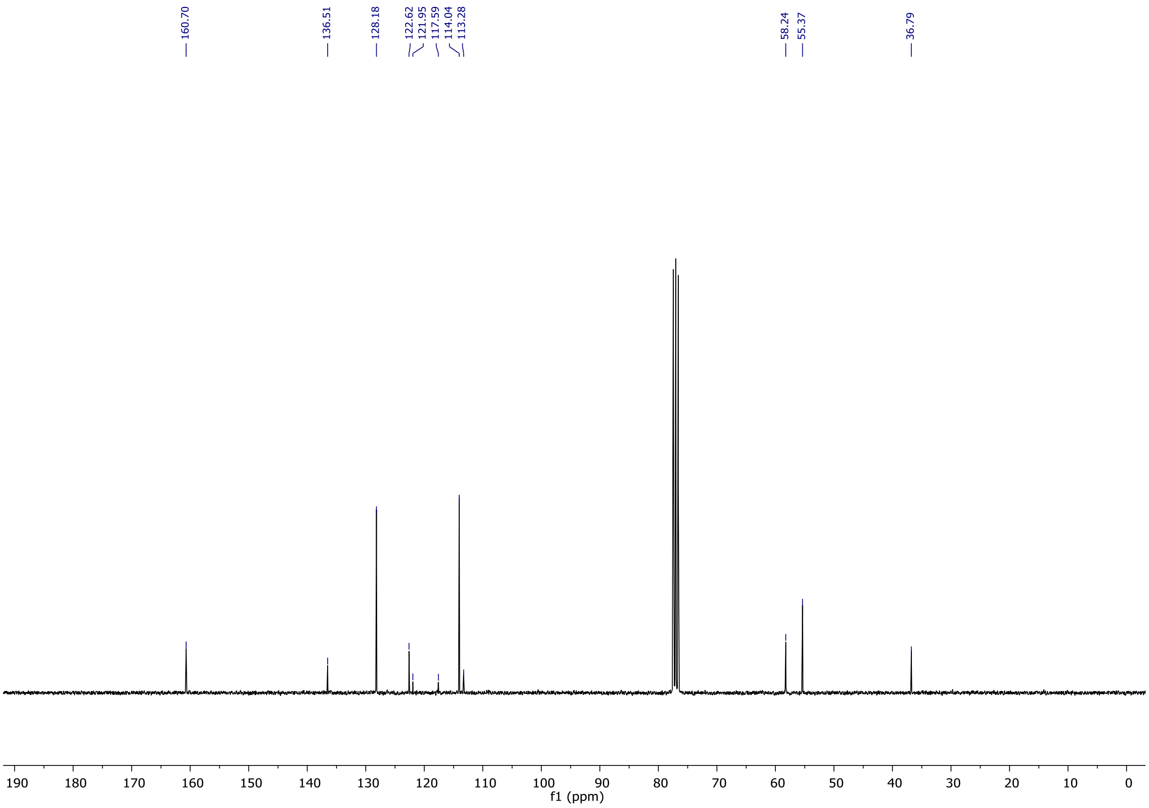

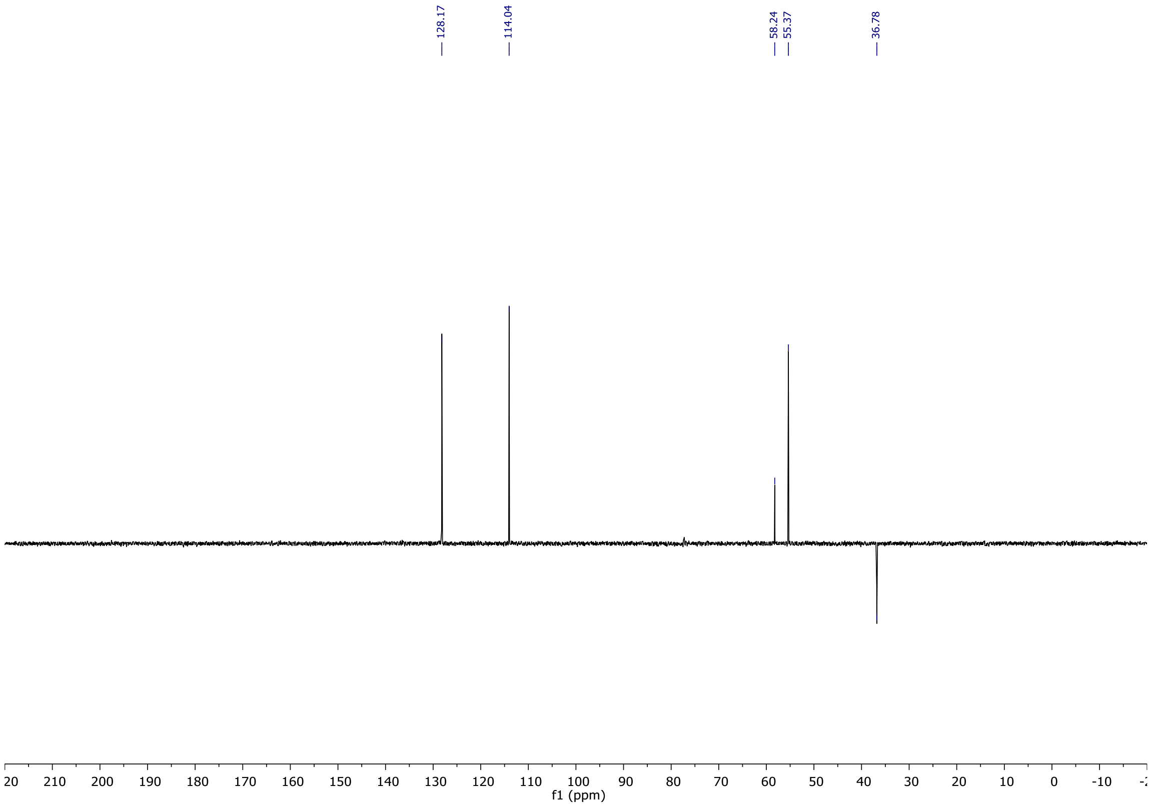

^{19}F NMR compound **3e** ($CDCl_3$, 282 MHz, 25 °C)

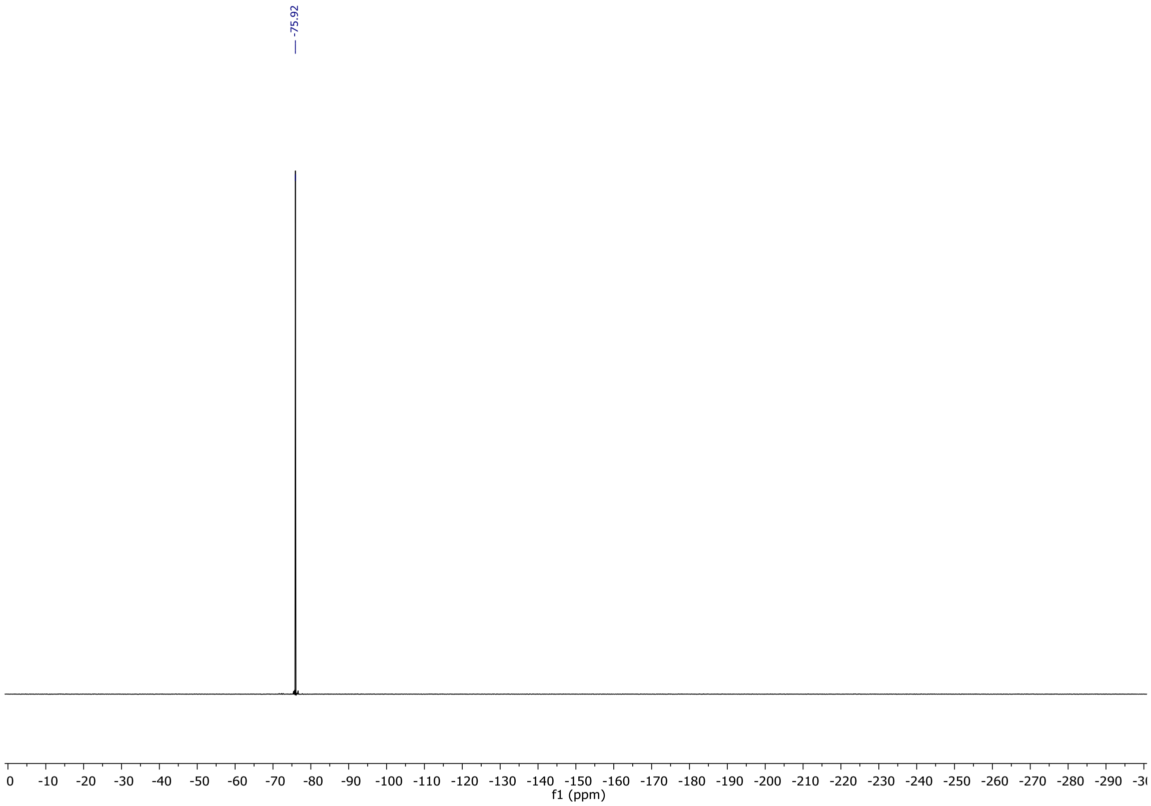

^{1}H NMR compound **3f** ($CDCl_3$, 300 MHz, 25 °C)

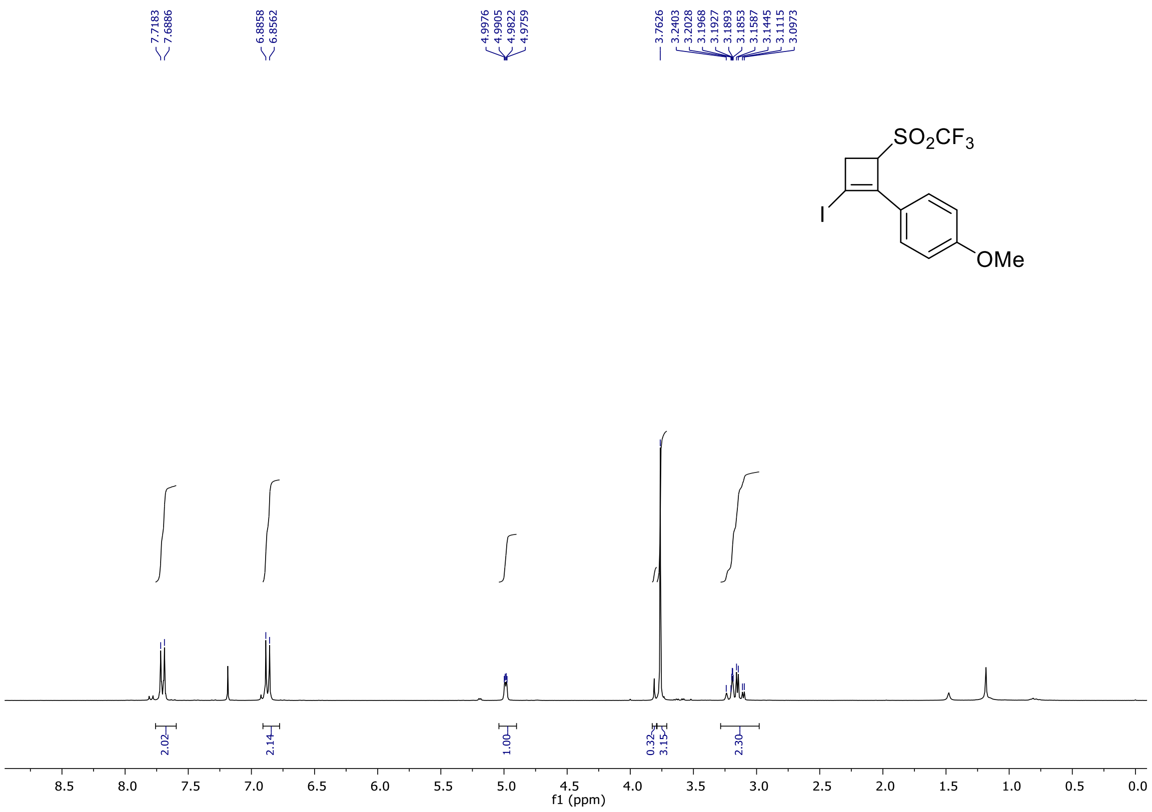

^{13}C NMR compound **3f** ($CDCl_3$, 75 MHz, 25 °C)

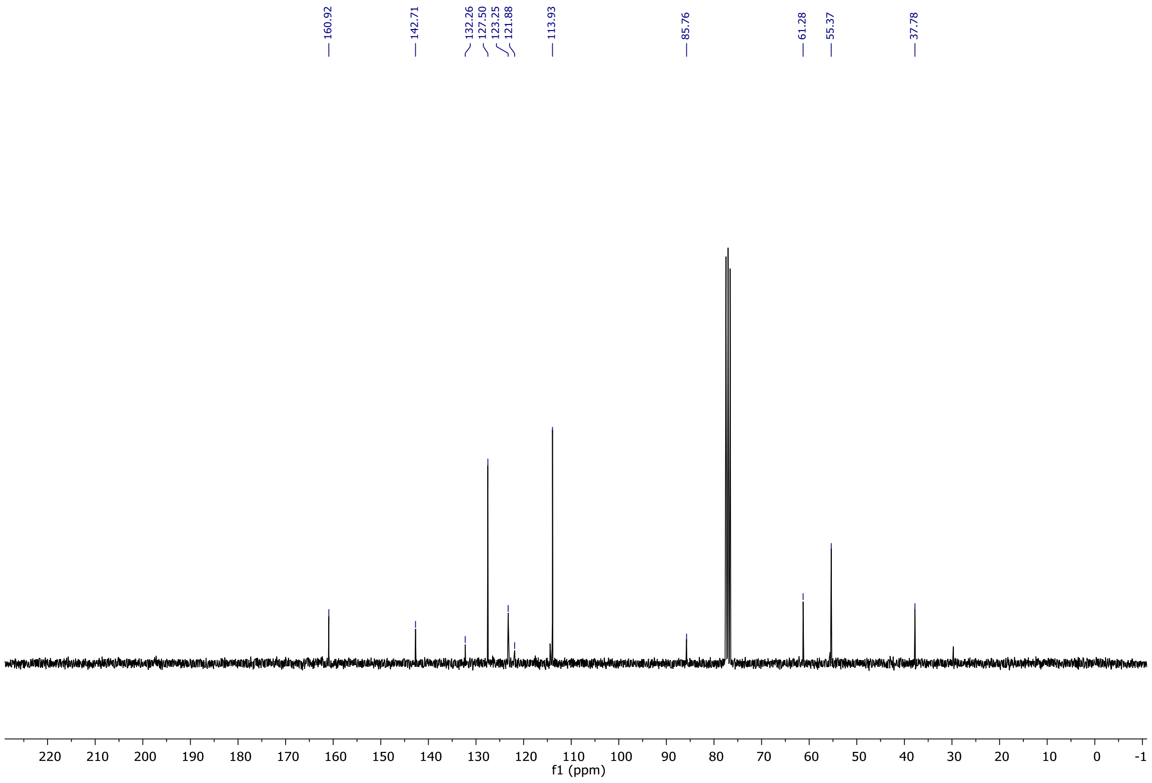

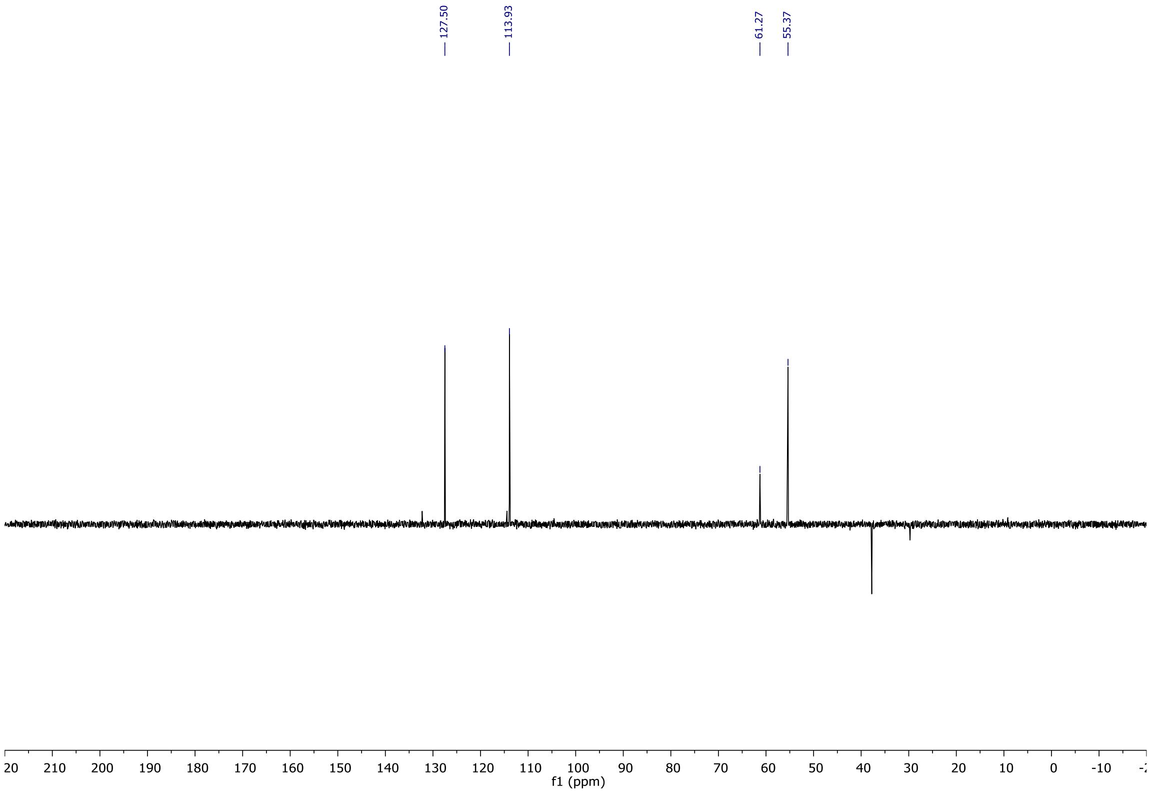

^{19}F NMR compound **3f** ($CDCl_3$, 282 MHz, 25 °C)

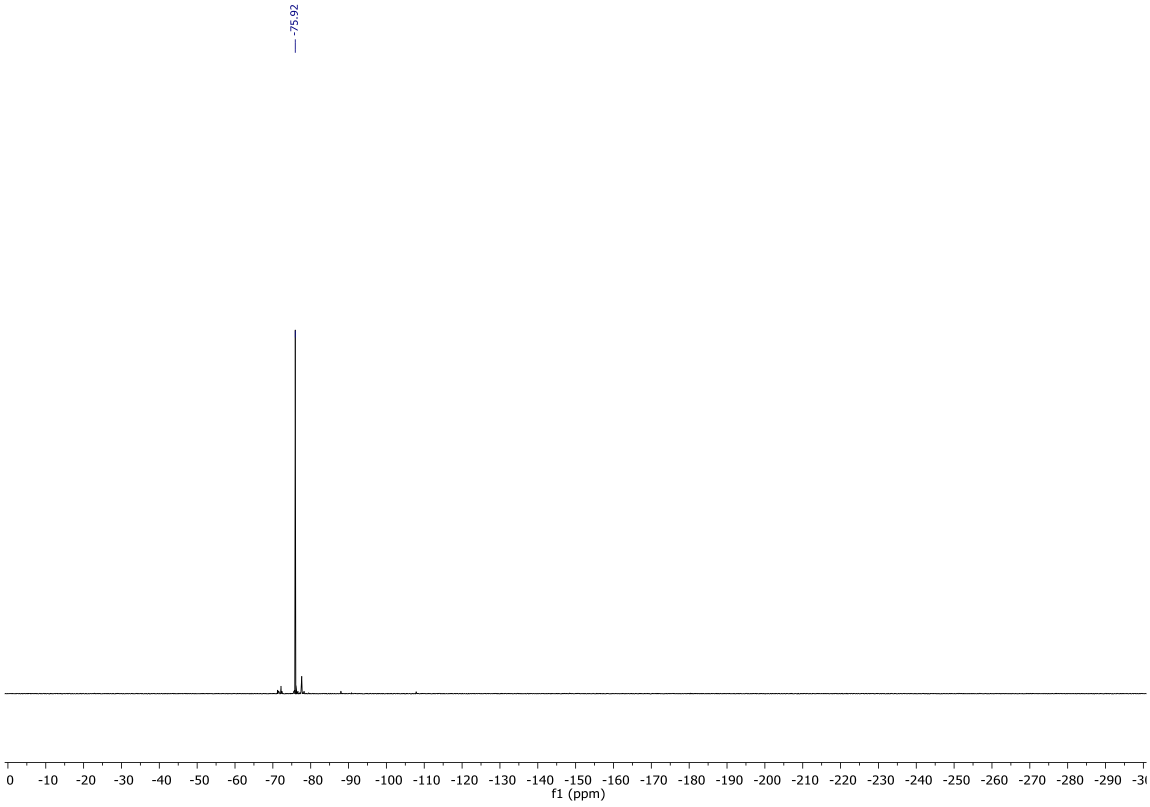

^{1}H NMR compound **3g** ($CDCl_3$, 300 MHz, 25 °C)

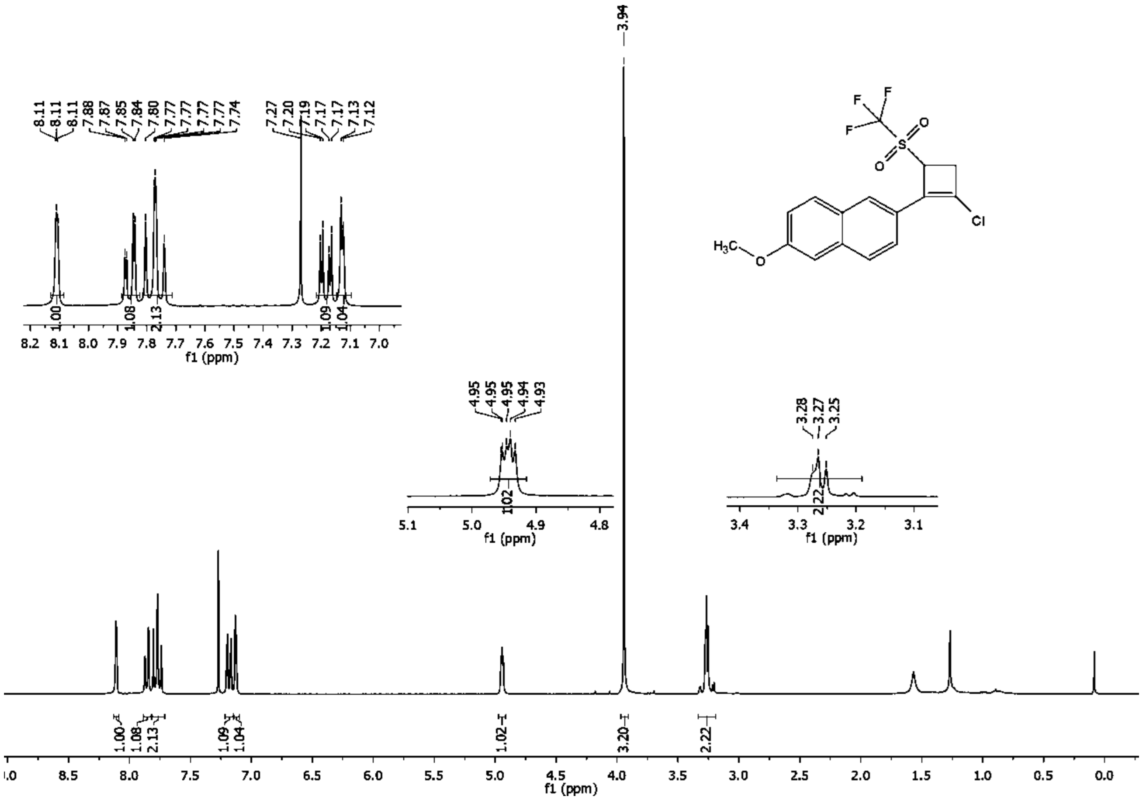

^{13}C NMR compound **3g** ($CDCl_3$, 176 MHz, 25 °C)

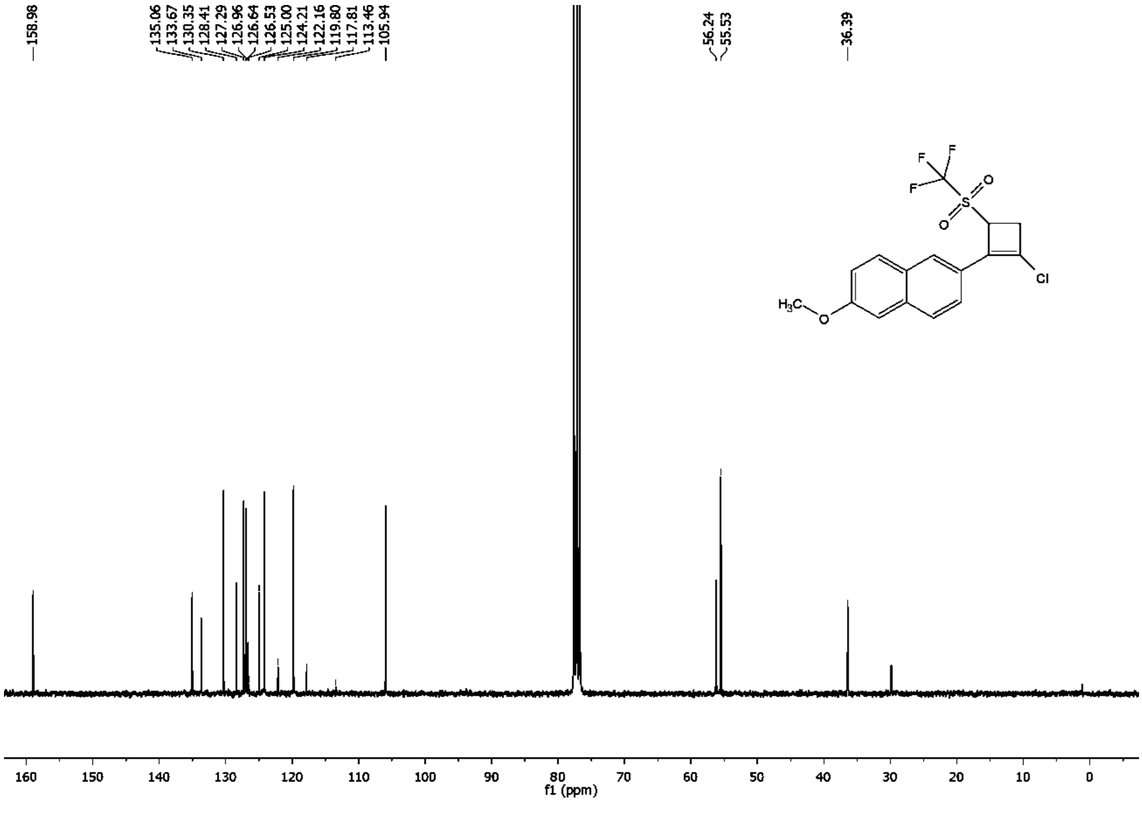

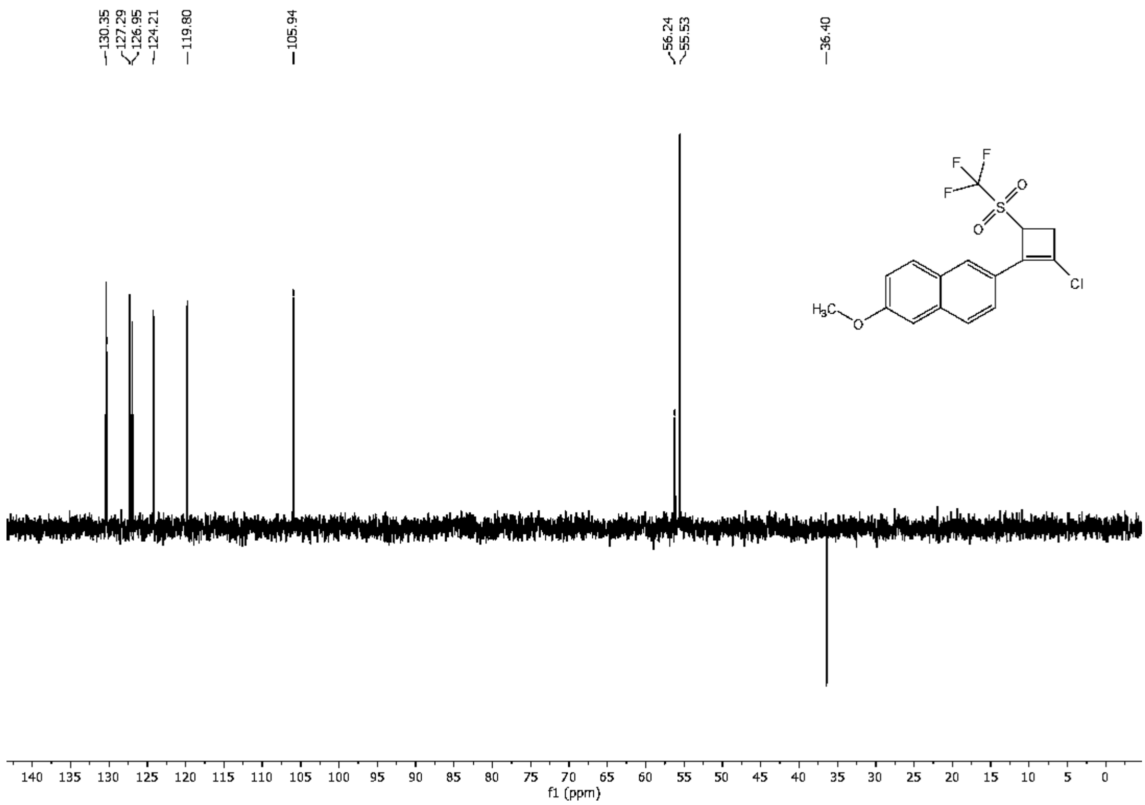

^{19}F NMR compound **3g** ($CDCl_3$, 282 MHz, 25 °C)

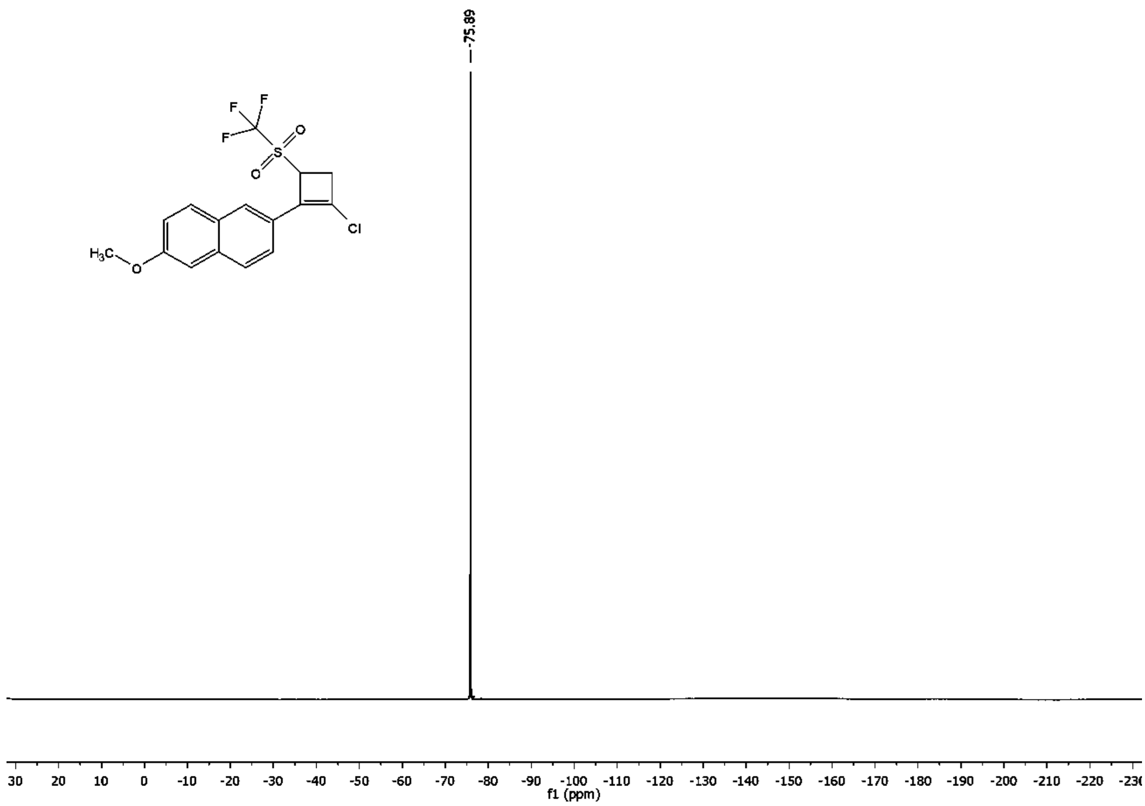

^{1}H NMR compound **3g-d₂** ($CDCl_3$, 300 MHz, 25 °C)

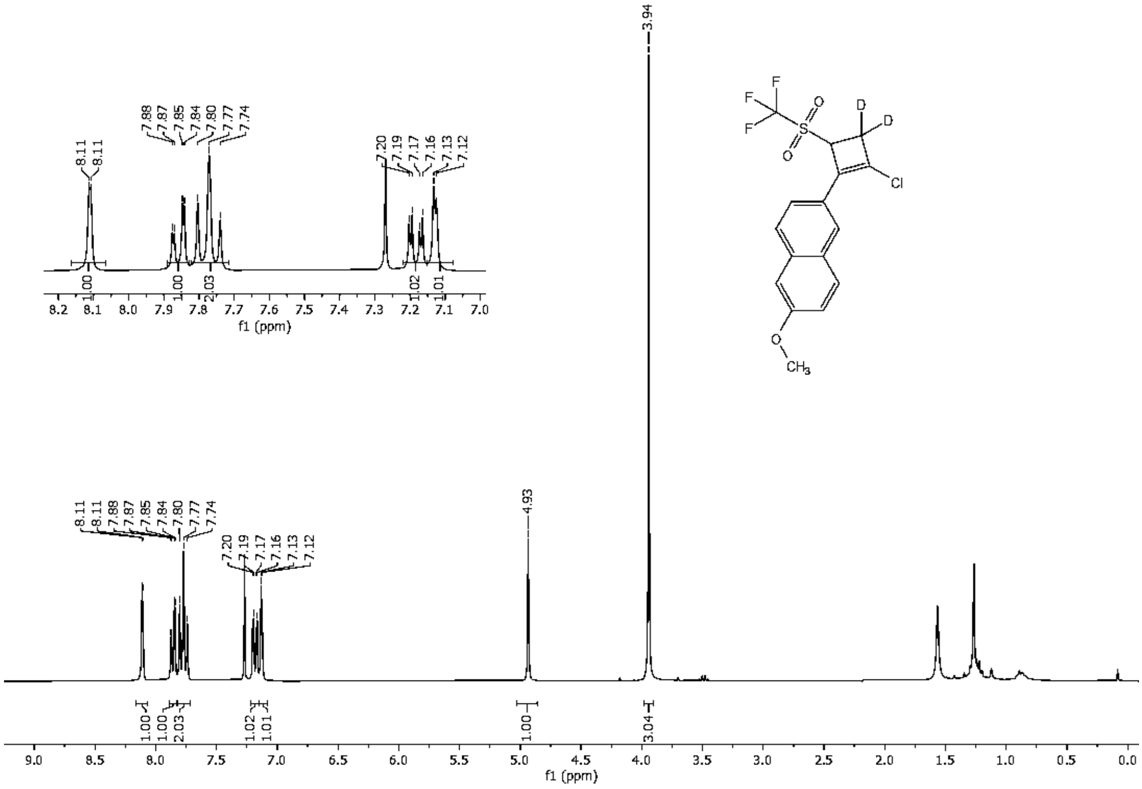

^{13}C NMR compound **3g-d₂** ($CDCl_3$, 176 MHz, 25 °C)

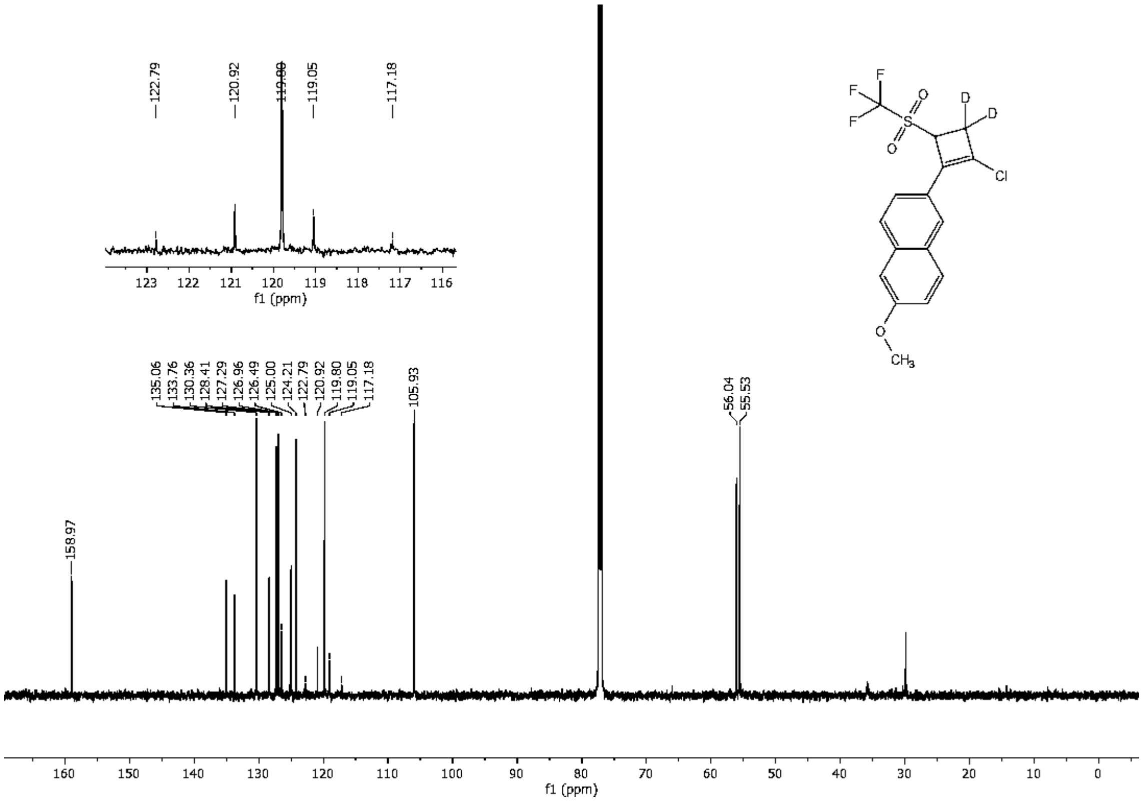

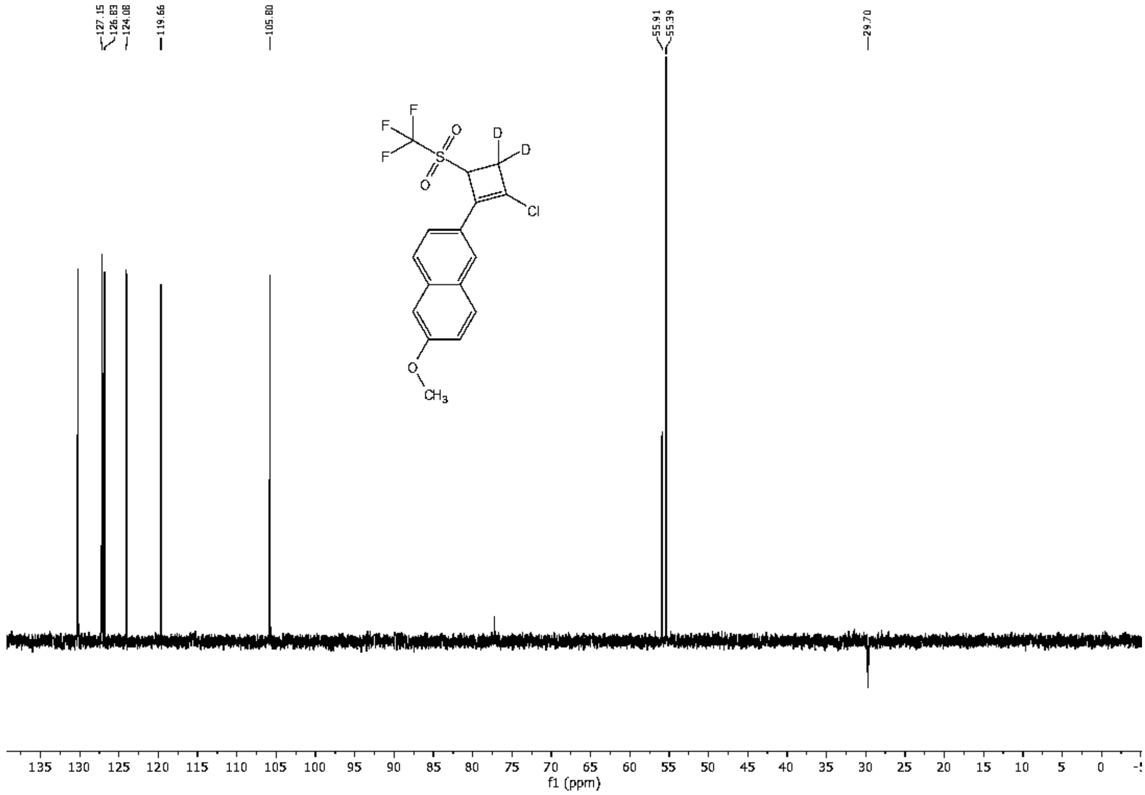

^{19}F NMR compound **3g-d₂** (CDCl$_3$, 282 MHz, 25 ^{o}C)

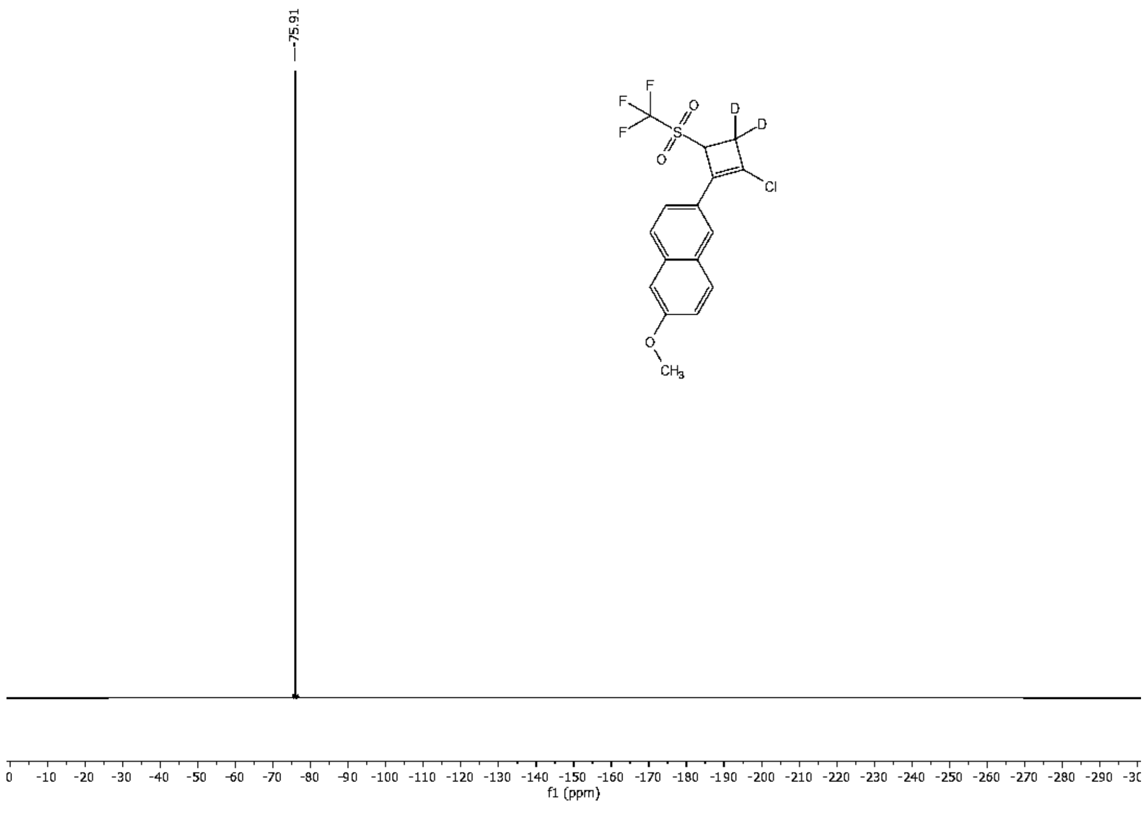

^{2}H NMR compound **3g-d₂** (CDCl$_3$, 107 MHz, 25 ^{o}C)

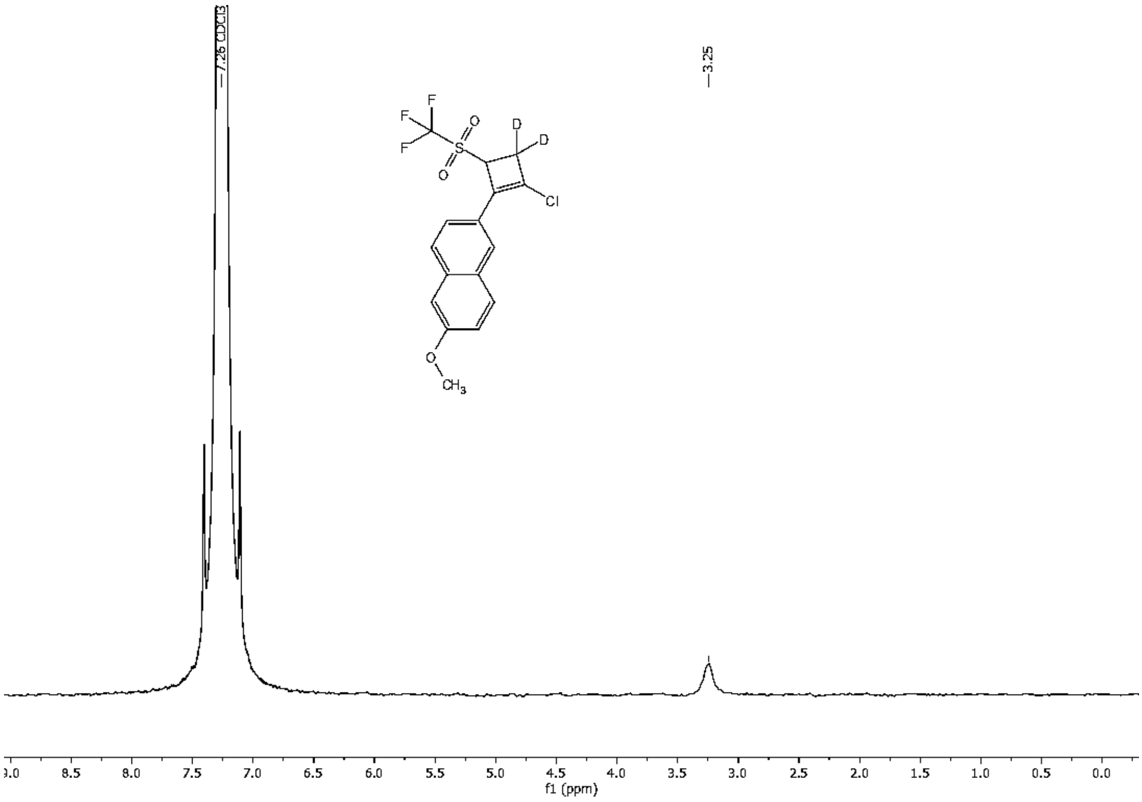

^{1}H NMR compound **3h** ($CDCl_3$, 300 MHz, 25 °C)

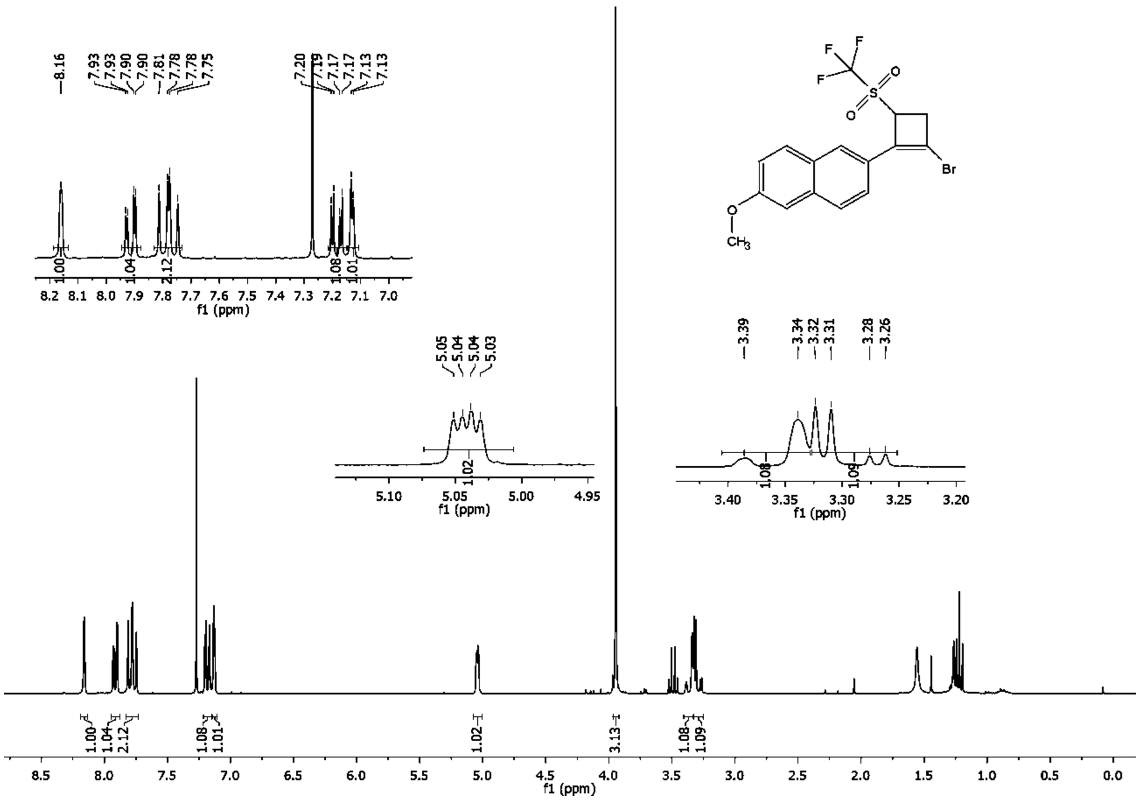

^{13}C NMR compound **3h** ($CDCl_3$, 176 MHz, 25 °C)

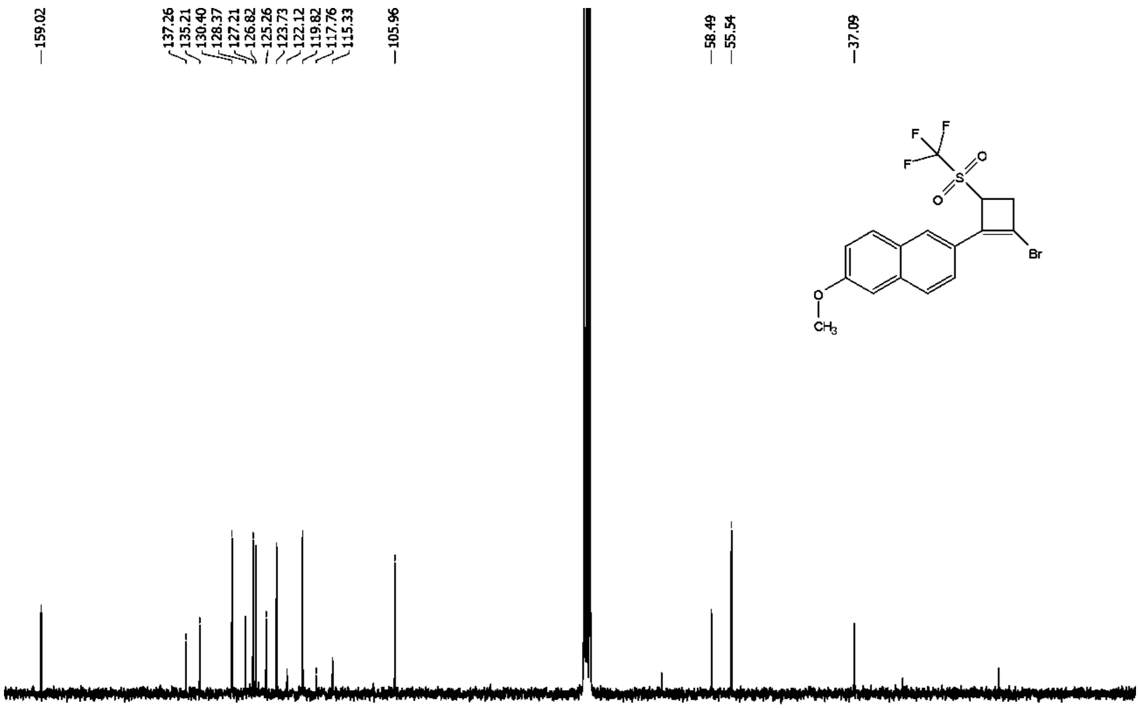

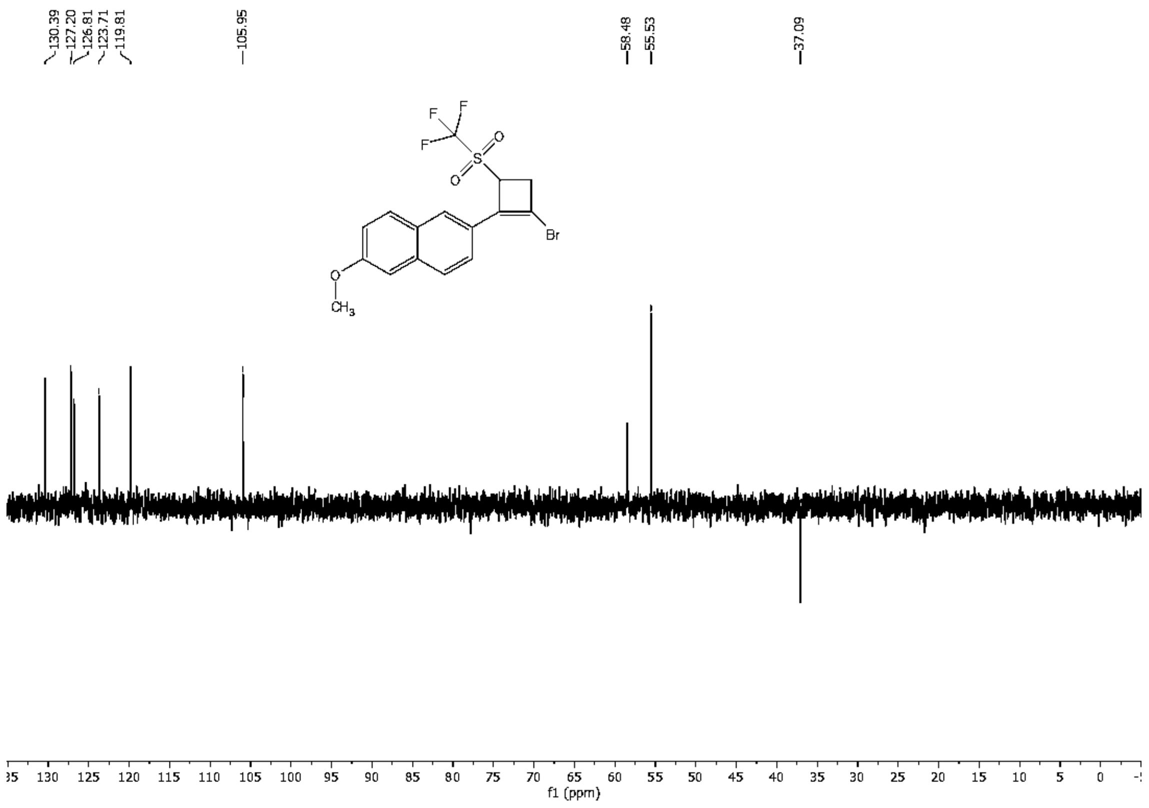

^{19}F NMR compound **3h** ($CDCl_3$, 282 MHz, 25 °C)

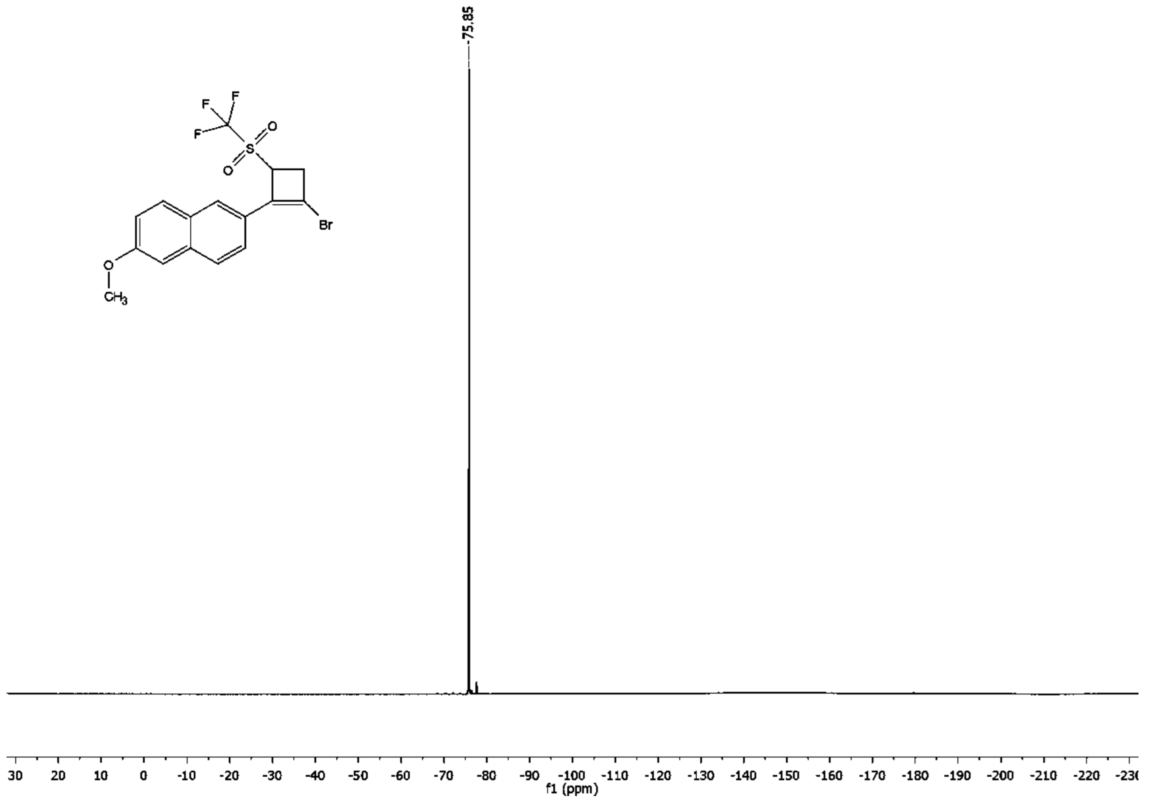

^{1}H NMR compound **3h-d$_2$** ($CDCl_3$, 300 MHz, 25 °C)

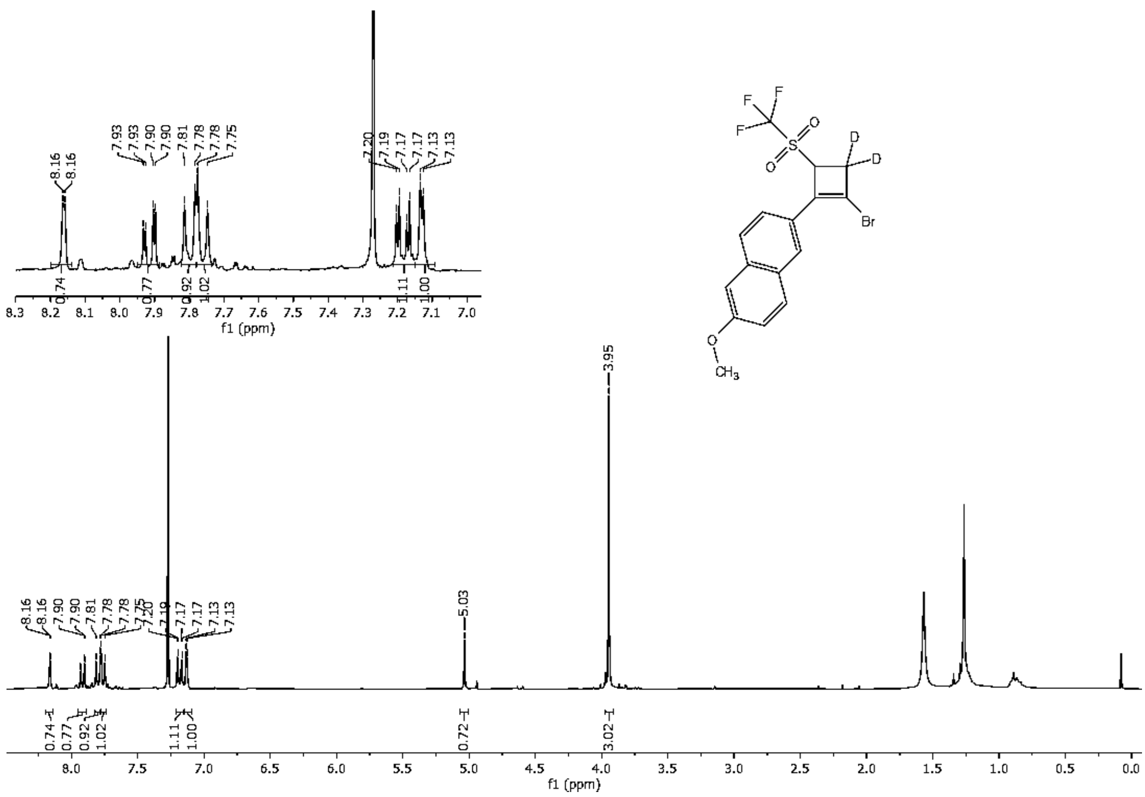

^{13}C NMR compound **3h-d$_2$** ($CDCl_3$, 75 MHz, 25 °C)

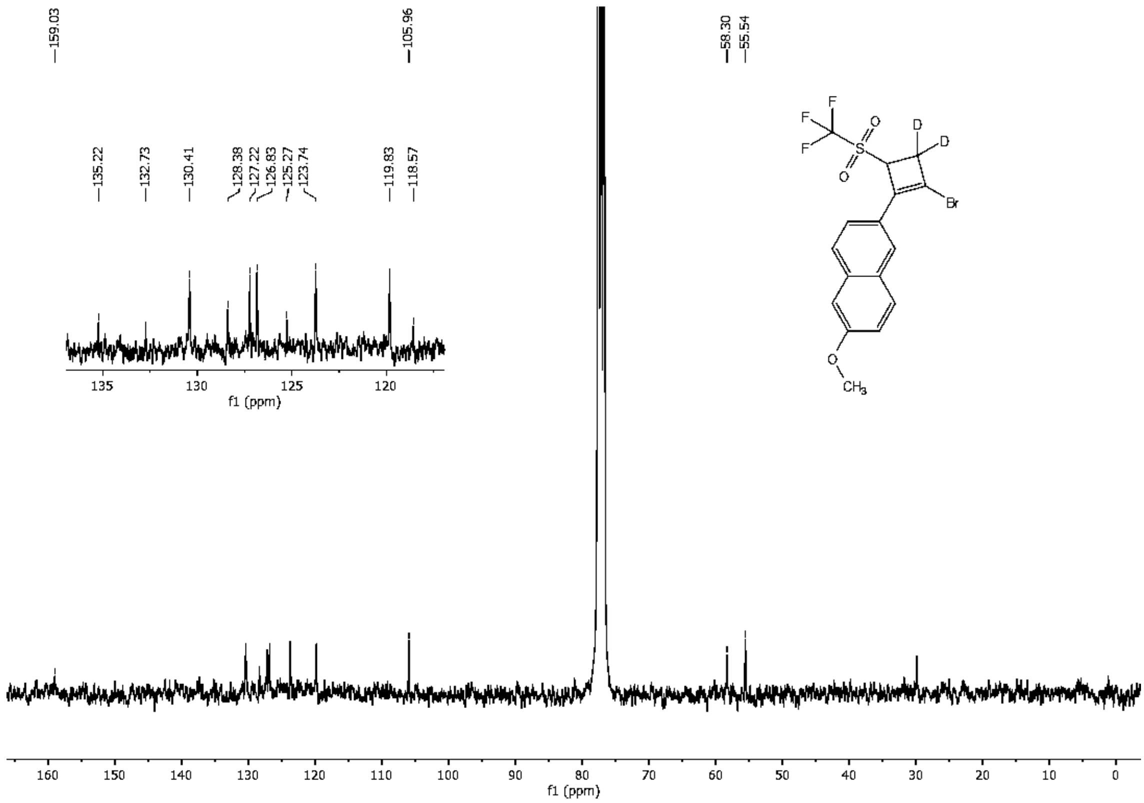

^{19}F NMR compound **3h-d$_2$** ($CDCl_3$, 282 MHz, 25 °C)

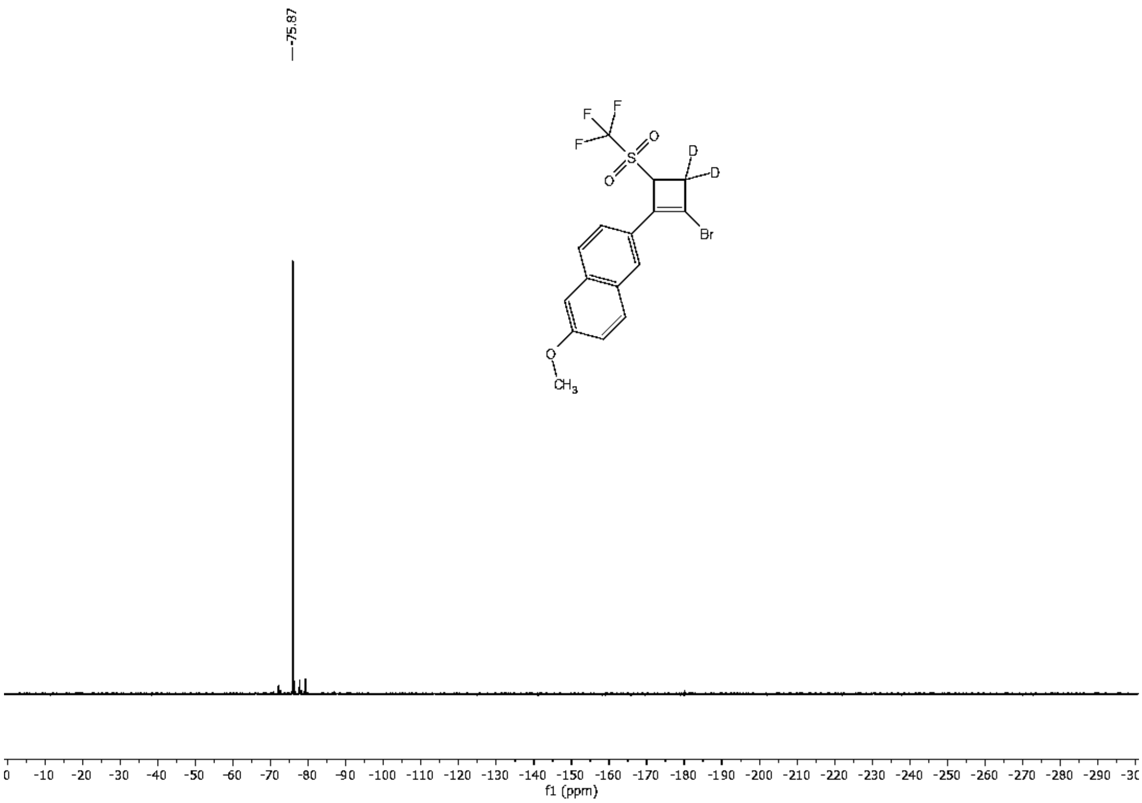

^{1}H NMR compound **3i** ($CDCl_3$, 300 MHz, 25 °C)

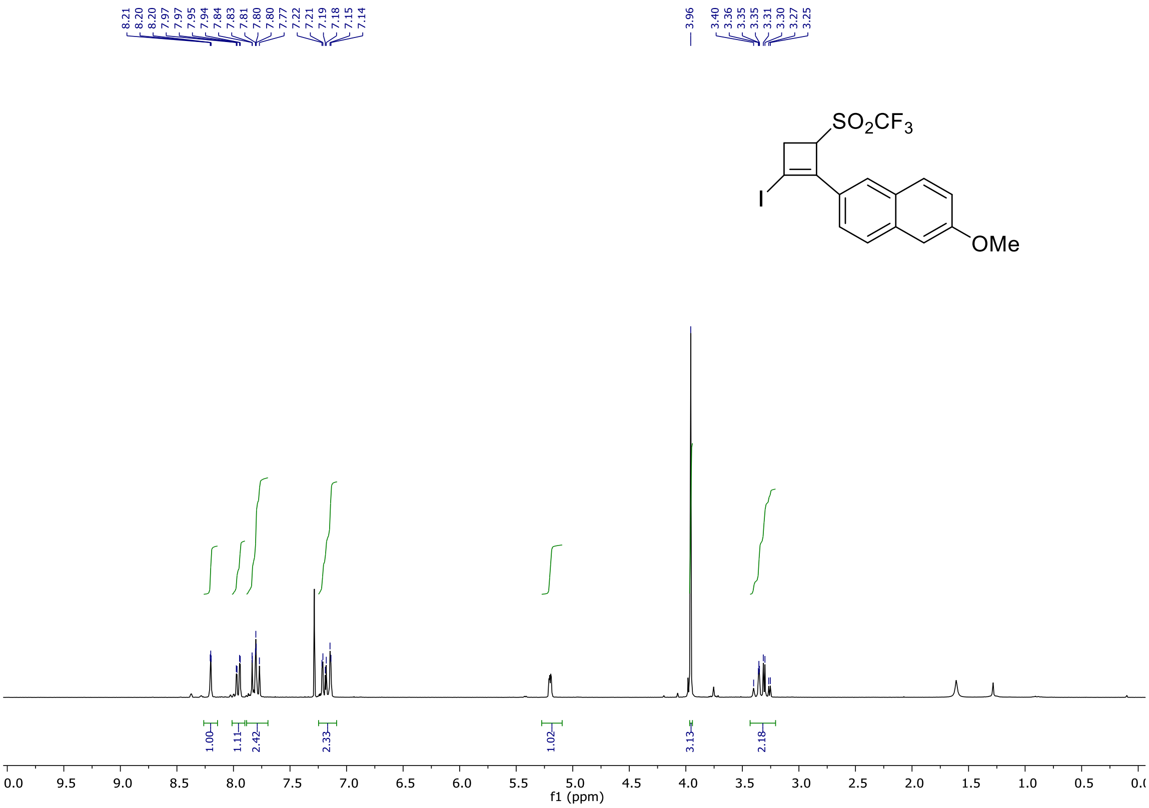

^{13}C NMR compound **3i** ($CDCl_3$, 75 MHz, 25 °C)

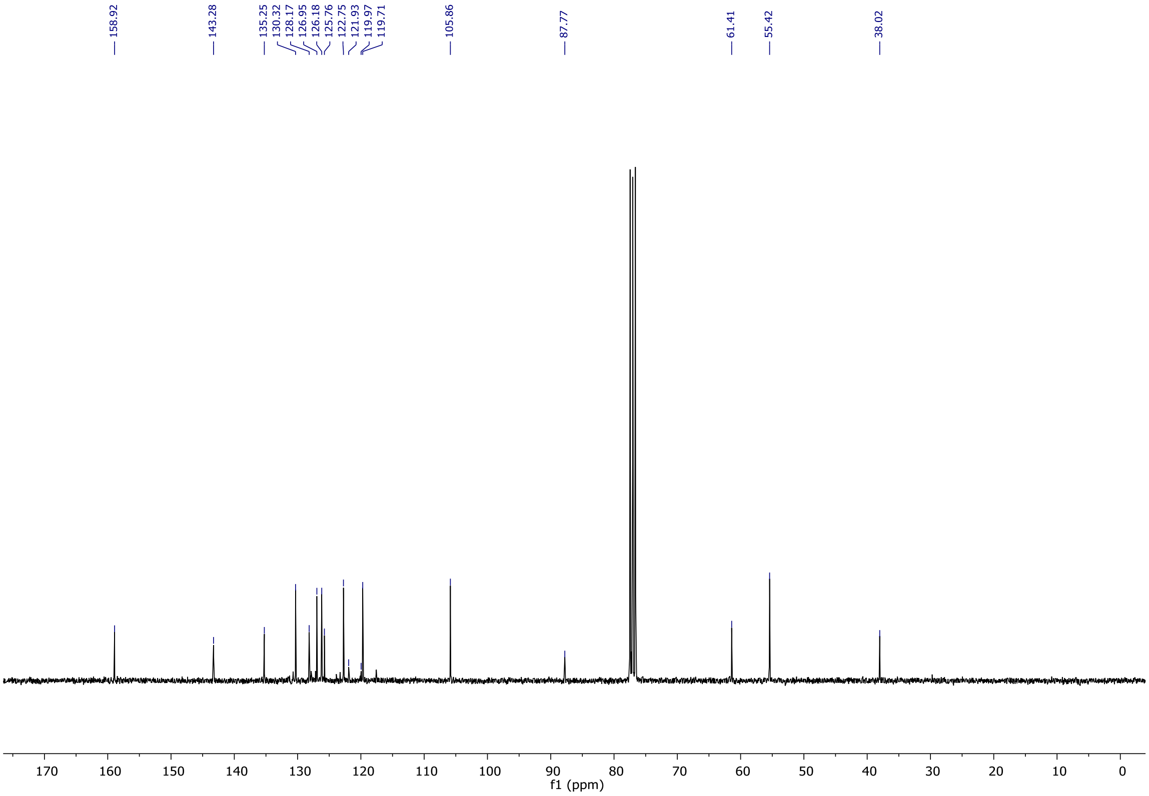

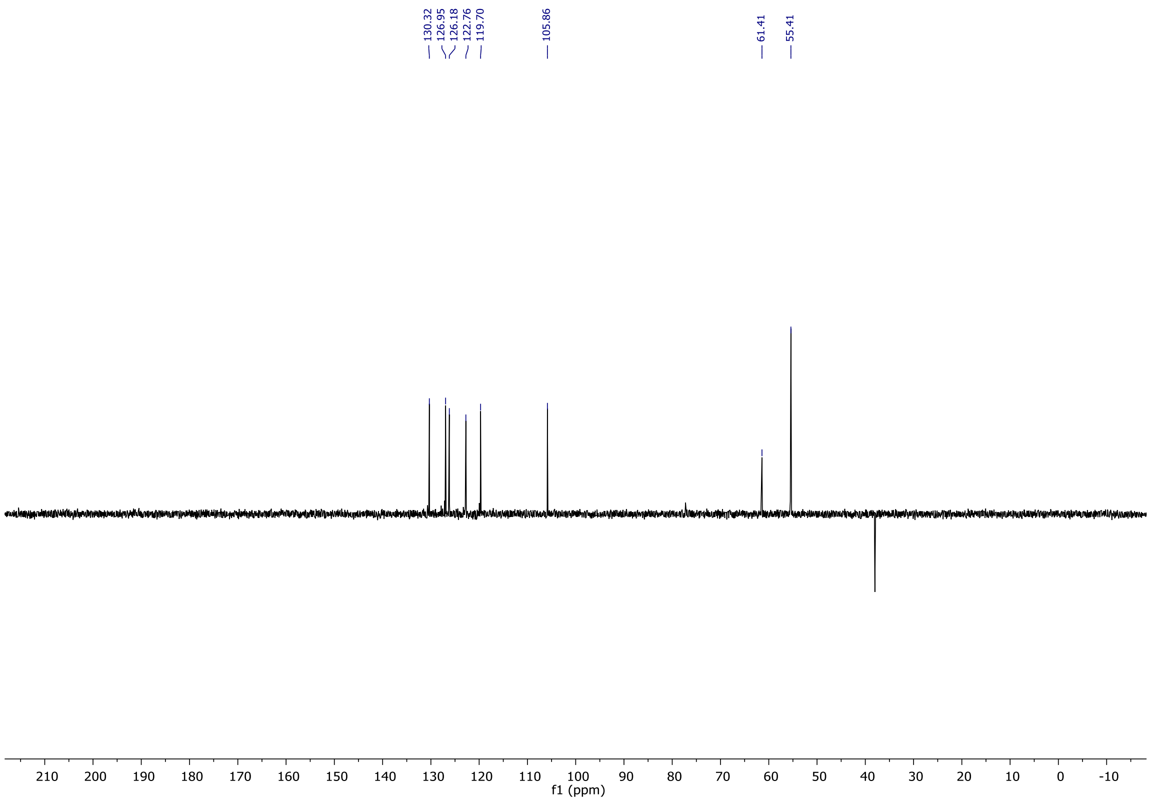
130.32
126.95
126.18
122.76
119.70
105.86
61.41
55.41
210 200 190 180 170 160 150 140 130 120 110 100 90 80 70 60 50 40 30 20 10 0 -10
f1 (ppm)

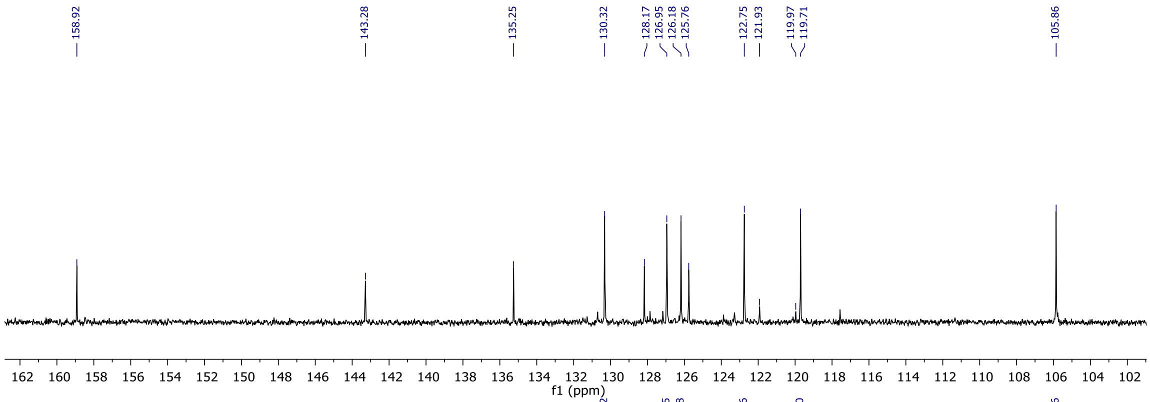
158.92
143.28
135.25
130.32
128.17
126.95
126.18
125.76
122.75
121.93
119.97
119.71
105.86
162 160 158 156 154 152 150 148 146 144 142 140 138 136 134 132 130 128 126 124 122 120 118 116 114 112 110 108 106 104 102
f1 (ppm)

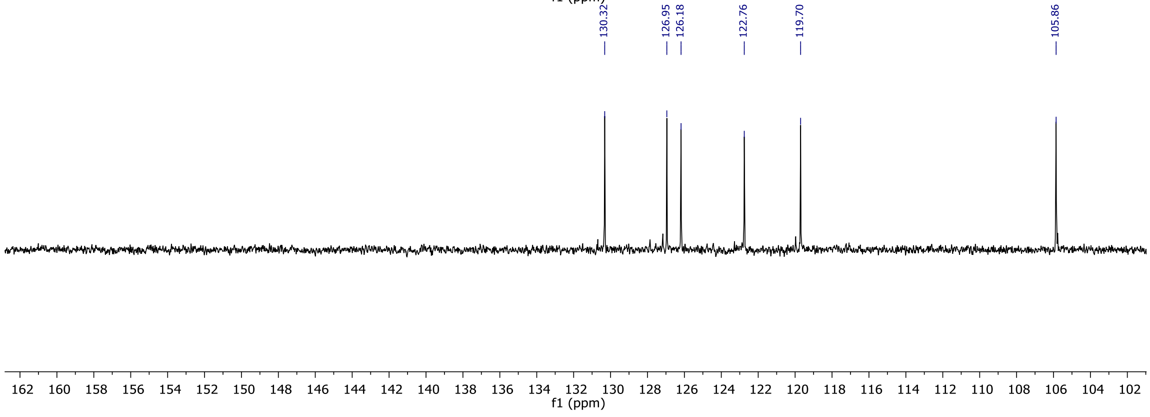
130.32
126.95
126.18
122.76
119.70
105.86
162 160 158 156 154 152 150 148 146 144 142 140 138 136 134 132 130 128 126 124 122 120 118 116 114 112 110 108 106 104 102
f1 (ppm)

^{1}H NMR compound **3j** ($CDCl_3$, 300 MHz, 25 °C)

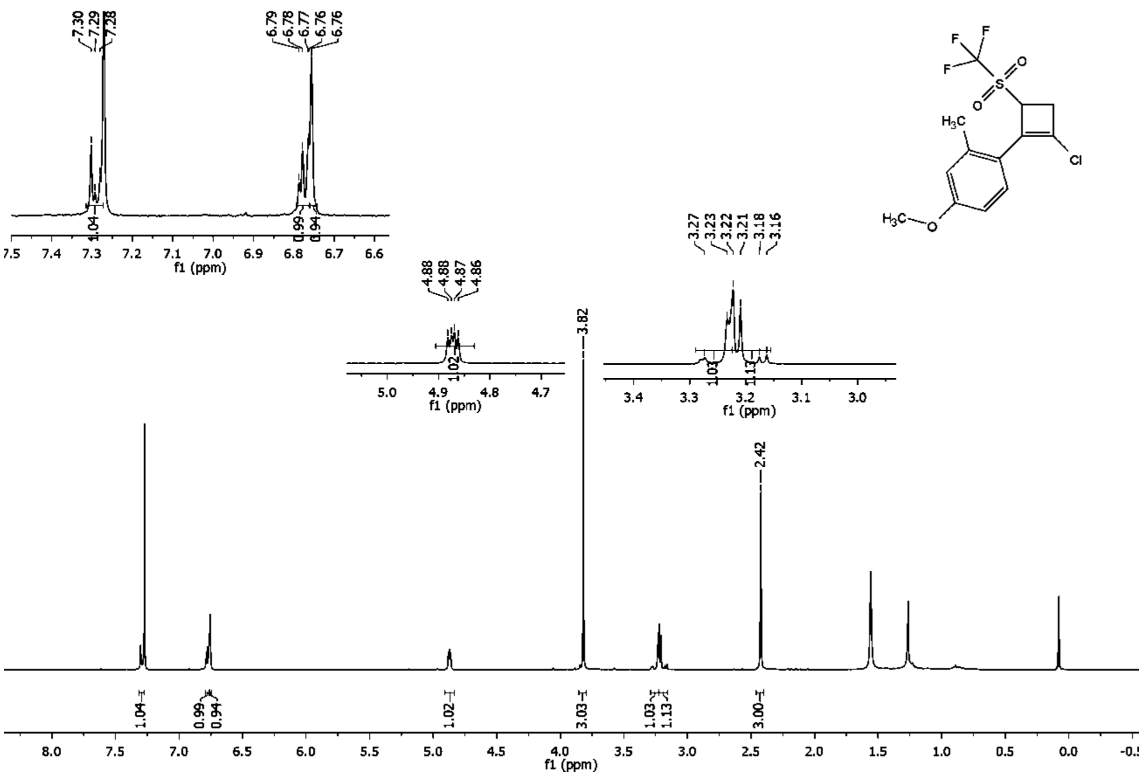

^{13}C NMR compound **3j** ($CDCl_3$, 75 MHz, 25 °C)

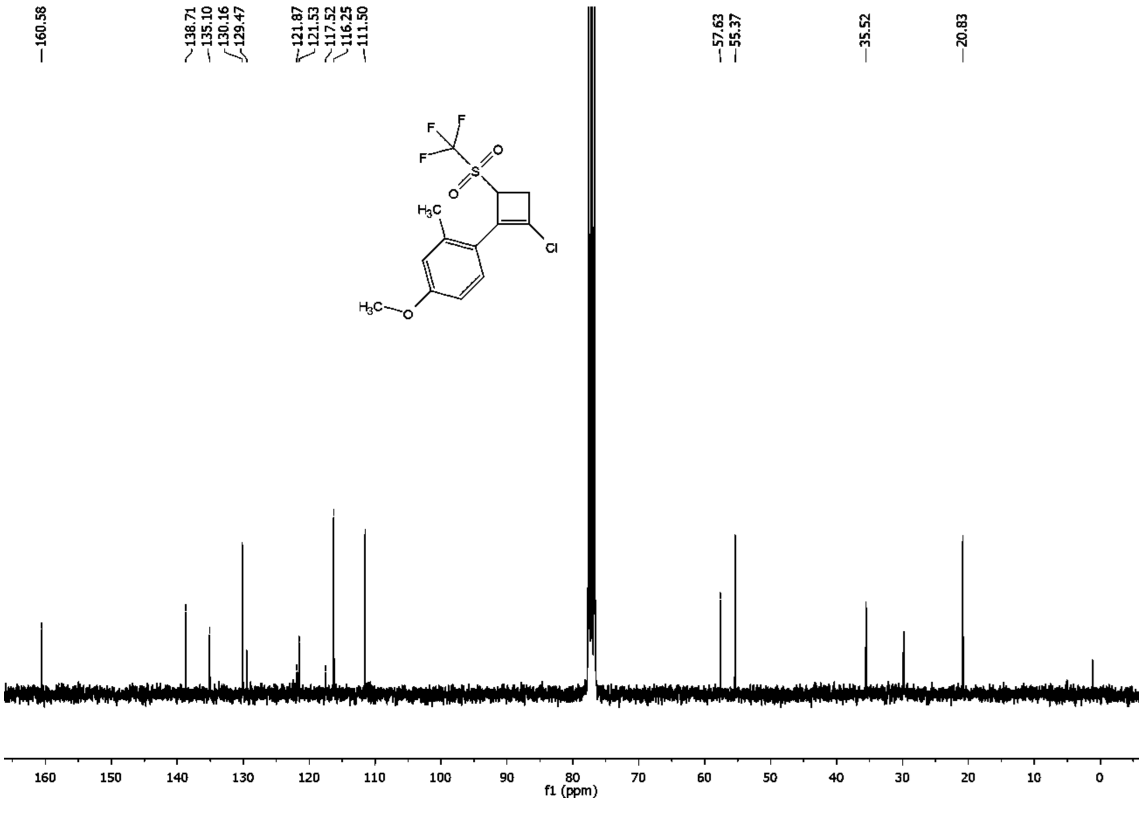

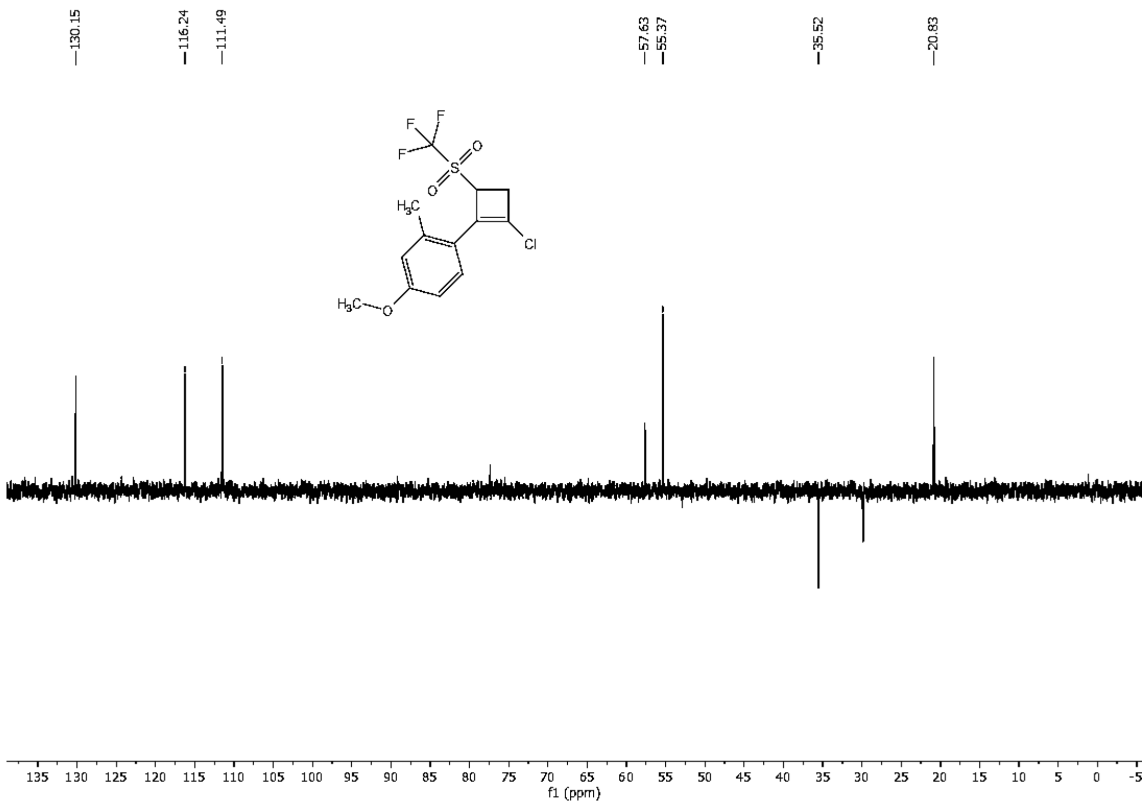

^{19}F NMR compound **3j** ($CDCl_3$, 282 MHz, 25 °C)

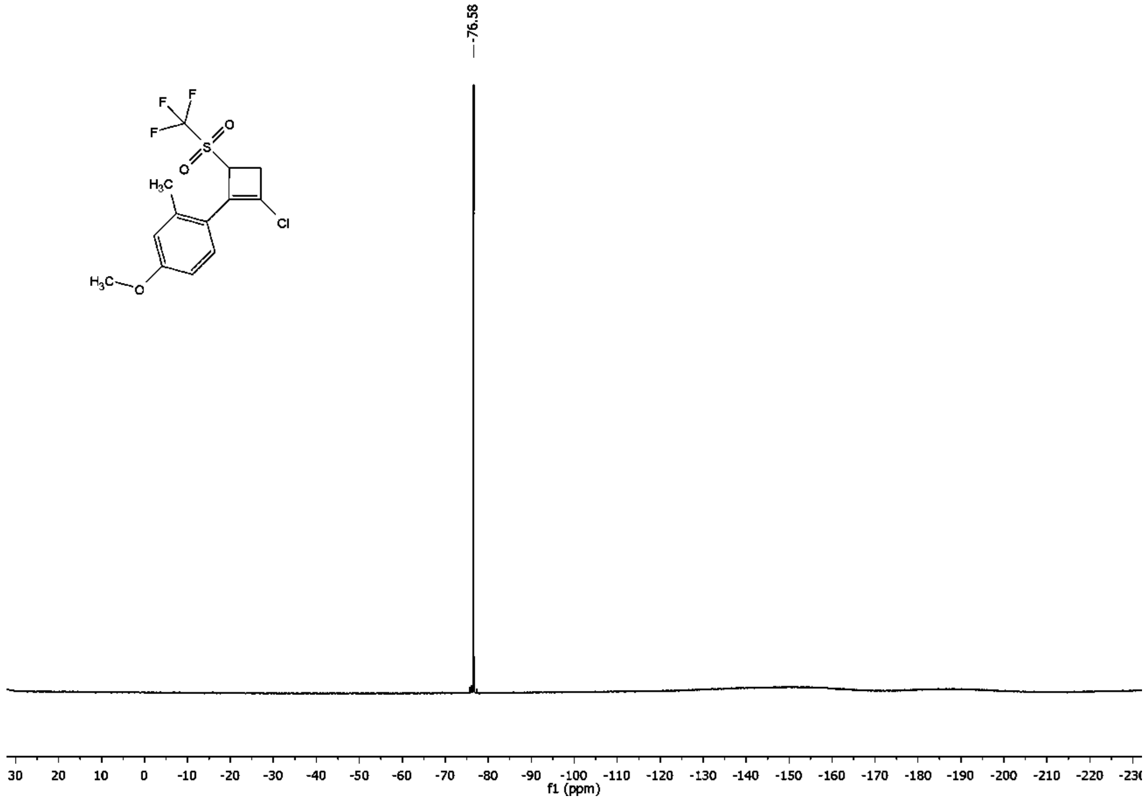

^{1}H NMR compound **3j-d₂** (CDCl$_3$, 300 MHz, 25 °C)

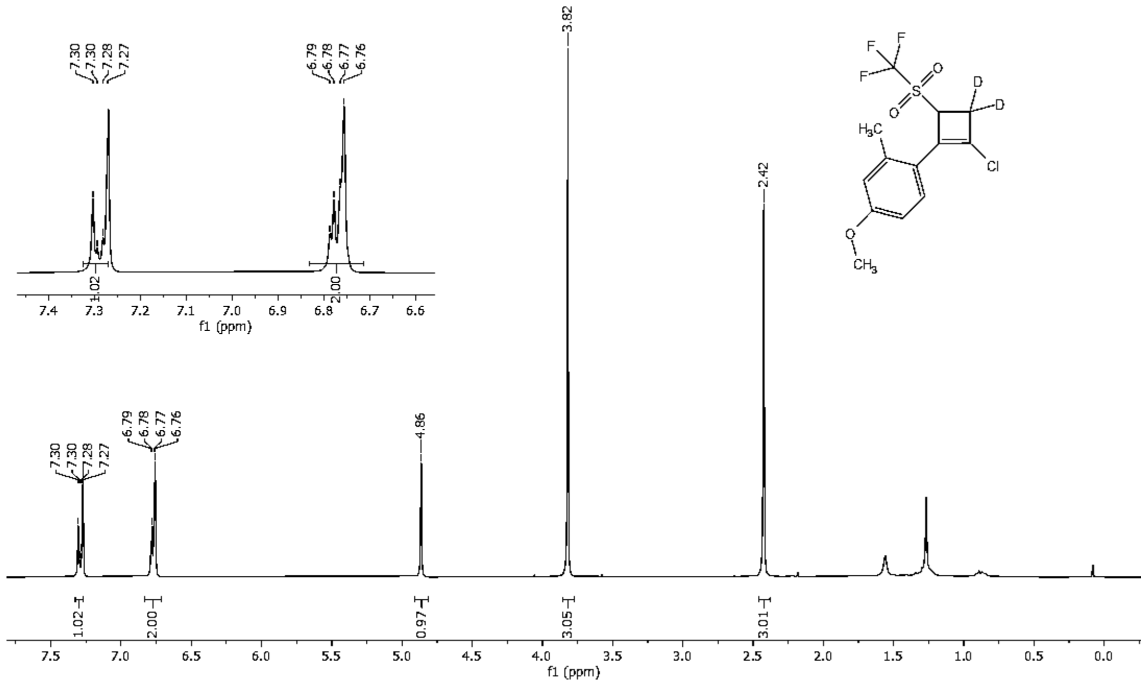

^{13}C NMR compound **3j-d₂** (CDCl$_3$, 75 MHz, 25 °C)

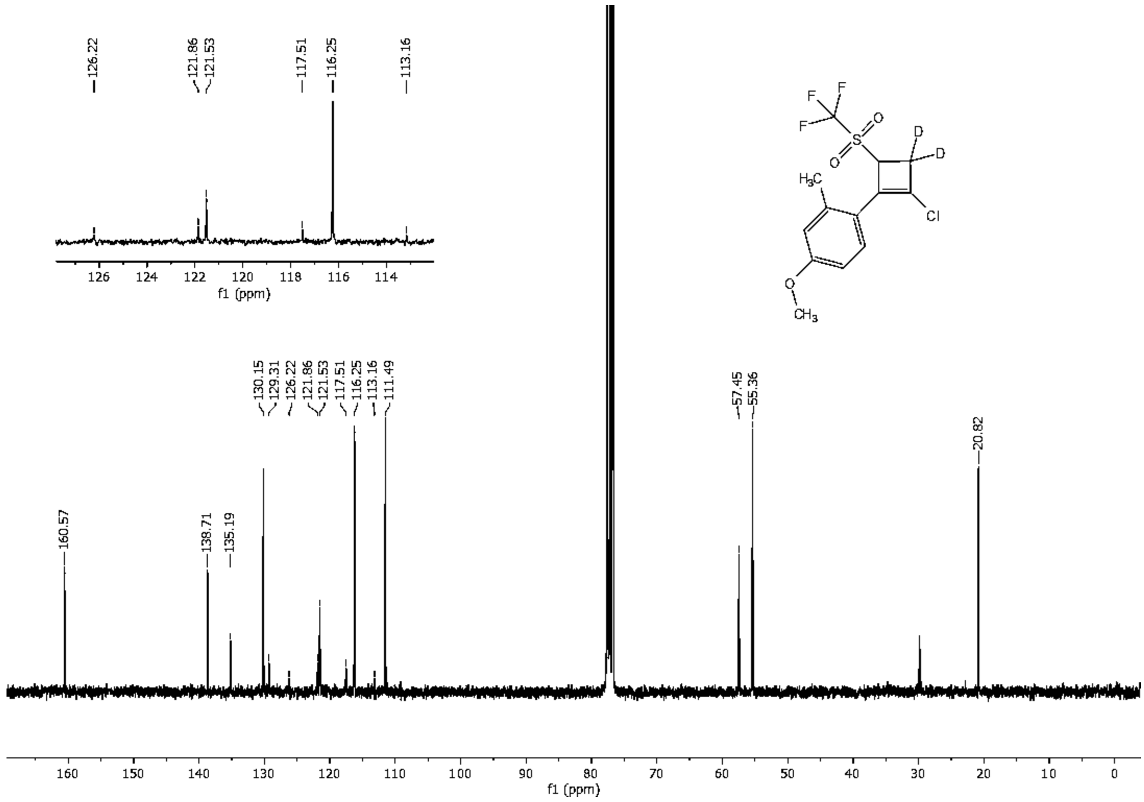

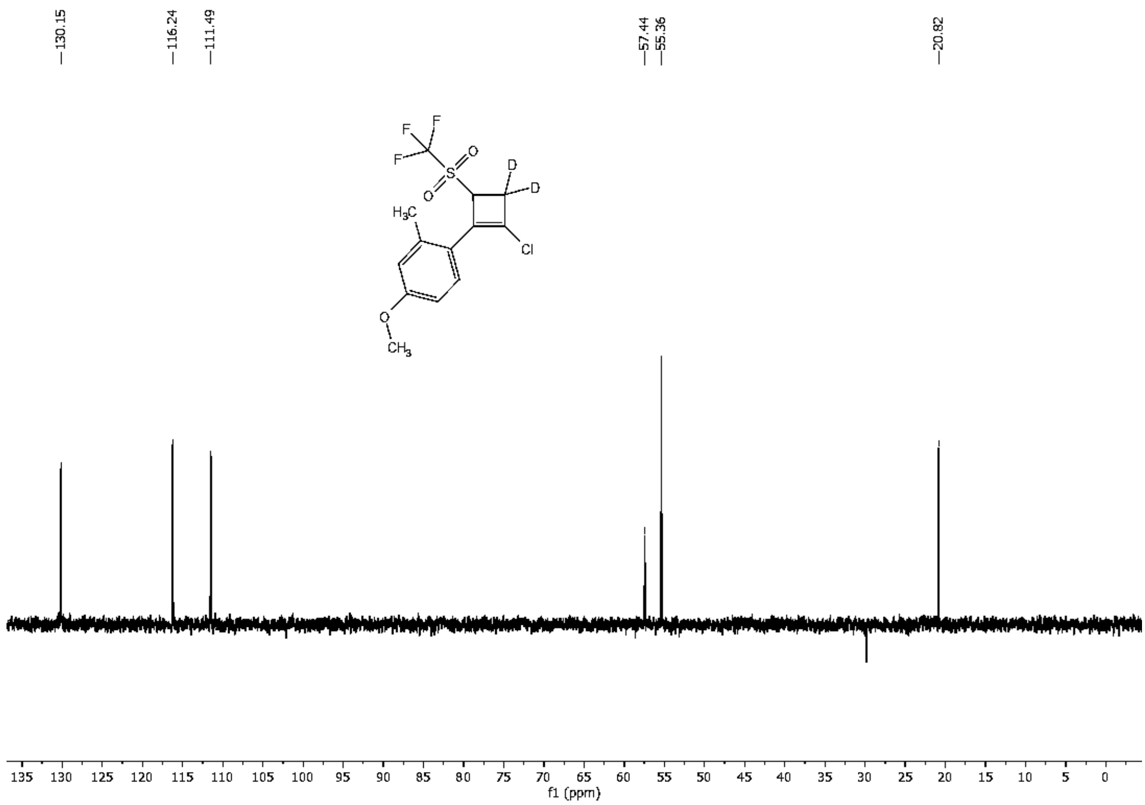

^{19}F NMR compound **3j-d₂** ($CDCl_3$, 282 MHz, 25 °C)

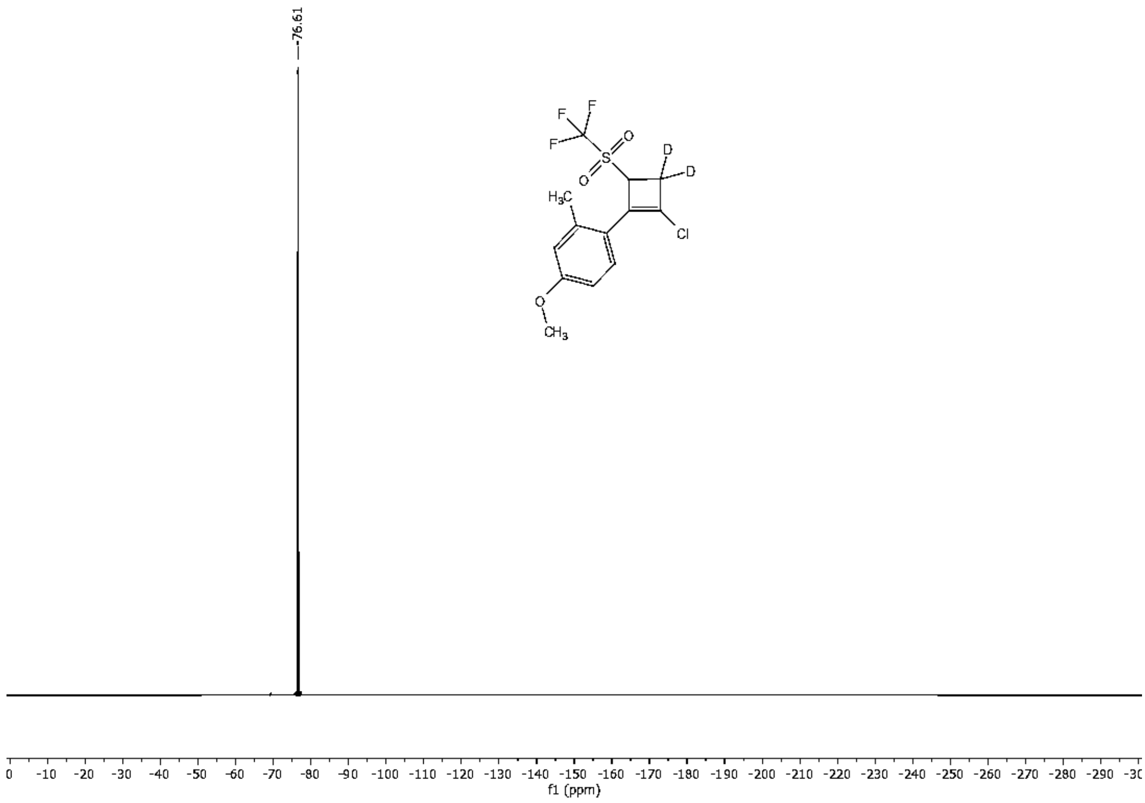

^{1}H NMR compound **3k** ($CDCl_3$, 300 MHz, 25 °C)

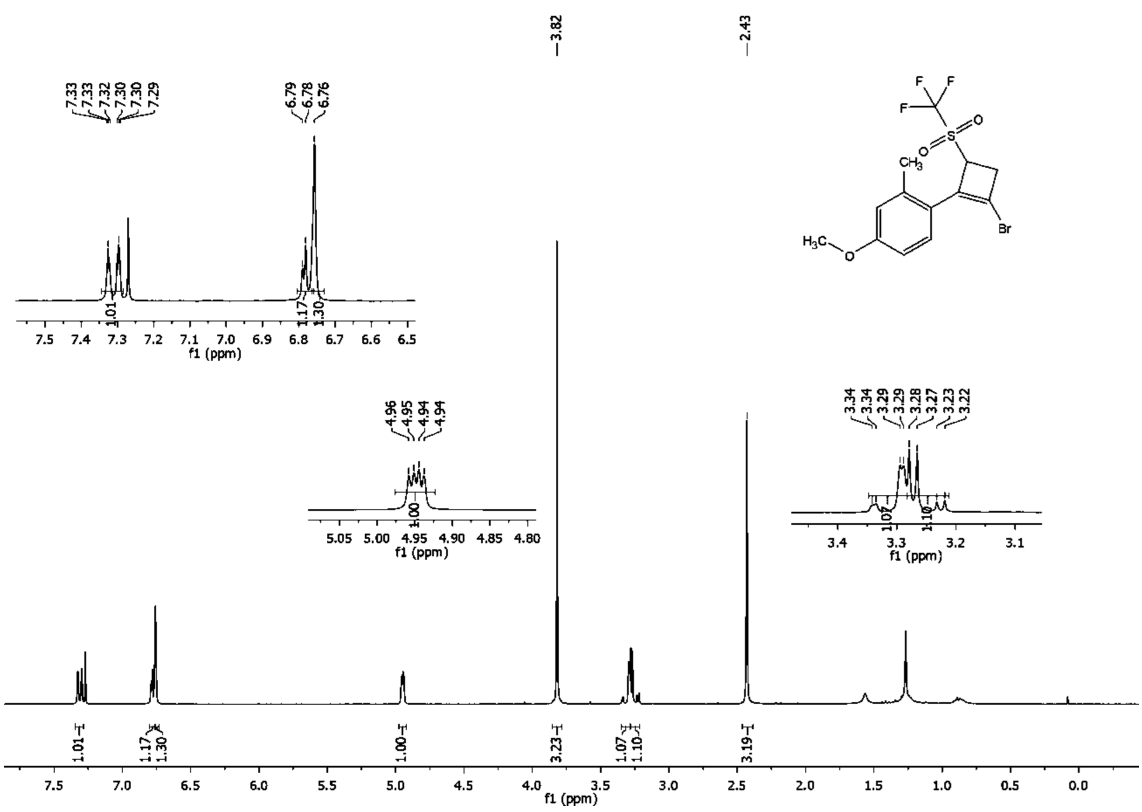

^{13}C NMR compound **3k** ($CDCl_3$, 75 MHz, 25 °C)

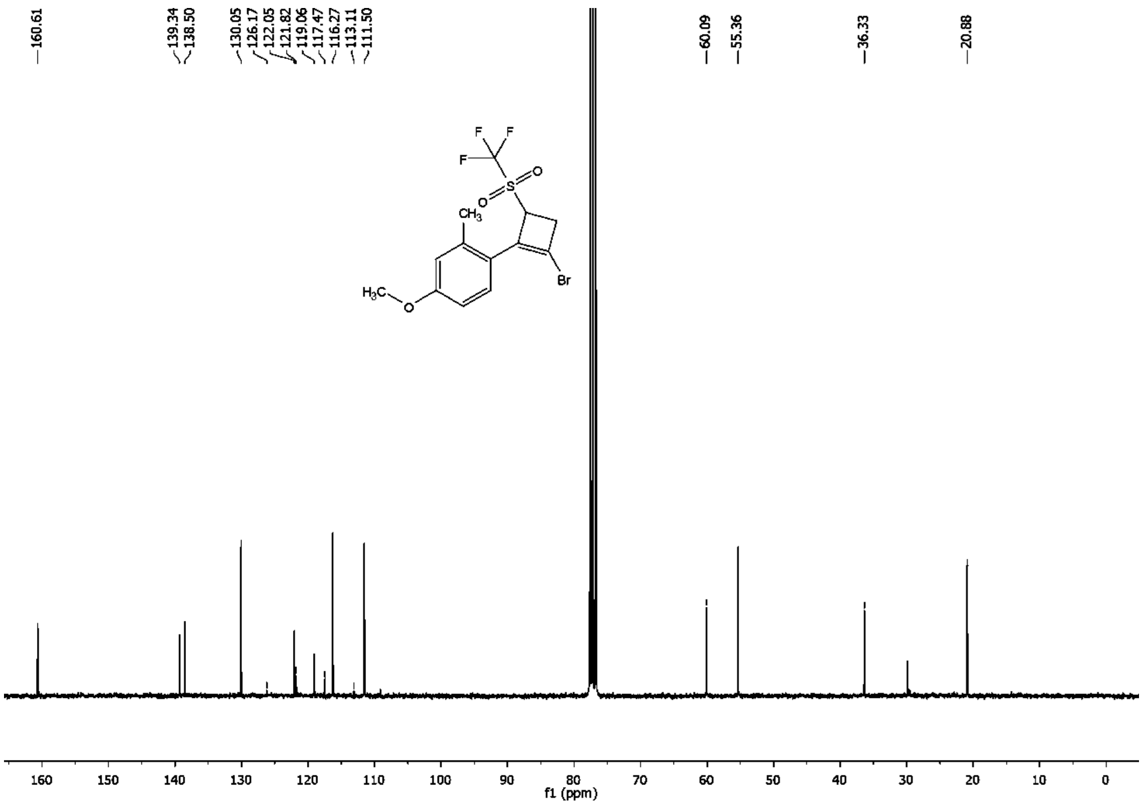

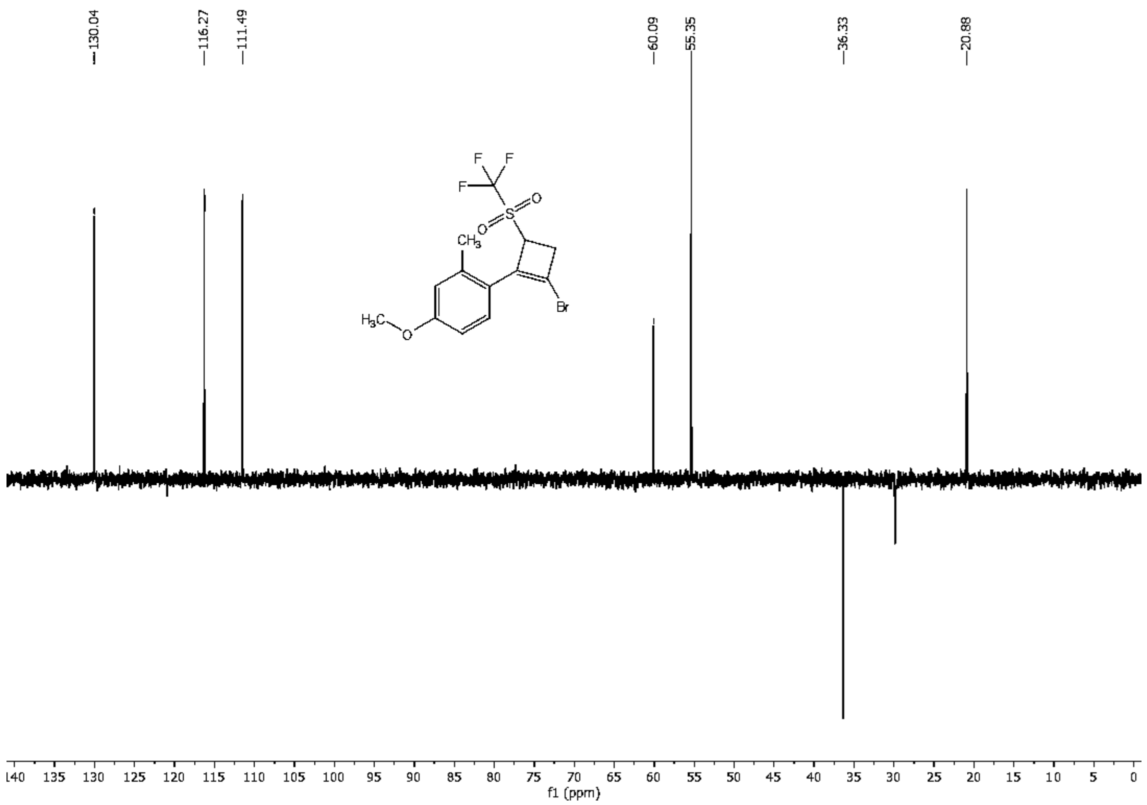

^{19}F NMR compound **3k** ($CDCl_3$, 282 MHz, 25 ^{o}C)

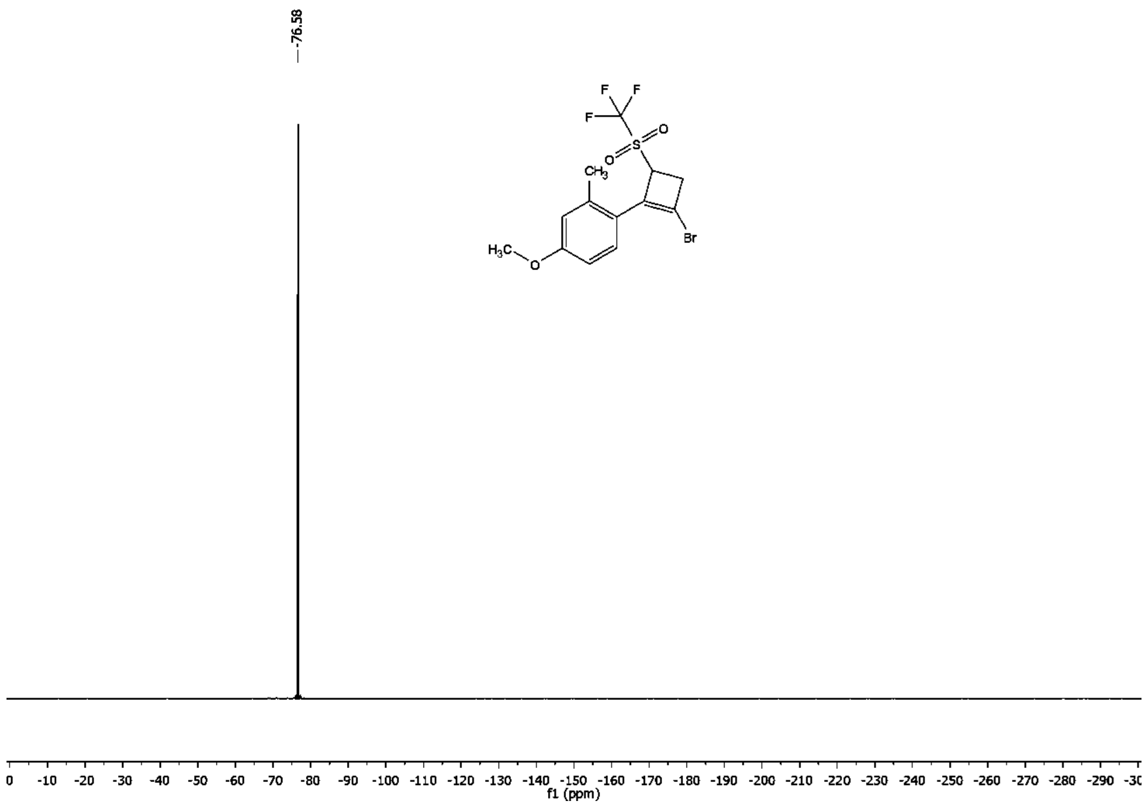

^{1}H NMR compound **3l** ($CDCl_3$, 300 MHz, 25 °C)

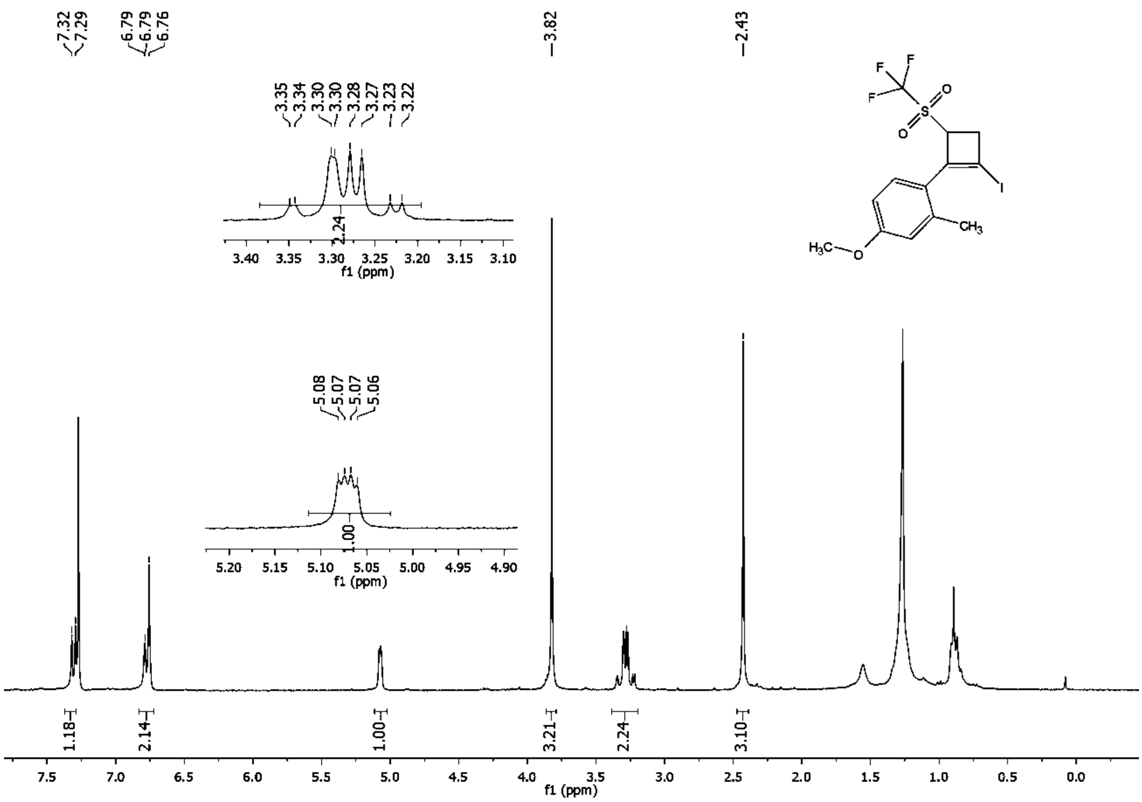

^{13}C NMR compound **3l** ($CDCl_3$, 75 MHz, 25 °C)

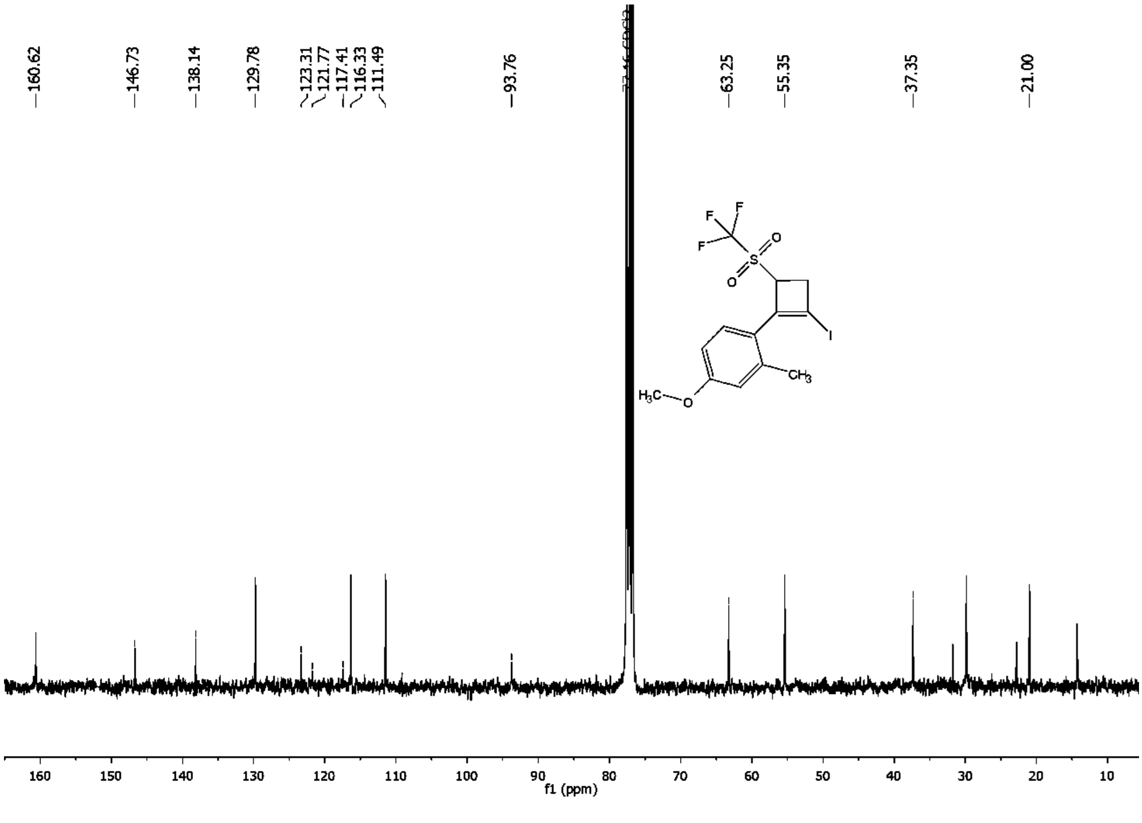

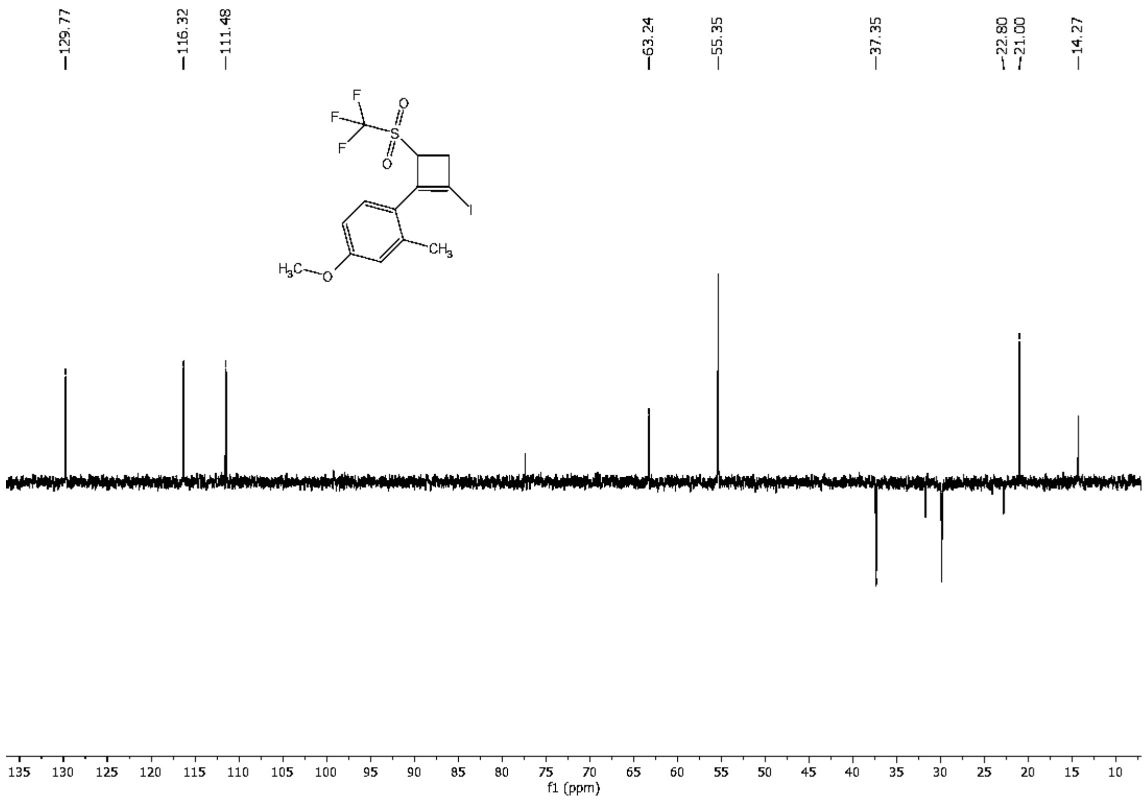

^{19}F NMR compound **3l** ($CDCl_3$, 282 MHz, 25 °C)

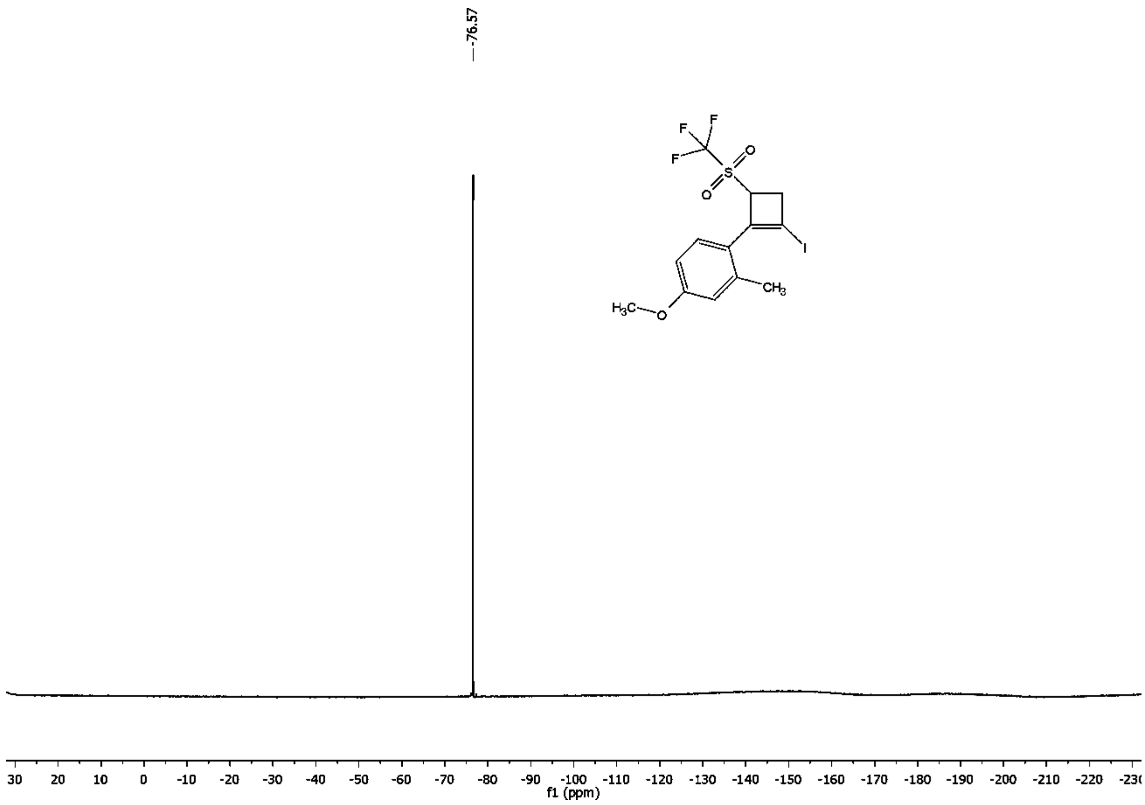

^{1}H NMR compound **3m** ($CDCl_3$, 300 MHz, 25 °C)

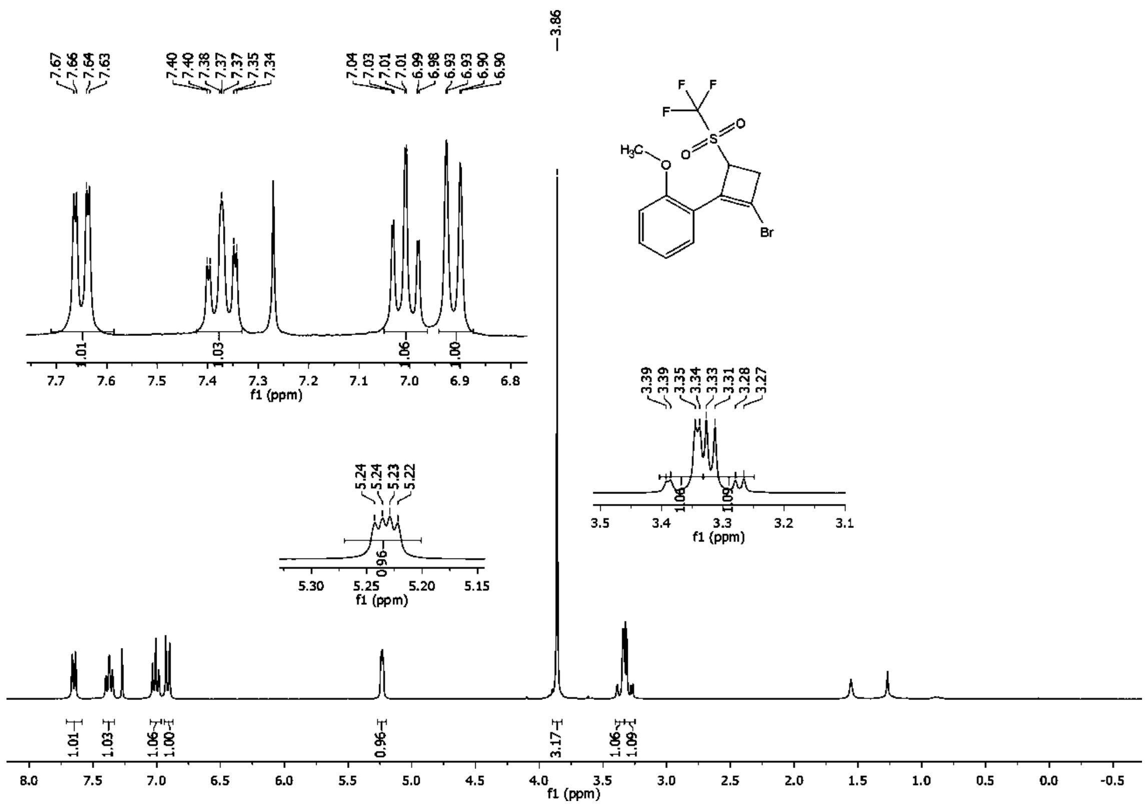

^{13}C NMR compound **3m** ($CDCl_3$, 75 MHz, 25 °C)

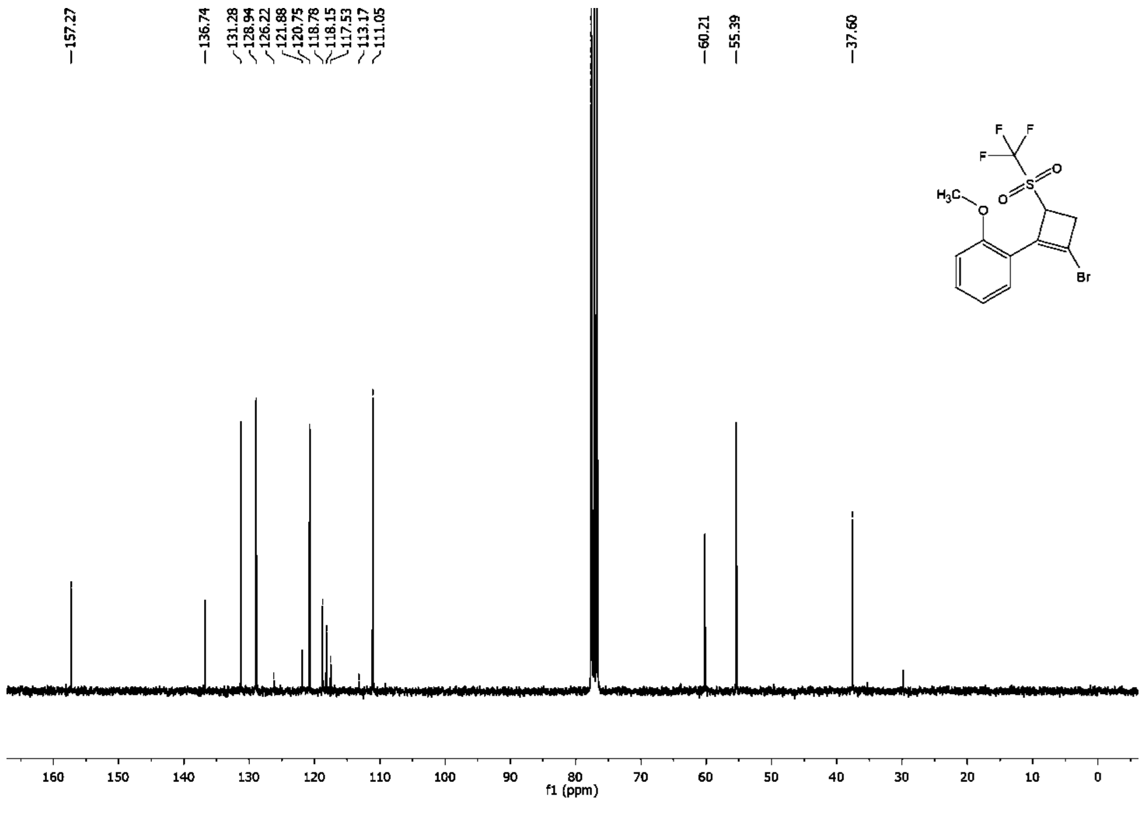

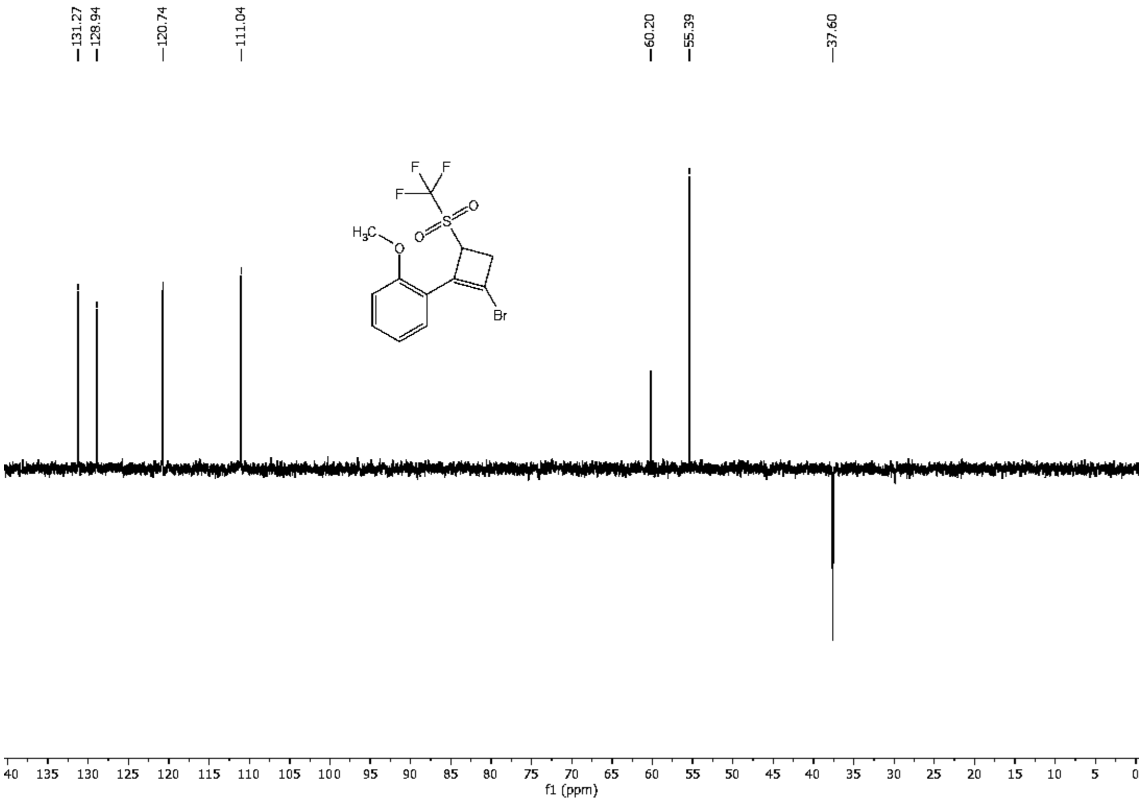

^{19}F NMR compound **3m** ($CDCl_3$, 282 MHz, 25 ^{o}C)

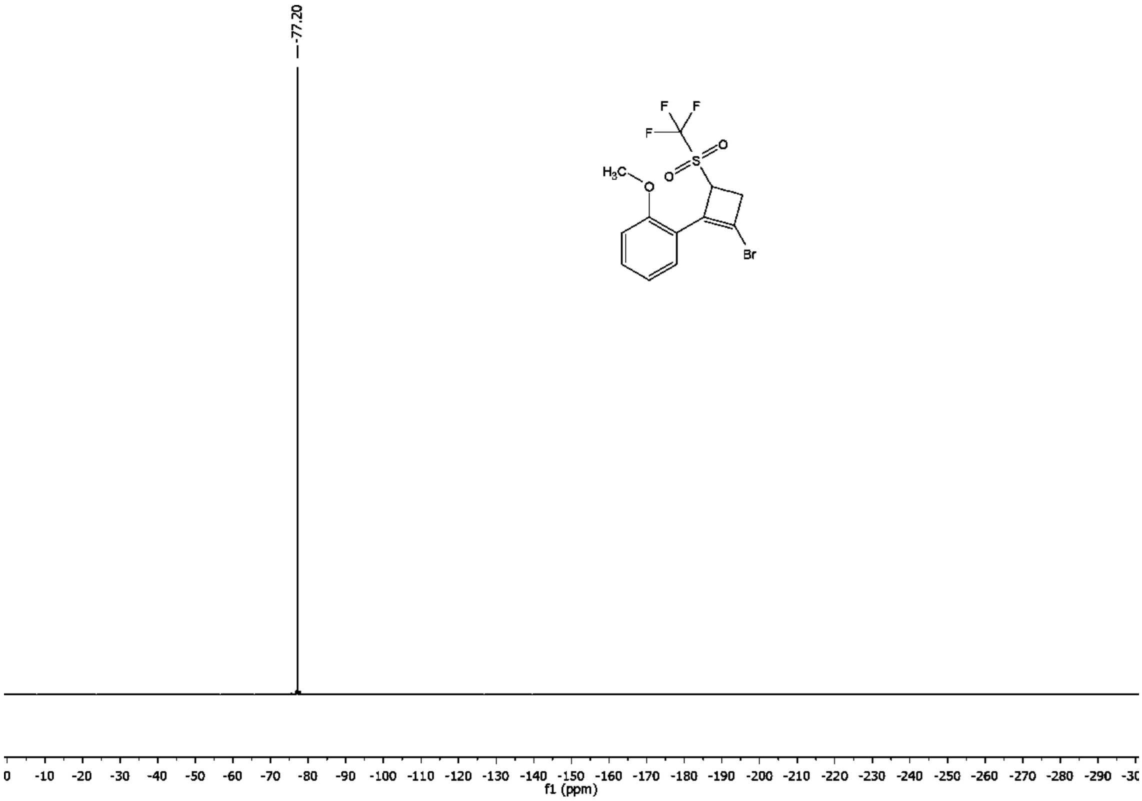

^{1}H NMR compound **3n** ($CDCl_3$, 300 MHz, 25 °C)

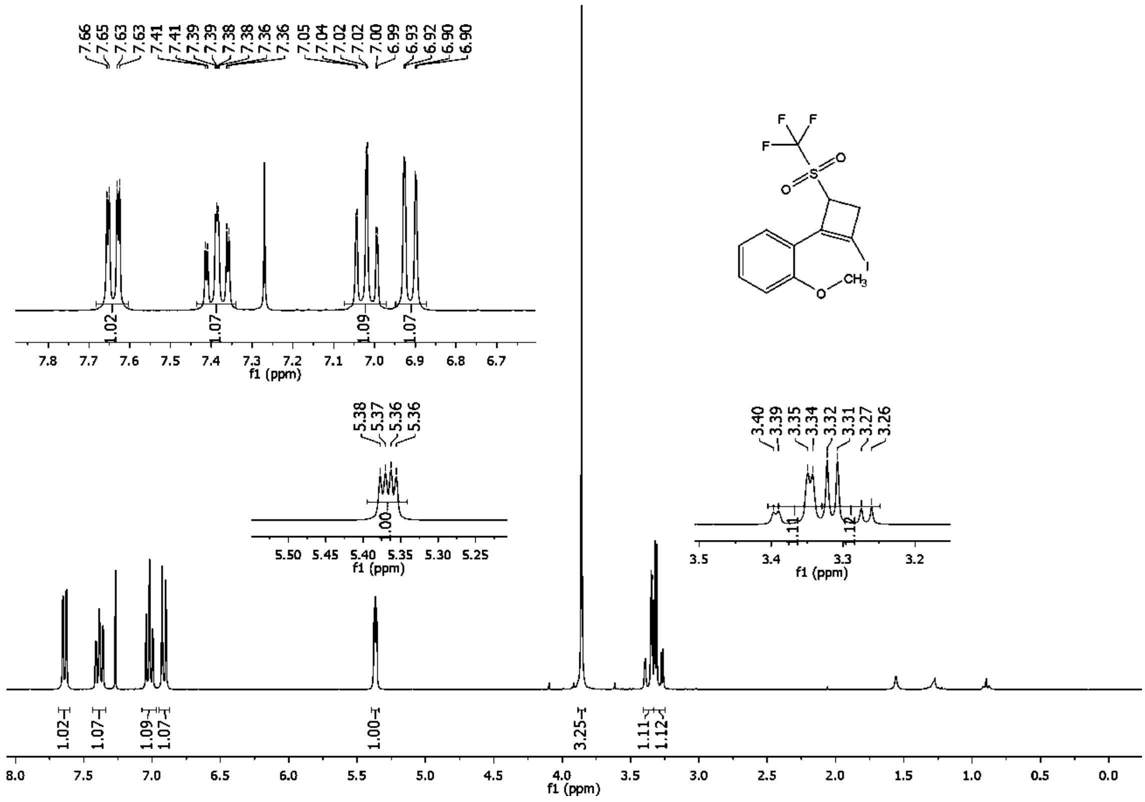

^{13}C NMR compound **3n** ($CDCl_3$, 75 MHz, 25 °C)

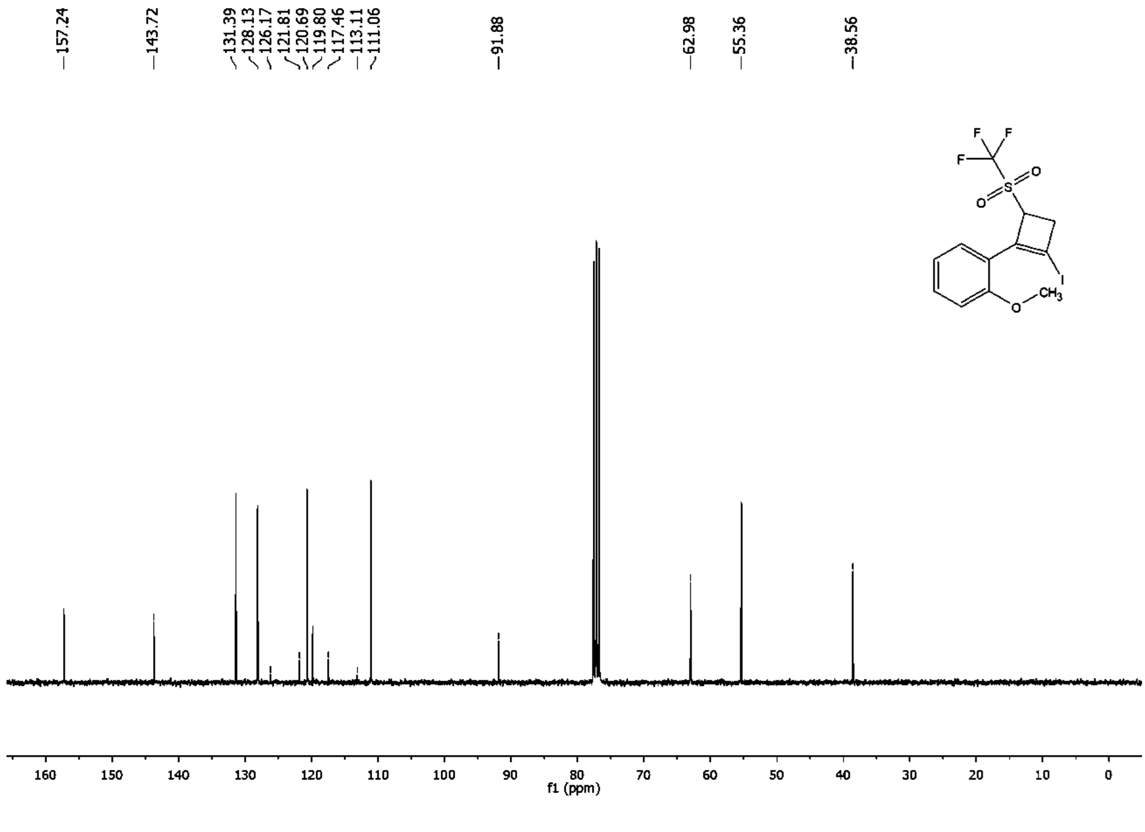

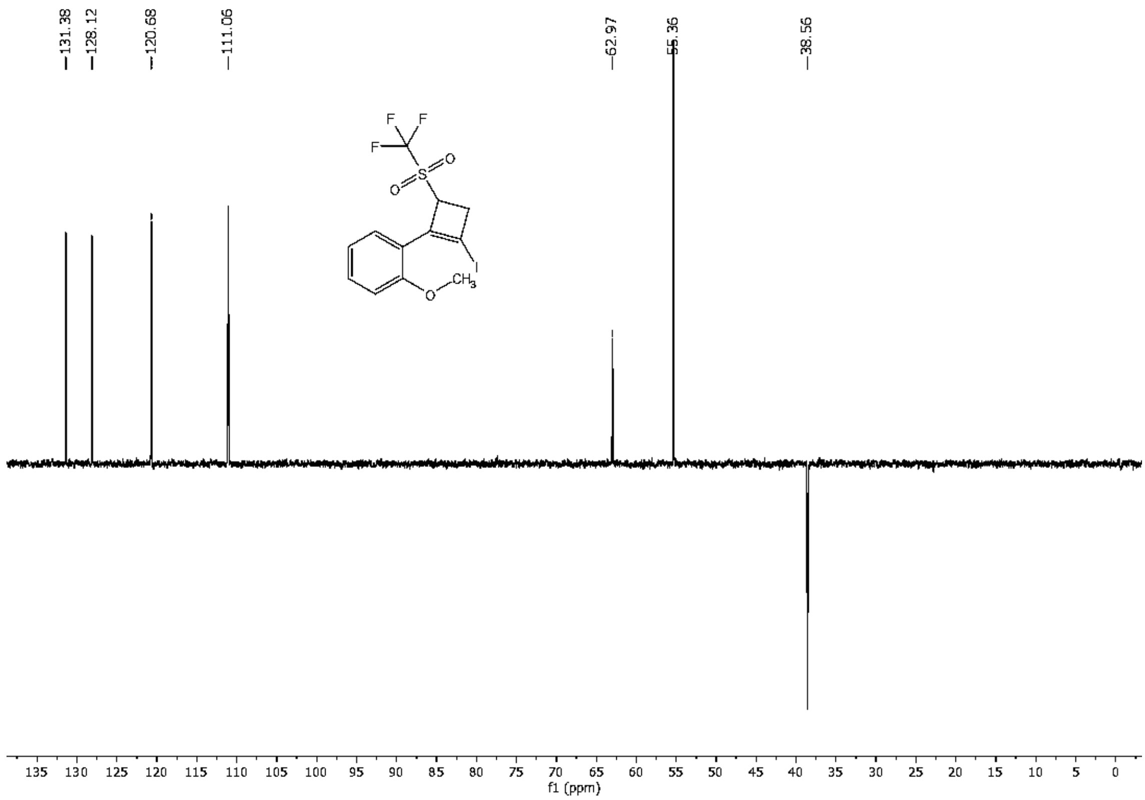

^{19}F NMR compound **3n** ($CDCl_3$, 282 MHz, 25 °C)

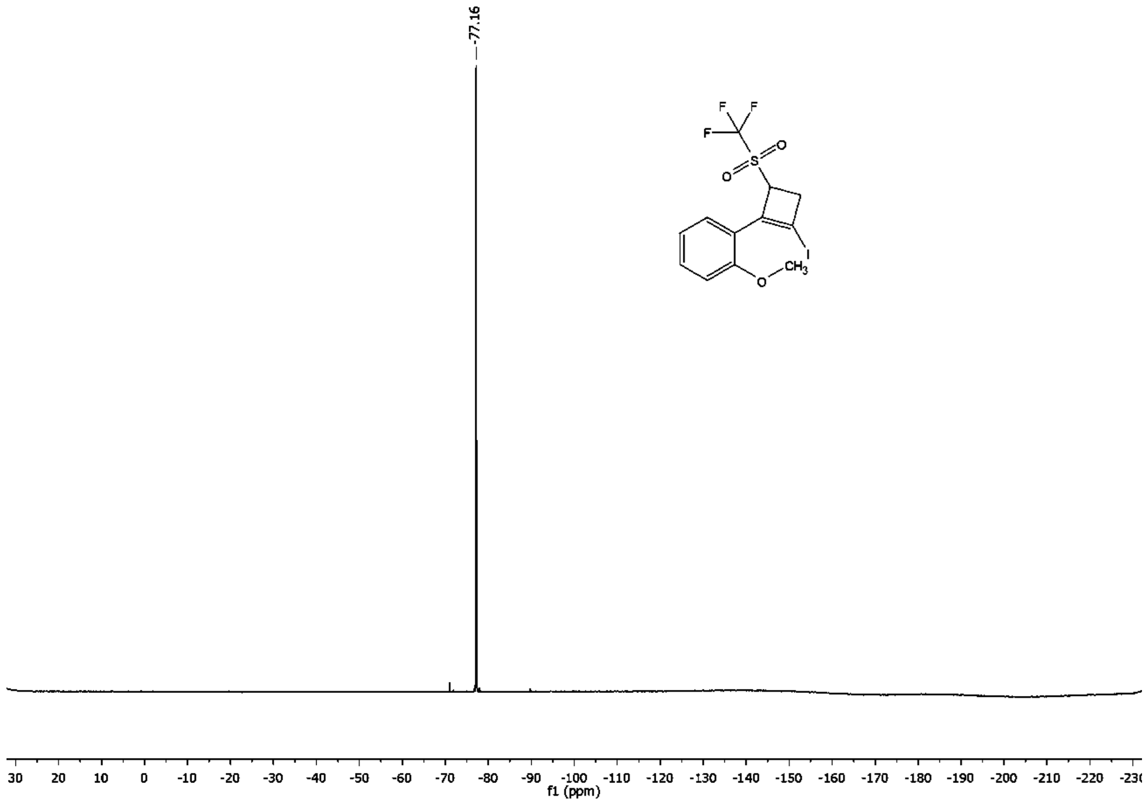

^{1}H NMR compound **3o** ($CDCl_3$, 700 MHz, 25 °C)

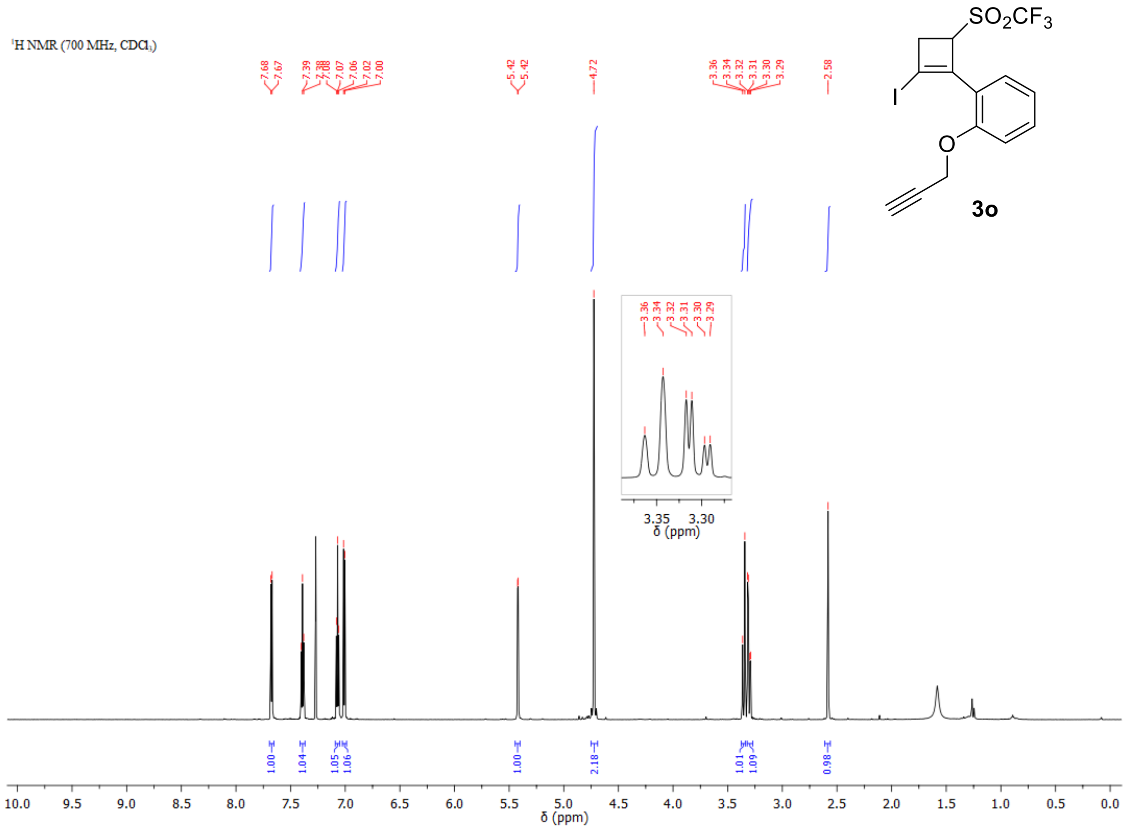

^{13}C NMR compound **3o** ($CDCl_3$, 175 MHz, 25 °C)

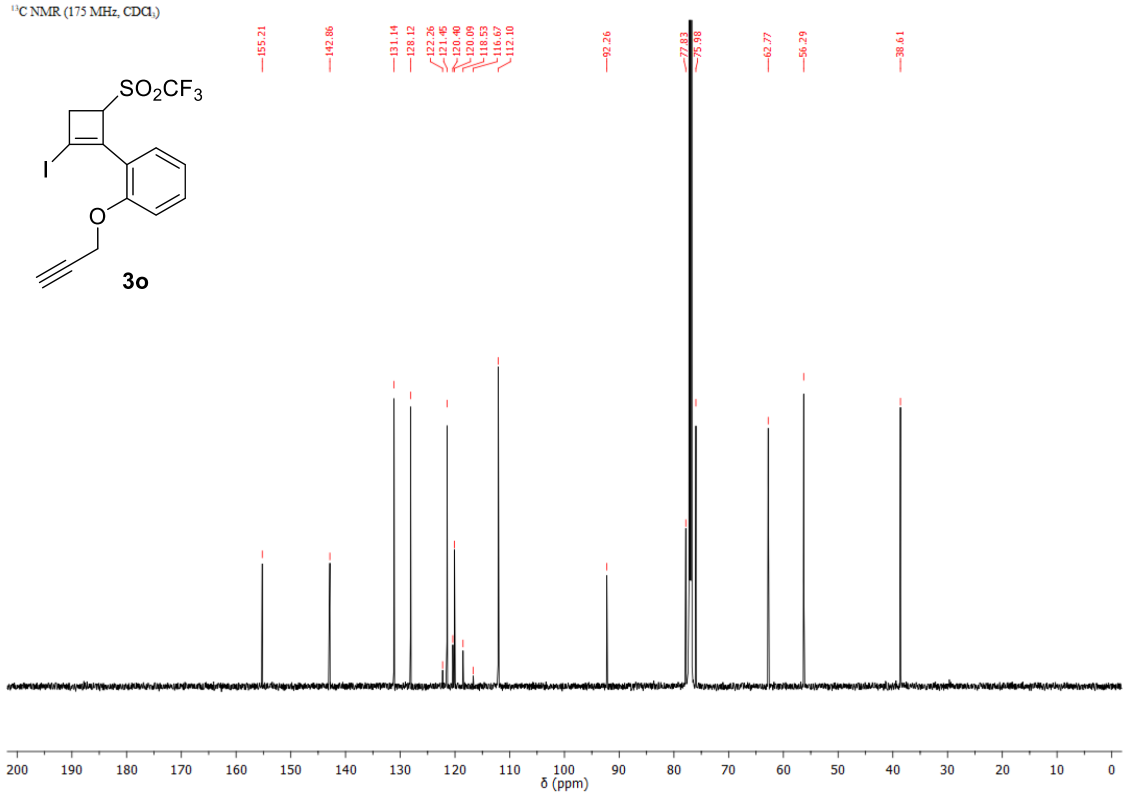

^{1}H – ^{1}H COSY compound **3o**

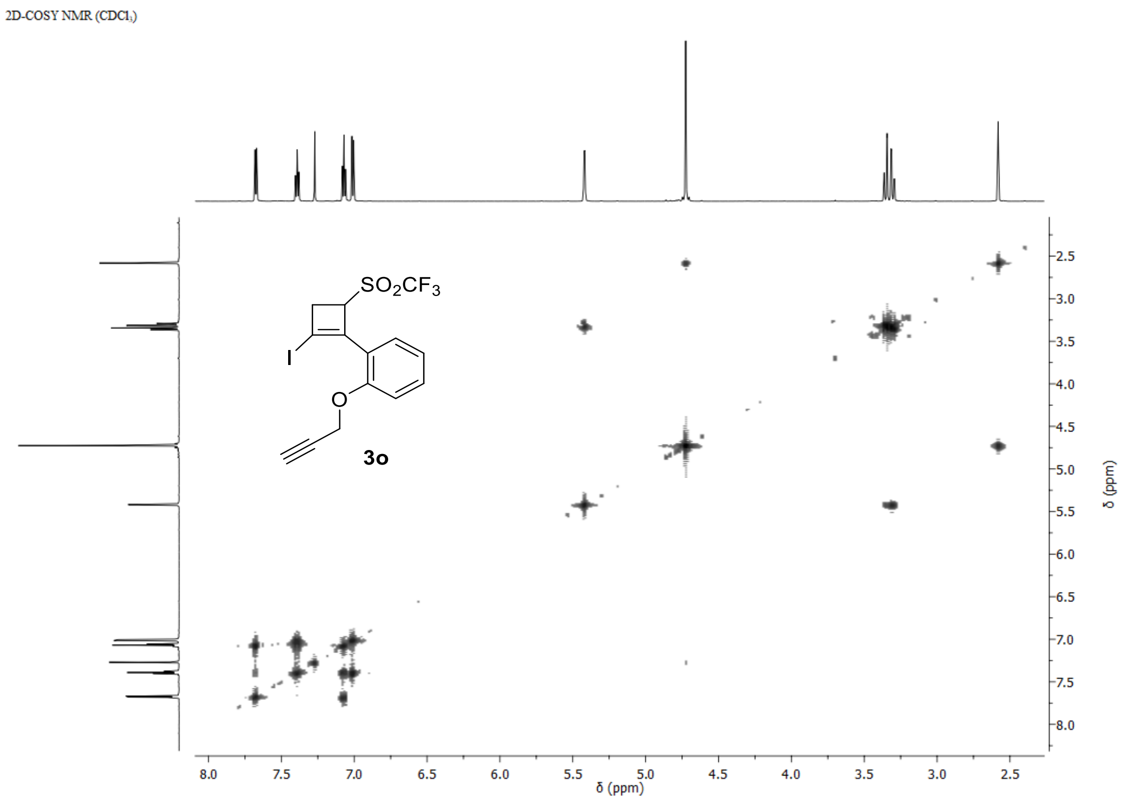

^{1}H – ^{13}C HMQC compound **3o**

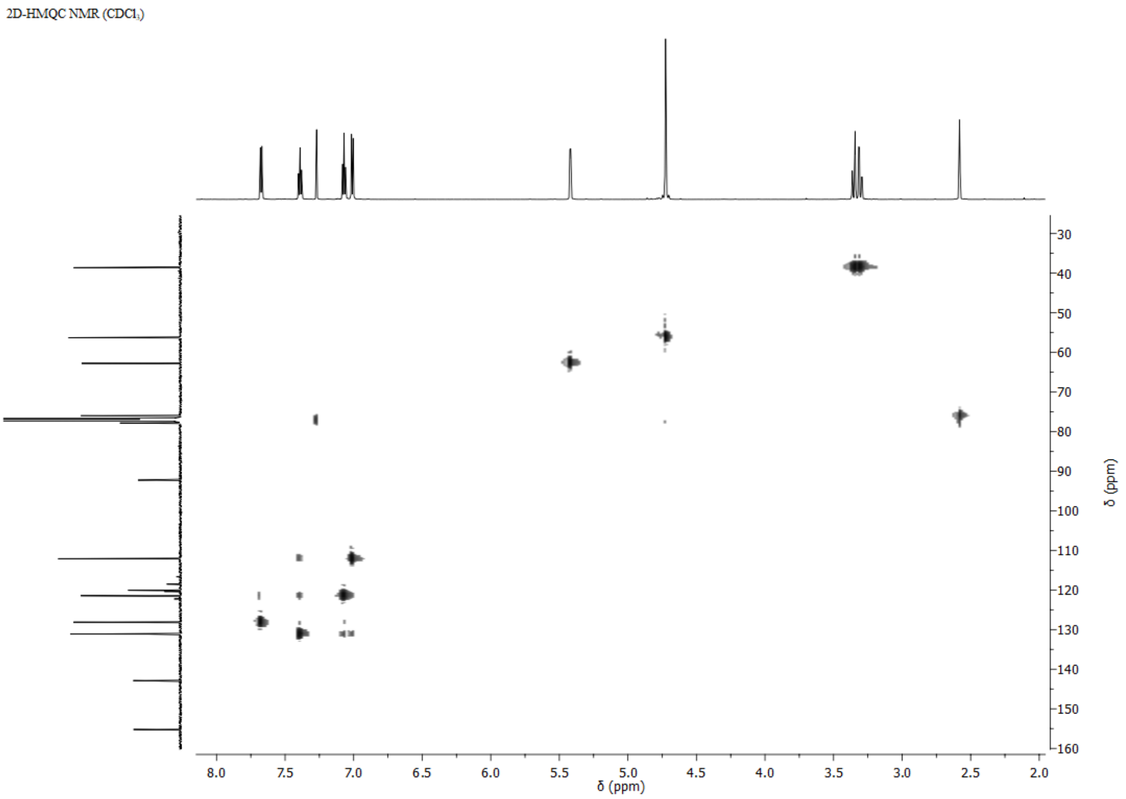

^{1}H – ^{13}C HMBC compound **3o**

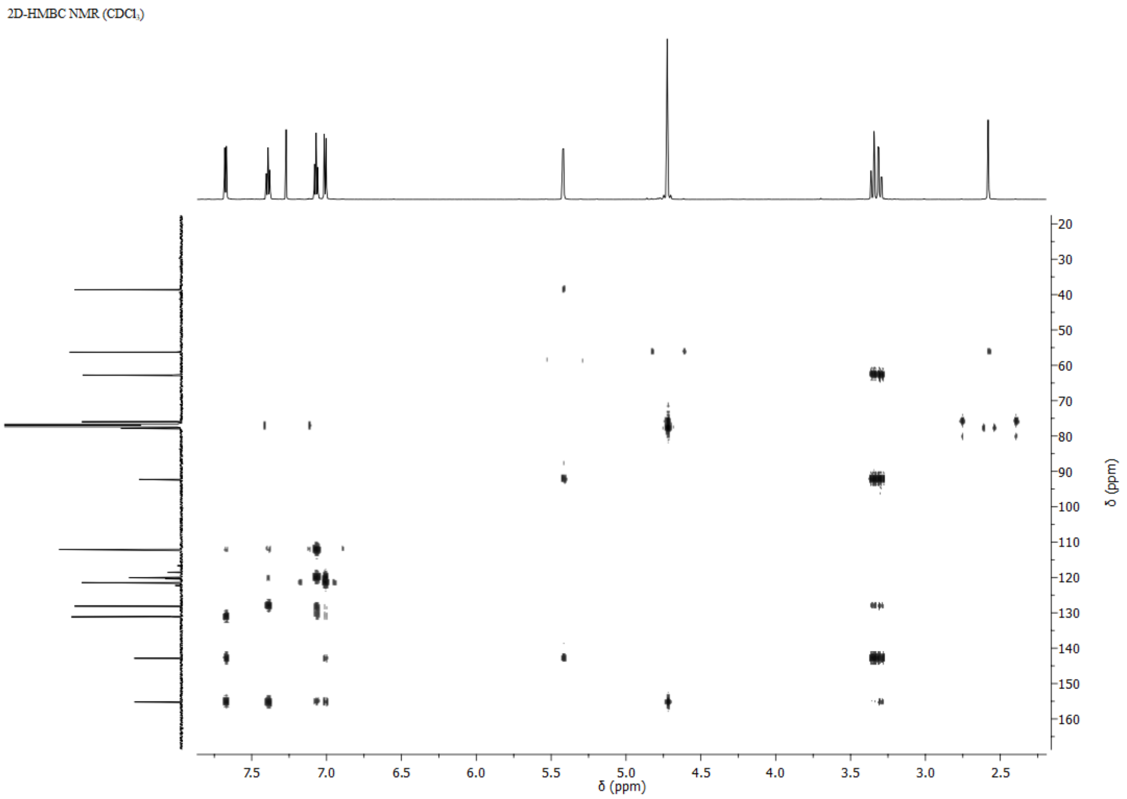

^{19}F NMR compound **3o** ($CDCl_3$, 282 MHz, 25 °C)

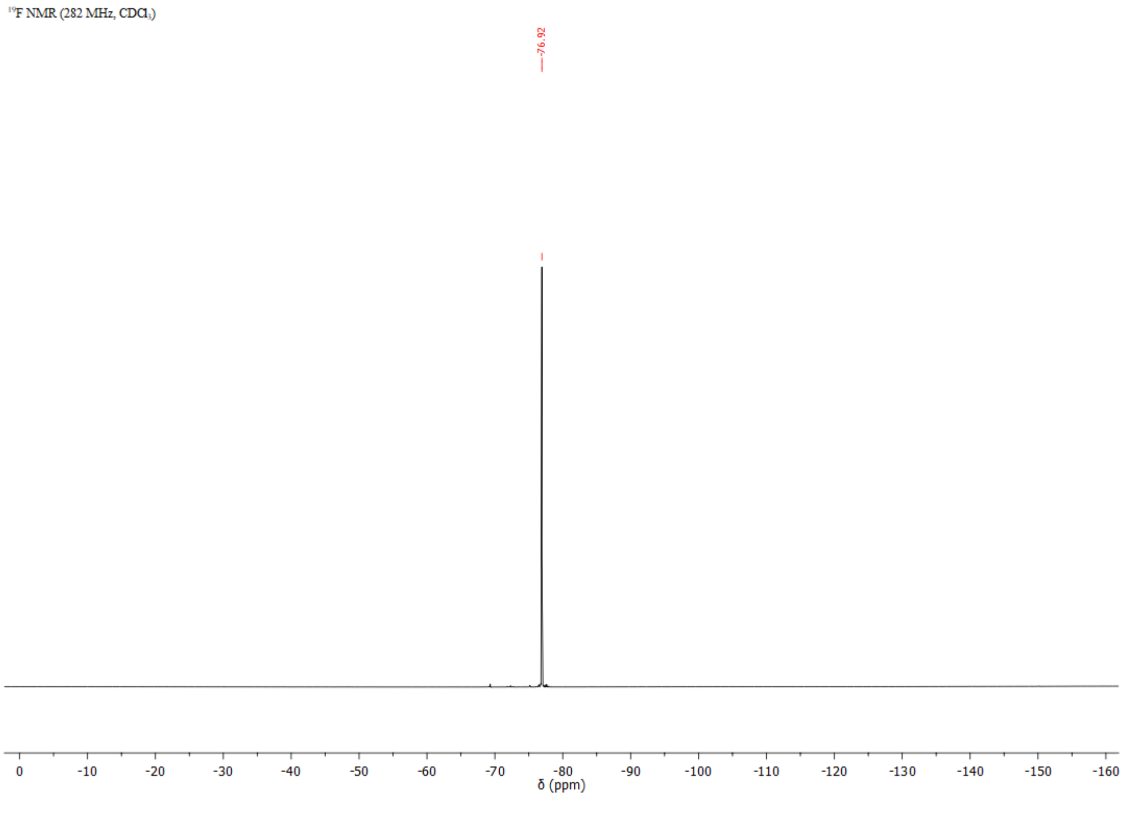

^{1}H NMR compound **3p** ($CDCl_3$, 300 MHz, 25 °C)

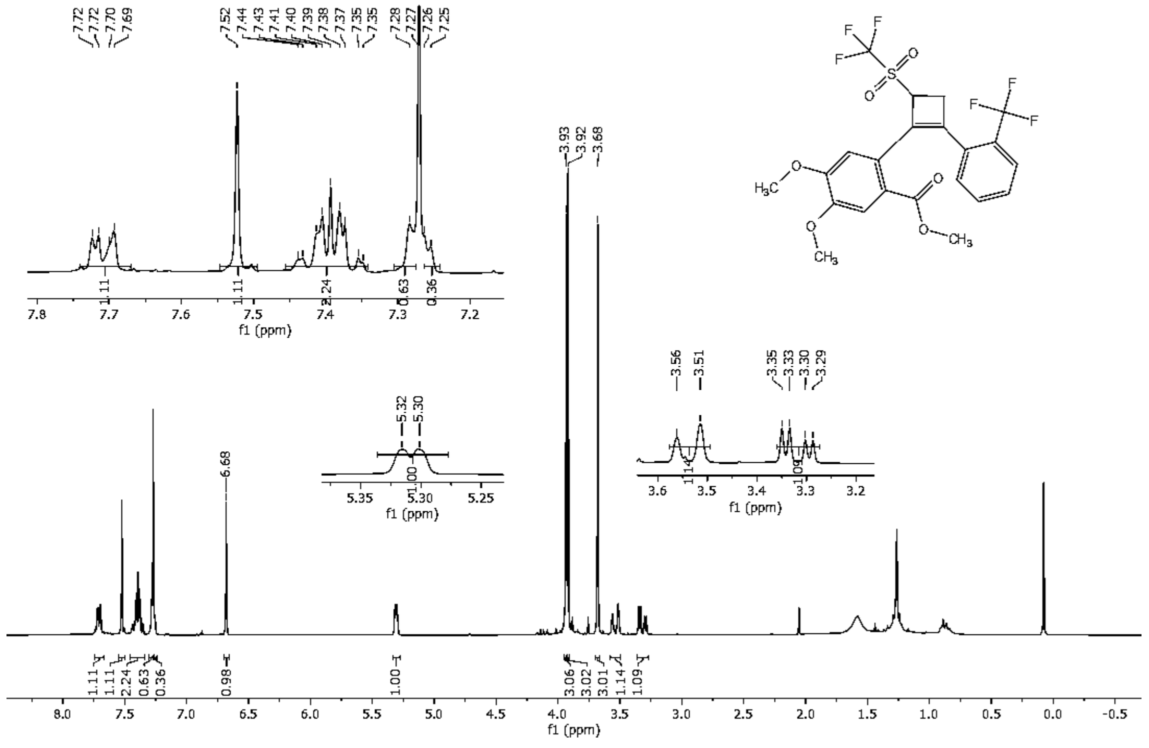

^{13}C NMR compound **3p** ($CDCl_3$, 75 MHz, 25 °C)

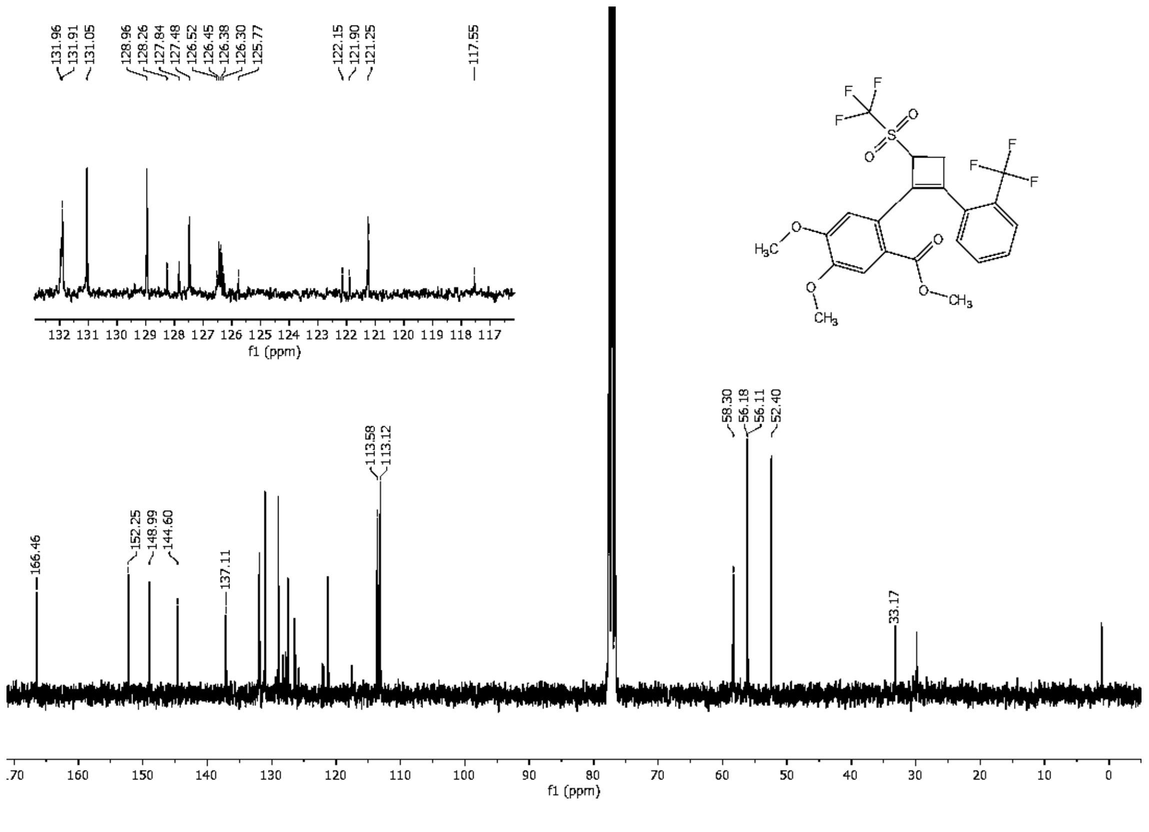

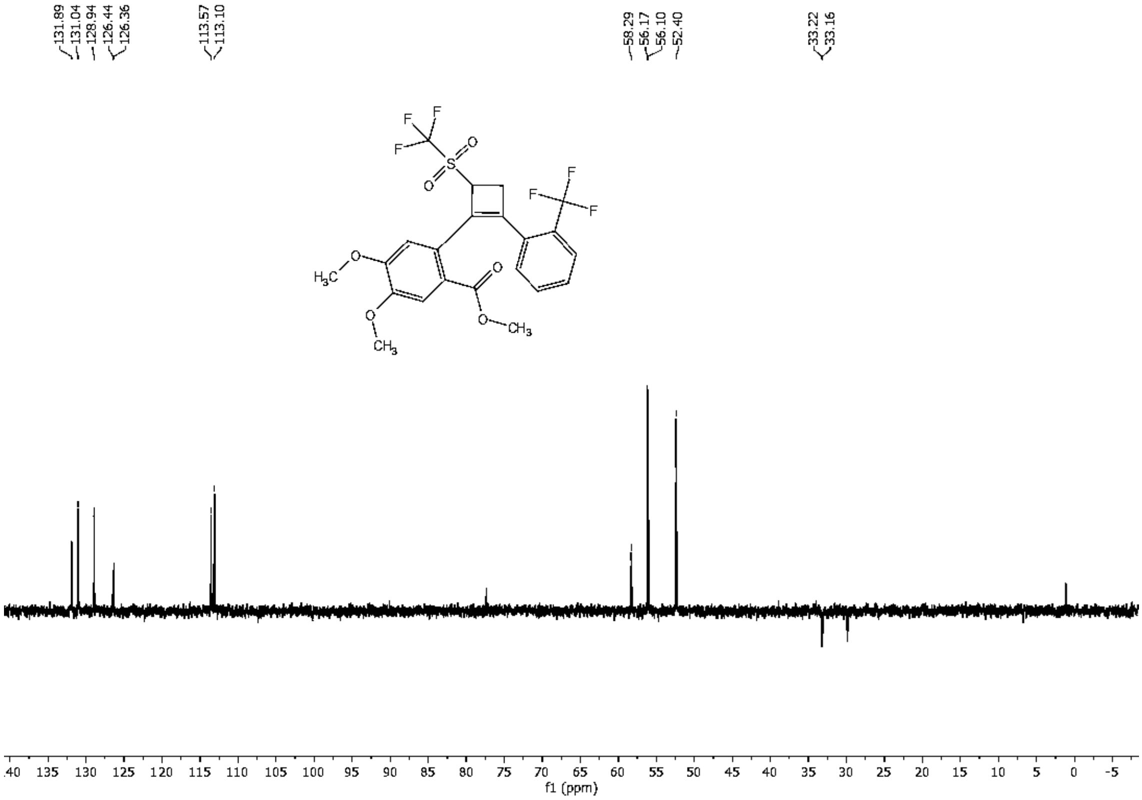

^{19}F NMR compound **3p** ($CDCl_3$, 282 MHz, 25 °C)

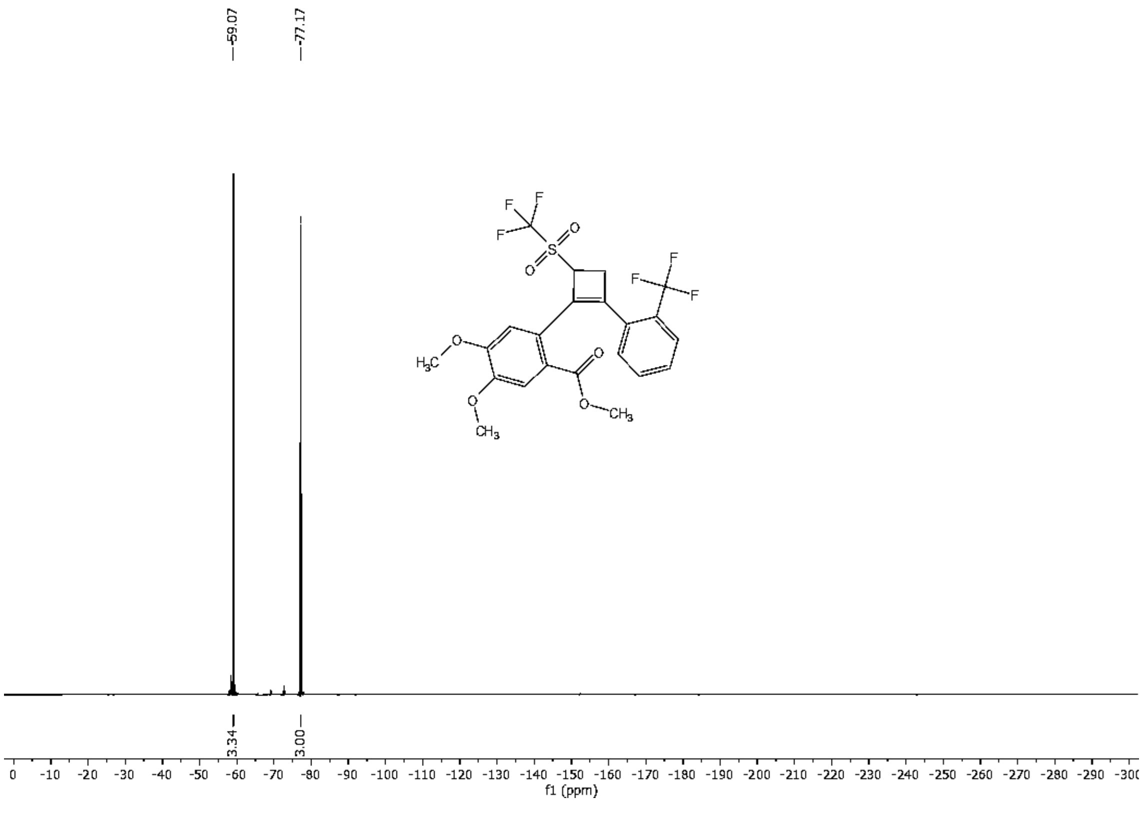

^{1}H NMR compound **3q** ($CDCl_3$, 300 MHz, 25 °C)

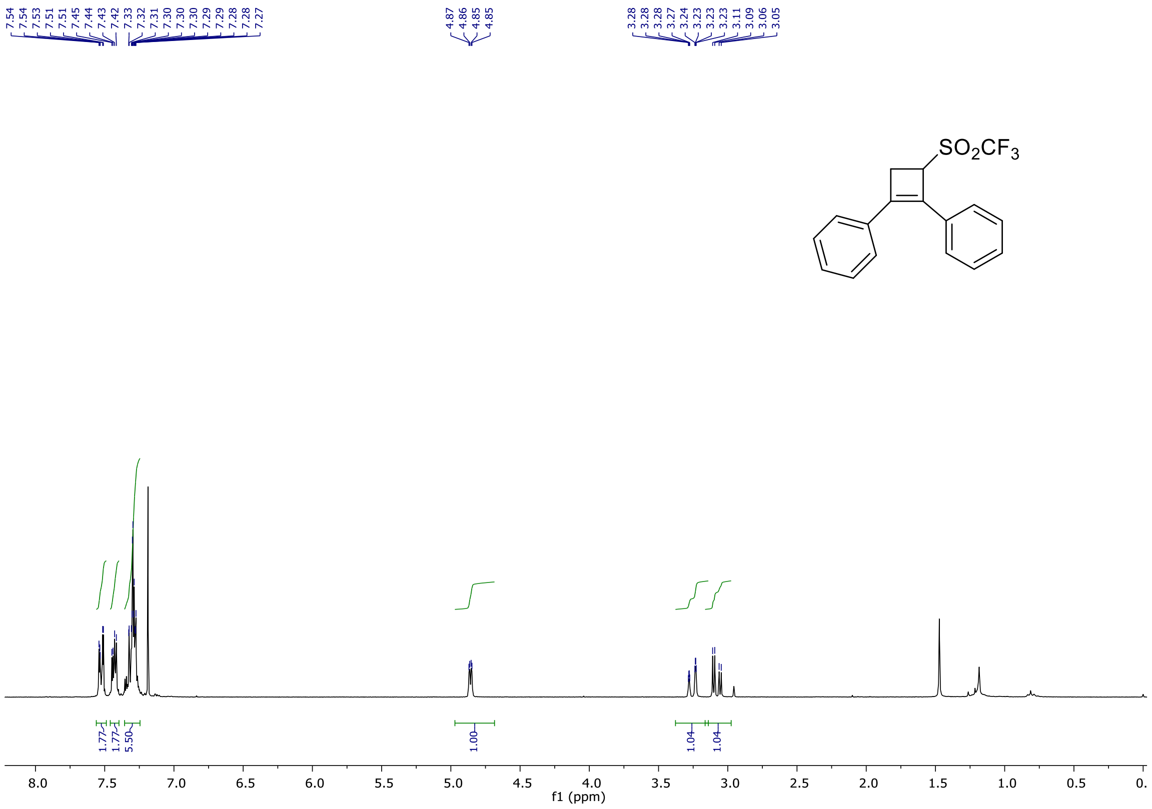

^{13}C NMR compound **3q** ($CDCl_3$, 75 MHz, 25 °C)

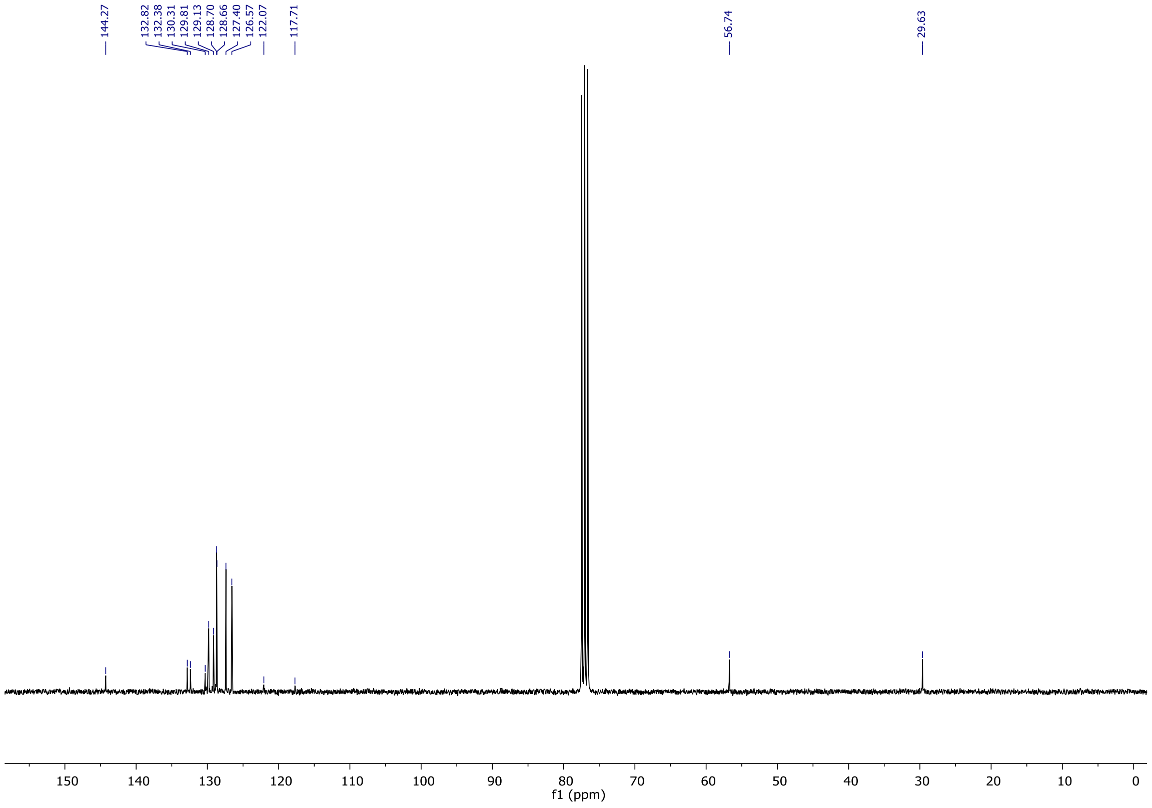

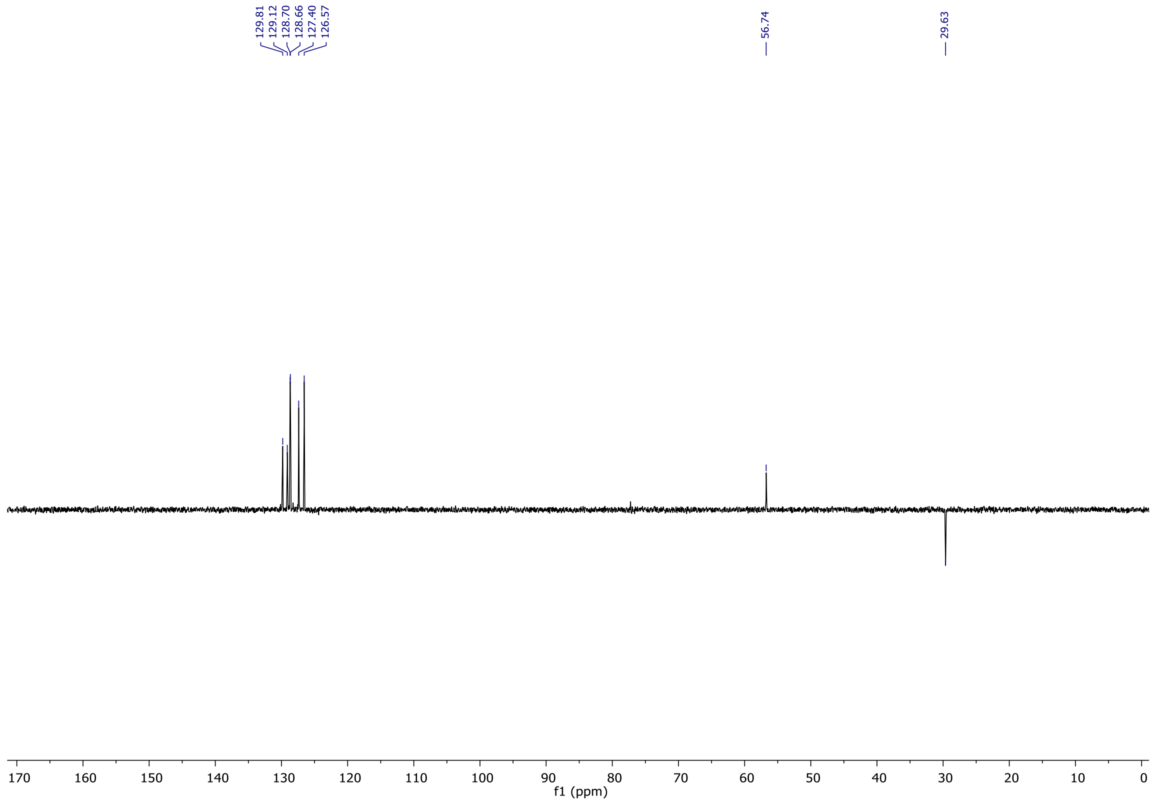

^{19}F NMR compound **3q** ($CDCl_3$, 282 MHz, 25 °C)

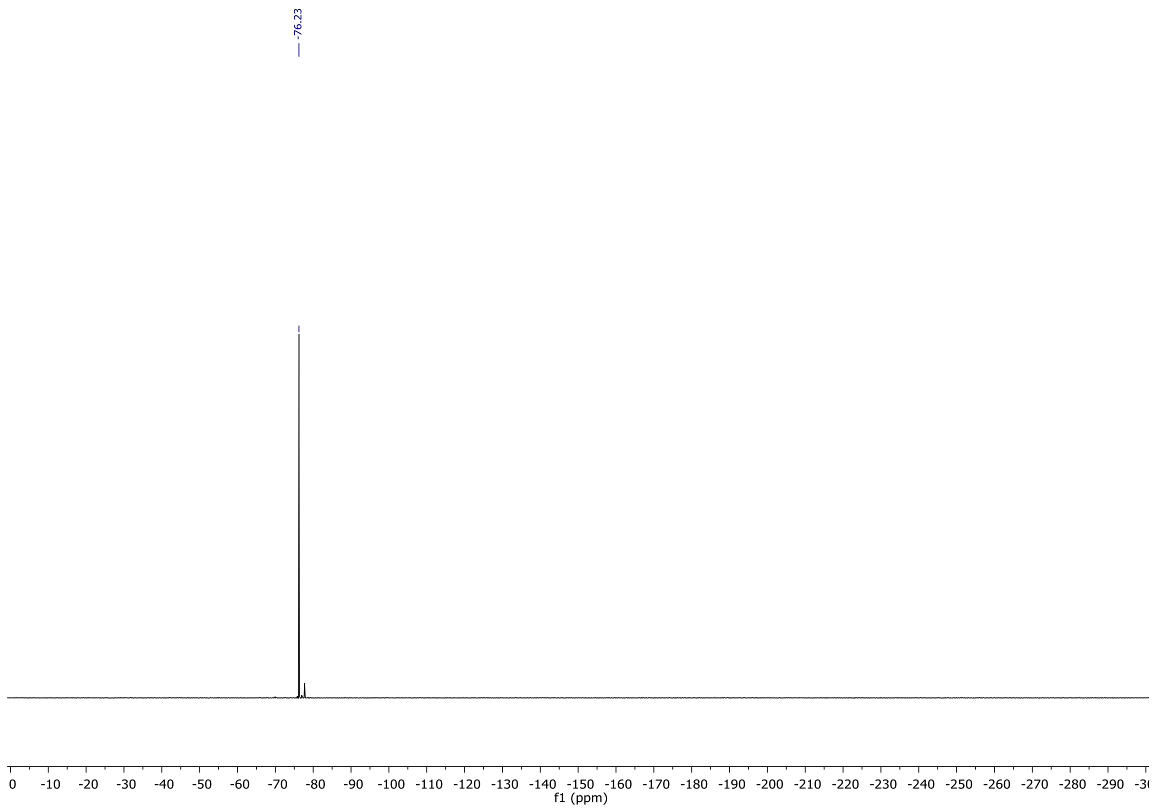

^{1}H NMR compound **3r** ($CDCl_3$, 300 MHz, 25 °C)

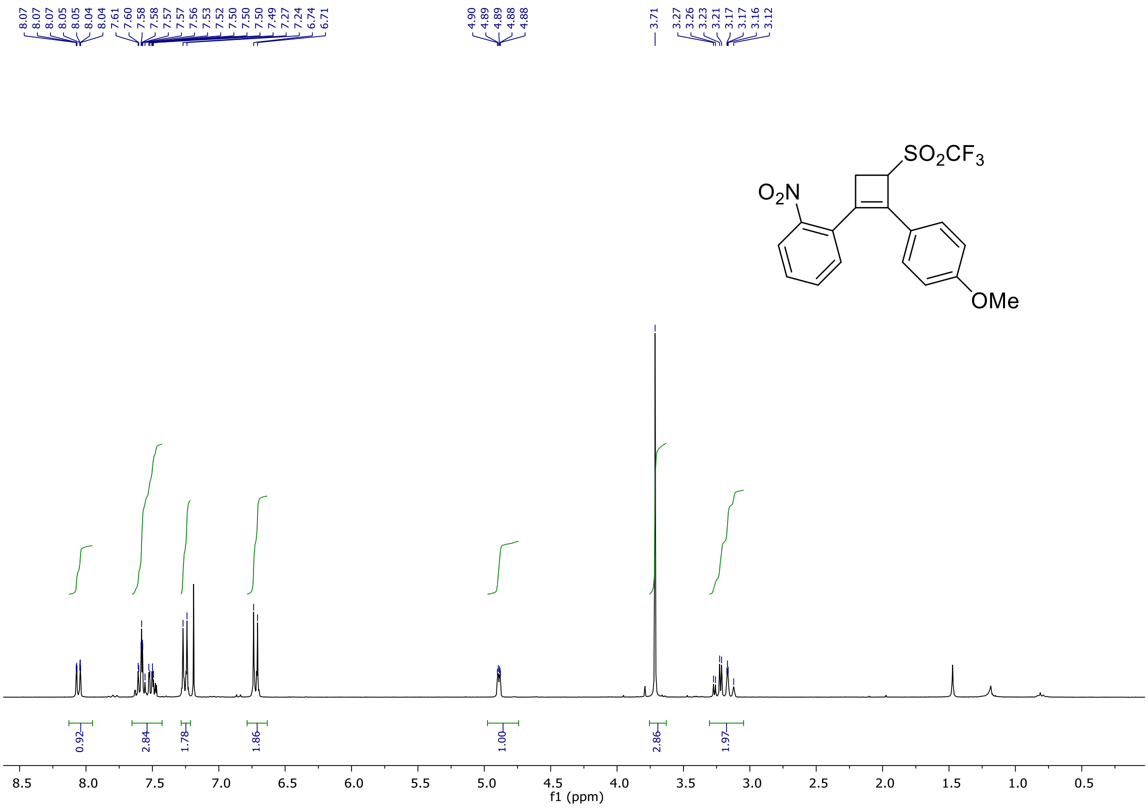

^{13}C NMR compound **3r** ($CDCl_3$, 75 MHz, 25 °C)

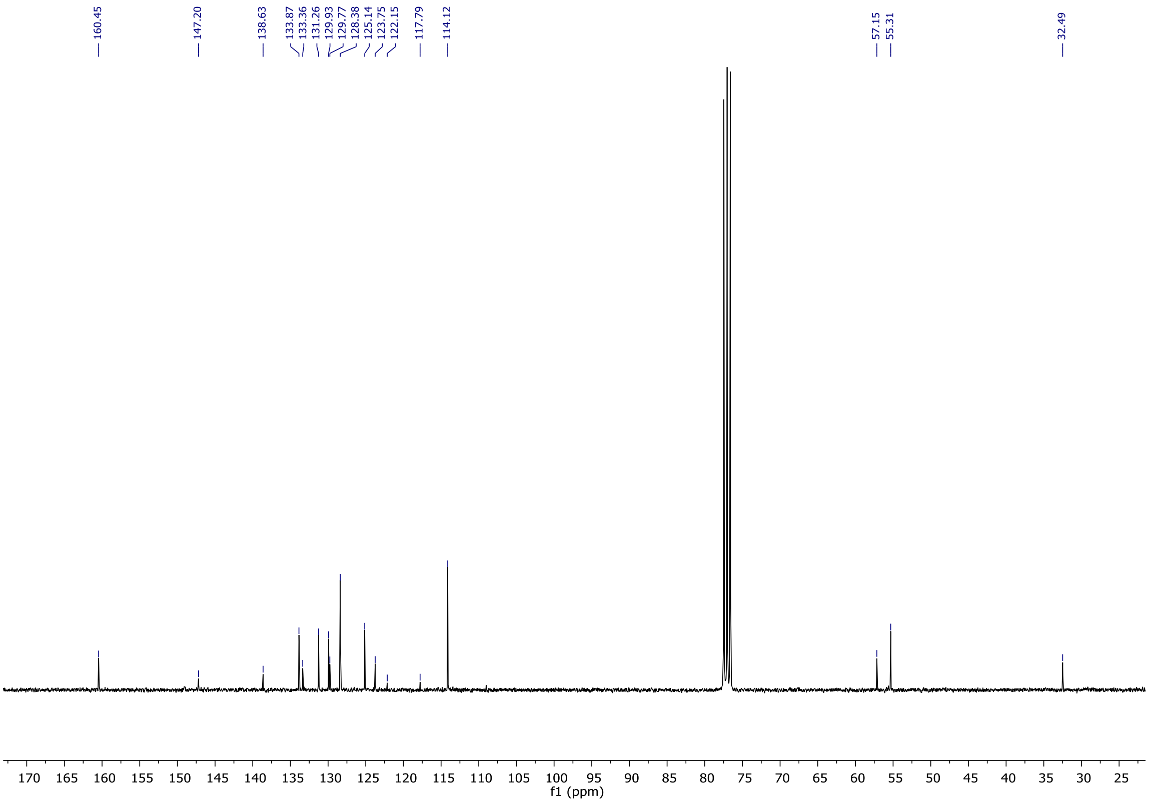

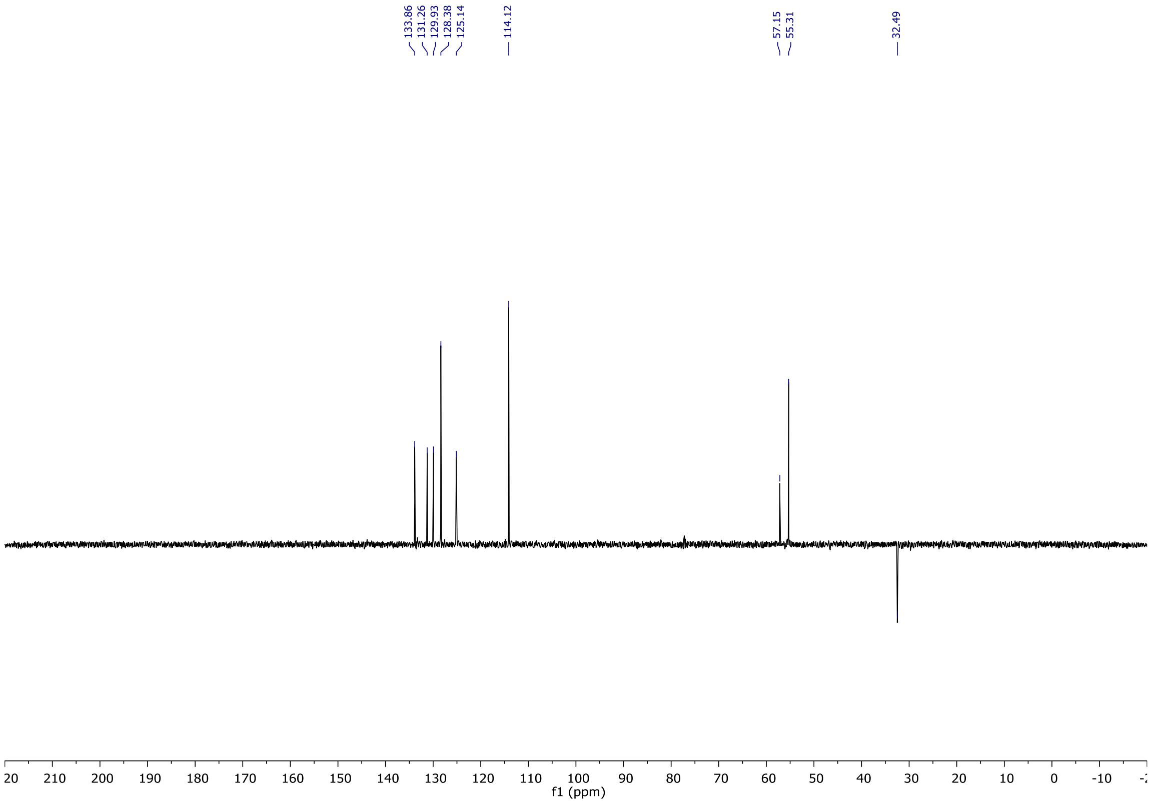
133.86
131.26
129.93
128.38
125.14
114.12
57.15
55.31
32.49
f1 (ppm)

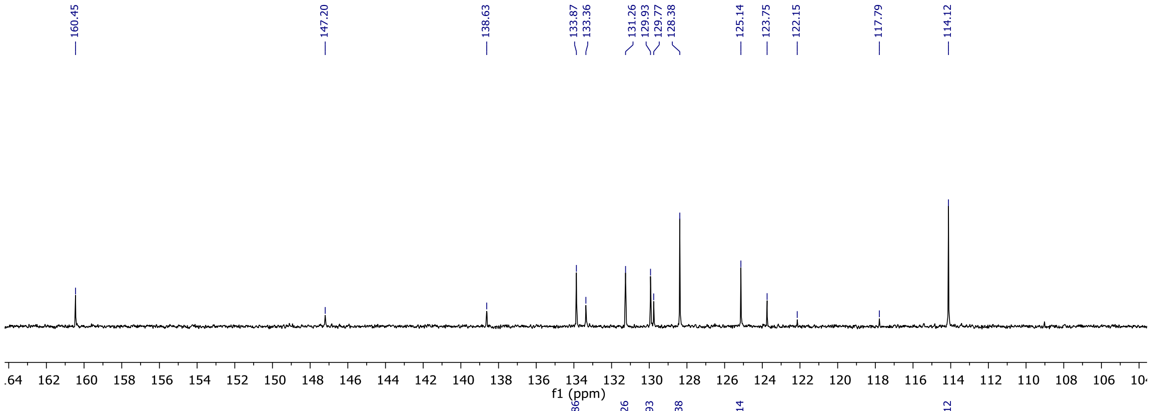
160.45
147.20
138.63
133.87
133.36
131.26
129.93
129.77
128.38
125.14
123.75
122.15
117.79
114.12
f1 (ppm)

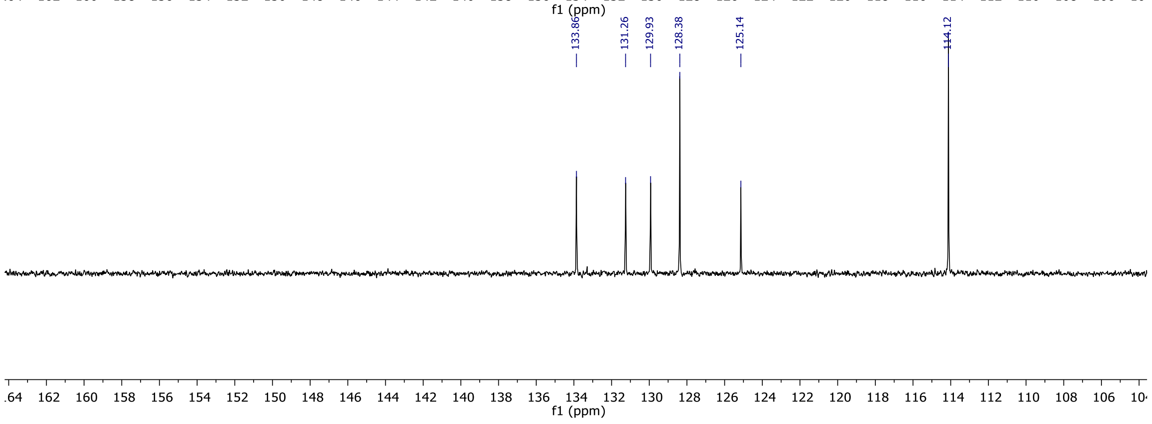
133.86
131.26
129.93
128.38
125.14
114.12
f1 (ppm)

^{19}F NMR compound **3r** ($CDCl_3$, 282 MHz, 25 °C)

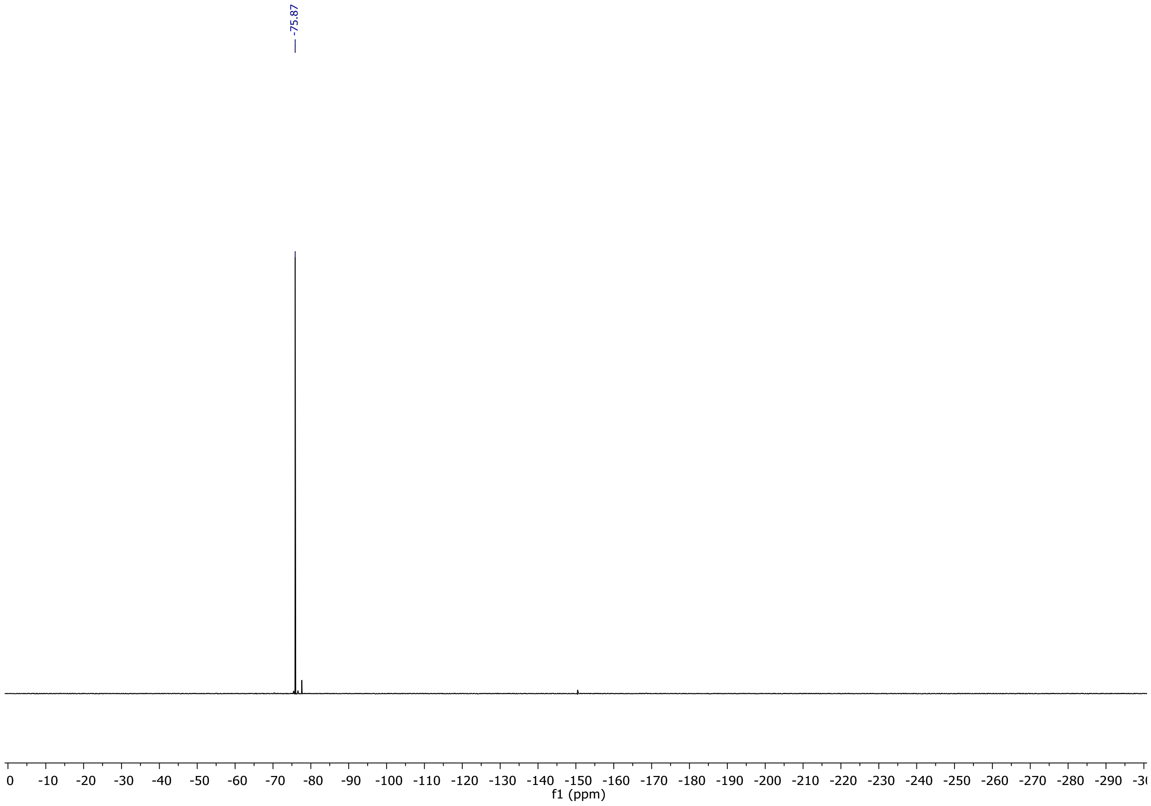

^{1}H NMR compound **3s** ($CDCl_3$, 300 MHz, 25 °C)

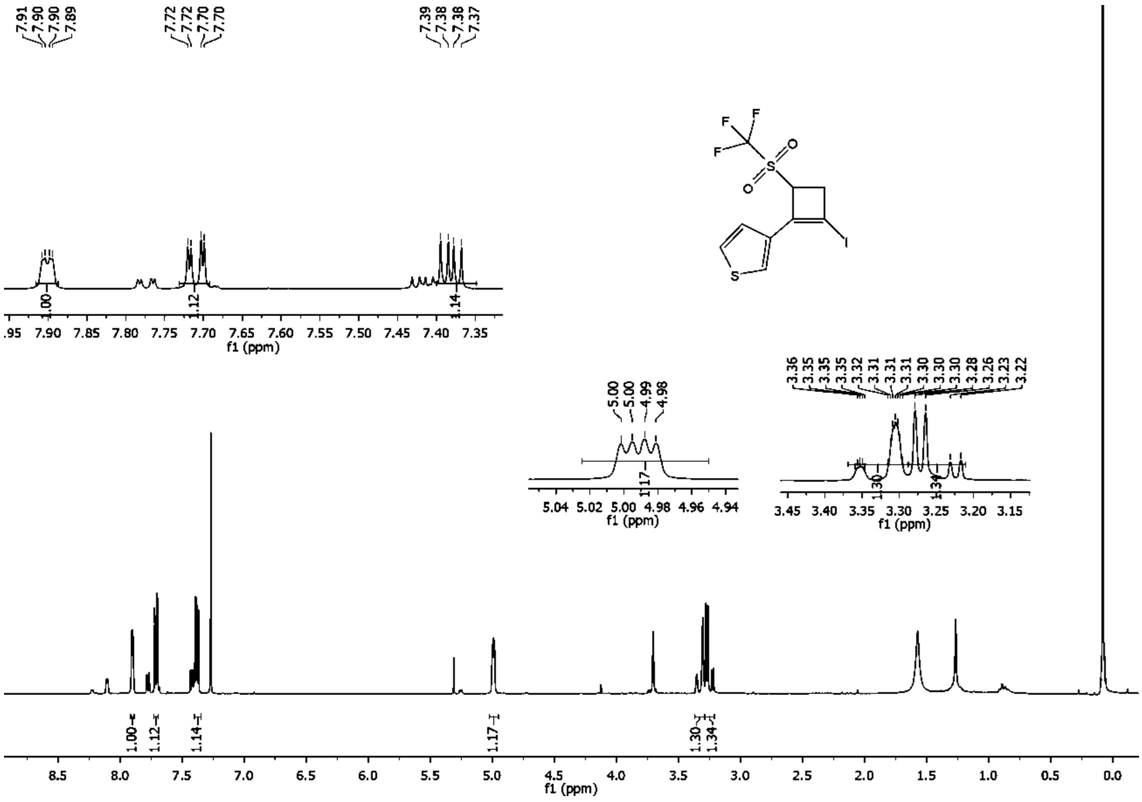

^{13}C NMR compound **3s** ($CDCl_3$, 75 MHz, 25 °C)

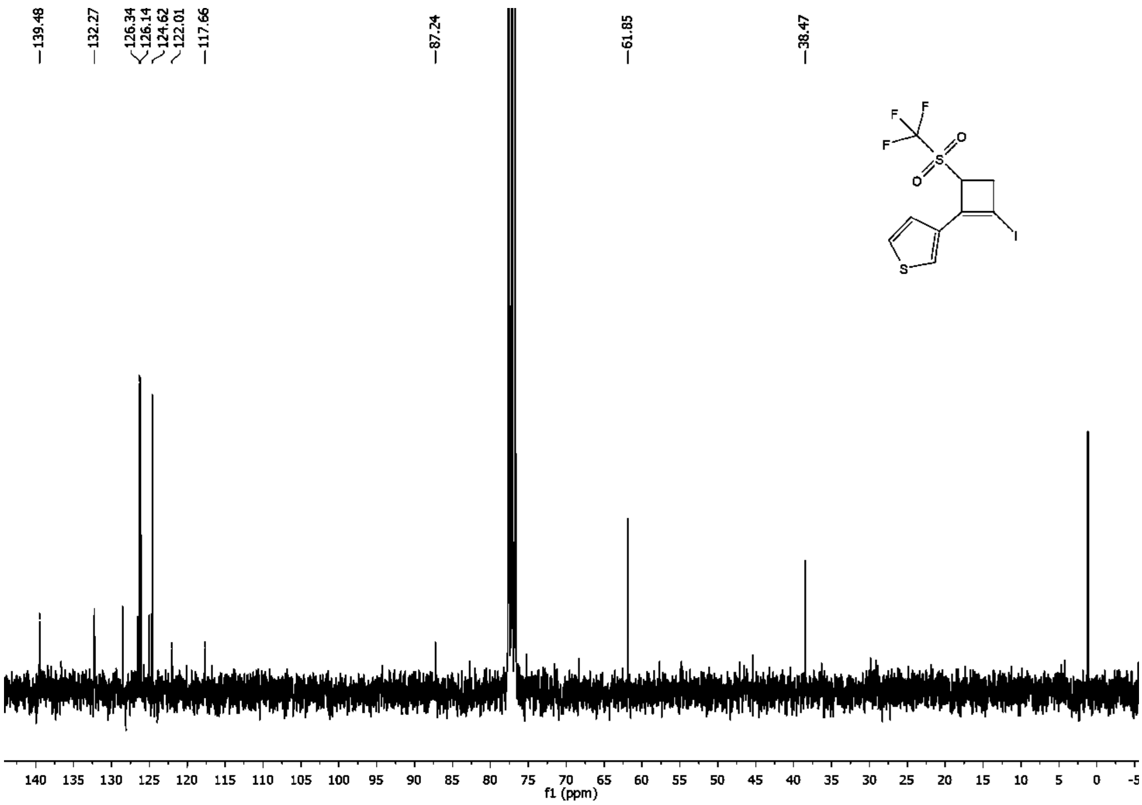

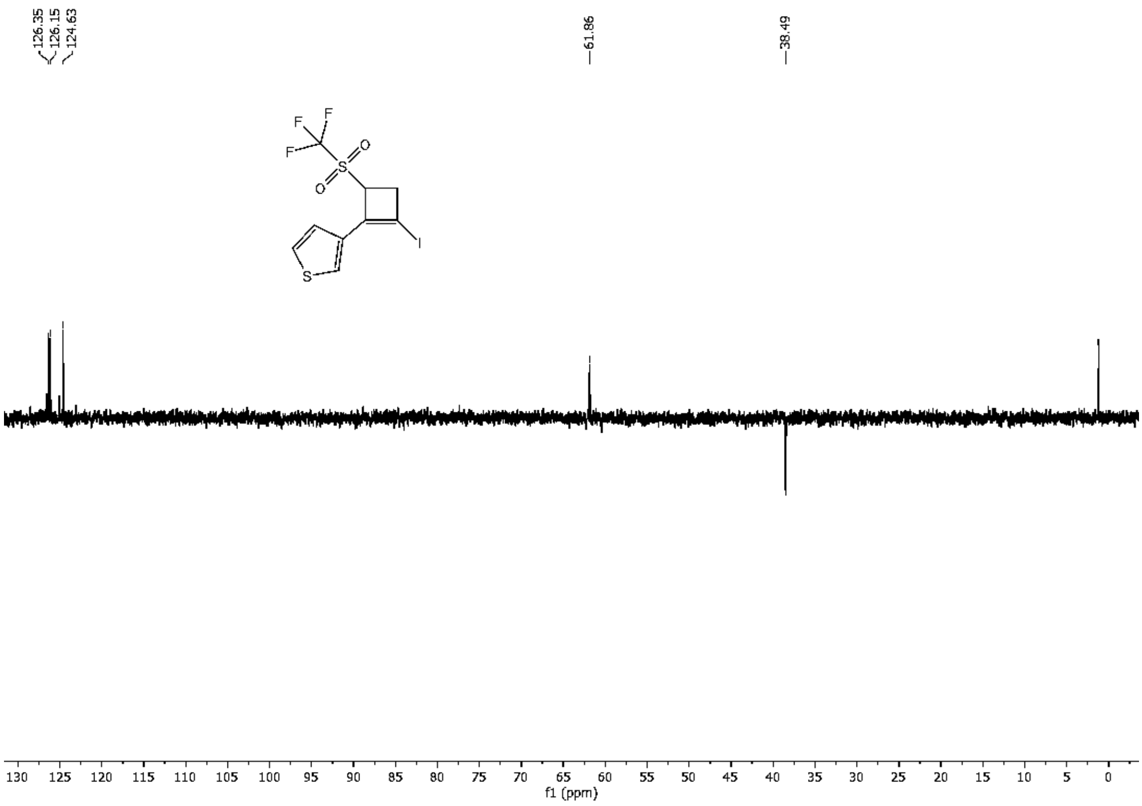

^{19}F NMR compound **3s** ($CDCl_3$, 282 MHz, 25 °C)

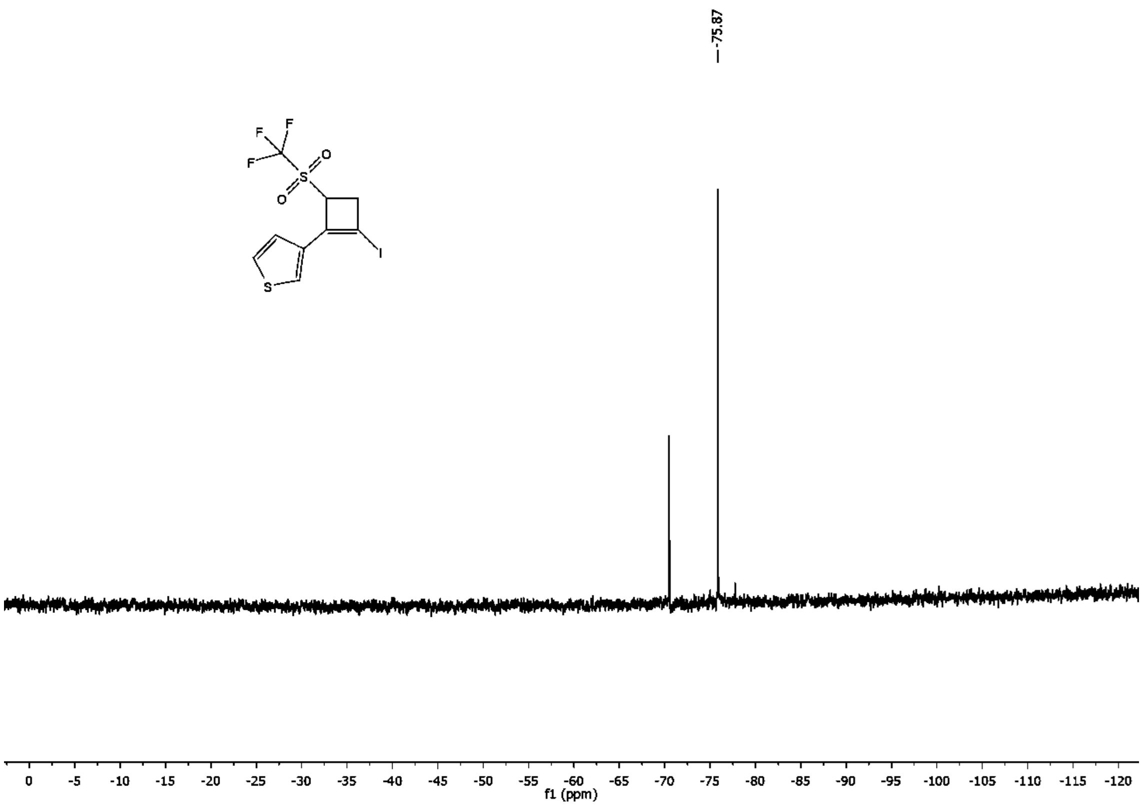

^{1}H NMR compound **3t** ($CDCl_3$, 300 MHz, 25 °C)

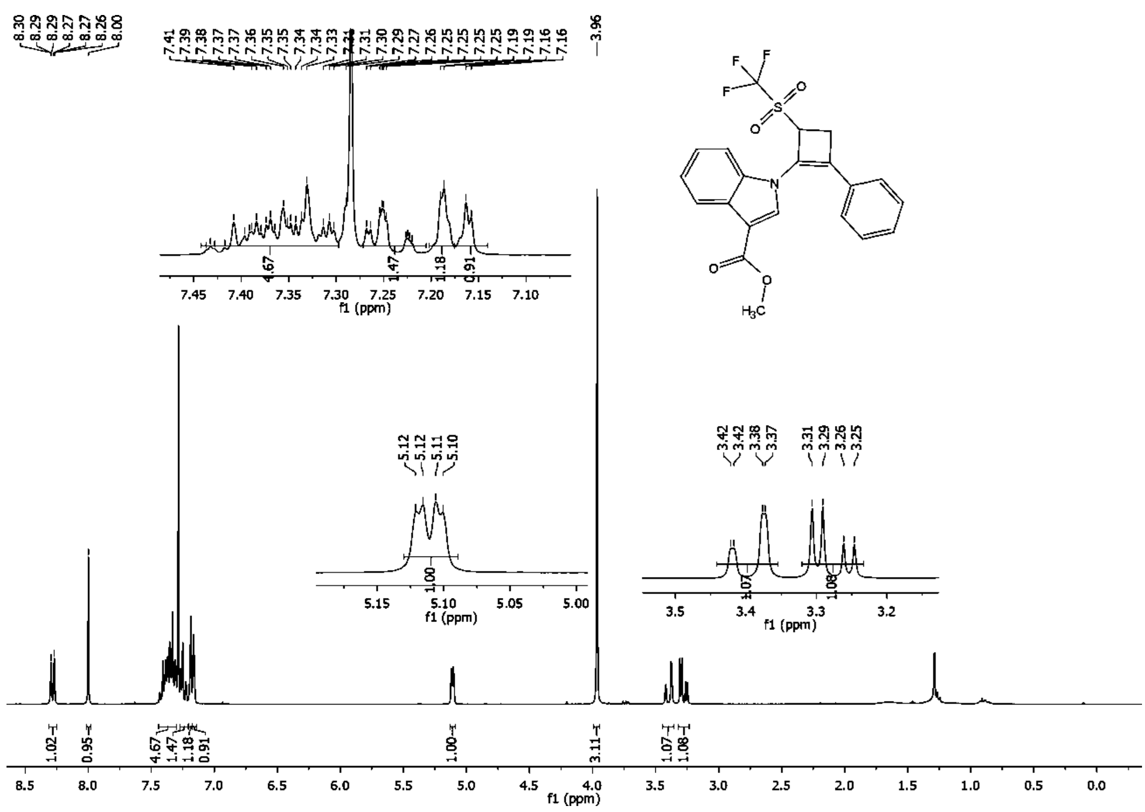

^{13}C NMR compound **3t** ($CDCl_3$, 75 MHz, 25 °C)

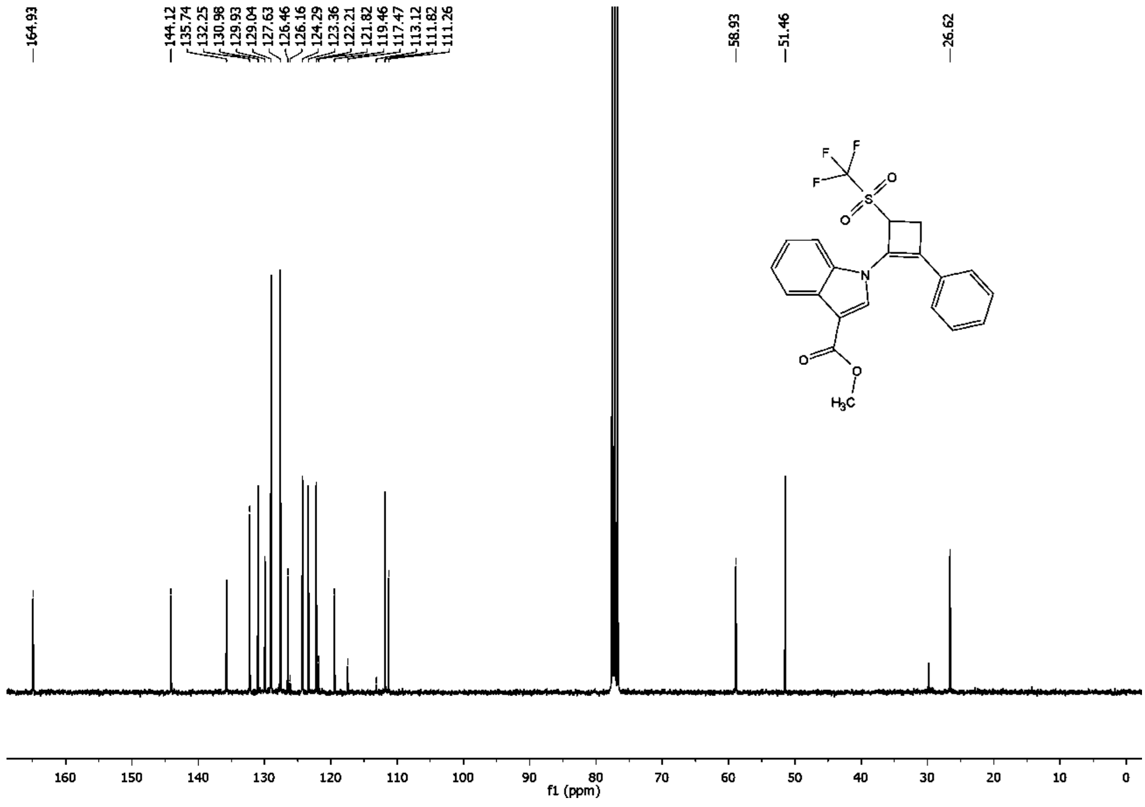

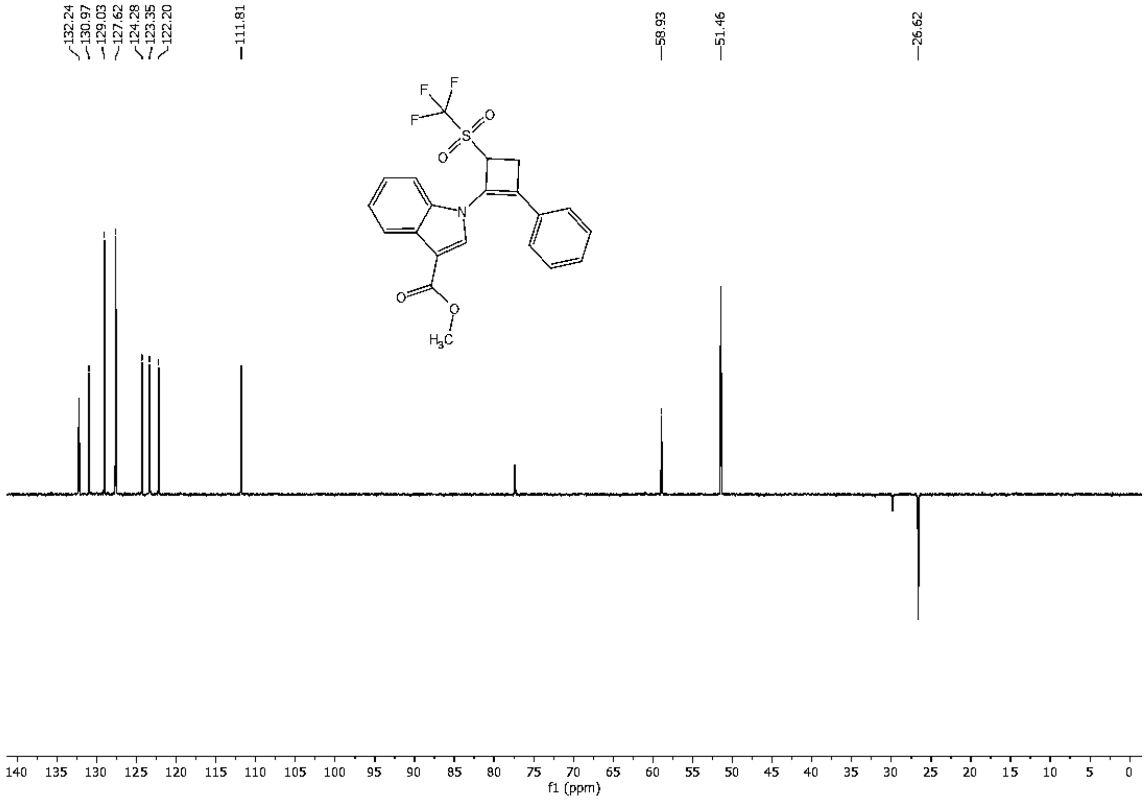

^{19}F NMR compound **3t** ($CDCl_3$, 282 MHz, 25 °C)

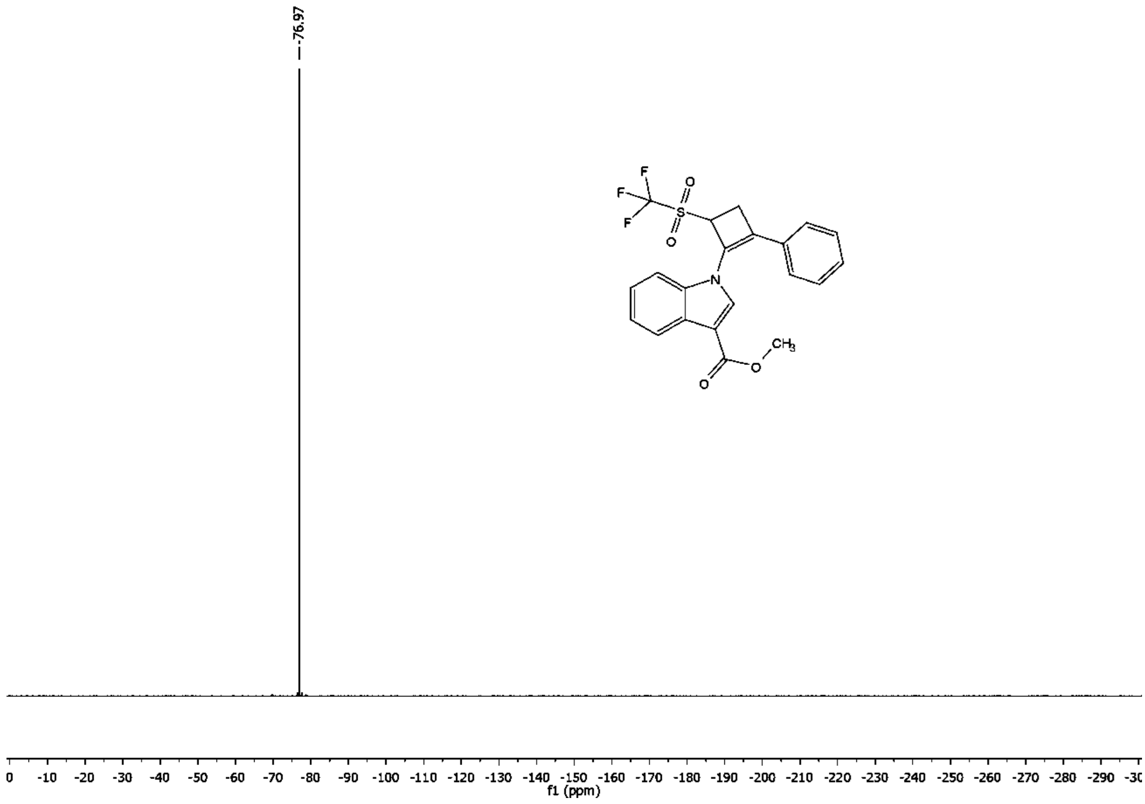

^{1}H NMR compound **3u** ($CDCl_3$, 300 MHz, 25 °C)

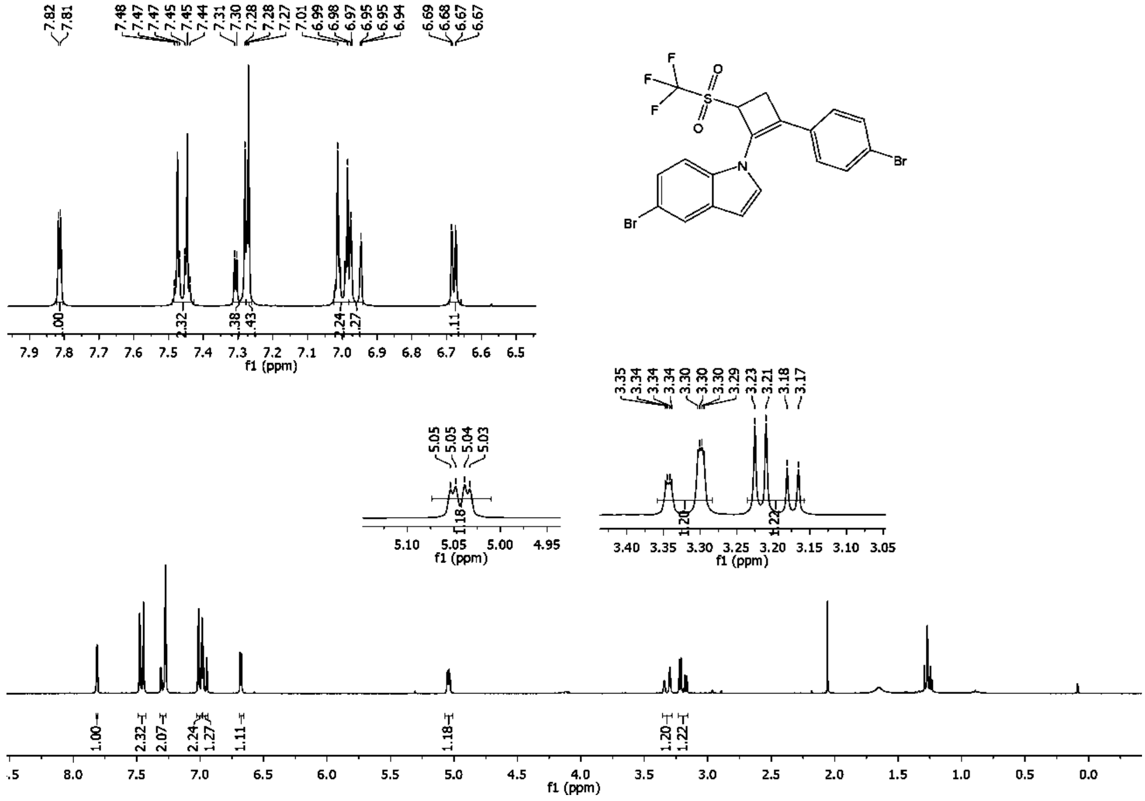

^{13}C NMR compound **3u** ($CDCl_3$, 75 MHz, 25 °C)

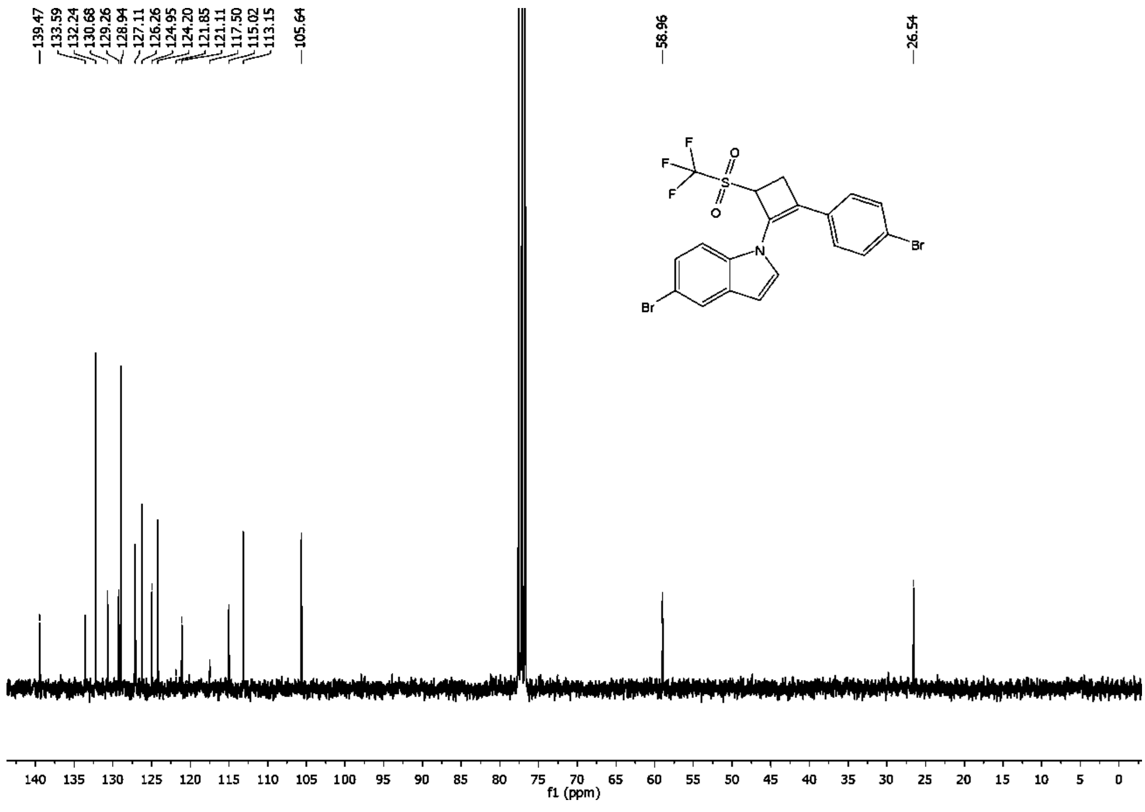

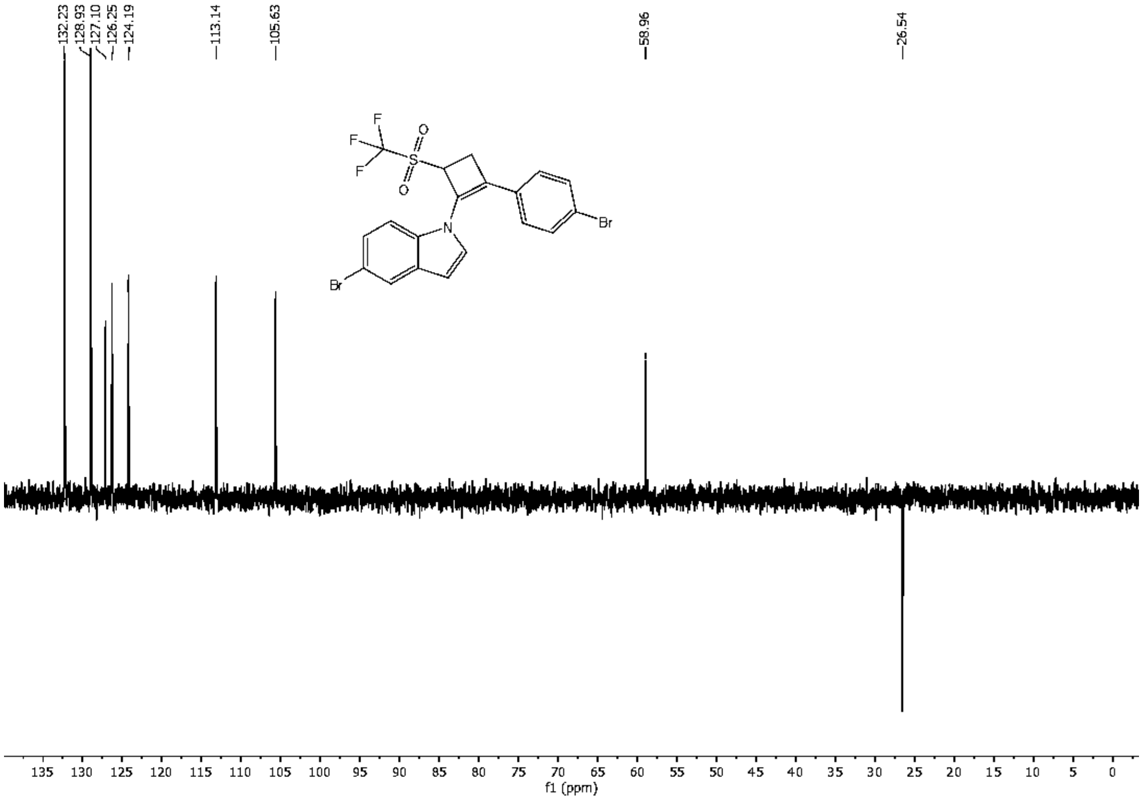

^{19}F NMR compound **3u** ($CDCl_3$, 282 MHz, 25 ^oC)

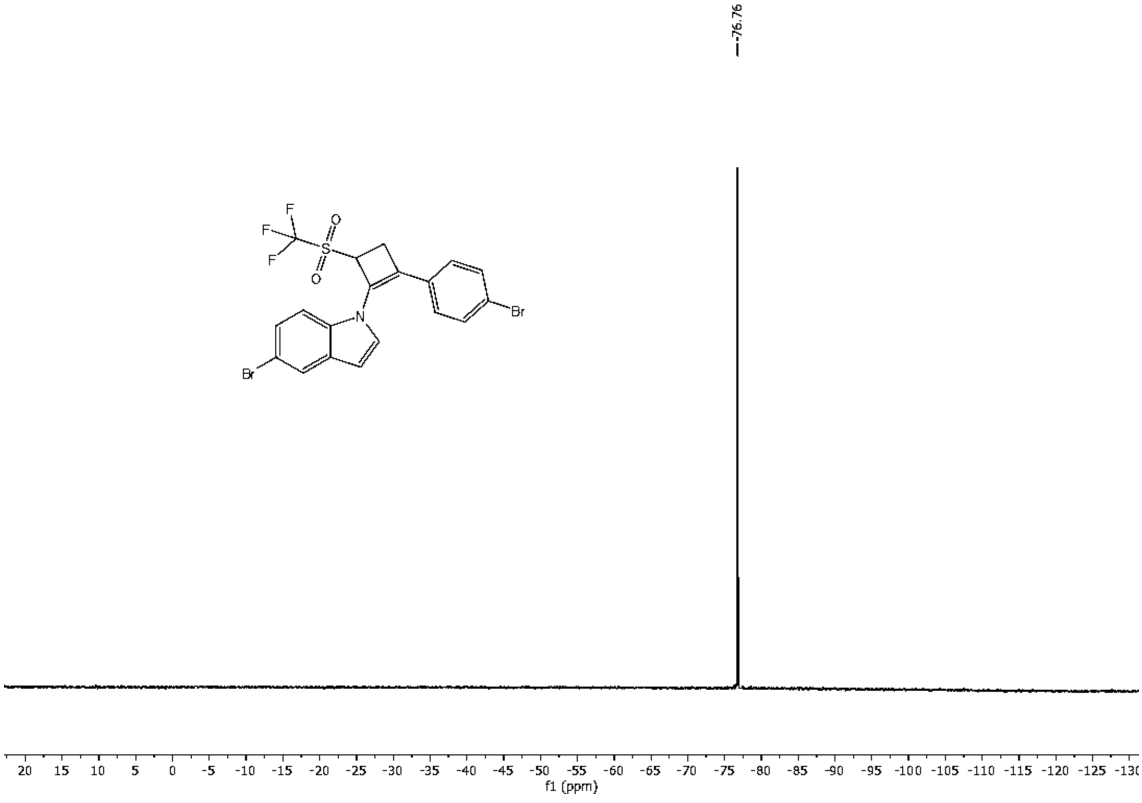

^{1}H NMR compound **3u-d₂** ($CDCl_3$, 300 MHz, 25 ^{o}C)

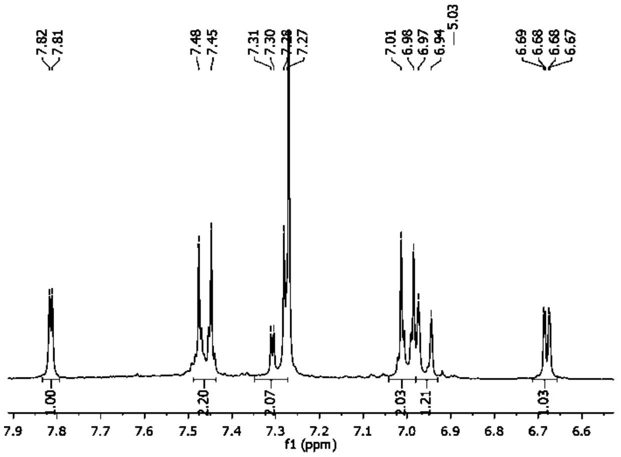

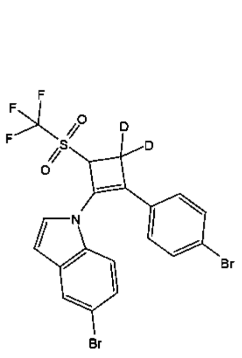

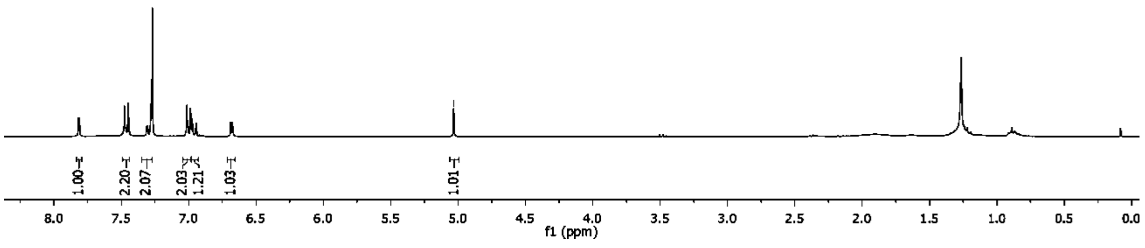

^{13}C NMR compound **3u-d₂** ($CDCl_3$, 176 MHz, 25 ^{o}C)

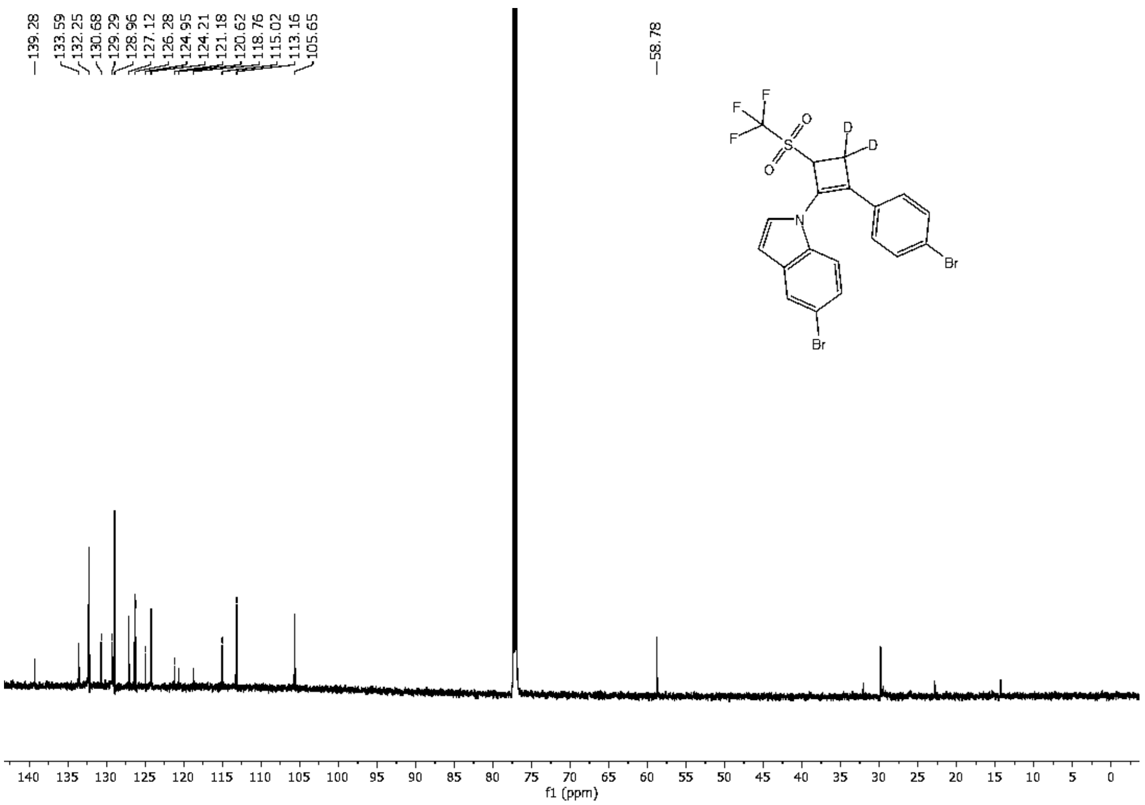

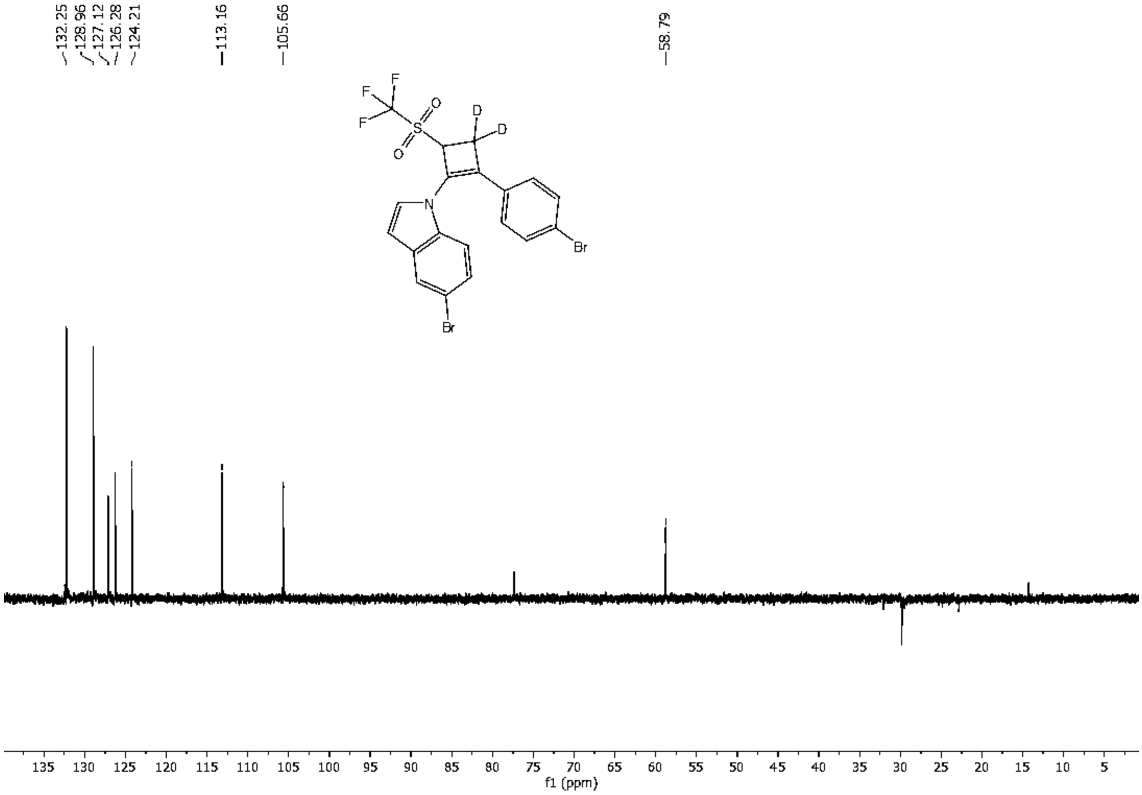

^{19}F NMR compound **3u-d$_2$** ($CDCl_3$, 282 MHz, 25 ^{o}C)

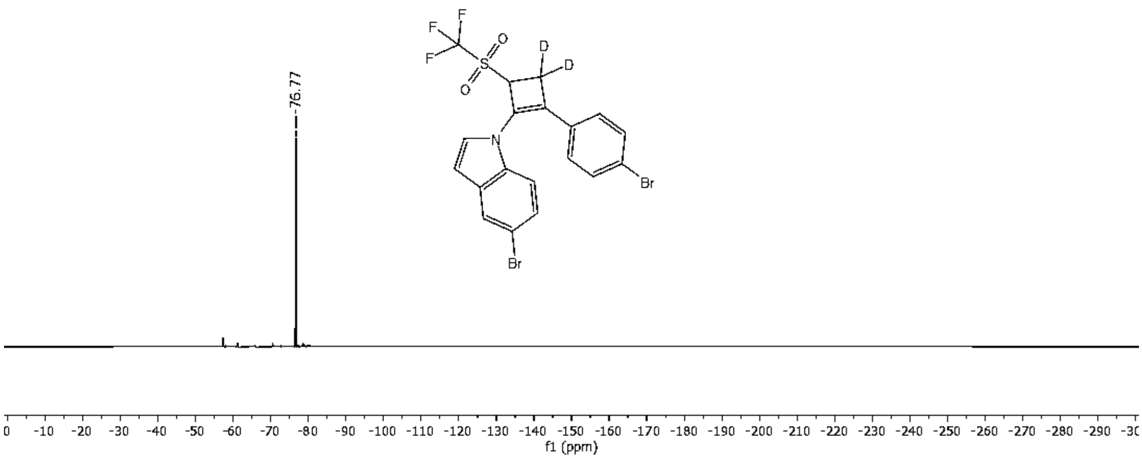

^{2}H NMR compound **3u-d₂** (CDCl$_3$, 107 MHz, 25 °C)

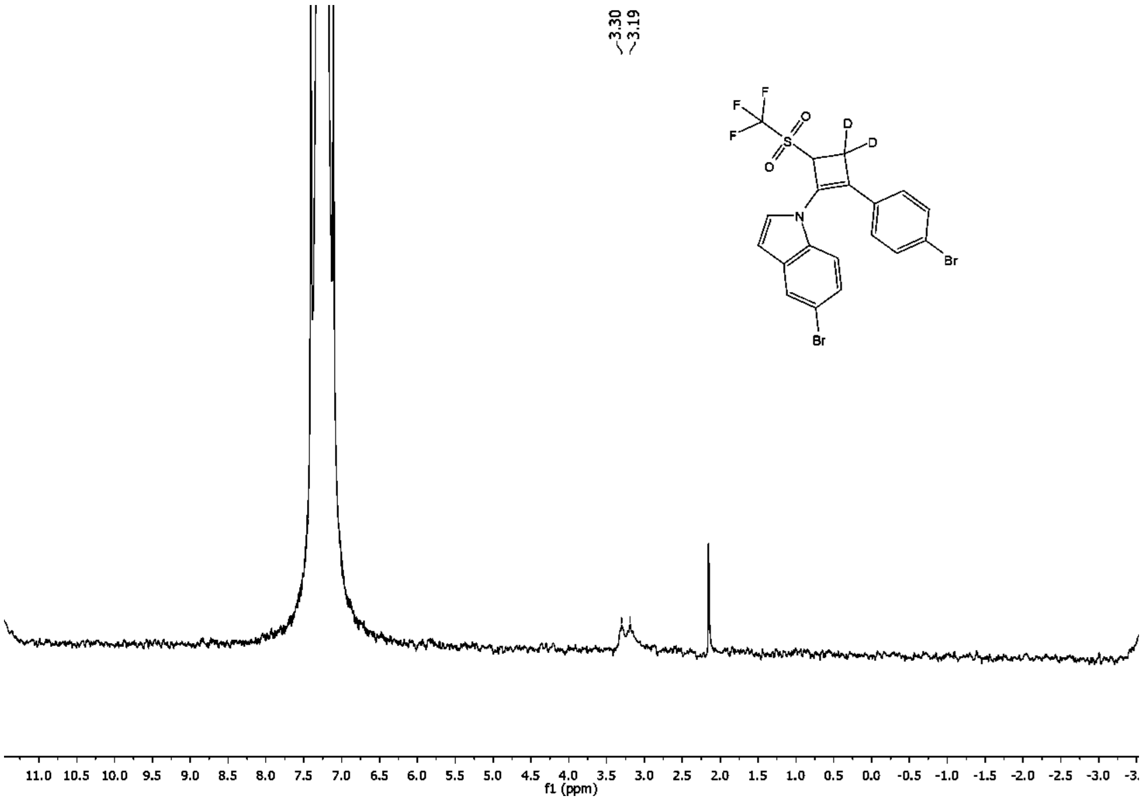

^{1}H NMR compound **3v** ($CDCl_3$, 300 MHz, 25 °C)

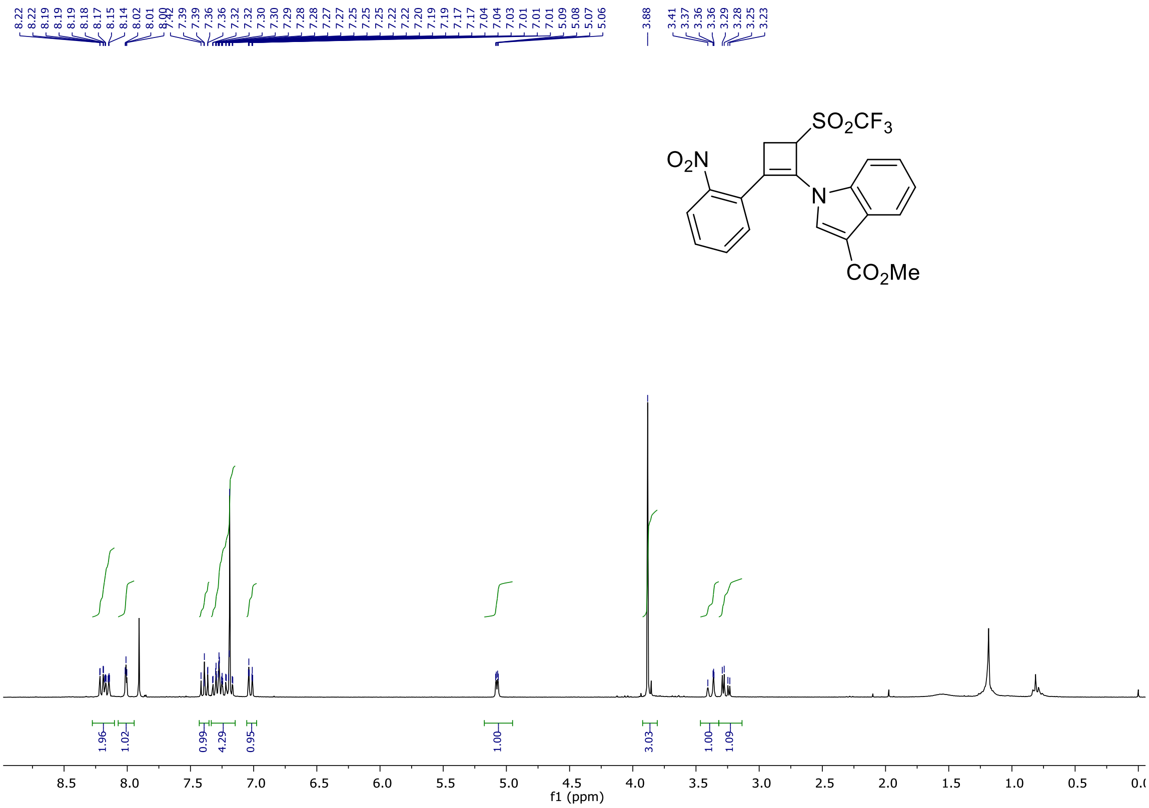

^{13}C NMR compound **3v** ($CDCl_3$, 75 MHz, 25 °C)

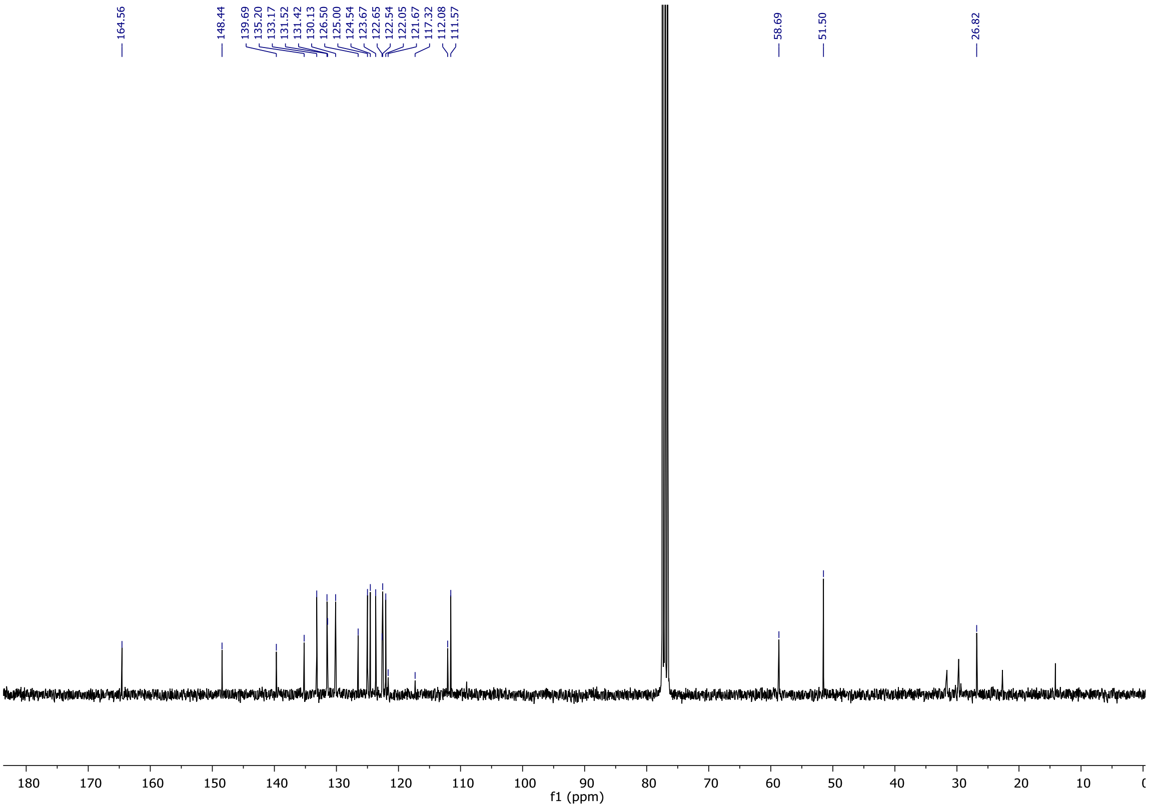

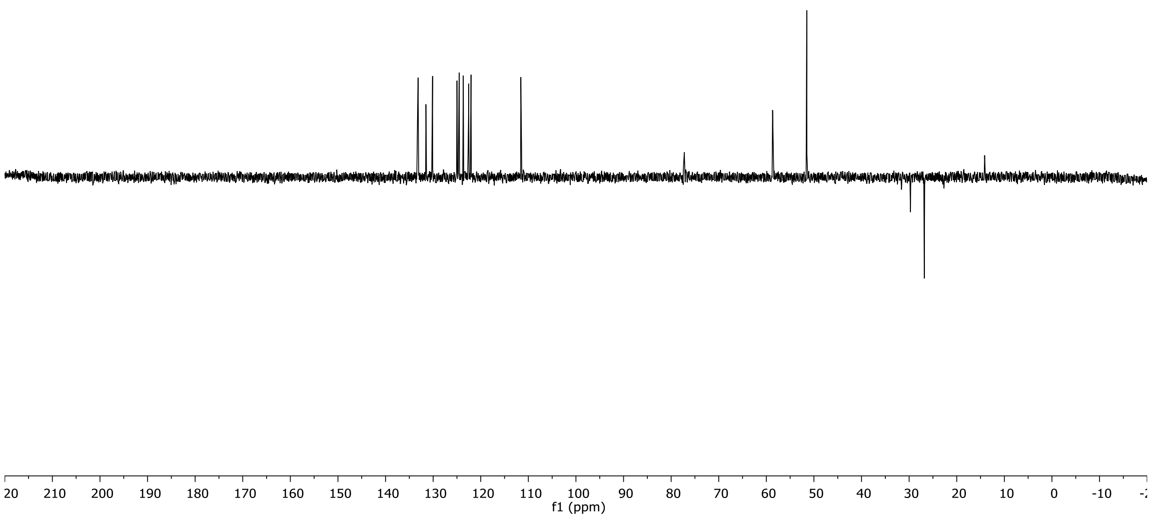
20 210 200 190 180 170 160 150 140 130 120 110 100 90 80 70 60 50 40 30 20 10 0 -10
f1 (ppm)

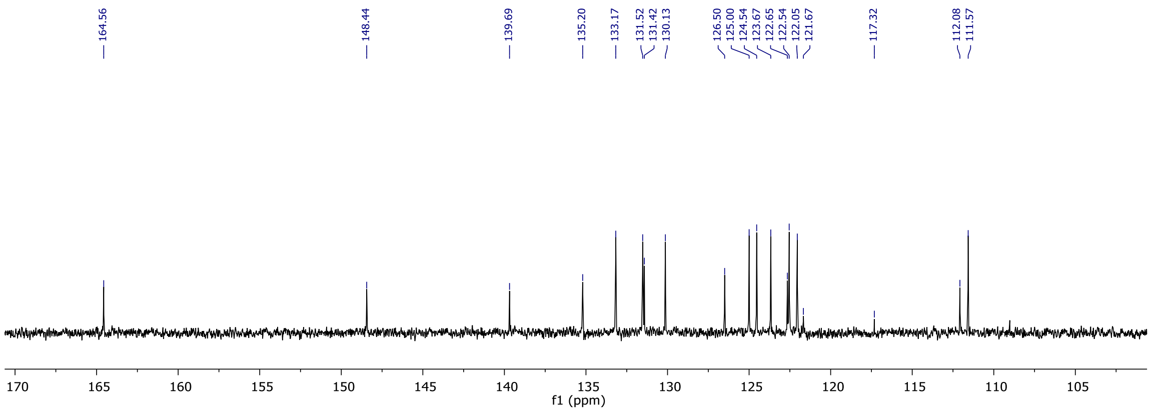
164.56
148.44
139.69
135.20
133.17
131.52
131.42
130.13
126.50
125.00
124.54
123.67
122.65
122.54
122.05
121.67
117.32
112.08
111.57
170 165 160 155 150 145 140 135 130 125 120 115 110 105
f1 (ppm)

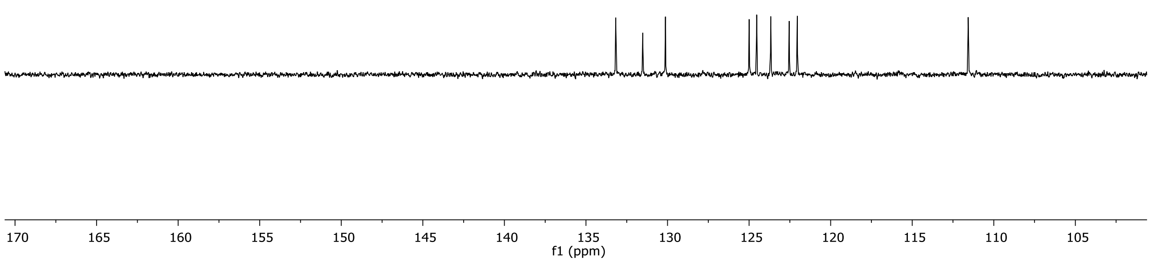
170 165 160 155 150 145 140 135 130 125 120 115 110 105
f1 (ppm)

^{19}F NMR compound **3v** ($CDCl_3$, 282 MHz, 25 °C)

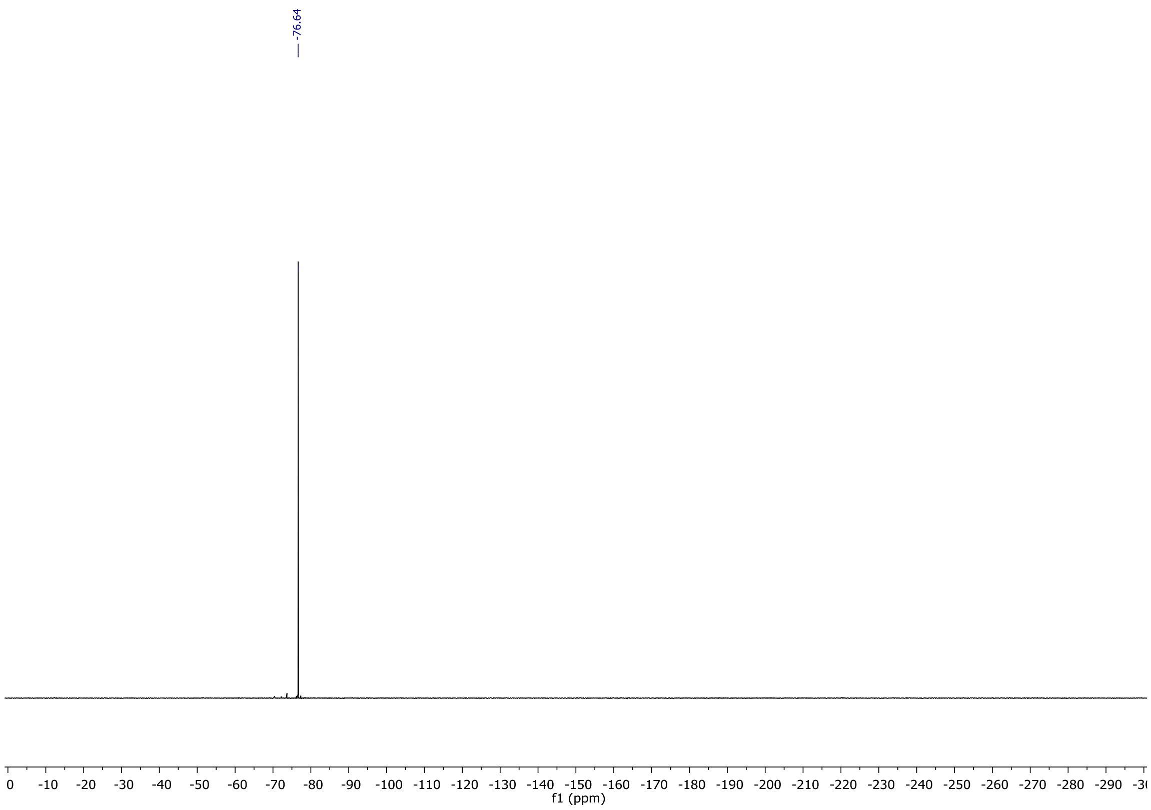

^{1}H NMR compound **3w** ($CDCl_3$, 300 MHz, 25 ^{o}C)

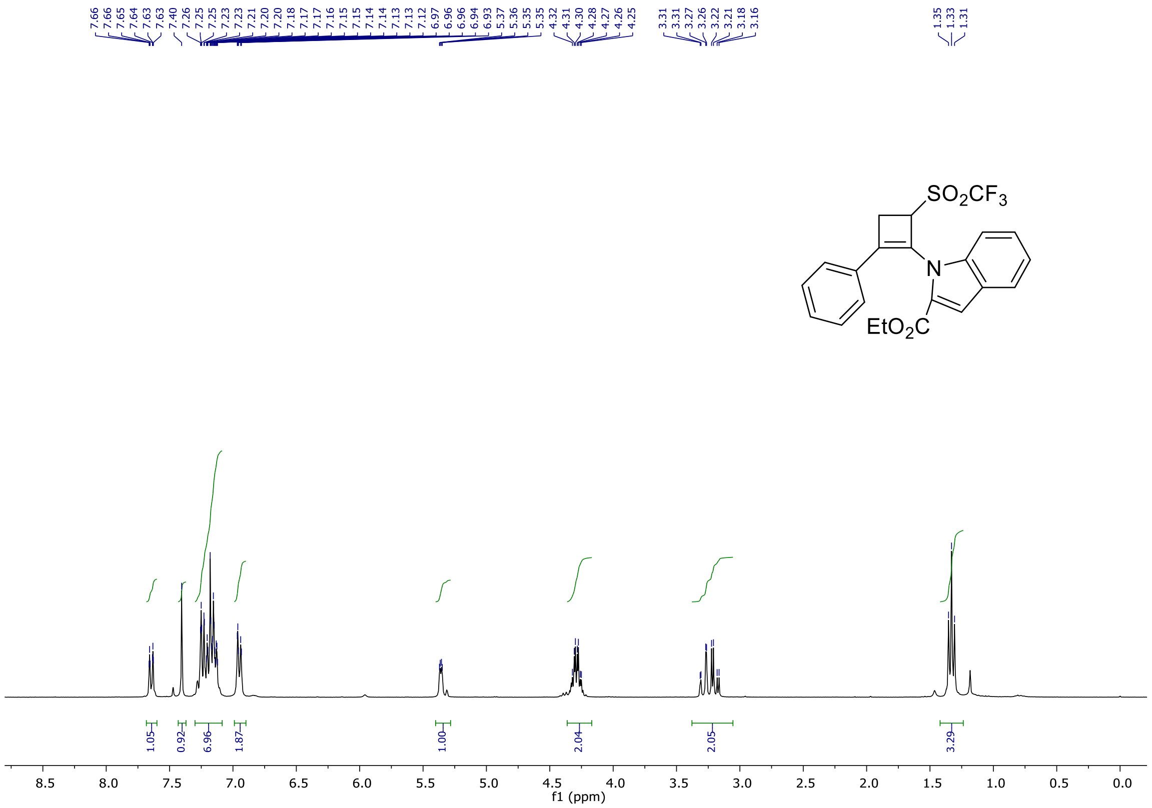

^{13}C NMR compound **3w** ($CDCl_3$, 75 MHz, 25 ^{o}C)

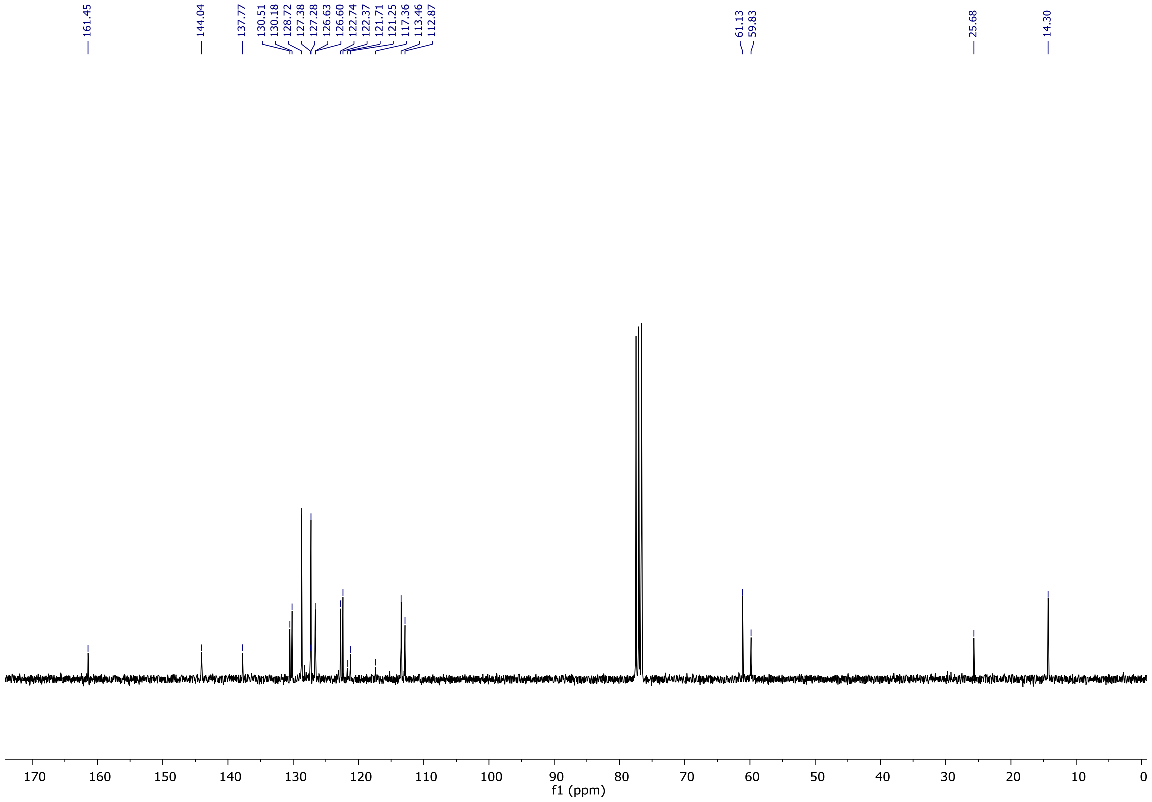

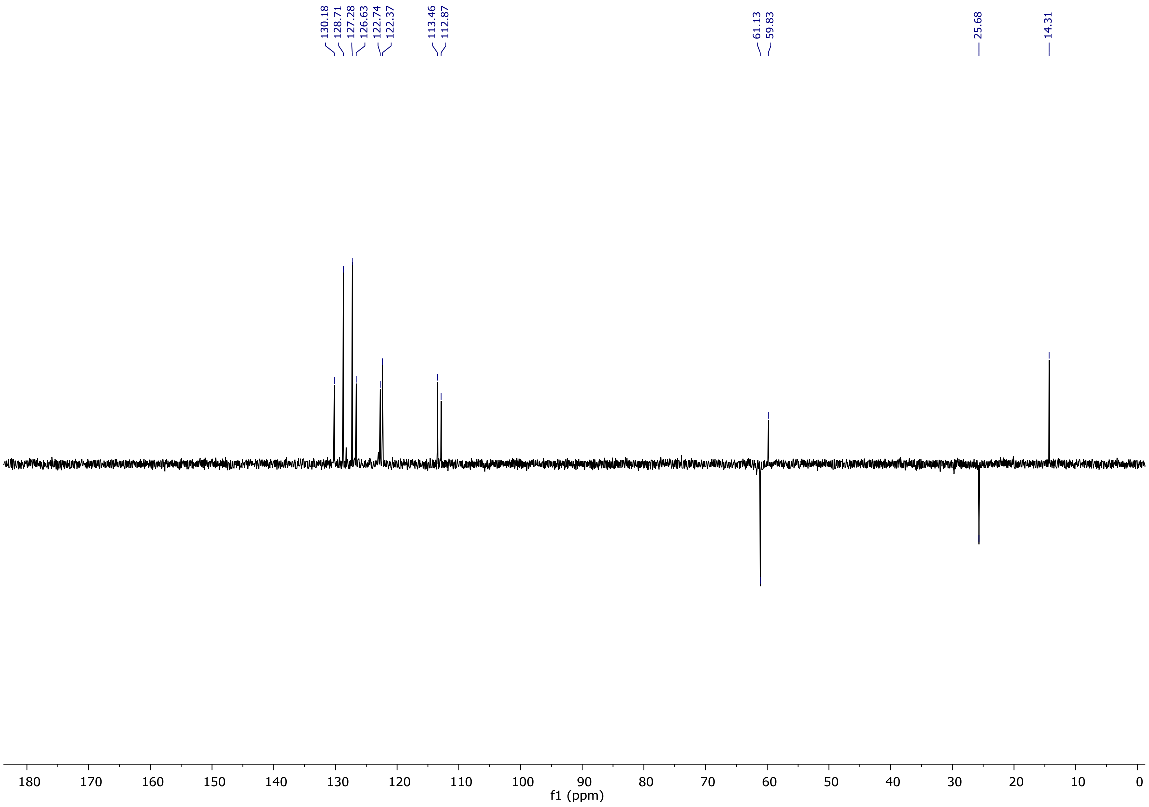
130.18
128.71
127.28
126.63
122.74
122.37
113.46
112.87
61.13
59.83
25.68
14.31
f1 (ppm)

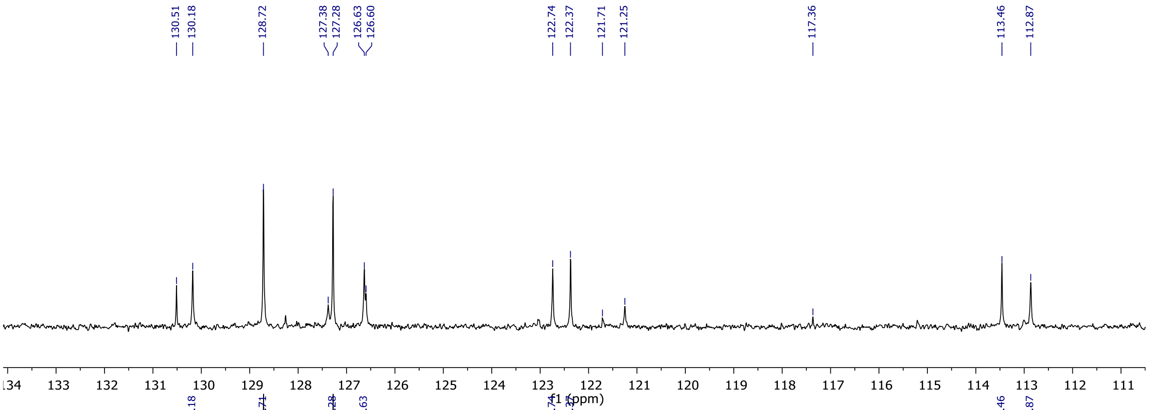
130.51
130.18
128.72
127.38
127.28
126.63
126.60
122.74
122.37
121.71
121.25
117.36
113.46
112.87
f1 (ppm)

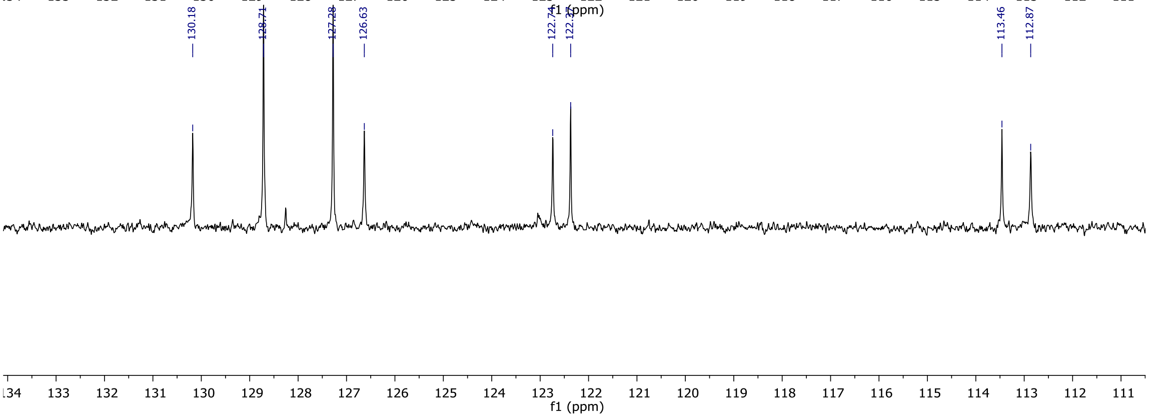
130.18
128.71
127.28
126.63
122.74
122.37
113.46
112.87
f1 (ppm)

^{19}F NMR compound **3w** ($CDCl_3$, 282 MHz, 25 °C)

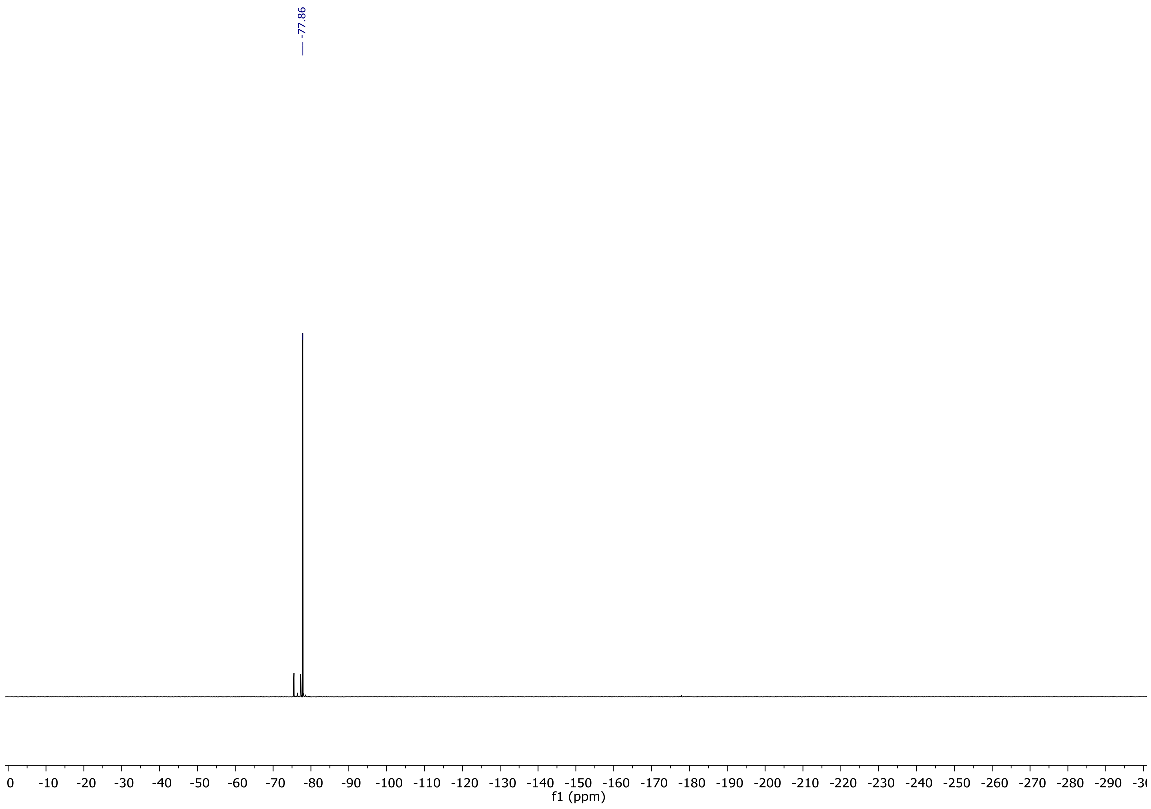

^{1}H NMR compound **3x** ($CDCl_3$, 300 MHz, 25 °C)

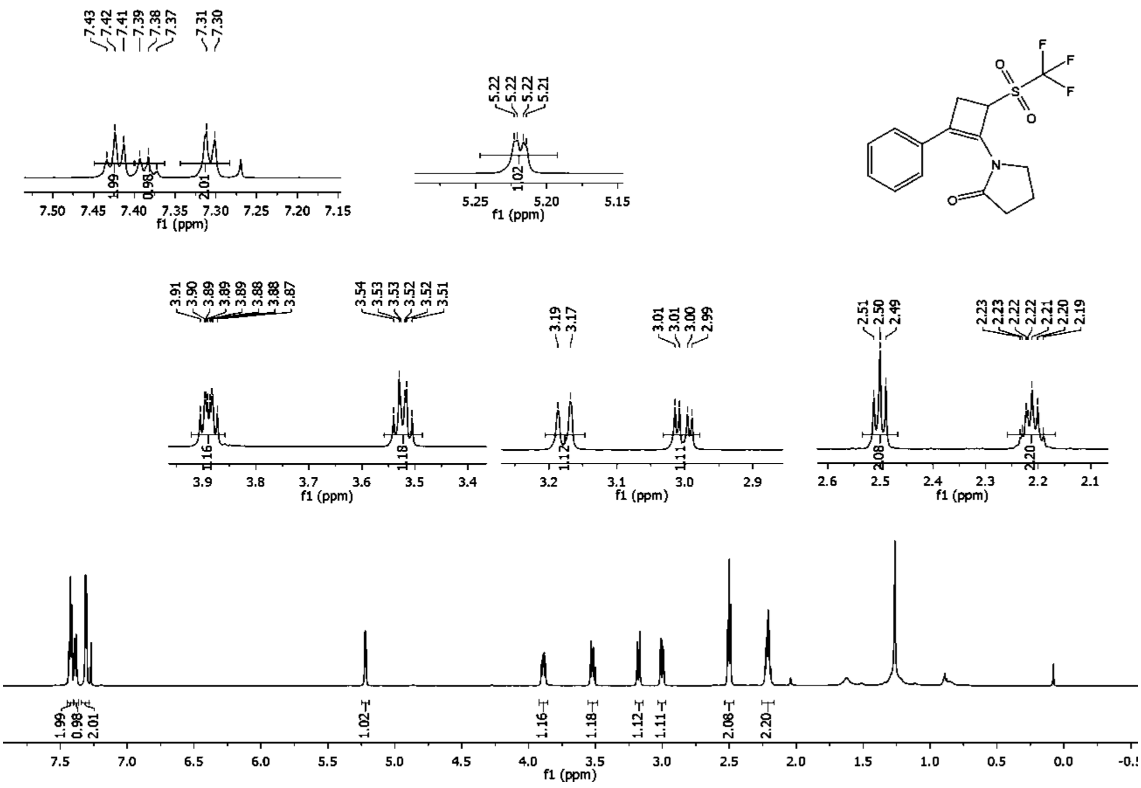

^{13}C NMR compound **3x** ($CDCl_3$, 176 MHz, 25 °C)

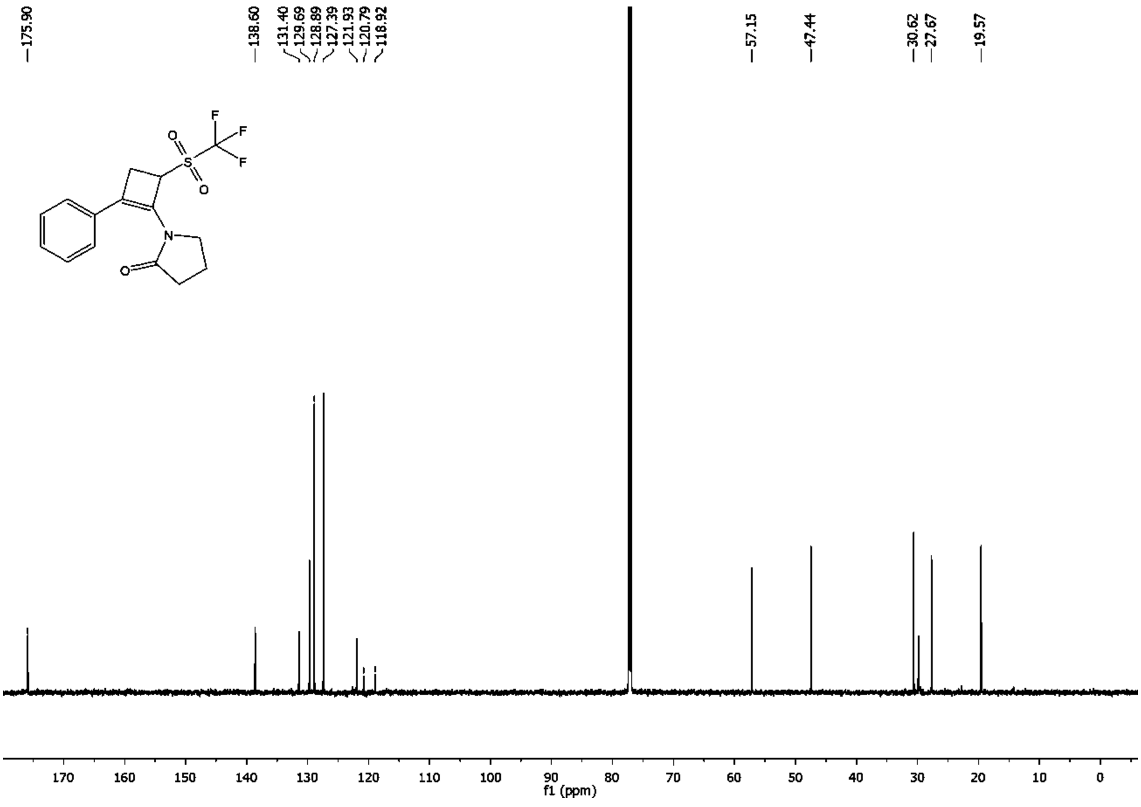

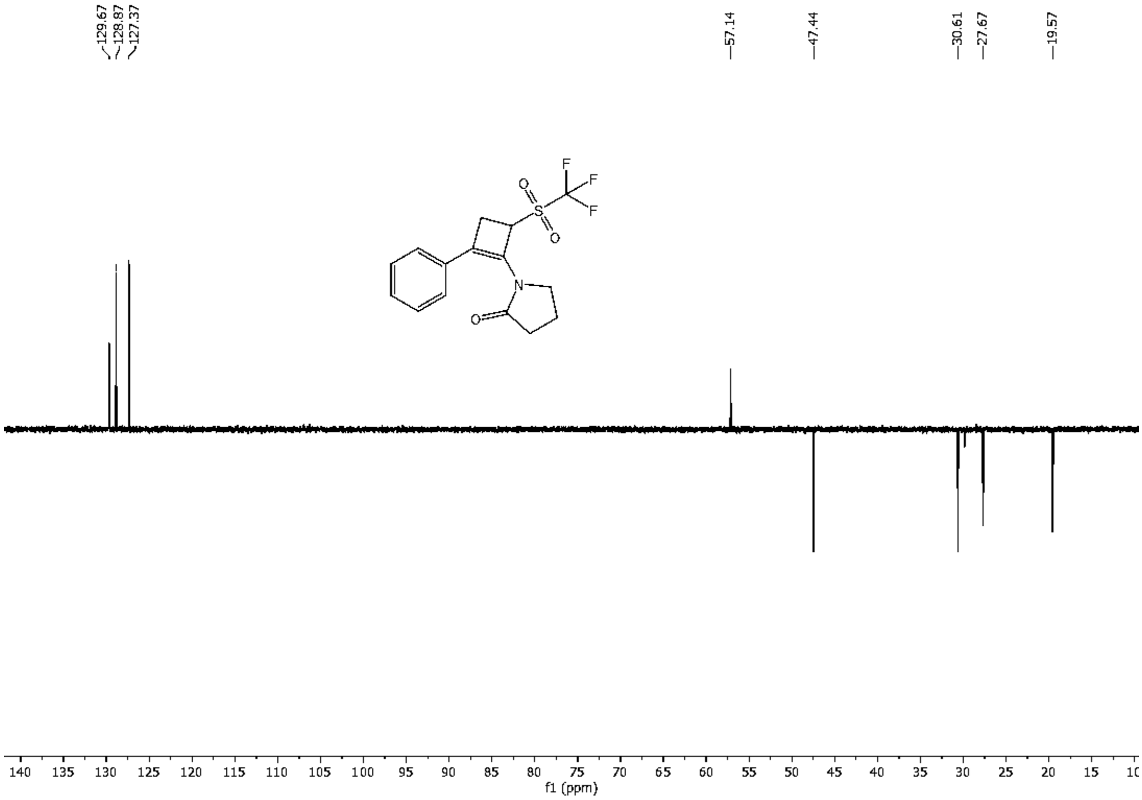

^{19}F NMR compound **3x** ($CDCl_3$, 282 MHz, 25 °C)

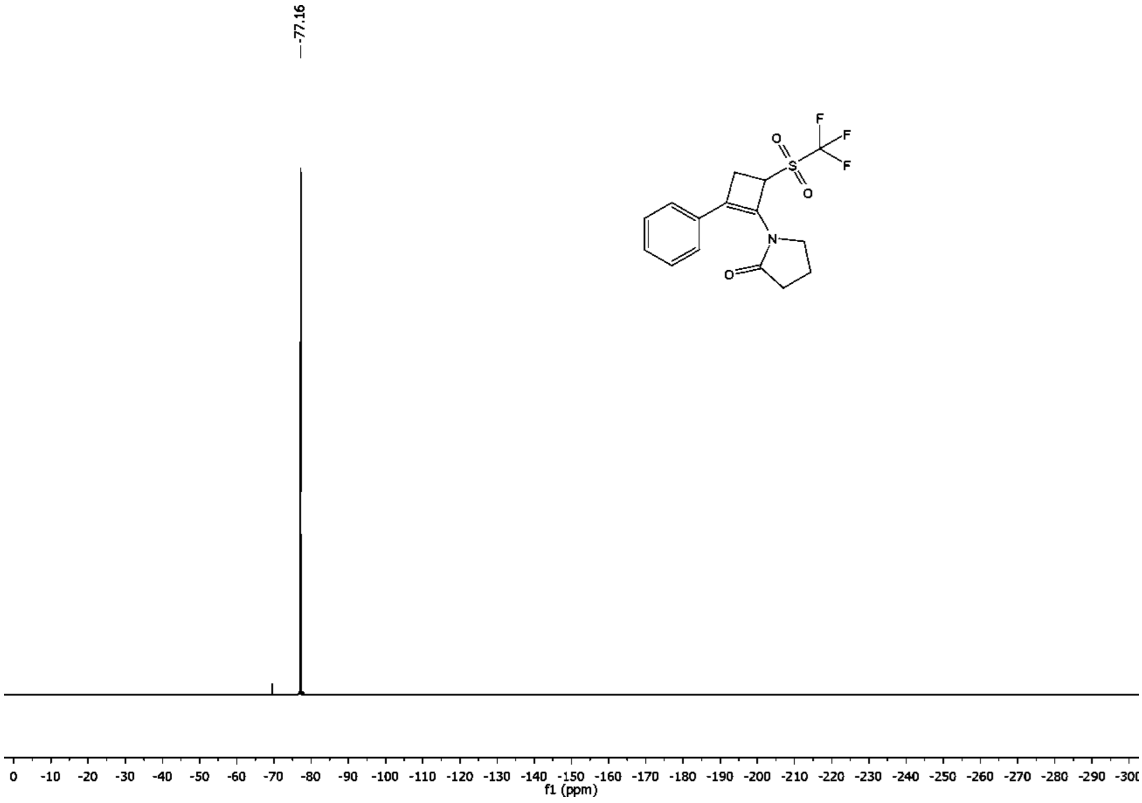

^{1}H NMR compound **3y** ($CDCl_3$, 300 MHz, 25 °C)

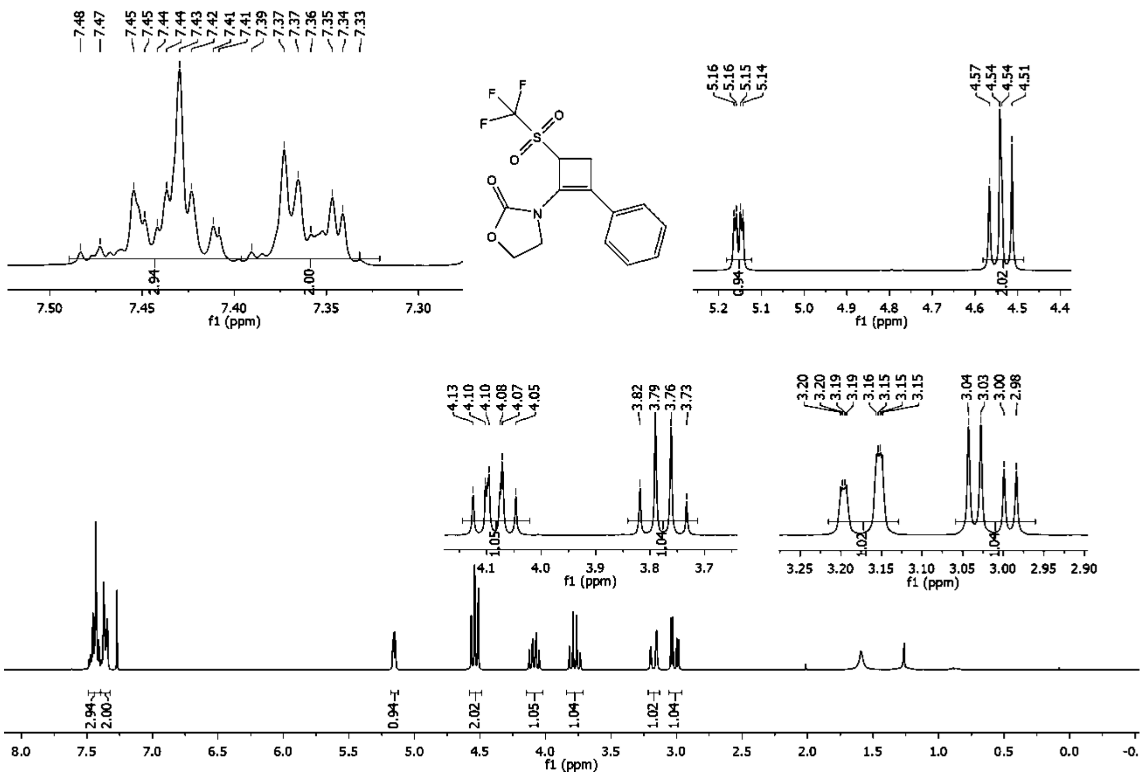

^{13}C NMR compound **3y** ($CDCl_3$, 75 MHz, 25 °C)

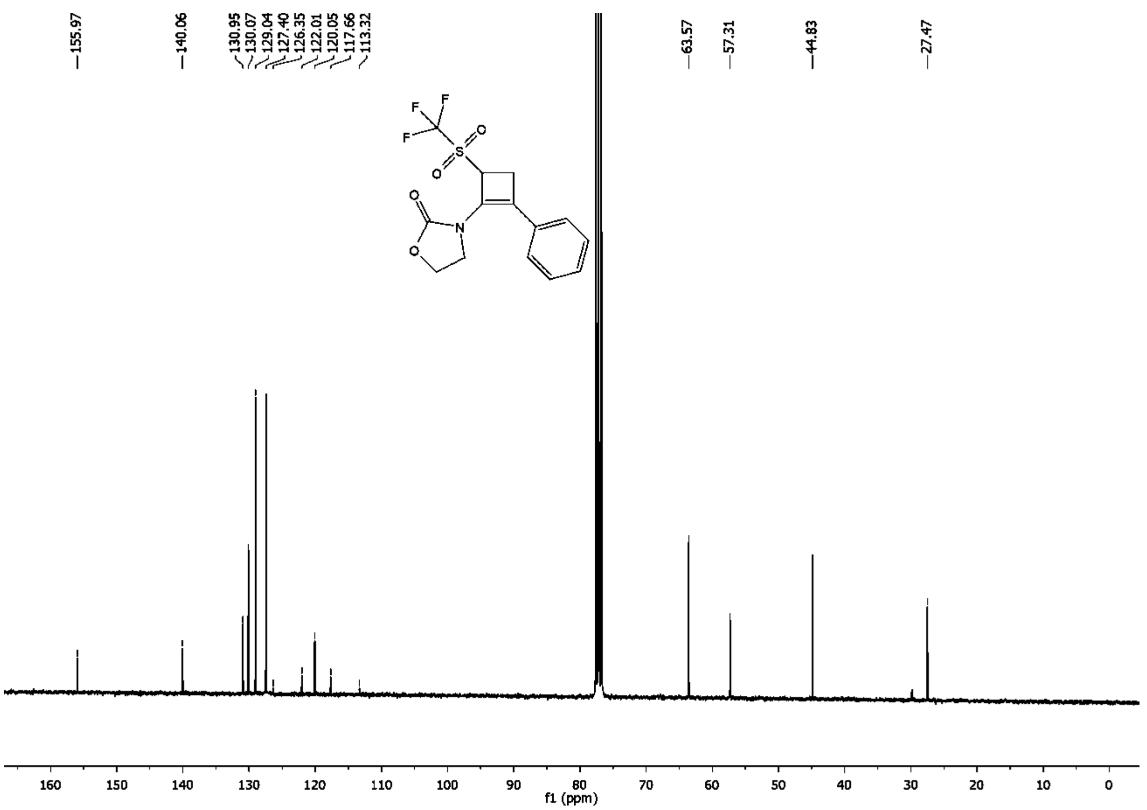

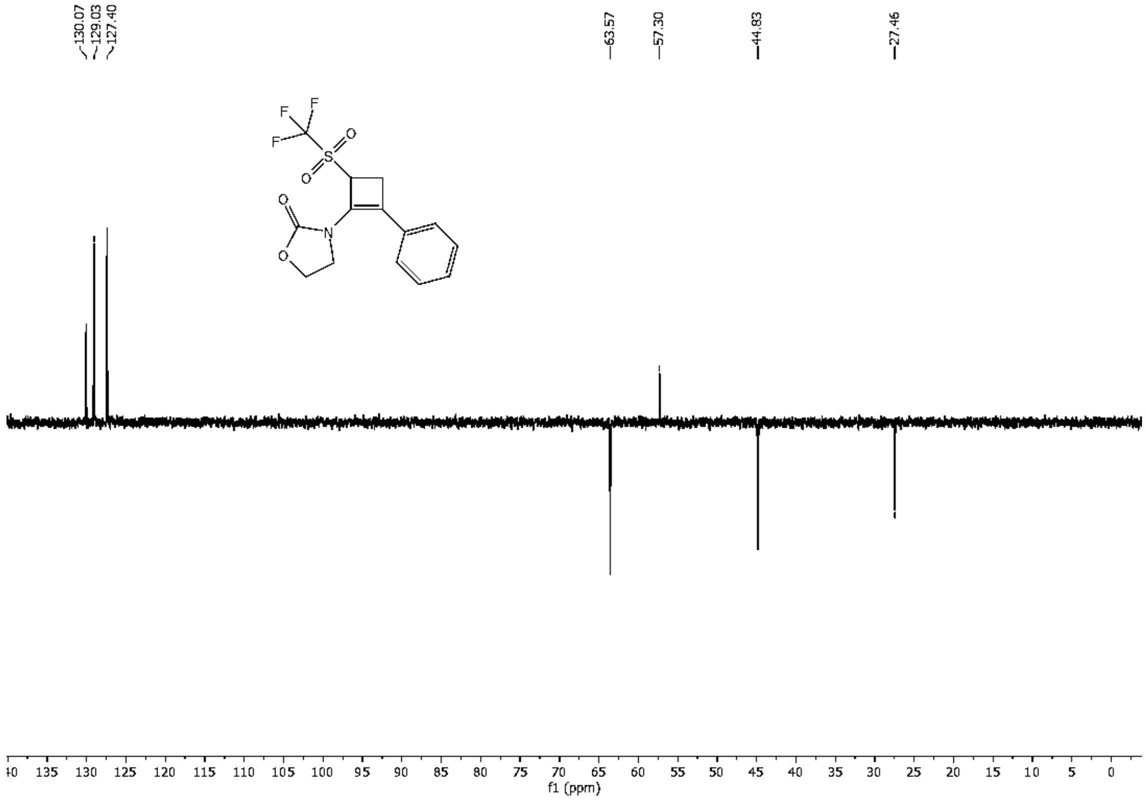

^{19}F NMR compound **3y** ($CDCl_3$, 282 MHz, 25 °C)

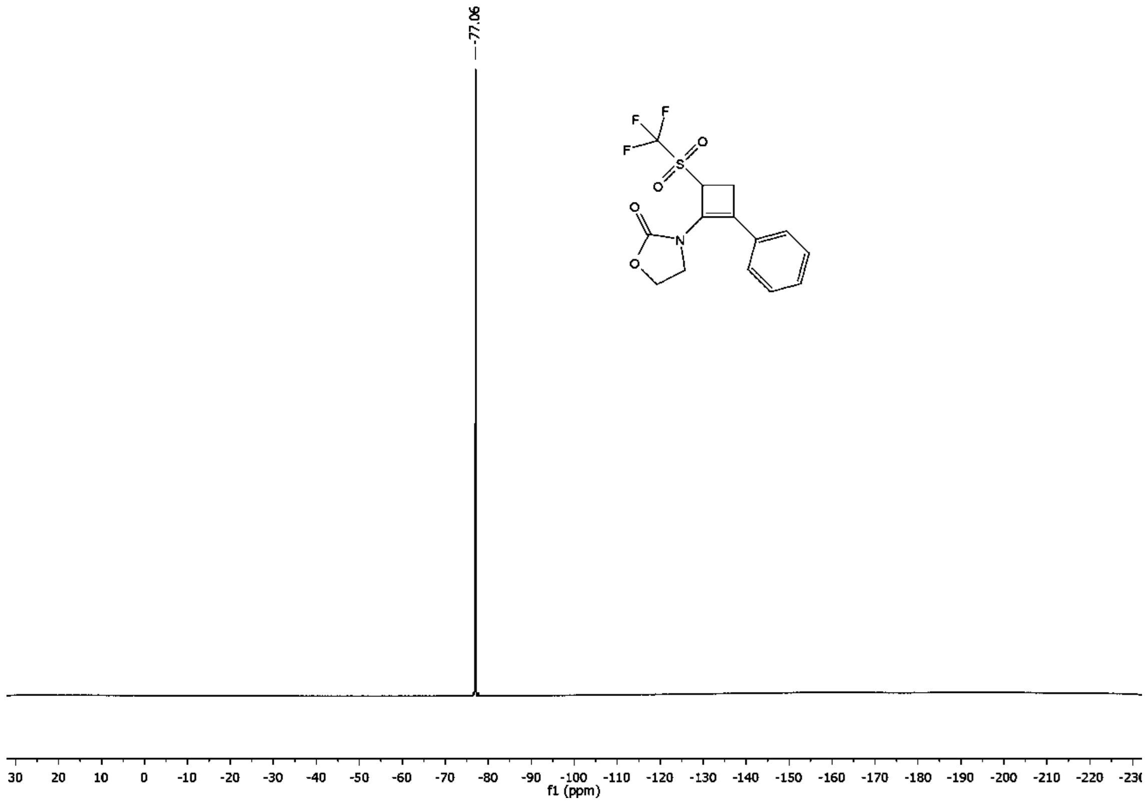

^{1}H NMR compound **3z** ($CDCl_3$, 300 MHz, 25 °C)

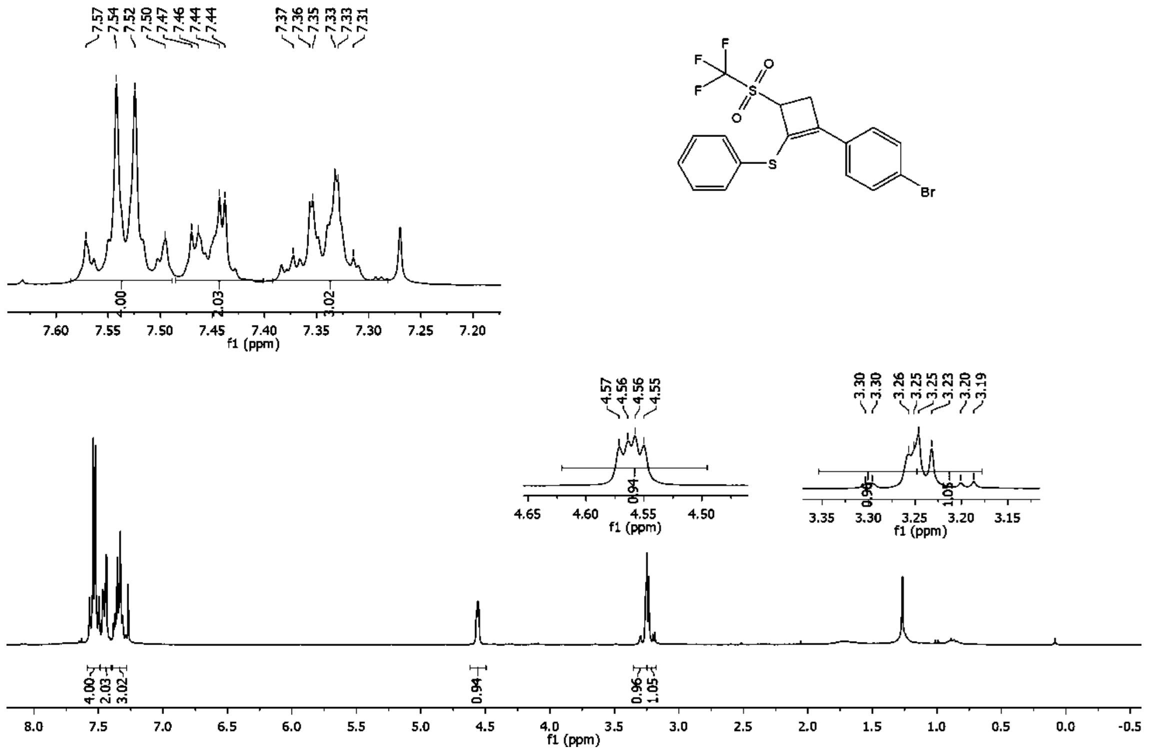

^{13}C NMR compound **3z** ($CDCl_3$, 75 MHz, 25 °C)

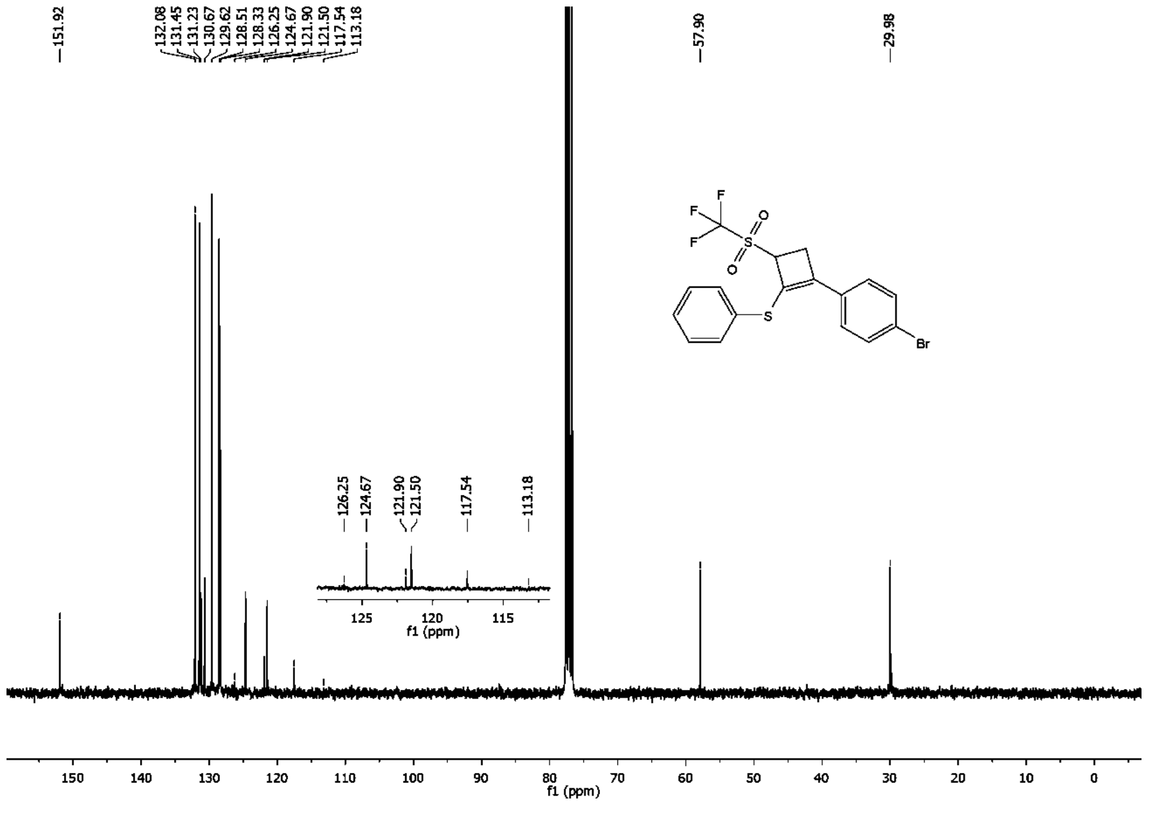

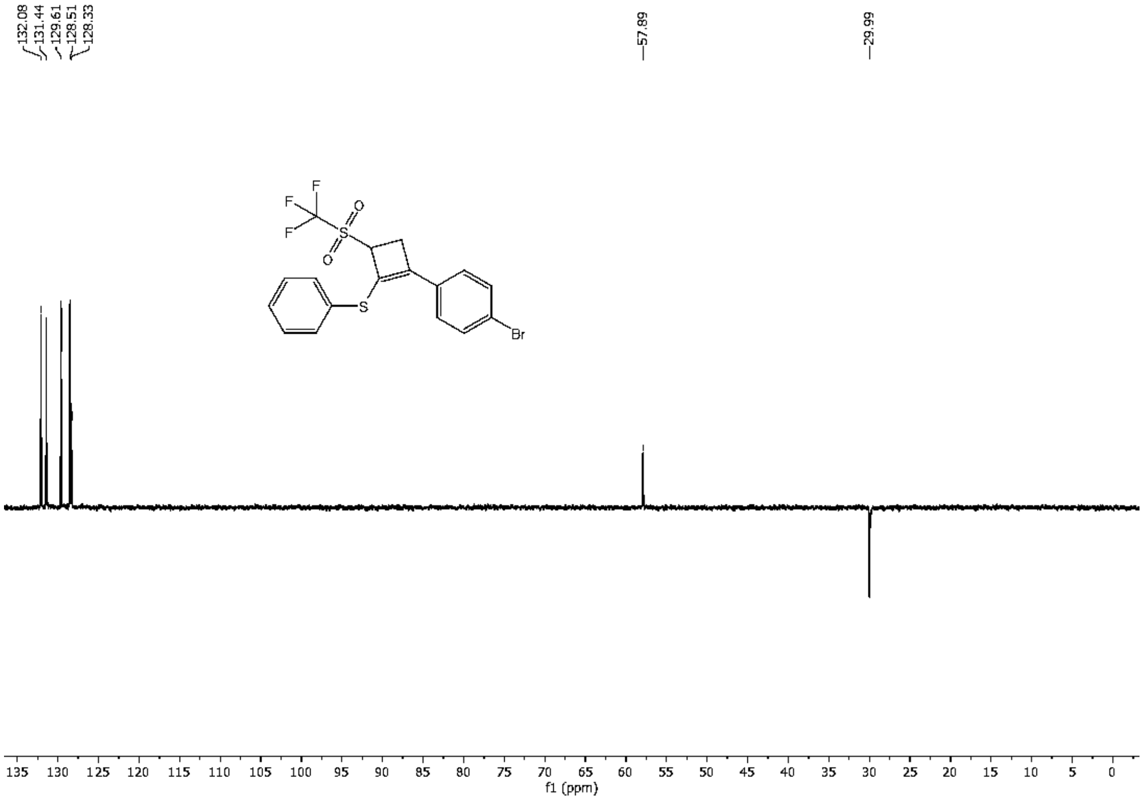

^{19}F NMR compound **3z** ($CDCl_3$, 282 MHz, 25 °C)

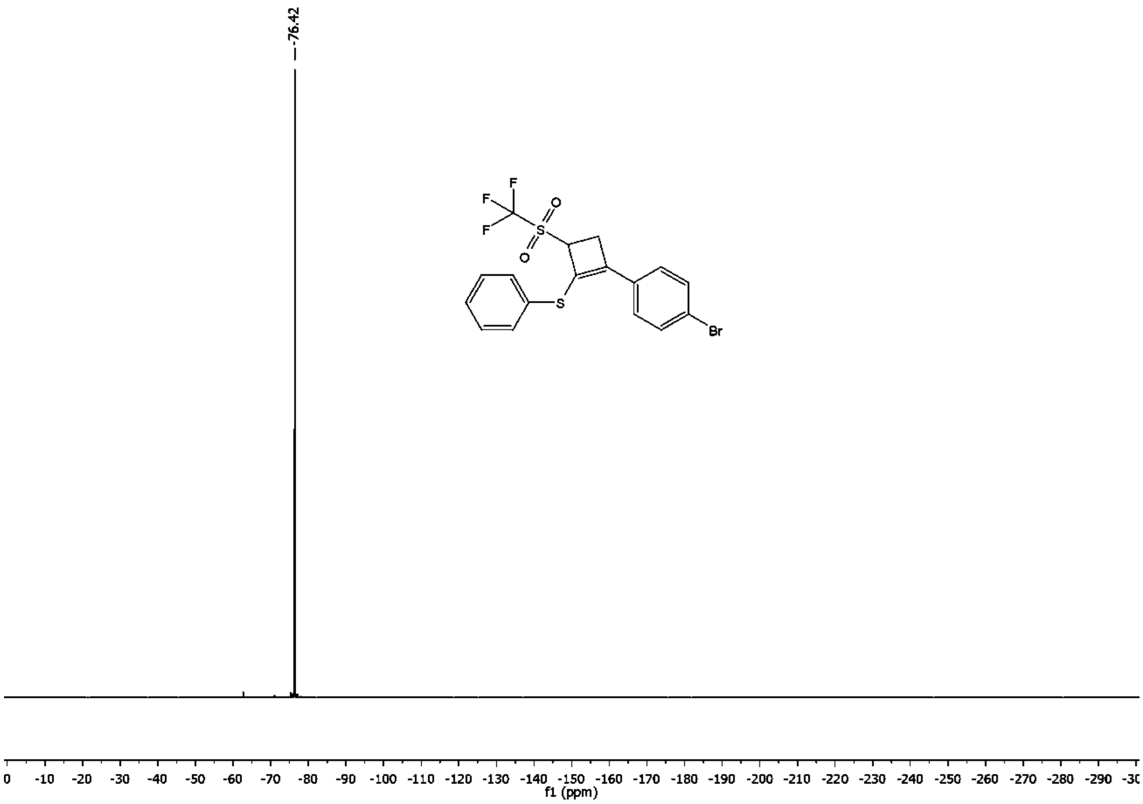

^{1}H NMR compound **4d** (Acetone, 700 MHz, 25 °C)

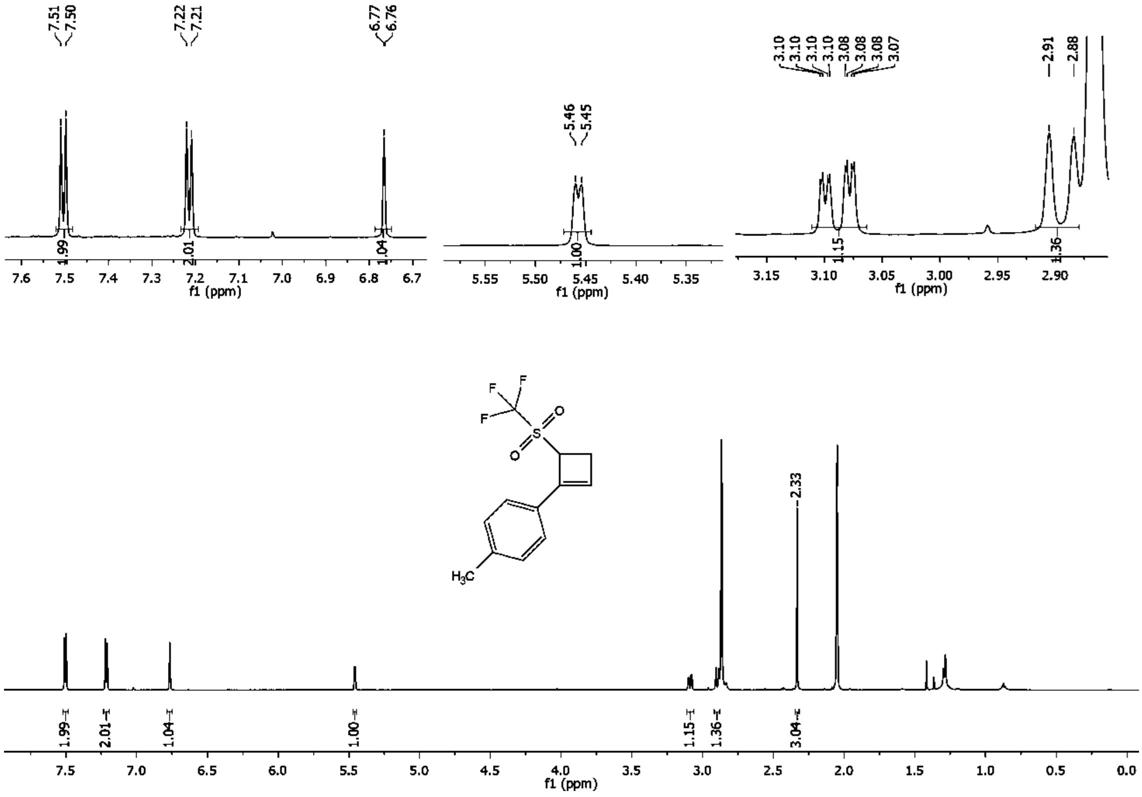

^{13}C NMR compound **4d** (acetone-d$_6$, 176 MHz, 25 °C)

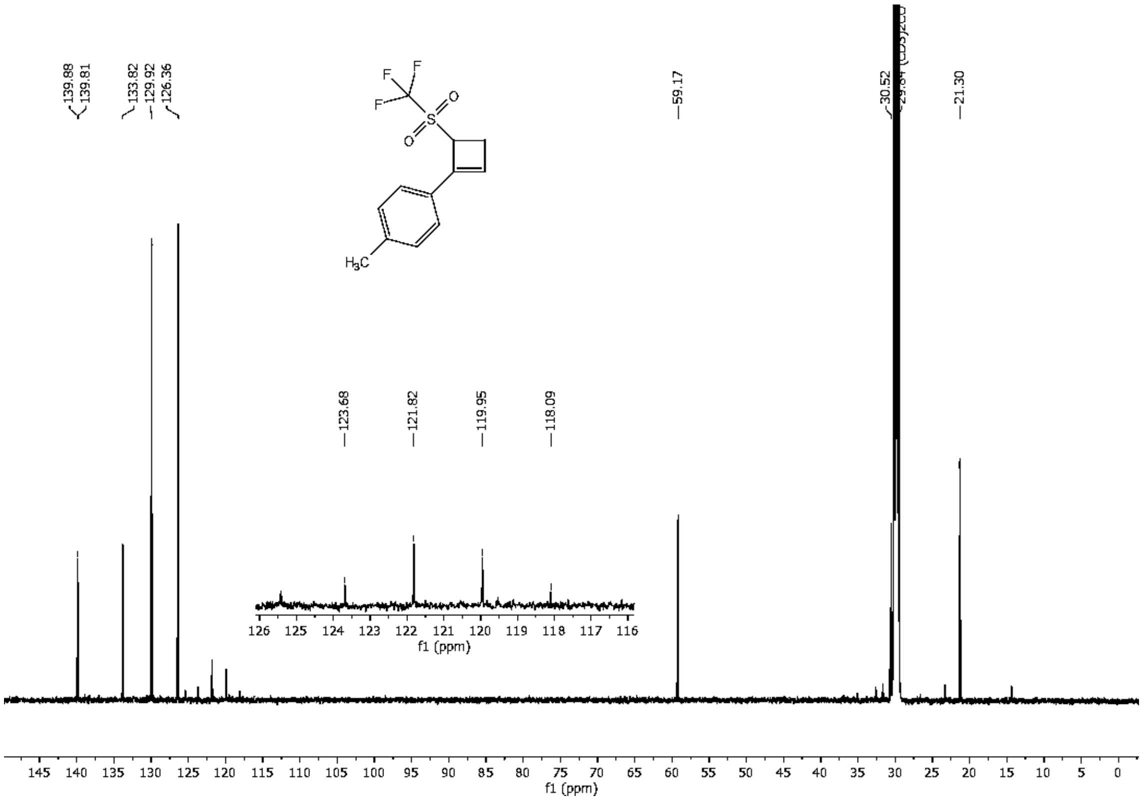

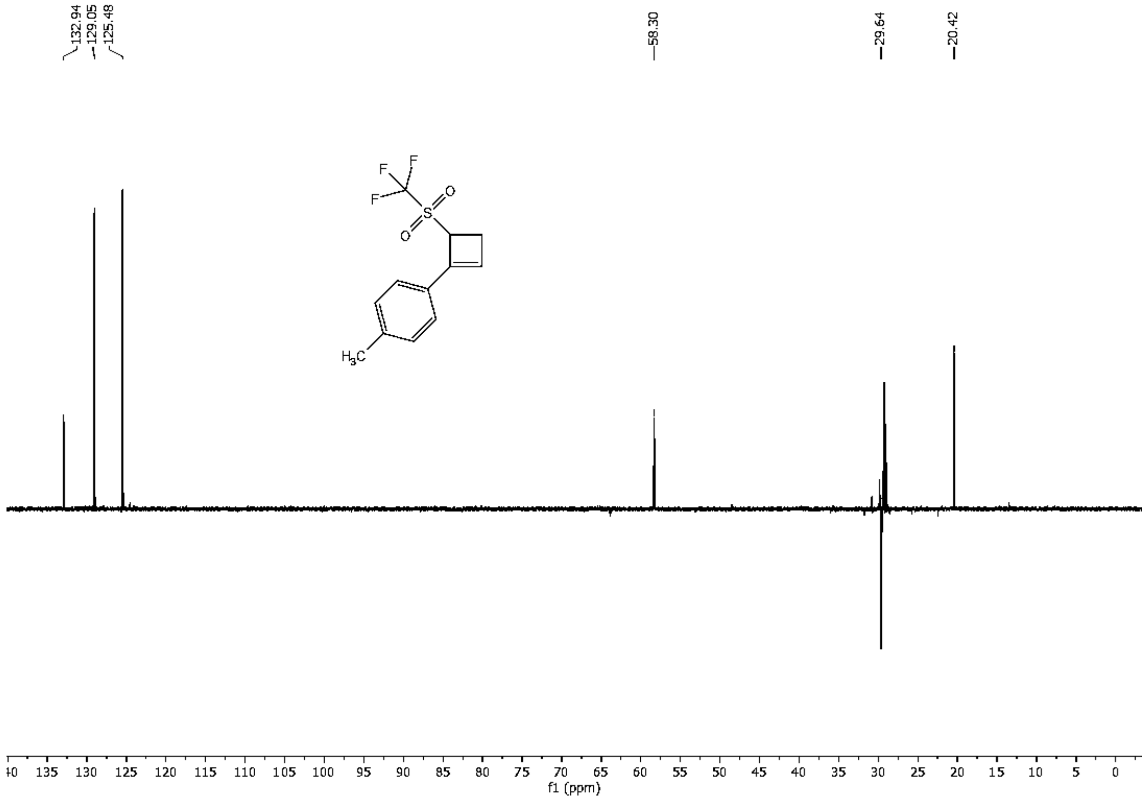
132.94
129.05
125.48
58.30
29.64
20.42
F
F
F
O
S
O
H3C
f1 (ppm)

^{19}F NMR compound **4d** (acetone-d_6, 282 MHz, 25 °C)

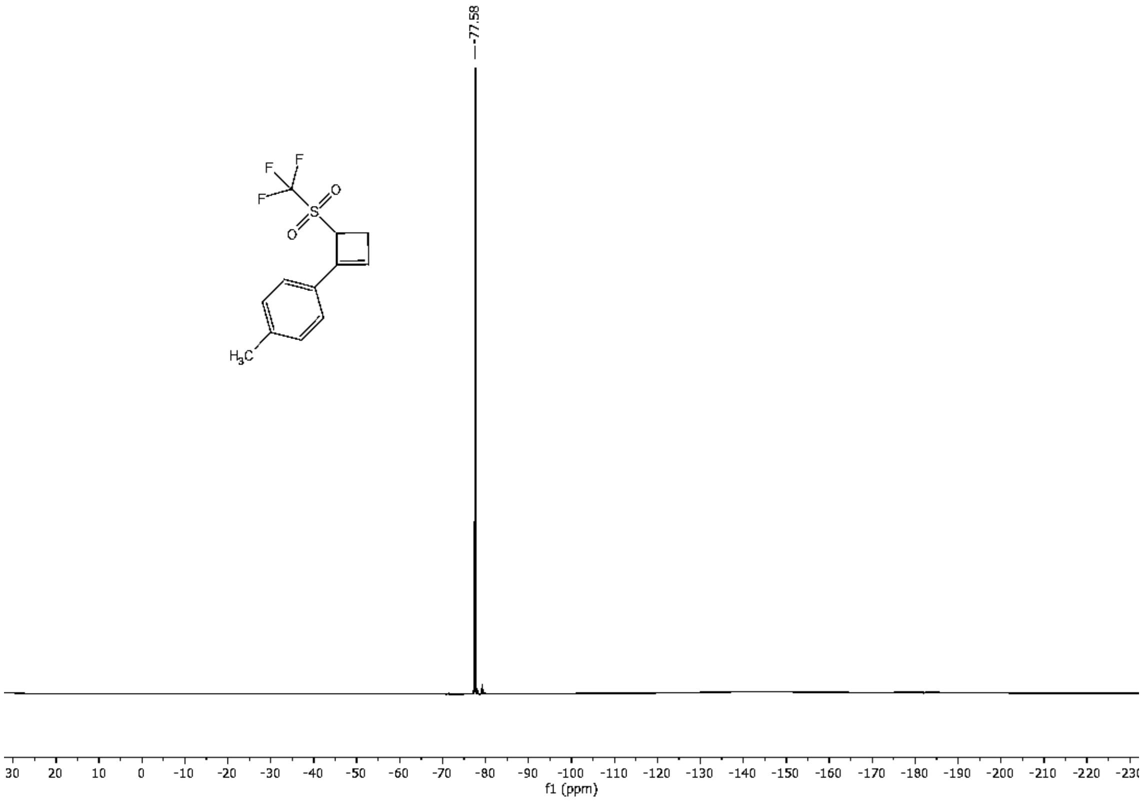

^{1}H NMR compound **4i** ($CDCl_3$, 300 MHz, 25 °C)

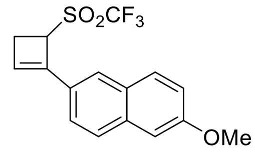

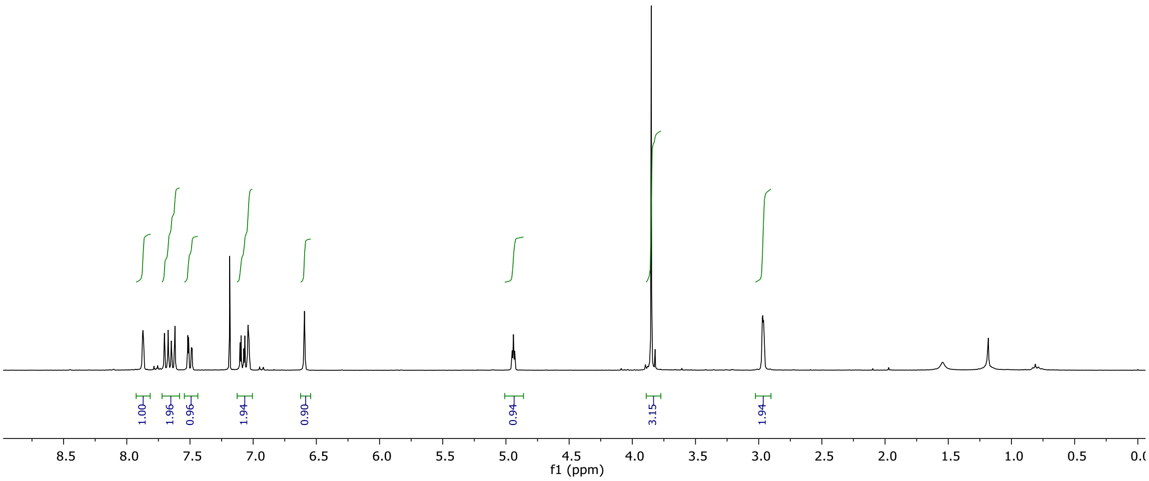

^{13}C NMR compound **4i** ($CDCl_3$, 75 MHz, 25 °C)

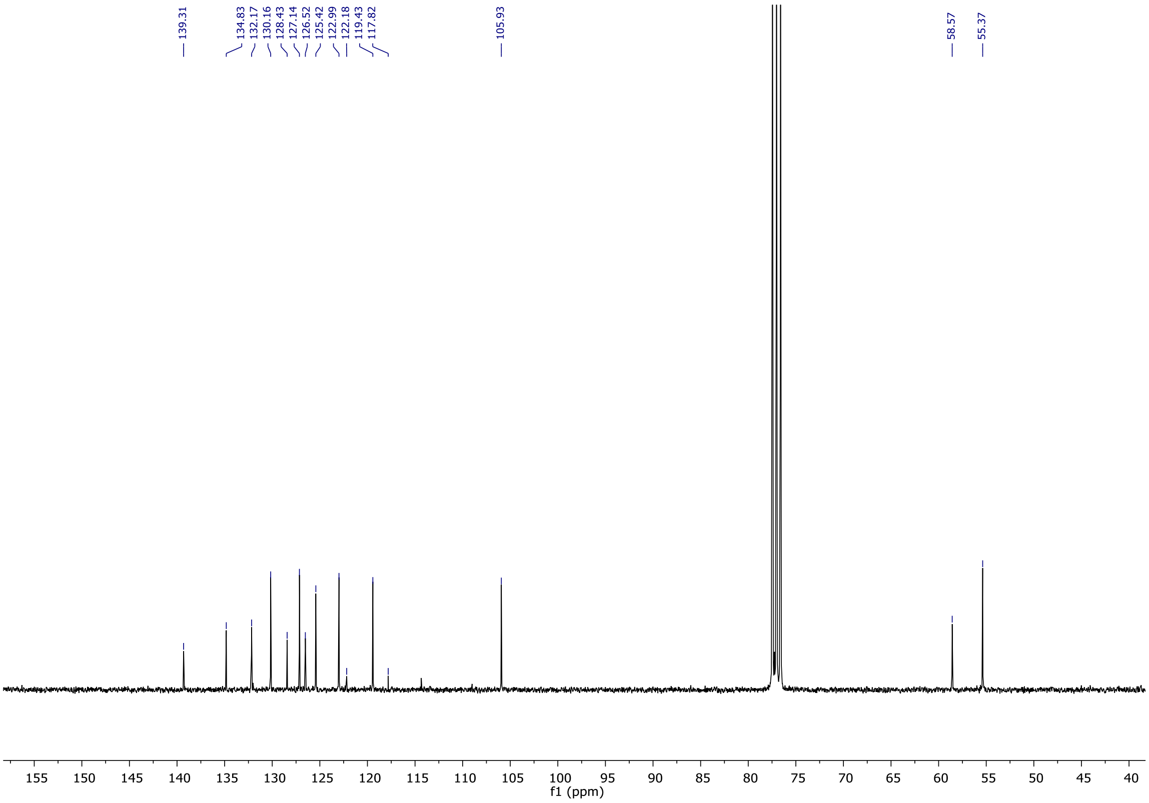

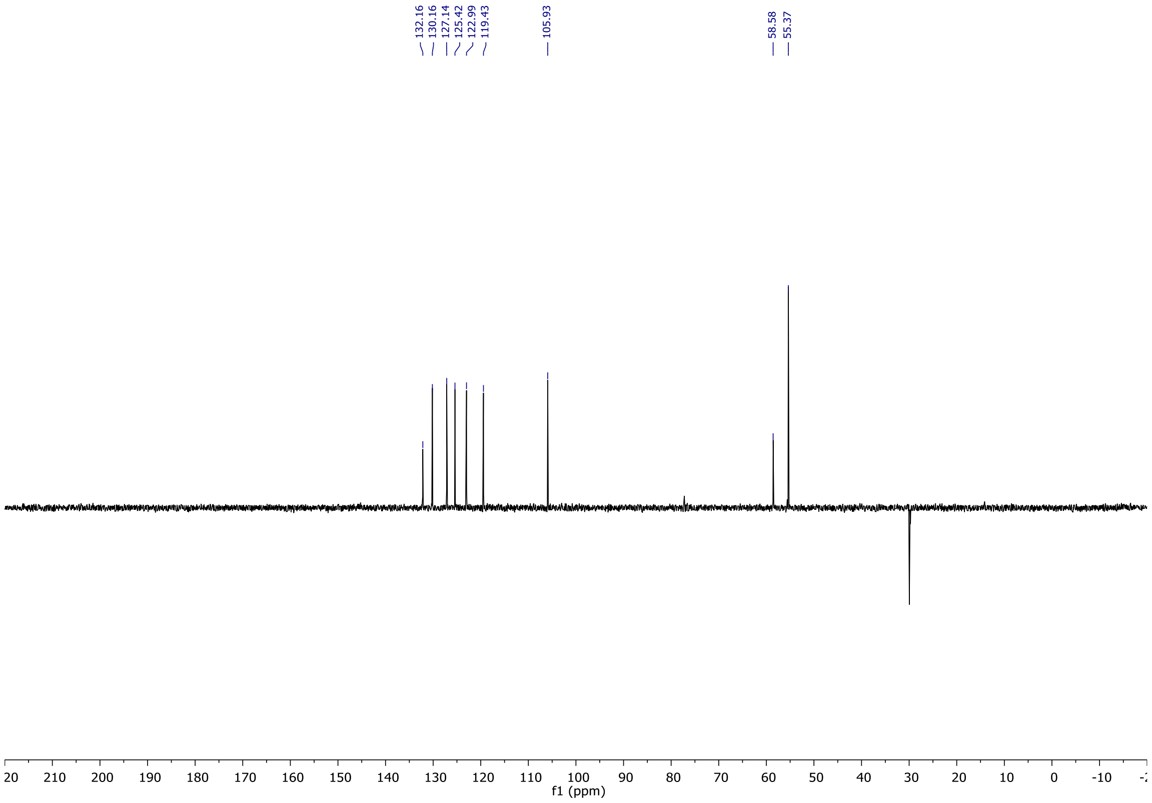
132.16
130.16
127.14
125.42
122.99
119.43
105.93
58.58
55.37
f1 (ppm)

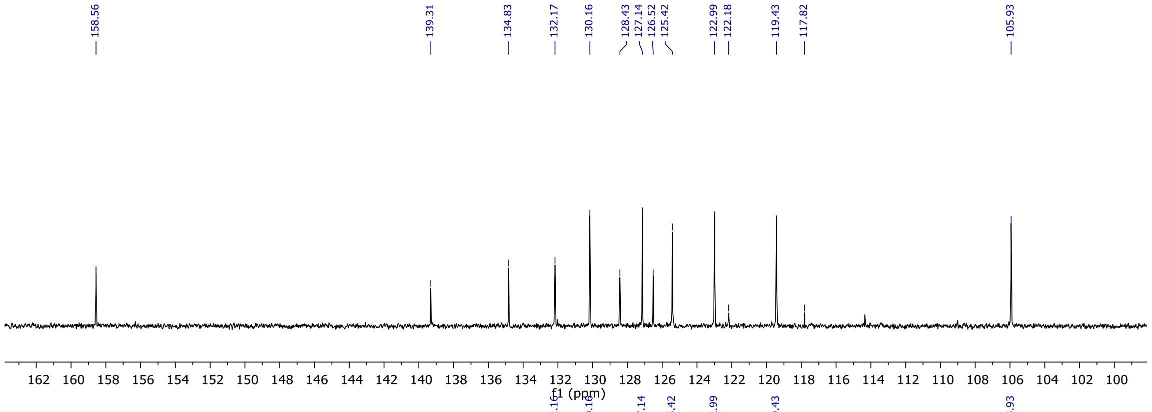
158.56
139.31
134.83
132.17
130.16
128.43
127.14
126.52
125.42
122.99
122.18
119.43
117.82
105.93
f1 (ppm)

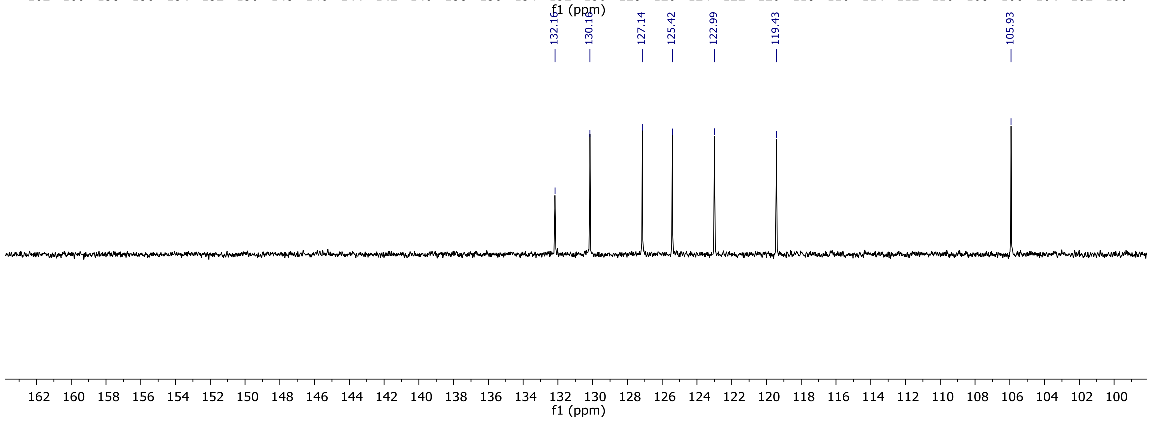
132.16
130.16
127.14
125.42
122.99
119.43
105.93
f1 (ppm)

^{19}F NMR compound **4i** ($CDCl_3$, 282 MHz, 25 ^{o}C)

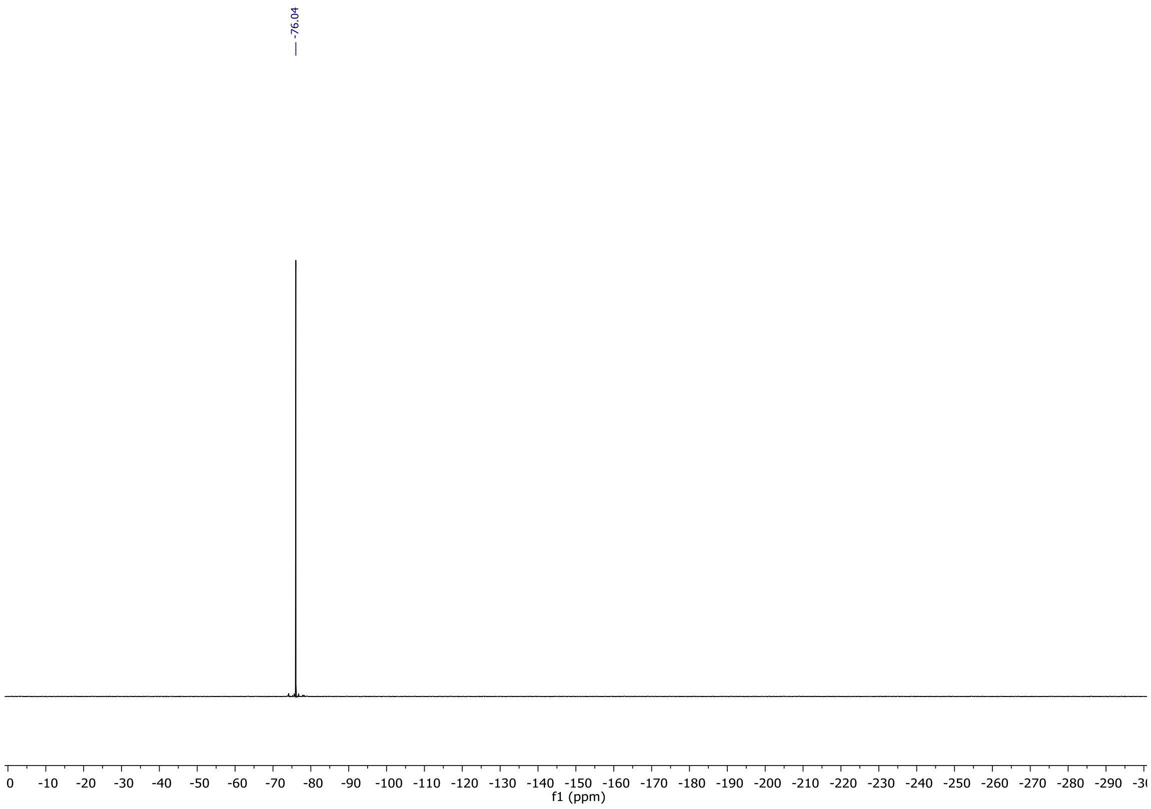

^{1}H NMR compound **5i** ($CDCl_3$, 300 MHz, 25 °C)

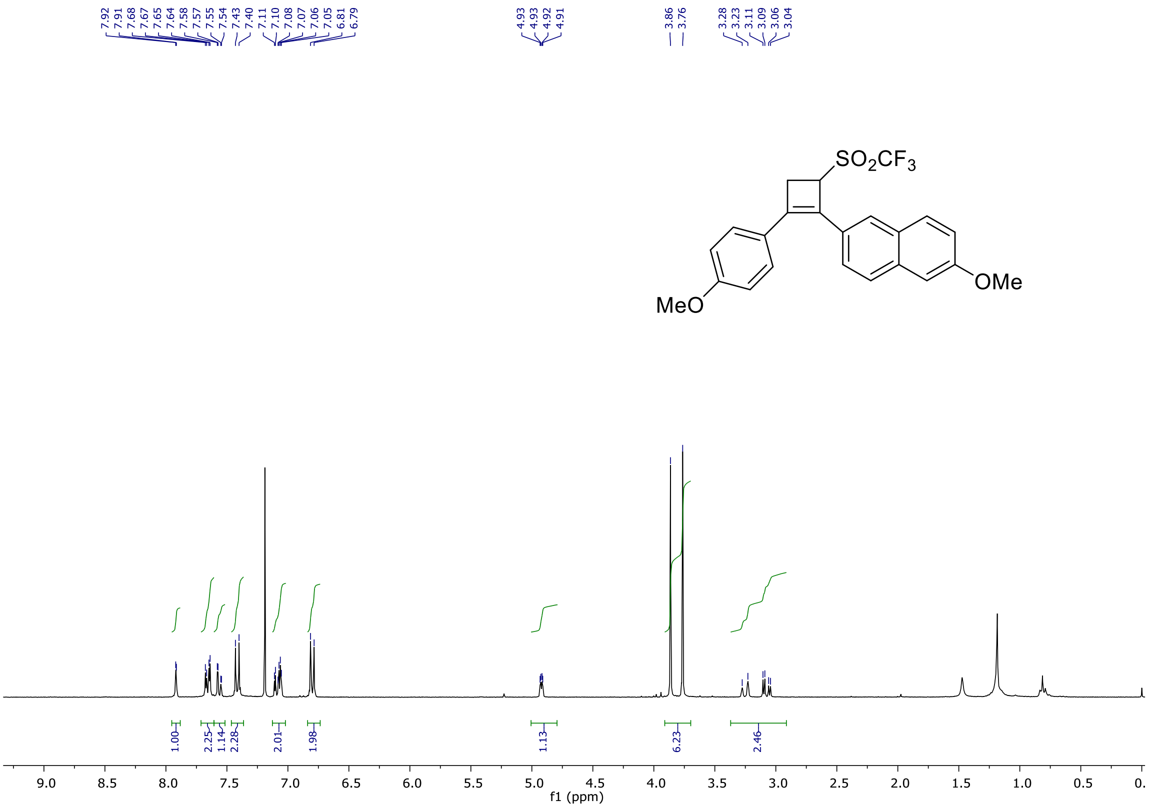

^{13}C NMR compound **5i** ($CDCl_3$, 75 MHz, 25 °C)

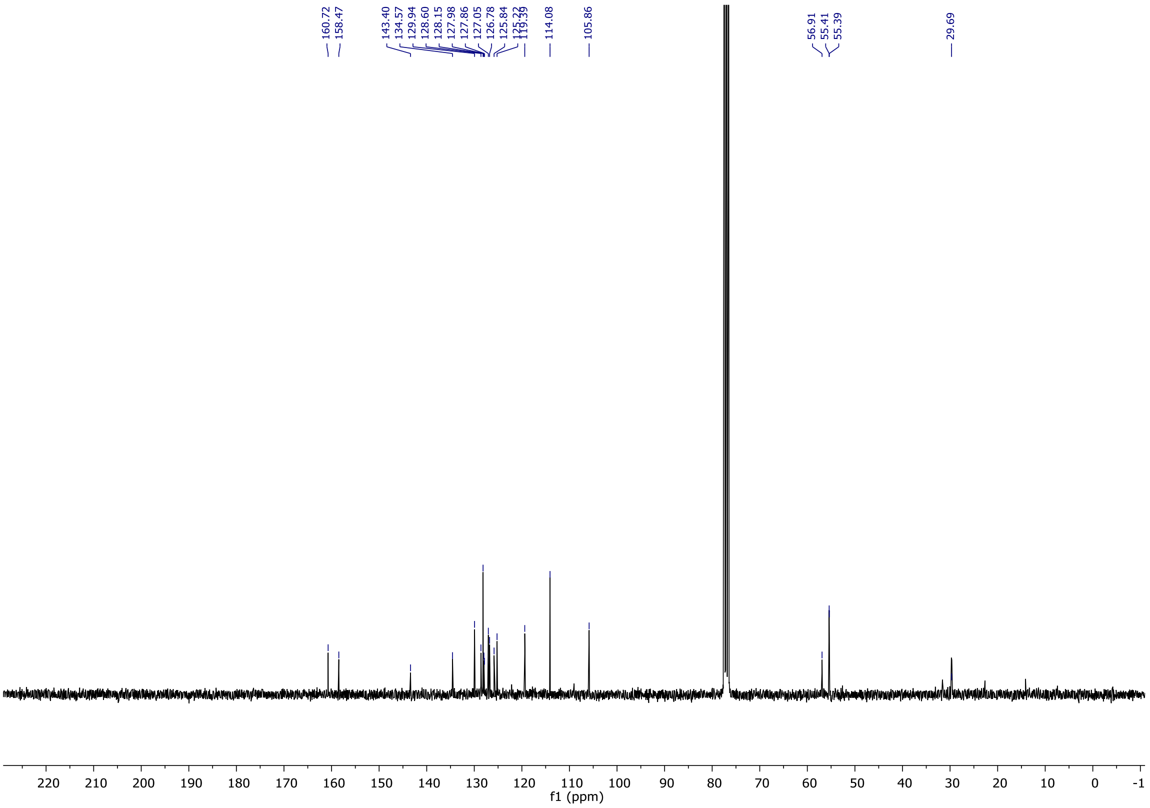

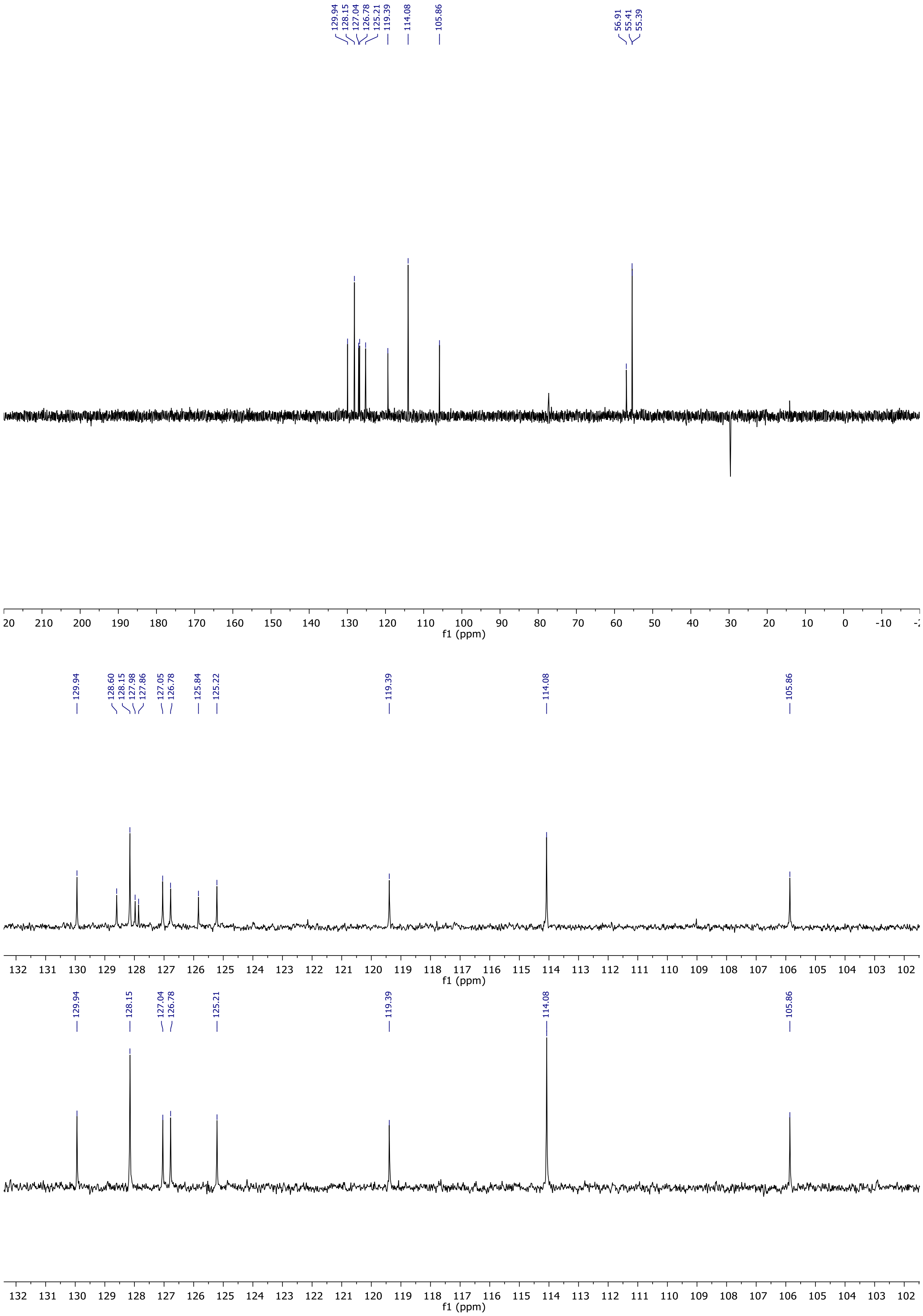
129.94
128.15
127.04
126.78
125.21
119.39
114.08
105.86
56.91
55.41
55.39
f1 (ppm)
129.94
128.60
128.15
127.98
127.86
127.05
126.78
125.84
125.22
119.39
114.08
105.86
f1 (ppm)
129.94
128.15
127.04
126.78
125.21
119.39
114.08
105.86
f1 (ppm)

^{19}F NMR compound **5i** ($CDCl_3$, 282 MHz, 25 ^{o}C)

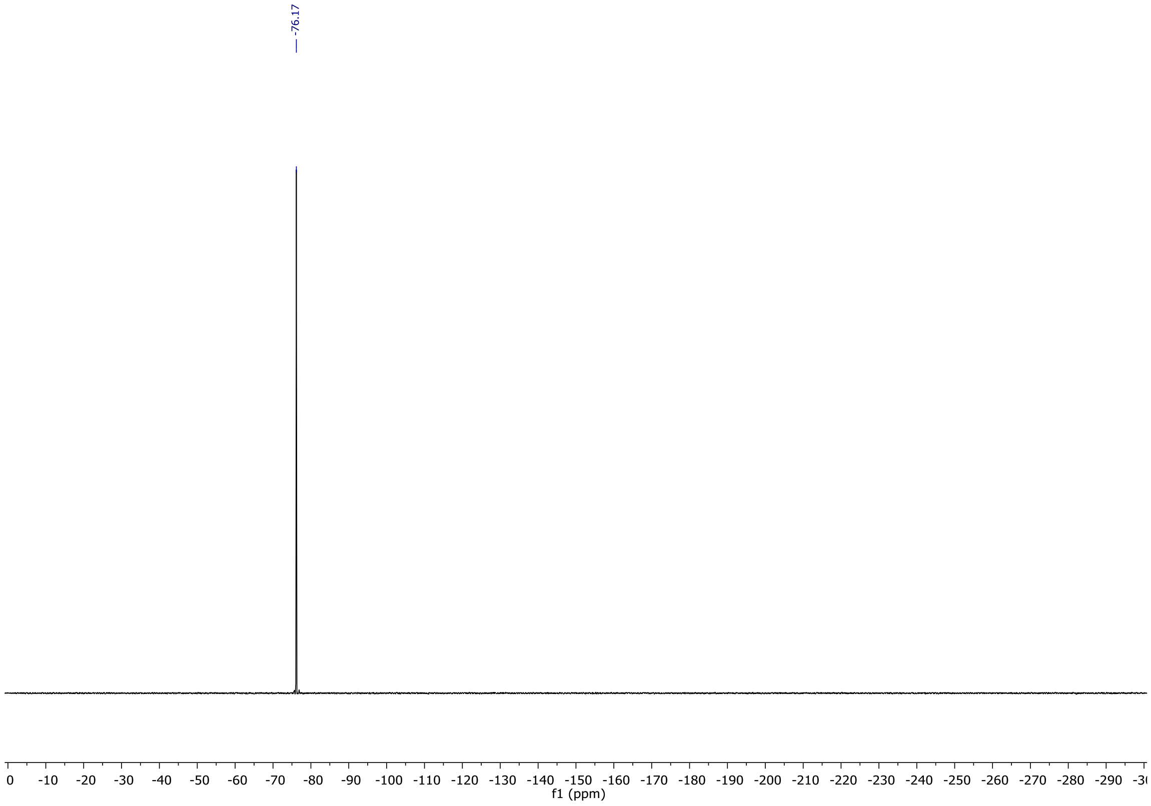

^{1}H NMR compound **5k** ($CDCl_3$, 700 MHz, 25 °C)

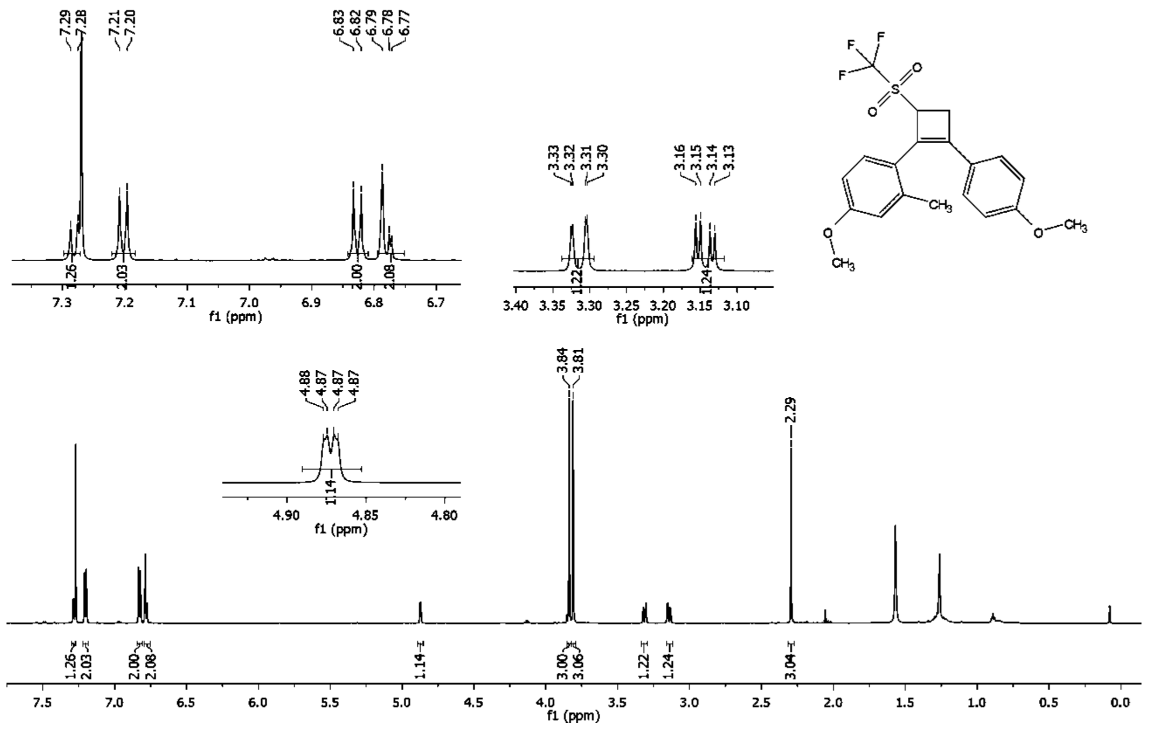

^{13}C NMR compound **5k** ($CDCl_3$, 176 MHz, 25 °C)

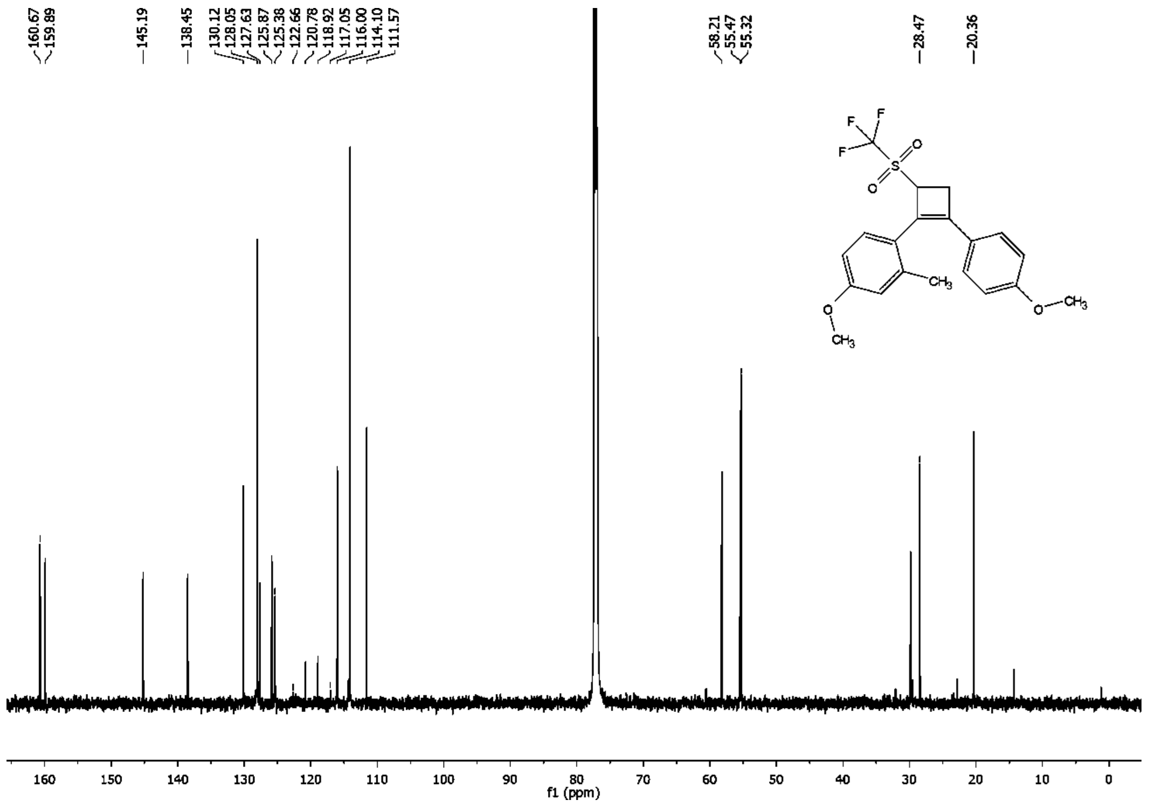

gHSQC NMR compound **5k** ($CDCl_3$, 700 MHz/176 MHz, 25 °C)

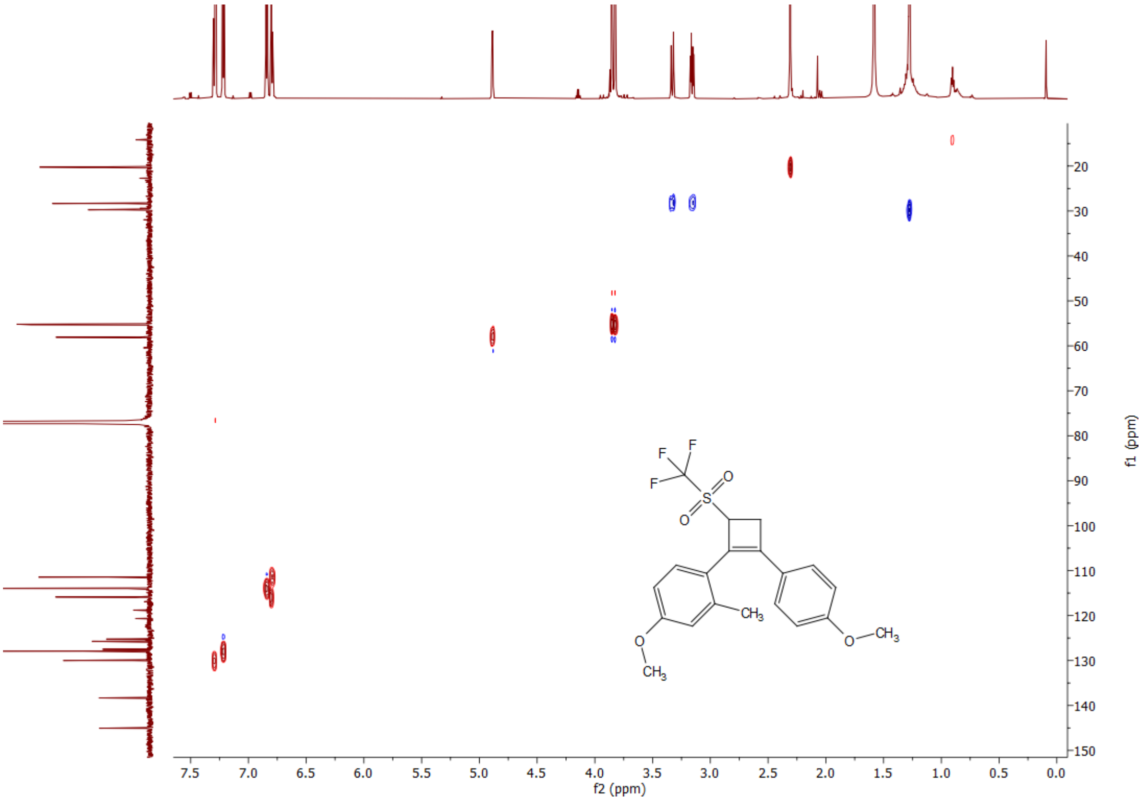

^{19}F NMR compound **5k** ($CDCl_3$, 282 MHz, 25 °C)

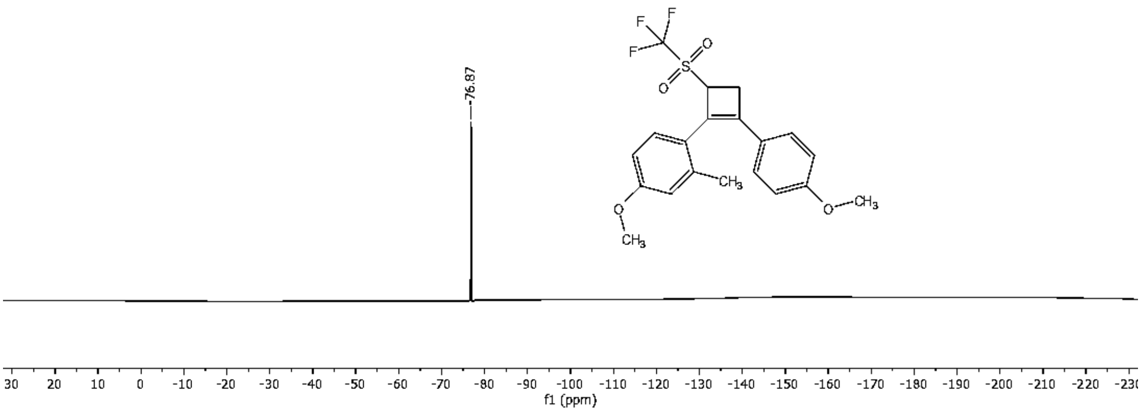

^{1}H NMR compound **5n** ($CDCl_3$, 300 MHz, 25 °C)

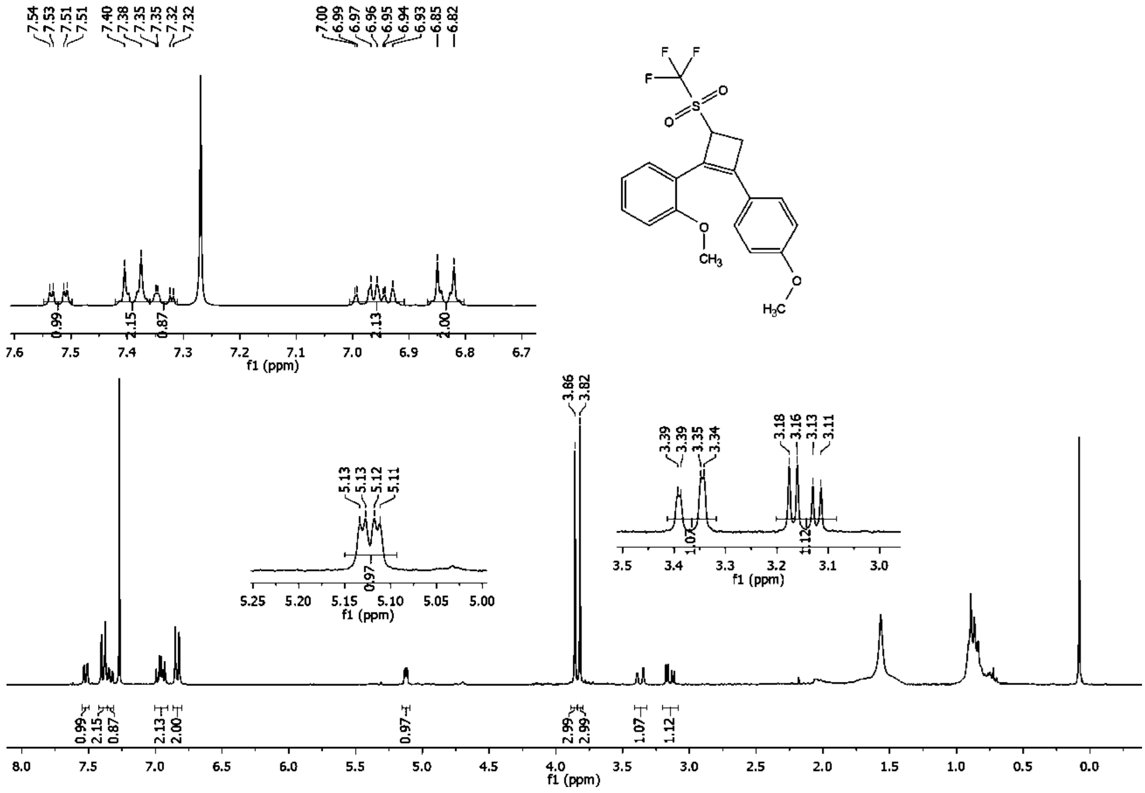

^{13}C NMR compound **5n** ($CDCl_3$, 126 MHz, 25 °C)

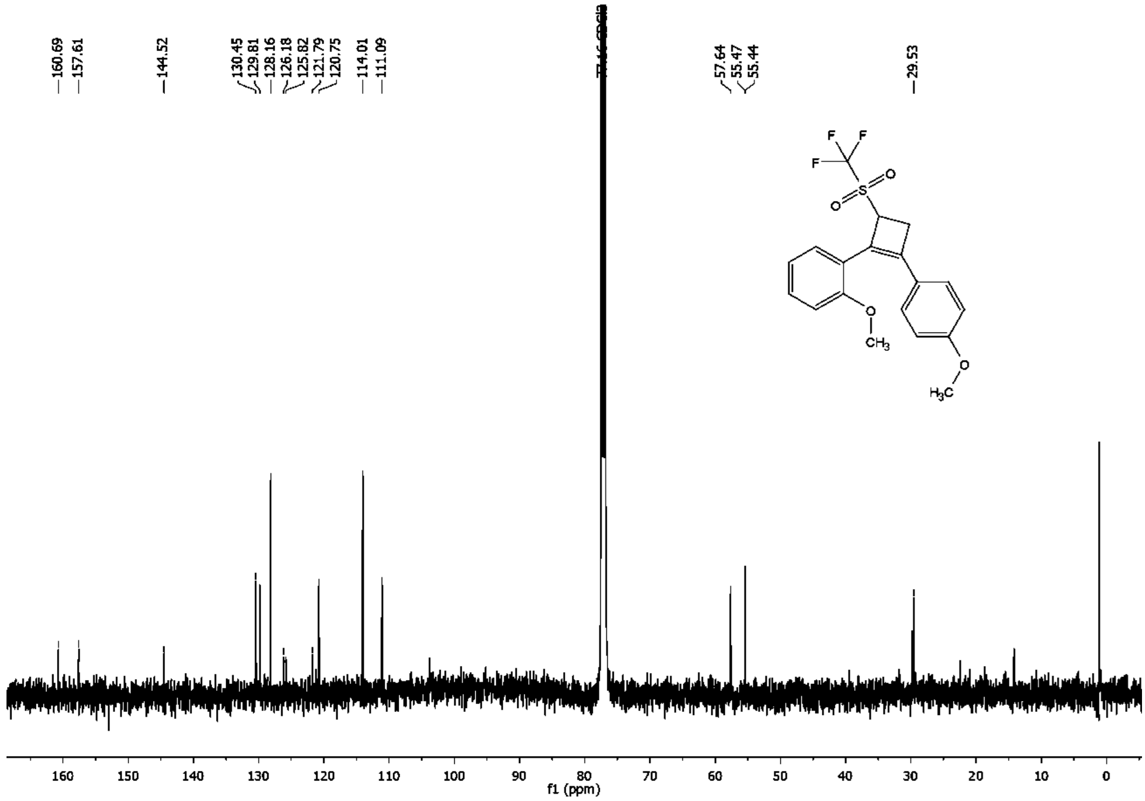

gHSQC of compound **5n** ($CDCl_3$, 500 MHz/126 MHz, 25 °C)

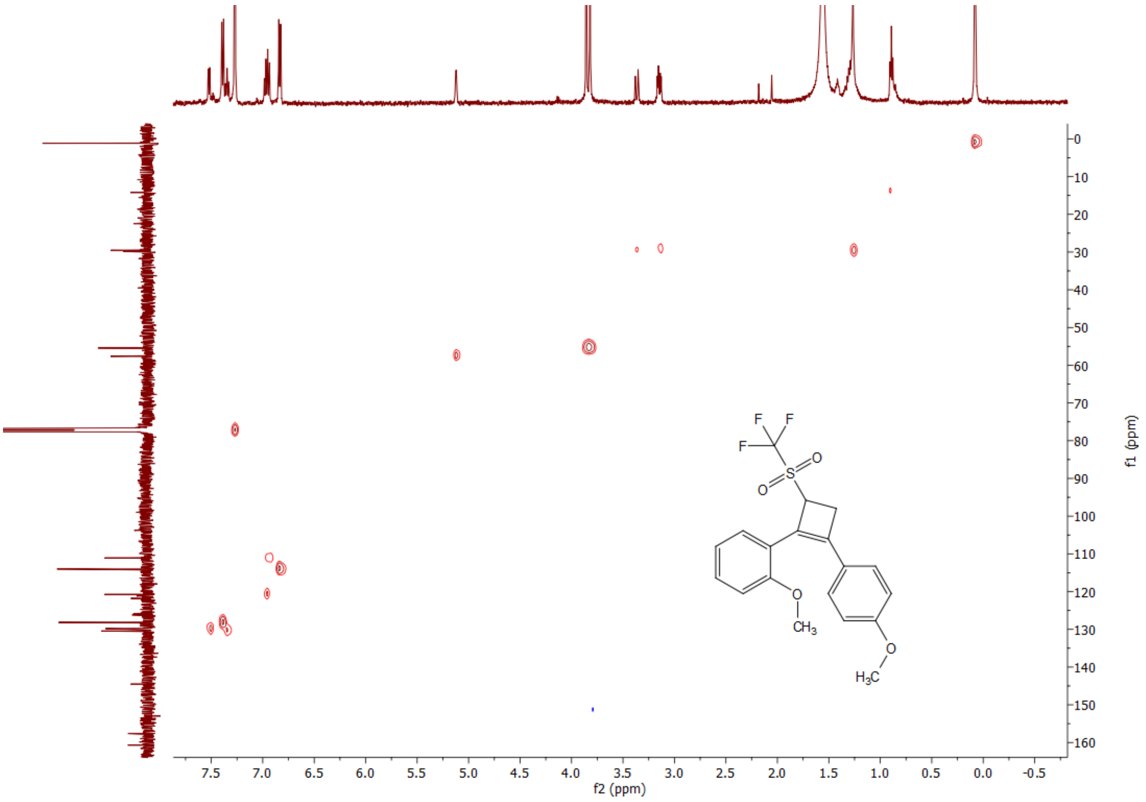

^{19}F NMR compound **5n** ($CDCl_3$, 470 MHz, 25 °C)

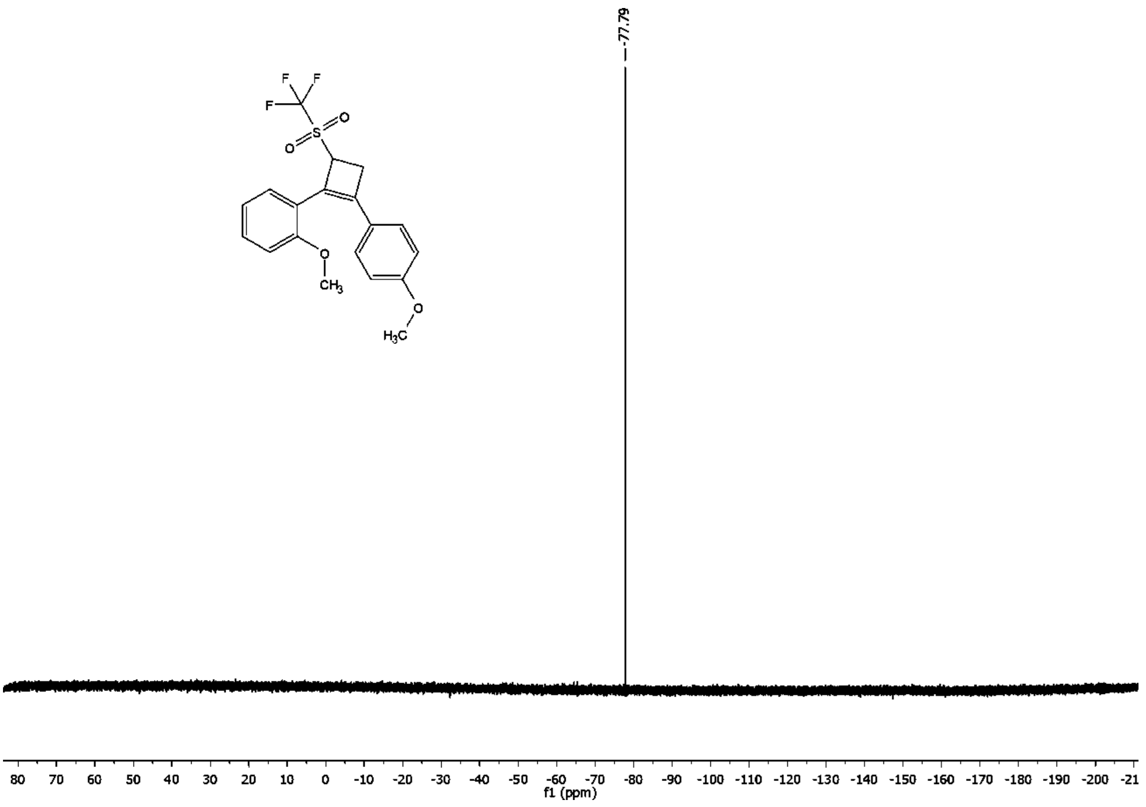

^{1}H NMR compound **6da** ($CDCl_3$, 700 MHz, 25 °C)

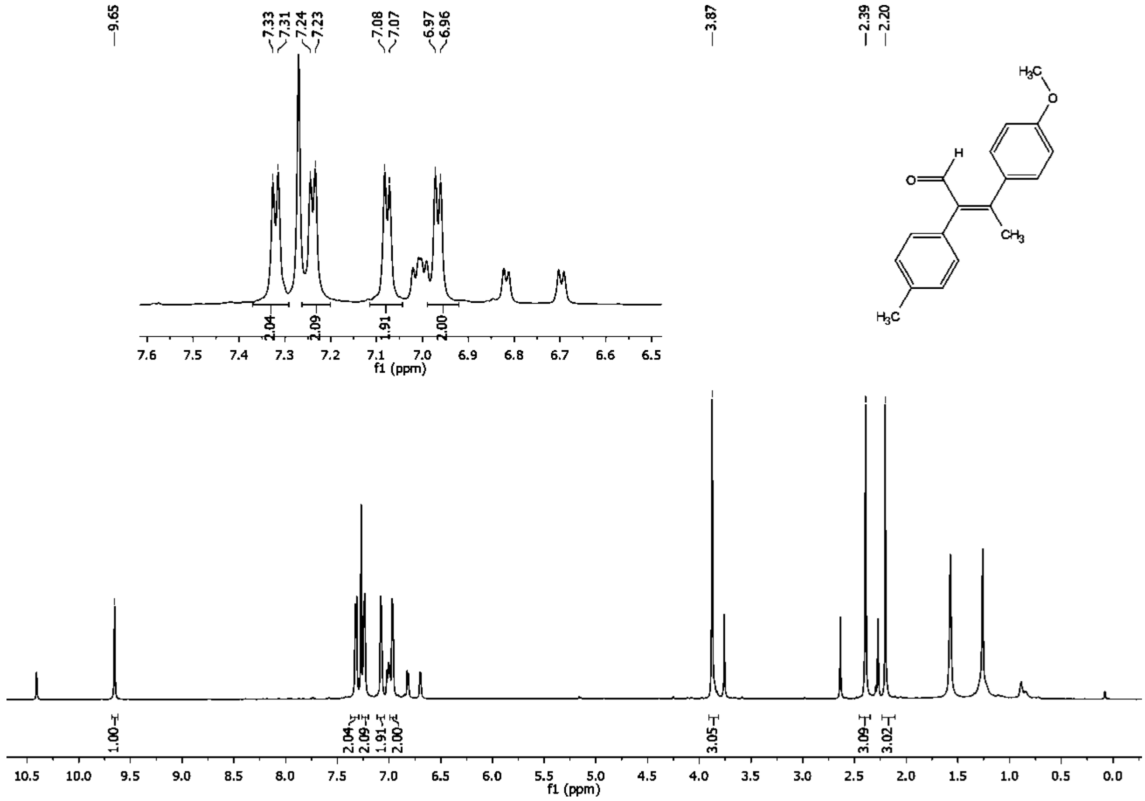

^{13}C NMR compound **6da** ($CDCl_3$, 176 MHz, 25 °C)

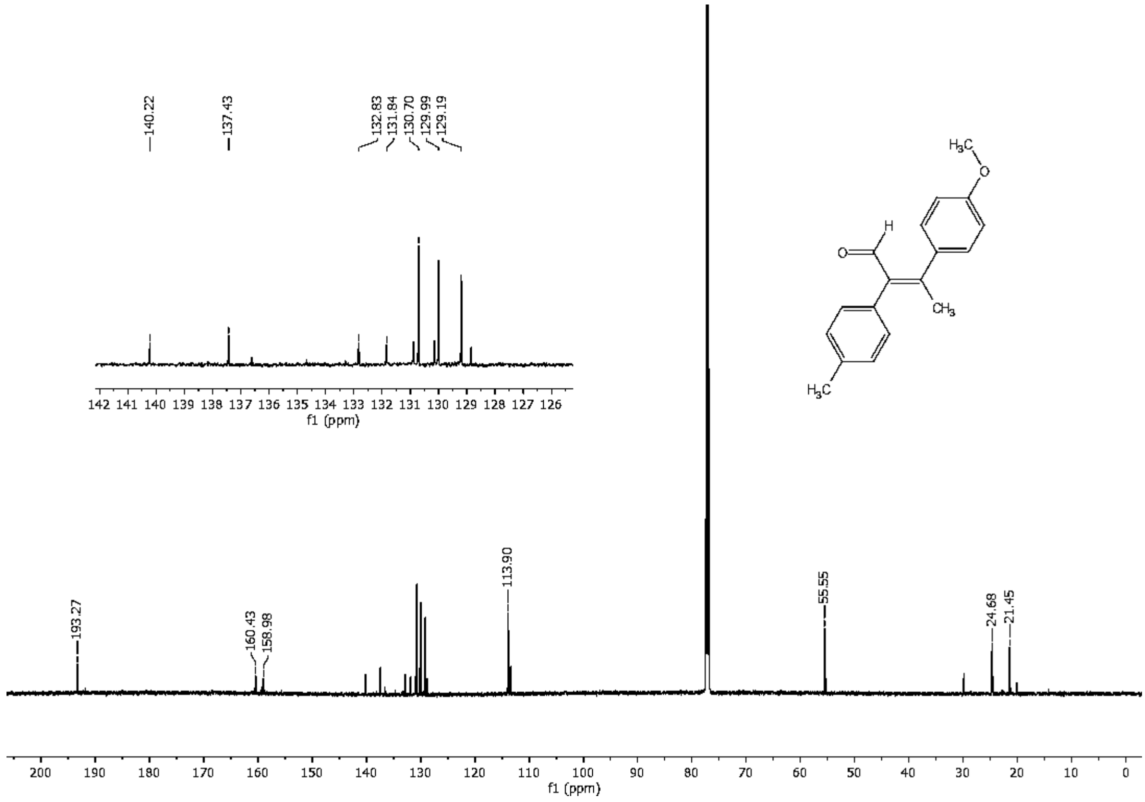

gCOSY NMR compound **6da** ($CDCl_3$, 700 MHz, 25 °C)

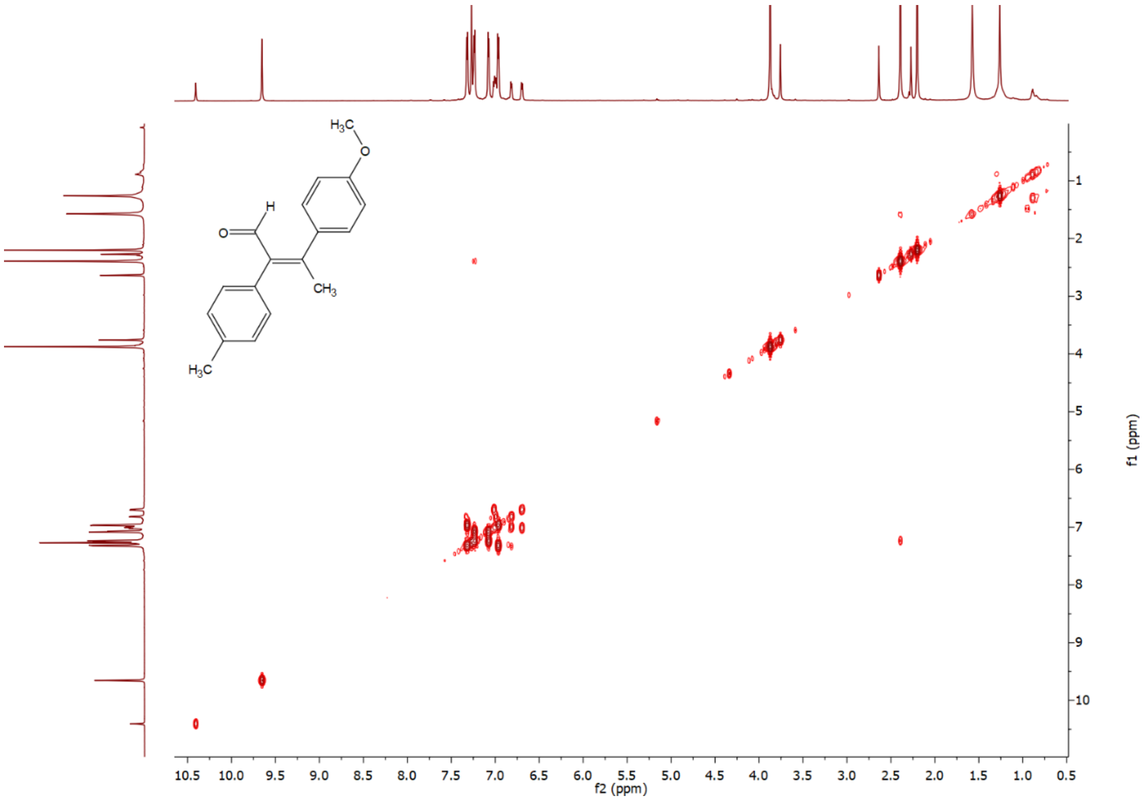

gHSQC-edited NMR compound **6da** ($CDCl_3$, 700 MHz/176 MHz, 25 °C)

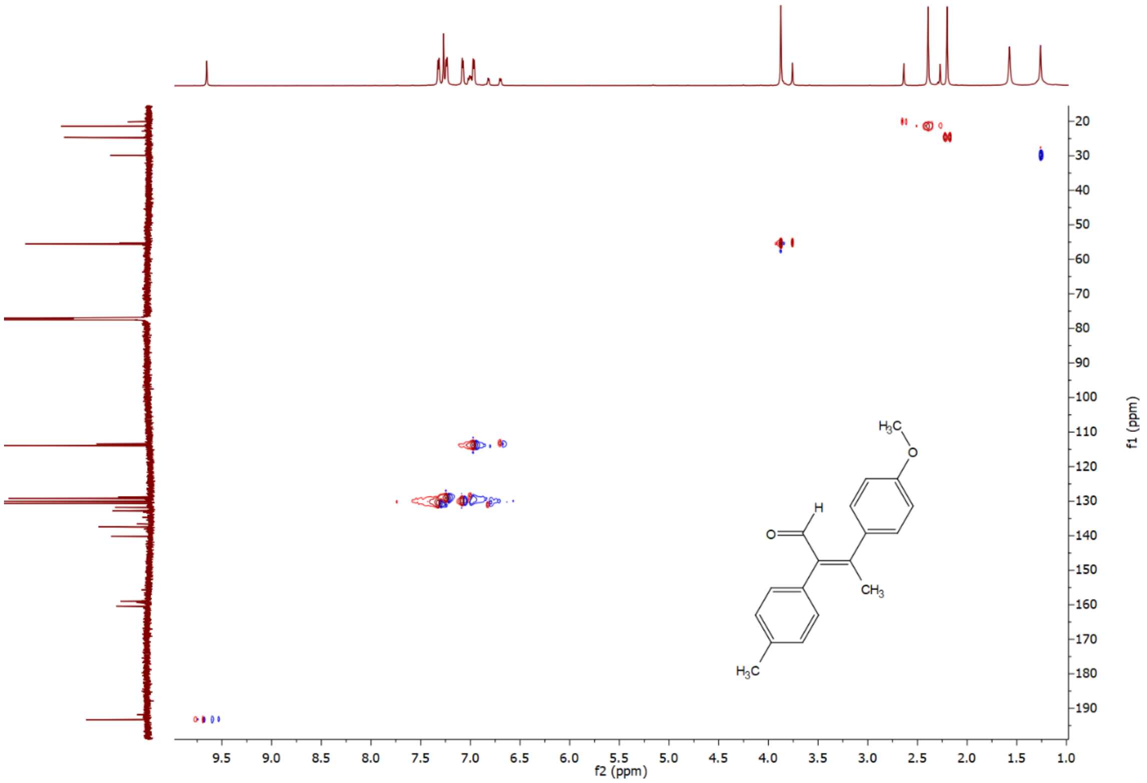

gHMBC NMR compound **6da** ($CDCl_3$, 700 MHz/176 MHz, 25 °C)

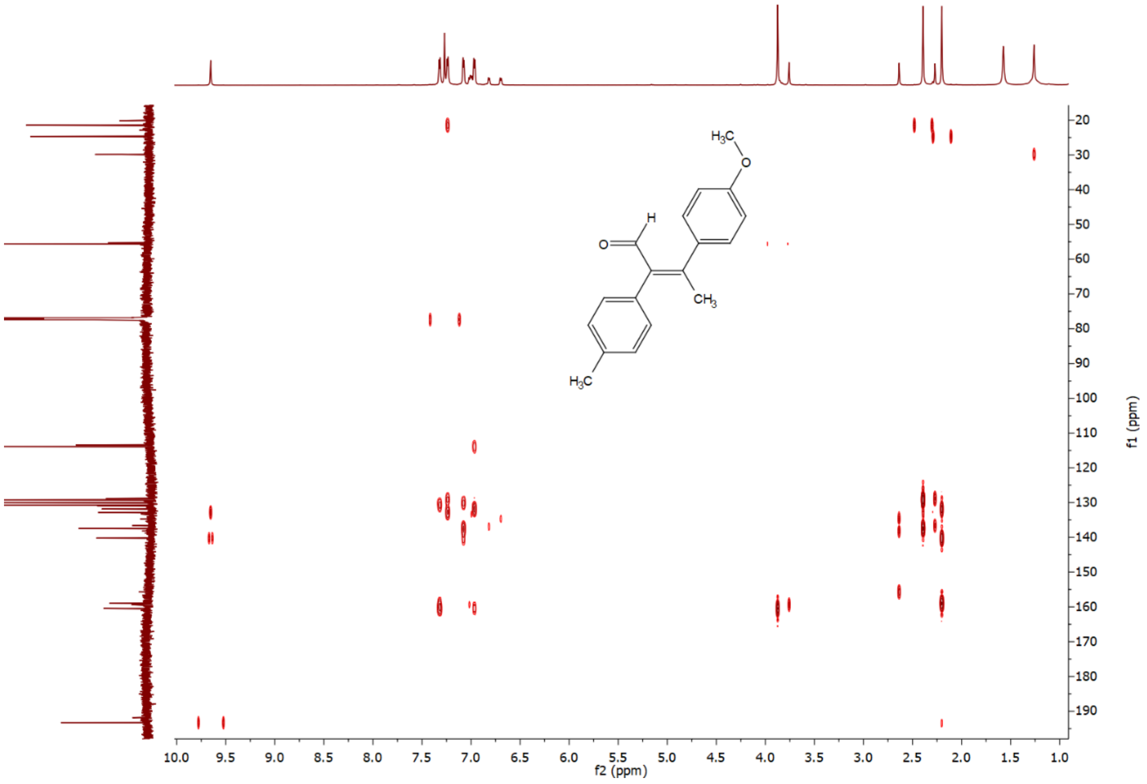

NOE NMR experiments of 6da and 6db

Irradiation of the methyl group at the alkene moiety showed effect on both Hc and Hb protons, in accordance with the Z configuration for compound **6da**.

Irradiation of the aldehyde proton only showed interaction with Hc protons, also supporting Z configuration.

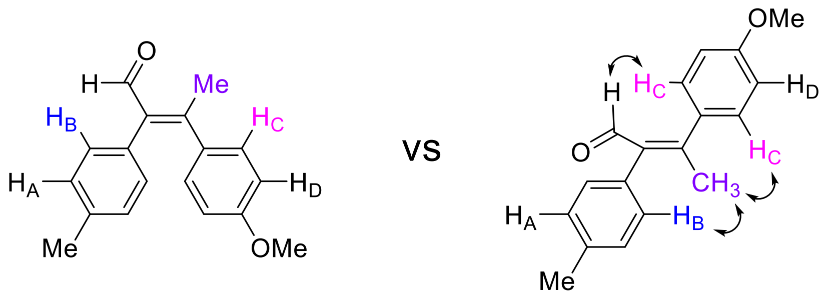

E - isomer *Z* - isomer

NOE NMR – aldehyde irradiation of compound **6da** ($CDCl_3$, 700 MHz, 25 ^oC)

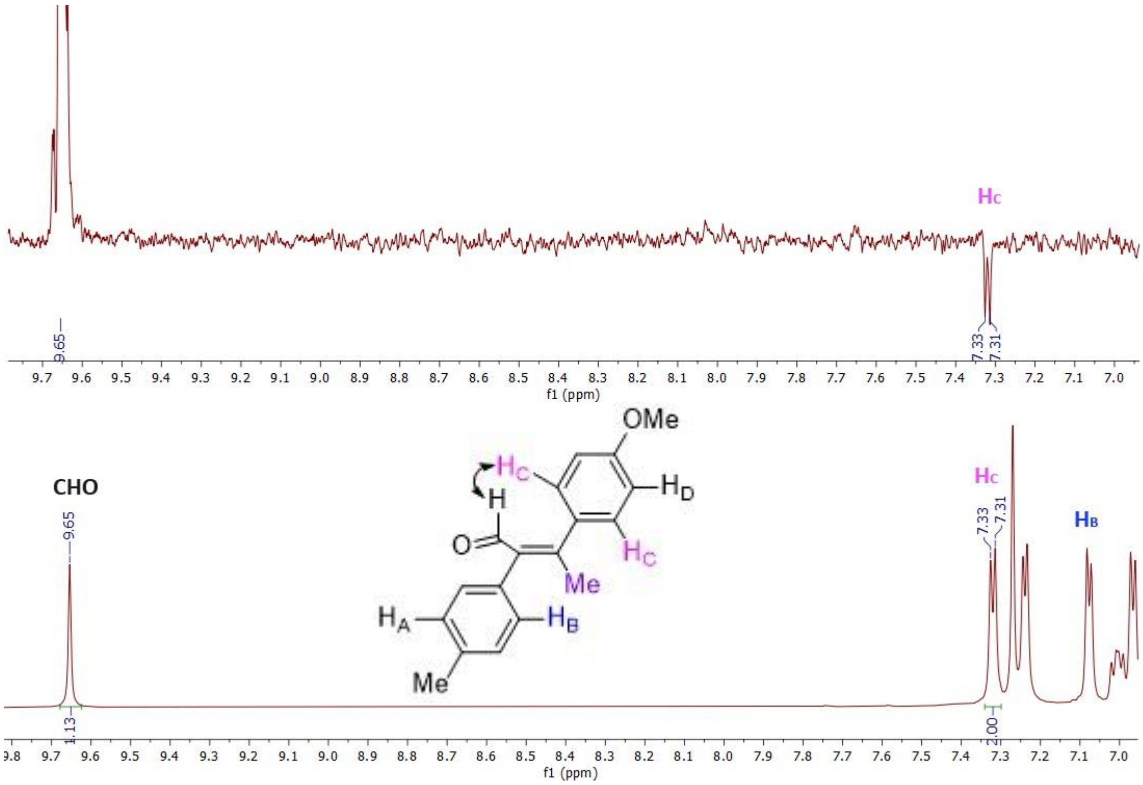

NOE NMR – methyl irradiation of compound **6da** ($CDCl_3$, 700 MHz, 25 ^oC)

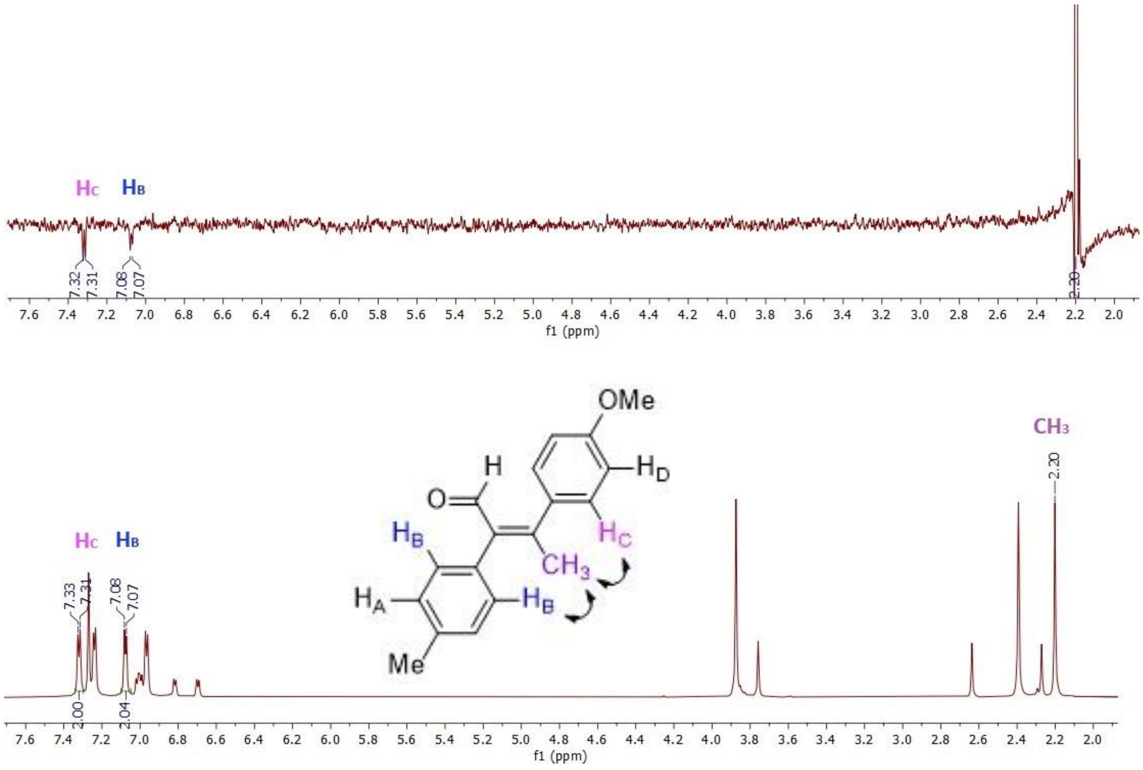

^{1}H NMR compound **6db** ($CDCl_3$, 500 MHz, 25 °C)

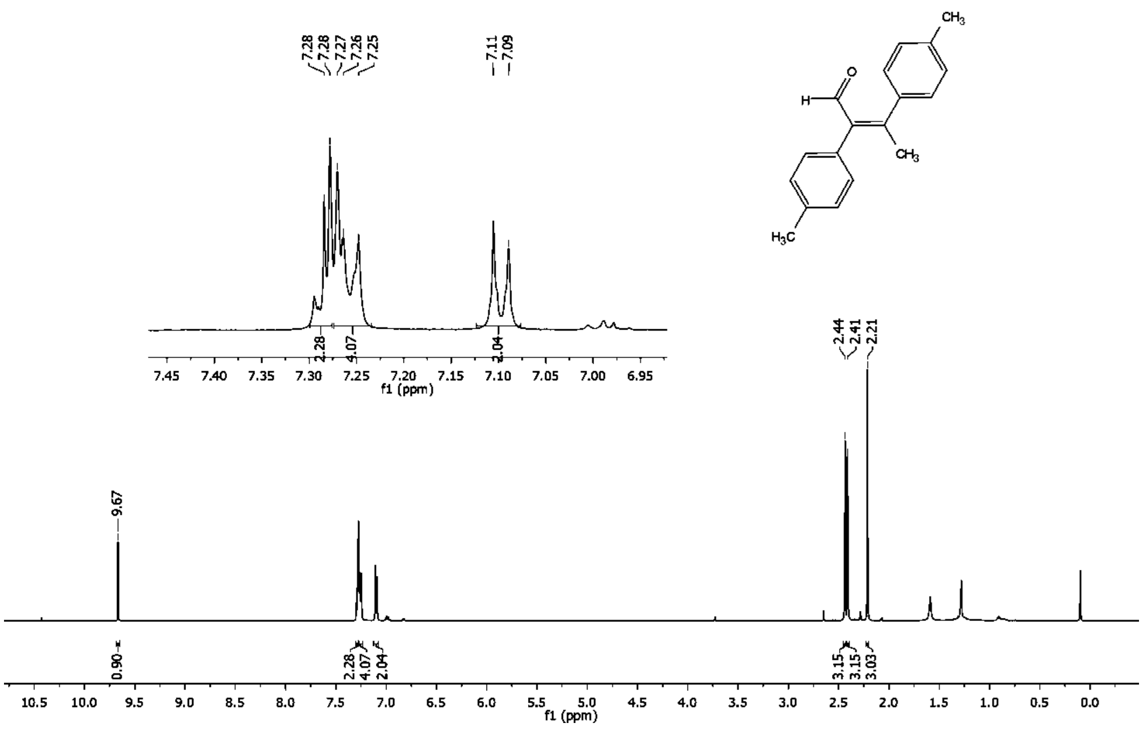

^{13}C NMR compound **6db** ($CDCl_3$, 126 MHz, 25 °C)

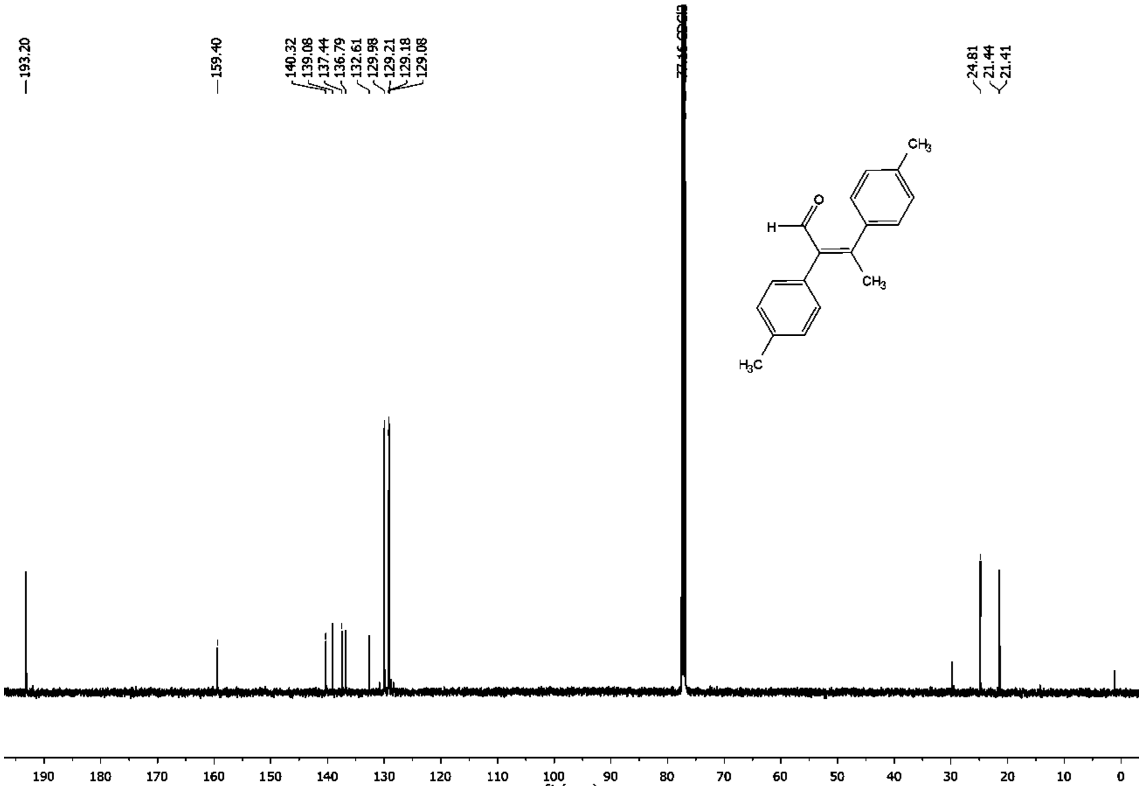

gHSQC NMR compound **6db** ($CDCl_3$, 500 MHz/126 MHz, 25 °C)

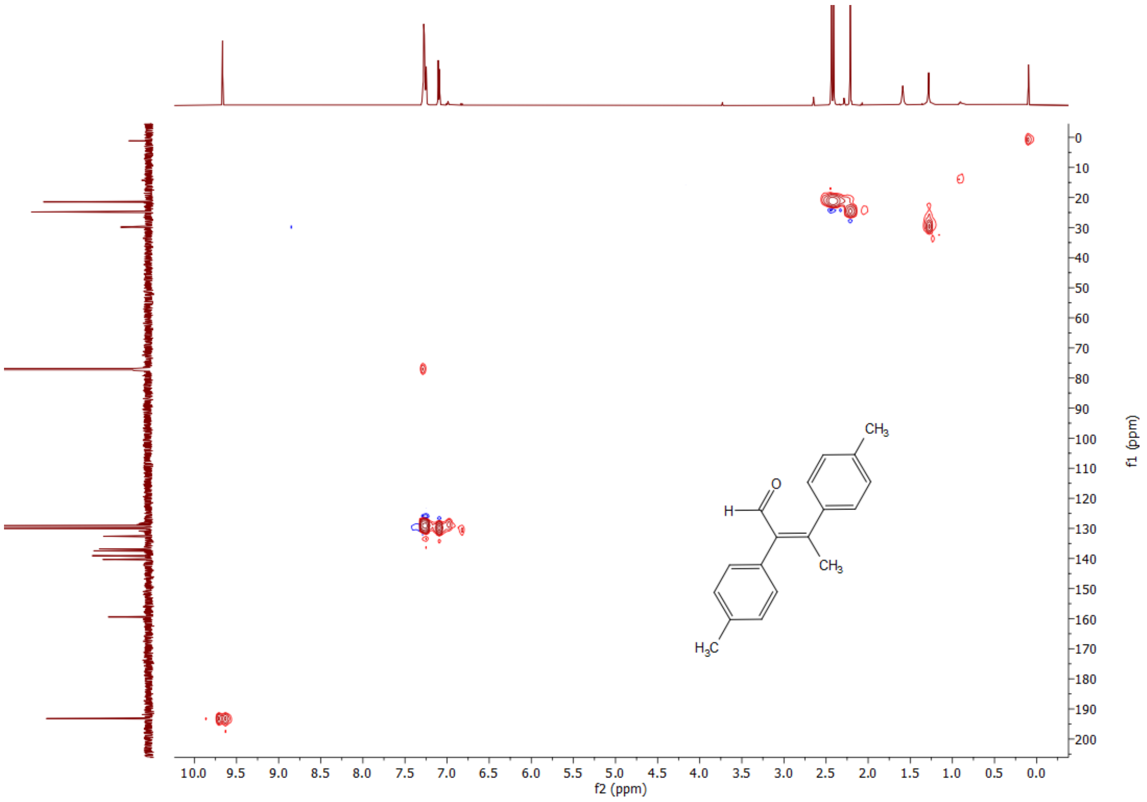

^{1}H NMR compound **7a** ($CDCl_3$, 300 MHz, 25 °C)

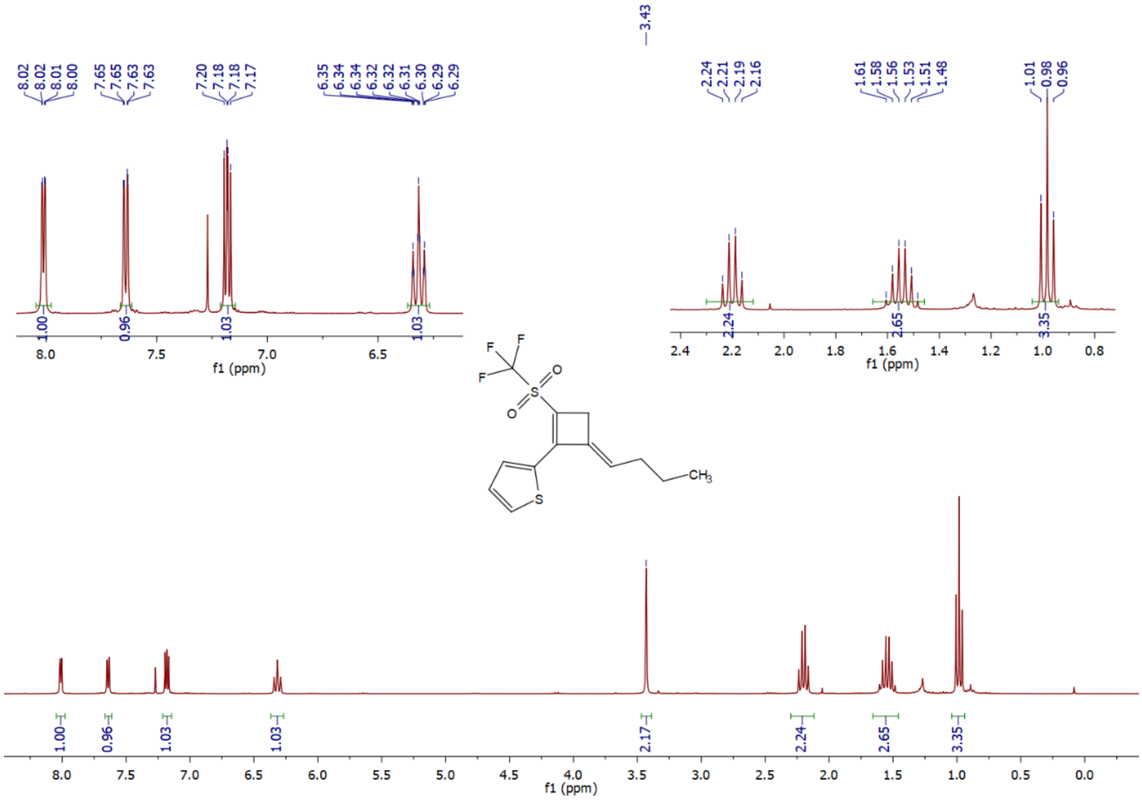

^{13}C NMR compound **7a** ($CDCl_3$, 176 MHz, 25 °C)

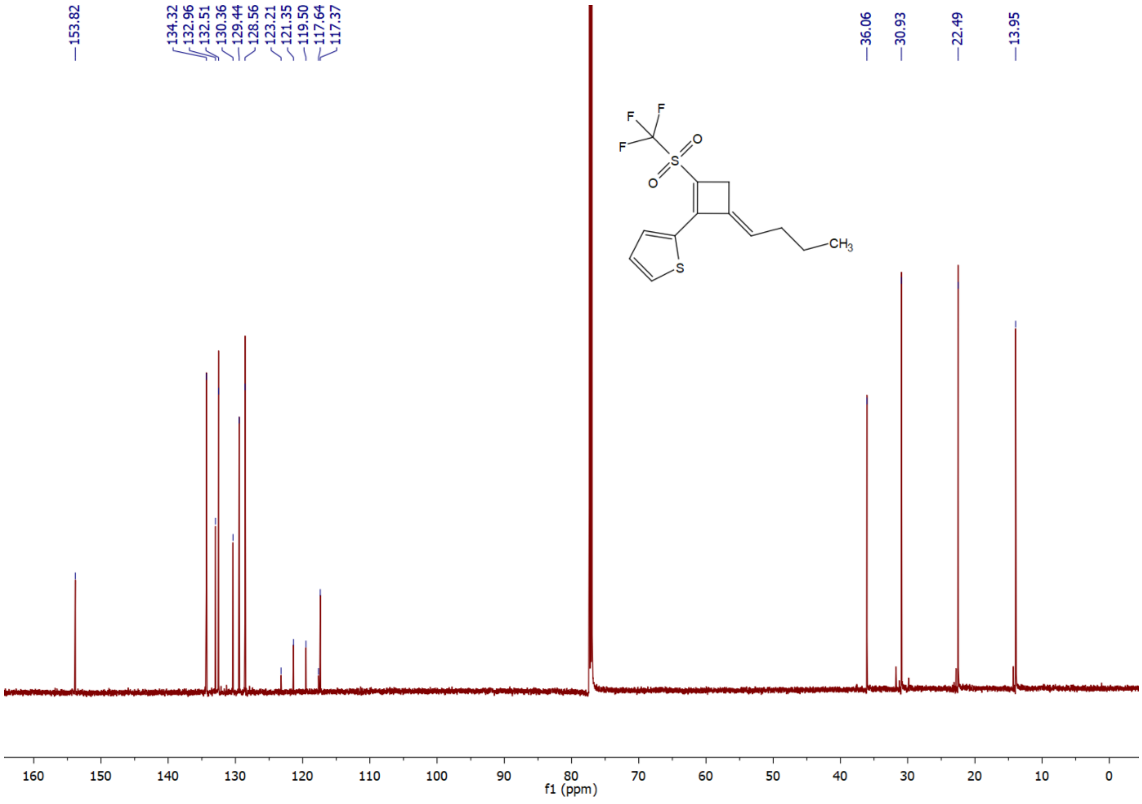

^{19}F NMR compound **7a** ($CDCl_3$, 282 MHz, 25 °C)

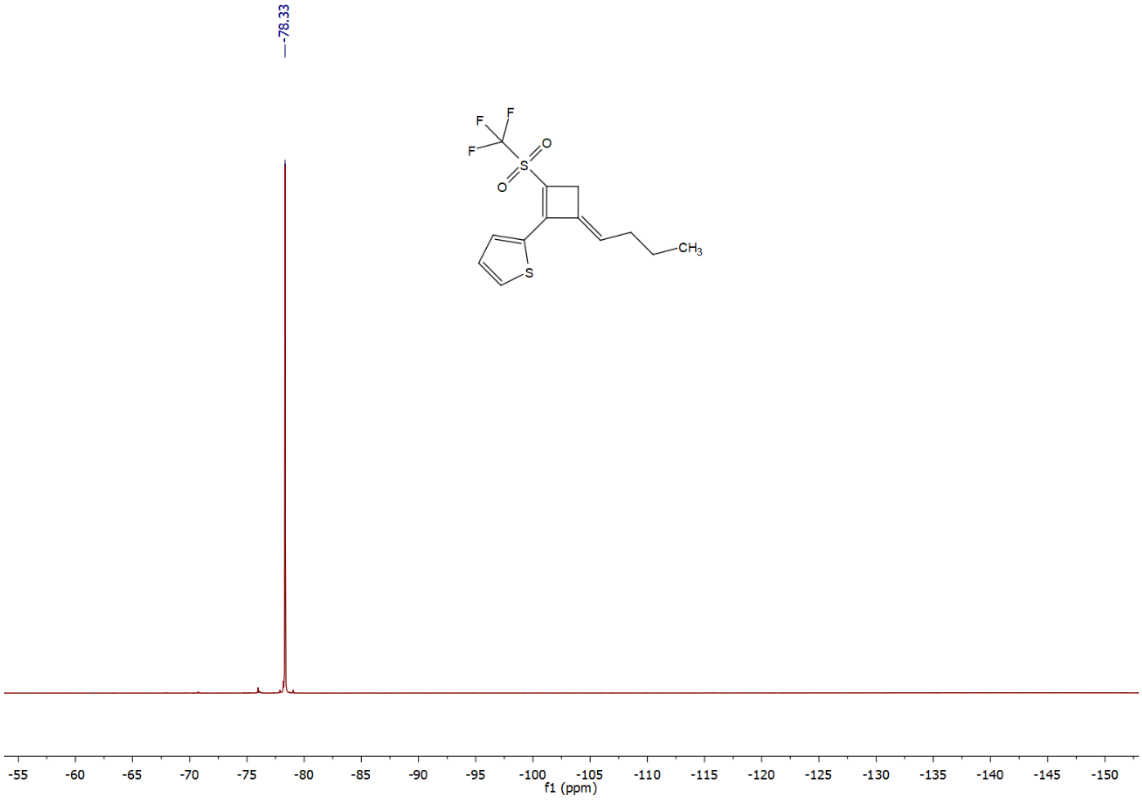

COSY NMR compound **7a** ($CDCl_3$, 700 MHz, 25 °C)

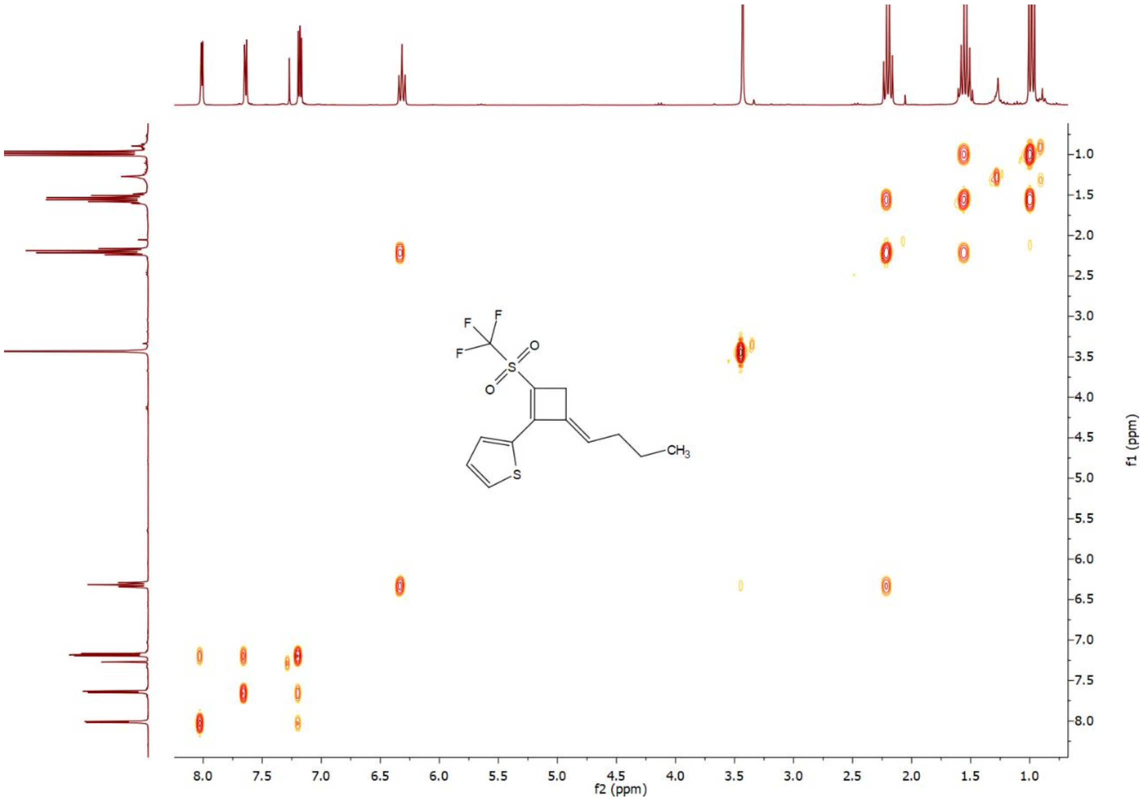

HSQC NMR compound **7a** ($CDCl_3$, 700 MHz/176 MHz, 25 °C)

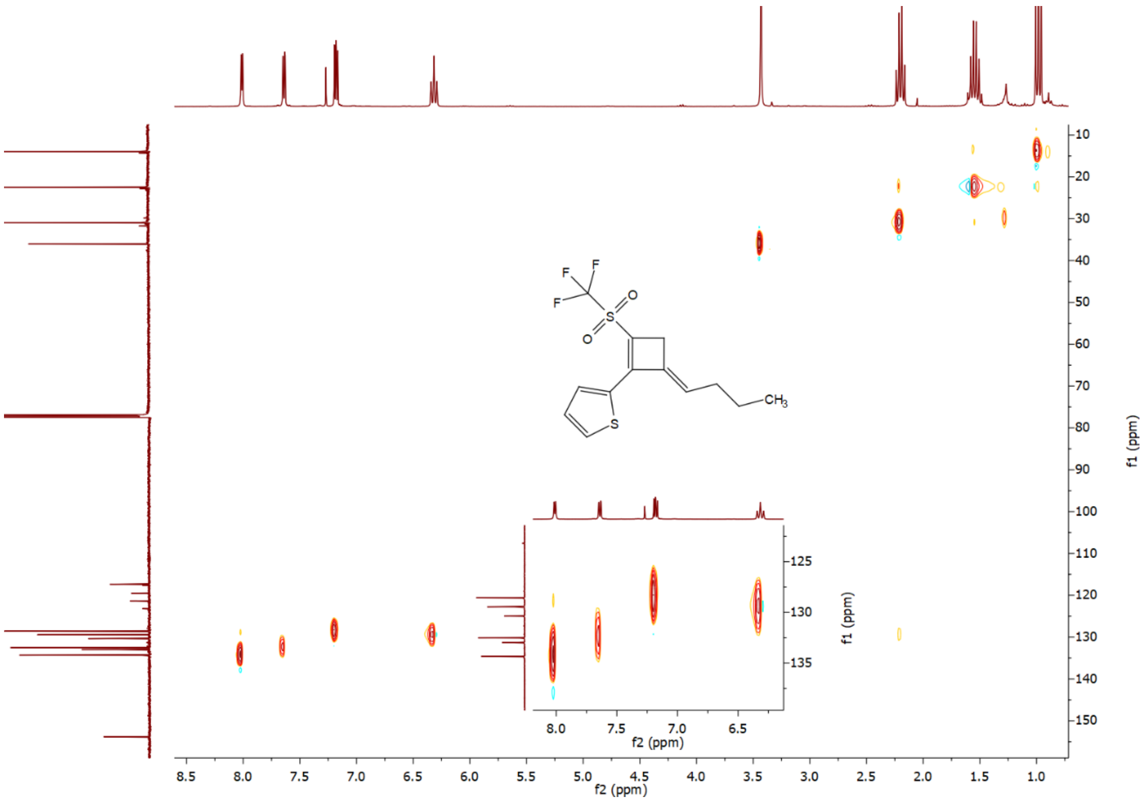

HMBC NMR compound **7a** ($CDCl_3$, 700 MHz/176 MHz, 25 °C)

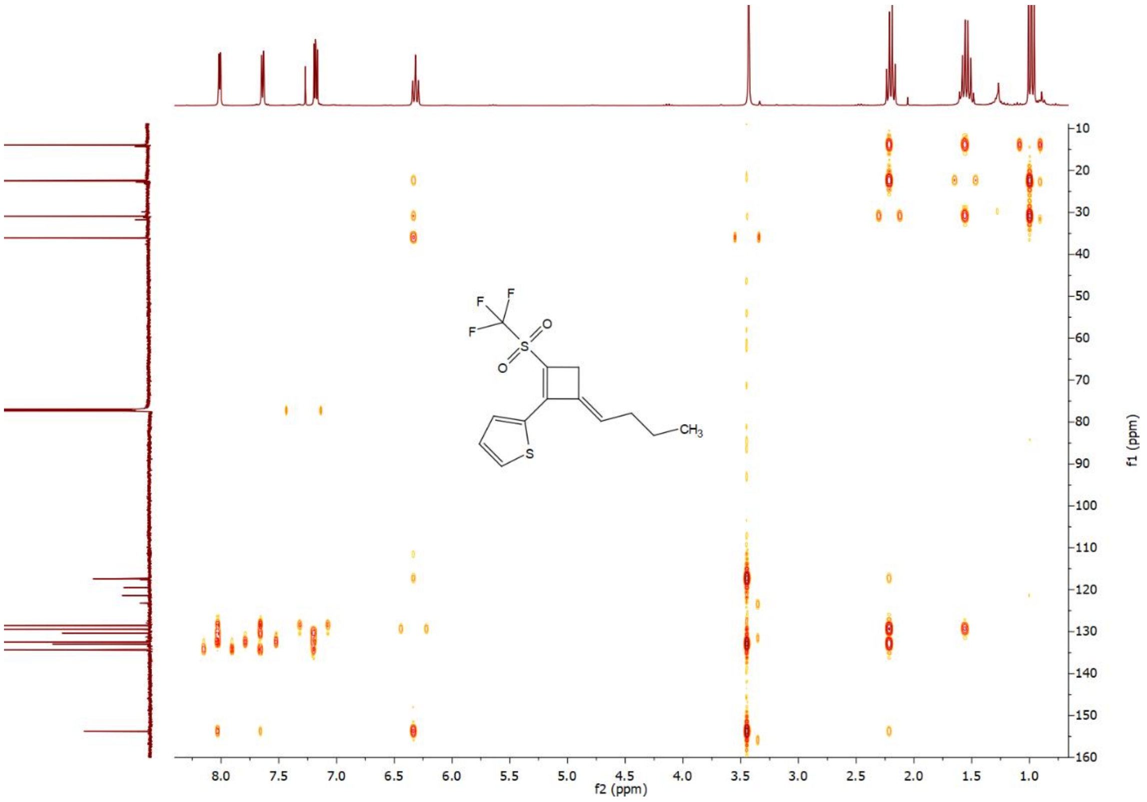

Supplement: Supplementary file 1 — ol4c01514_si_001.pdf [file ol4c01514_si_001.pdf]
